# Development and validation of a Radiopathomics model based on CT scans and whole slide images for discriminating between Stage I-II and Stage III gastric cancer

**DOI:** 10.1186/s12885-024-12021-2

**Published:** 2024-03-22

**Authors:** Yang Tan, Li-juan Feng, Ying-he Huang, Jia-wen Xue, Zhen-Bo Feng, Li-ling Long

**Affiliations:** 1 Department of Pathology, The First Affiliated Hospital of Guangxi Medical University, Nanning, Guangxi China; 2 Department of Radiology, The First Affiliated Hospital of Guangxi Medical University, Nanning, Guangxi China; 3 Department of Pathology, The First Affiliated Hospital of Guangxi University of Chinese Medicine, Nanning, Guangxi China; 4 Key Laboratory of Early Prevention and Treatment for Regional High Frequency Tumor, Gaungxi Medical University, Ministry of Education, Nanning, Guangxi China; 5 Guangxi Key Laboratory of Immunology and Metabolism for Liver Diseases, Nanning, Guangxi China

**Keywords:** Computed tomography, Whole slide image, Gastric Cancer, Radiopathomics, Radiomics, Pathomics, Machine learning

## Abstract

**Objective:**

This study aimed to develop and validate an artificial intelligence radiopathological model using preoperative CT scans and postoperative hematoxylin and eosin (HE) stained slides to predict the pathological staging of gastric cancer (stage I-II and stage III).

**Methods:**

This study included a total of 202 gastric cancer patients with confirmed pathological staging (training cohort: *n* = 141; validation cohort: *n* = 61). Pathological histological features were extracted from HE slides, and pathological models were constructed using logistic regression (LR), support vector machine (SVM), and NaiveBayes. The optimal pathological model was selected through receiver operating characteristic (ROC) curve analysis. Machine learnin algorithms were employed to construct radiomic models and radiopathological models using the optimal pathological model. Model performance was evaluated using ROC curve analysis, and clinical utility was estimated using decision curve analysis (DCA).

**Results:**

A total of 311 pathological histological features were extracted from the HE images, including 101 Term Frequency-Inverse Document Frequency (TF-IDF) features and 210 deep learning features. A pathological model was constructed using 19 selected pathological features through dimension reduction, with the SVM model demonstrating superior predictive performance (AUC, training cohort: 0.949; validation cohort: 0.777). Radiomic features were constructed using 6 selected features from 1834 radiomic features extracted from CT scans via SVM machine algorithm. Simultaneously, a radiopathomics model was built using 17 non-zero coefficient features obtained through dimension reduction from a total of 2145 features (combining both radiomics and pathomics features). The best discriminative ability was observed in the SVM_radiopathomics model (AUC, training cohort: 0.953; validation cohort: 0.851), and clinical decision curve analysis (DCA) demonstrated excellent clinical utility.

**Conclusion:**

The radiopathomics model, combining pathological and radiomic features, exhibited superior performance in distinguishing between stage I-II and stage III gastric cancer. This study is based on the prediction of pathological staging using pathological tissue slides from surgical specimens after gastric cancer curative surgery and preoperative CT images, highlighting the feasibility of conducting research on pathological staging using pathological slides and CT images.

**Supplementary Information:**

The online version contains supplementary material available at 10.1186/s12885-024-12021-2.

## Introduction

Gastric cancer, one of the common digestive tract tumors, ranks fifth globally and third in China among newly diagnosed cases of gastric cancer [1, 2]. The diagnosis rate of early gastric cancer is relatively low, with most patients being diagnosed at an advanced stage [3]. Consequently, gastric cancer has a high mortality rate and ranks third among malignancy-related deaths [1]. With an aging population and the potential increase in cases of gastric cancer among younger individuals due to economic growth, gastric cancer is likely to remain a significant concern [3].

Currently, the pathological staging of gastric cancer is mainly based on the recognized American Joint Committee on Cancer (AJCC)/International Union Against Cancer (UICC) TNM (Tumor-Lymph Node-Metastasis) 8th edition staging manual, which includes Stage I, Stage II, Stage III, and Stage IV [4–7]. Compared to advanced gastric cancer (Stages III and IV), early-stage gastric cancer (Stages I and II) typically has a better prognosis. Cohort studies in both Western and Asian populations have shown a negative correlation between the stage of gastric cancer and prognosis, indicating that Stage I-II gastric cancer has a better prognosis than Stage III-IV [8–10]. Therefore, early identification of early-stage gastric cancer and proactive treatment can significantly improve the cure rate [11]. Gastric cancer is primarily treated with comprehensive therapies, with surgery being the mainstay [12, 13], and accurate pathological staging is crucial for diagnosis, treatment, and prognosis.

Pathological staging of gastric cancer entails meticulously examining tissue morphology in postoperative specimens, encompassing an exhaustive evaluation of the tumor, lymph nodes, and clinical metastasis. However, this process often necessitates specialized medical professionals and technicians for microscopic analysis, which may be subject to subjective interpretation, labor-intensive, and time-consuming, thereby presenting inherent limitations. Consequently, there is a pressing imperative for the integration of artificial intelligence into the prediction of gastric cancer pathological staging. This initiative aims to enhance diagnostic efficiency, accuracy, and consistency while concurrently reducing healthcare costs for patients.

In addition to postoperative pathological staging, gastric cancer commonly utilizes independent staging systems including pathological staging after neoadjuvant therapy and resection, as well as preoperative clinical staging [5]. Preoperative clinical staging heavily relies on CT imaging. CT is a commonly used diagnostic tool for preoperative diagnosis and staging of gastric cancer. However, there is a certain discrepancy between preoperative clinical staging based on CT assessment and the actual postoperative pathological staging. Studies by Zhao et al. and Feng et al. reported overall accuracies of 66.7% and 67.2%, respectively, for CT-based preoperative gastric cancer staging [14, 15]. At this point, the emergence of radiomics presents a promising role.

Radiomics converts various imaging modalities, such as CT and MRI, into high-dimensional data and has shown significant potential through machine learning techniques and clinical models in predicting histological classification, treatment response, and prognosis [16–20]. Some studies have reported the potential value of CT in differentiating between T2 and T3/4 stages of gastric cancer (based on tumor invasion depth) [19]. Additionally, CT image radiomics has shown good predictive capabilities for lymph node metastasis in gastric cancer, suggesting its potential for personalized prediction of gastric cancer lymph node metastasis [18, 21]. However, gastric cancer pathological staging is based on the comprehensive analysis of tumor invasion depth (T), lymph node metastasis (N), and distant metastasis (M). A few researchers have analyzed the correlation between CT volume or texture and pathological staging of gastric cancer [22, 23], suggesting a certain association between CT and pathological staging, indicating significant potential for predicting gastric cancer pathological staging through CT.

Furthermore, machine learning has gradually been applied in the field of gastric cancer pathology, bringing new possibilities to pathology research [24, 25]. In gastric cancer, machine learning based on low-cost HE-stained slides accurately classifies different types of gastric cancer, predicts driver gene mutations, and microsatellite instability, among other potential values [3]. However, to date, there has been limited research that integrates these two fields to develop a reliable model for accurately predicting the pathological staging of gastric cancer. This study represents the first attempt to utilize a variety of machine learning algorithms combined with histopathological and radiological features to develop multiple radiopathological models for predicting the pathological staging of gastric cancer.

Therefore, the objective of this study is to develop and validate a radiopathological model that integrates histopathological and radiological features for accurately predicting the pathological staging of gastric cancer, particularly in distinguishing between Stage I-II and Stage III patients. The specific research question primarily focuses on whether machine learning algorithms can be employed to construct a reliable model for accurately predicting the pathological staging of gastric cancer based on histopathological and radiological features.

## Materials and methods

### Patient population

**Fig. 1:**
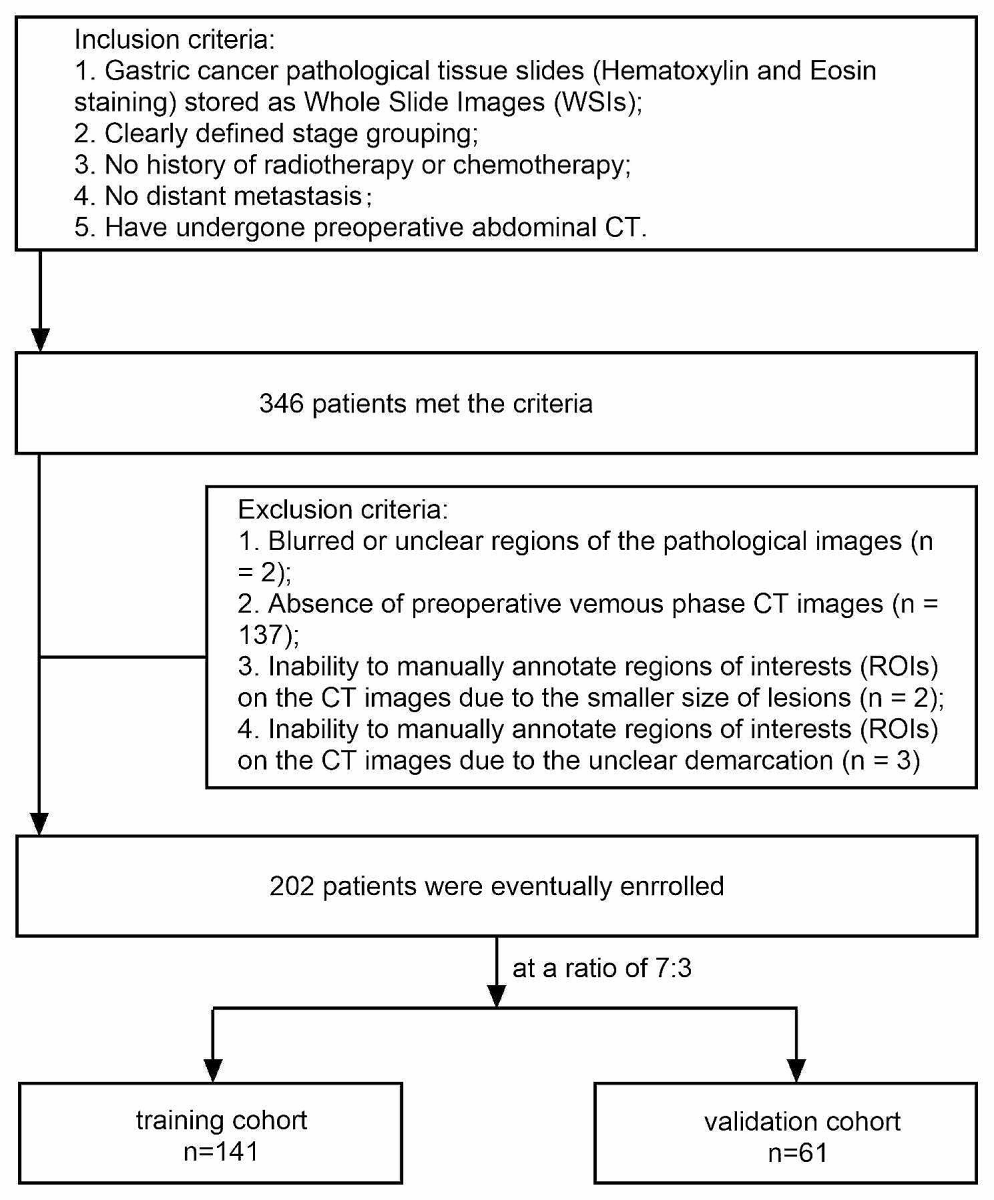
The flowchart for selecting the study patients

Ethical approval for this study was obtained from the Medical Ethics Committee of the First Affiliated Hospital of Guangxi Medical University (China). The approval notice, with reference number 2023-E398-01. Inclusion criteria were as follows: (1) Gastric cancer pathology tissue slides stored in the form of digital pathology whole slide images (WSI) stained with hematoxylin and eosin (HE); (2) Clear pathological staging; (3) No history of preoperative chemotherapy or radiation therapy; (4) No metastasis; (5) Have undergone preoperative abdominal CT. Exclusion criteria were as follows: (1) Blurriness in parts or all of the pathological slides; (2) Lack of preoperative venous phase CT images; (3) Inability to manually annotate ROI on CT images can be due to significant difficulties arising from the small size of early gastric cancer lesions or the unclear demarcation between the lesions and the surrounding normal boundaries, making precise annotation challenging. Data from a total of 202 gastric cancer patients who underwent curative surgical resection at our institution were included. Patients were randomly divided into training and validation cohorts in a 7:3 ratio. Each pathology slide and CT image corresponded to a patient. Pathology slides and CT images were allocated to patients in a 7:3 ratio. The pathological tissue slides are derived from surgical specimens of curative gastric cancer resection, and the CT images are obtained from preoperative examinations for curative gastric cancer surgery. The flowchart for selecting the study patients is shown in Fig. 1.

### Image acquisition

**Fig. 2:**
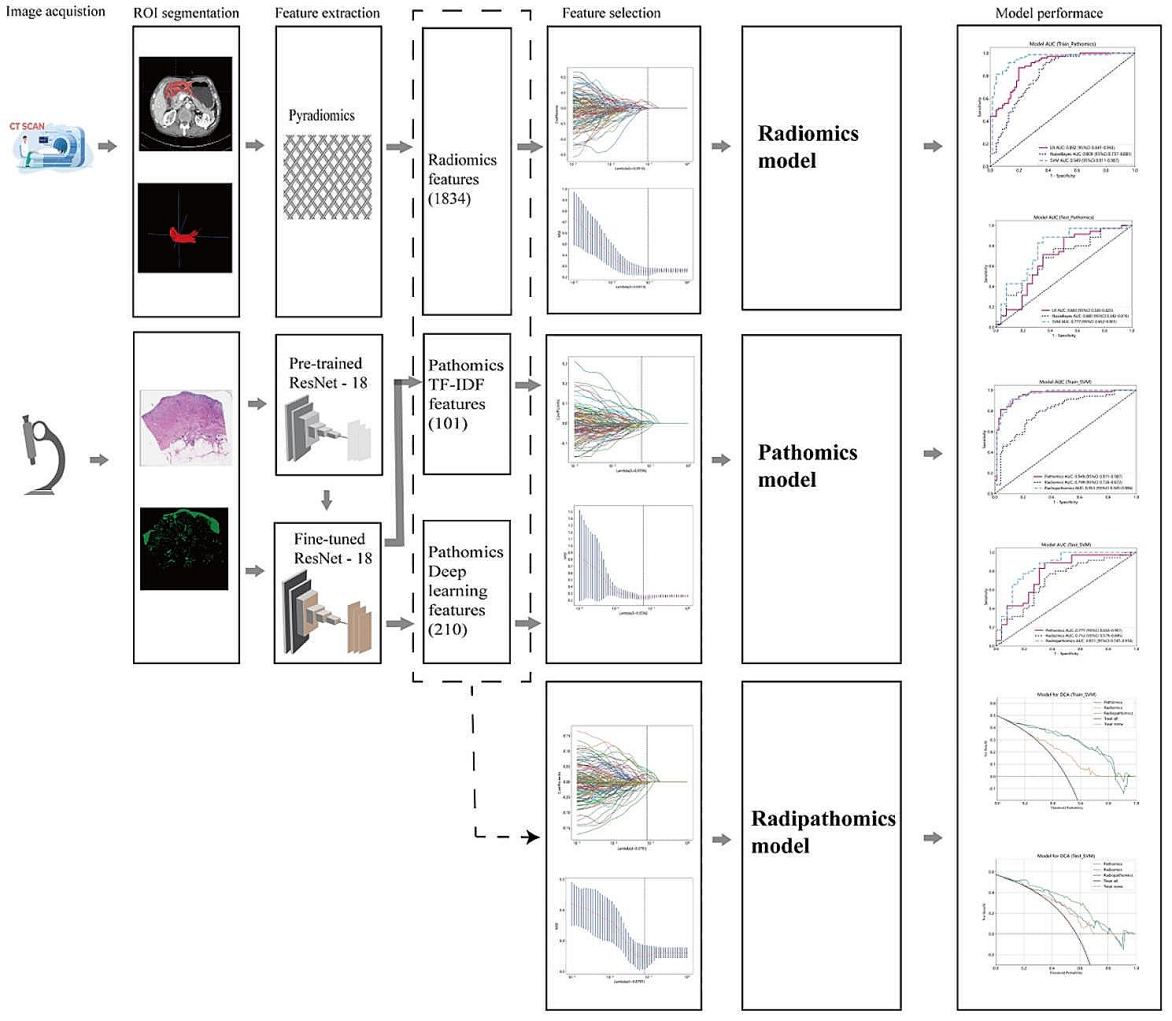
The workflow of radiomics, pathomics and radiopathomics models in this study ROI, Region of Interest; TF-IDF, Term Frequency-Inverse Document Frequency

The workflow of radiomics, pathomics, and radiopathomics models in this study is presented in Fig. 2. CT images of patients were acquired using three instruments, including a 64-channel CT scanner (LightSpeed VCT, GE Healthcare), a 256-channel CT scanner (Revolution, GE Healthcare), and a dual-source CT scanner (SOMATOM Definition Flash, Siemens Healthcare). The collected CT images were obtained during the venous phase. Pathological tissue slides were scanned using a slide scanner provided by Shenzhen Shengqiang Technology Co., Ltd. (http://www.sqray.com/product/list) at a 20x magnification.

### Image segmentation

All obtained CT images were in DICOM format. The window width was set to 300 Hounsfield Units (HU), and the window level was set to 50 HU. Pixel spacing was standardized to 1 mm through resampling (linear interpolation technique). Two experienced radiologists (with 5 and 8 years of diagnostic experience) manually segmented regions of interest (ROI) using ITK-SNAP software (version 4.0.1, http://www.itksnap.org/). An intraclass correlation coefficient (ICC) of ≥ 0.75 indicated robust results.

The acquired digital pathology images were in SDPC format and needed to be converted to TIF or SVS format for further QuPath standardization. QuPath, an open-source software for digital pathology image analysis (version 0.4.3, https://qupath.github.io/), was used to annotate tumor regions in pathological whole slide images (WSI) [26]. Subsequently, the annotated WSIs were segmented into patches measuring 512 × 512 pixels, with each patch representing a 20x magnification level. Reinhard standardization was applied to transform color channels of patches to approximate a predefined standard color distribution, thus preprocessing the pathological slides [27]. For both the ROI annotation of CT images and the ROI annotation of pathological whole-slide images, all tumor regions were identified.

### Feature extraction and selection

Traditional image features were extracted using the internal feature analysis program of Pyradiomics (http://pyradiomics.readthedocs.io) [28]. Extracted features included pixel grayscale values reflecting tissue density obtained directly from the original CT images, gradient values describing edge and contour information of the images, texture features including Gray-Level Co-occurrence Matrix (GLCM) and Gray-Level Run Length Matrix (GLRLM), and shape features representing objects or regions in the images. In addition to the features directly obtained from CT, a logarithmic transformation was applied to the sigma parameter, associated with wavelet transform, scale-space, or other feature extraction methods, to enhance the extraction of texture features from the images.

Standardized pathological patches underwent transfer learning on ResNet-18 pre-trained on the ImageNet dataset to build a deep learning model for distinguishing tumors from non-tumorous regions within patches. Model training employed the stochastic gradient descent optimization algorithm to adjust the weights and biases of the deep learning model, further minimizing the cross-entropy loss function. The model was trained to better predict labels of samples by minimizing the cross-entropy loss function. A learning rate of 0.01 and the adaptive moment estimation optimizer were implemented for 3 epochs with a batch size of 128. The progress of DL model training was observed. After training completion, Term Frequency-Inverse Document Frequency (TF-IDF) was used to extract features from patches’ prediction results [29]. The reason TF-IDF technique was chosen to extract features from the prediction results of the deep learning model lies in its successful application in the field of text mining and its ability to evaluate the importance of keywords. In our study, we applied TF-IDF technique to the prediction results of the deep learning model with the aim of extracting key features for further analysis and understanding of pathological information in images. During the transfer learning process, parameters of the ResNet-18 model were adjusted. The penultimate layer of the modified ResNet-18 was then used to extract features from image blocks in both the training and validation datasets.

Both extracted pathological features and CT image features underwent the following operations: first, z-score normalization (mean = 0, standard deviation = 1) was applied to standardize each feature to conform to a standard normal distribution. Then, Spearman rank correlation coefficient was utilized for statistical analysis to measure the correlation between two variables. When the Spearman correlation coefficient between features was > 0.9, one of the highly correlated features was retained. This method employs a “greedy approach.” It selects the most redundant feature at each step to retain, aiming to minimize the correlation between features and thus enhance the model’s generalization ability and performance. Finally, feature dimensionality reduction was carried out using L1 regularization and the Least Absolute Shrinkage and Selection Operator (LASSO) regression to select strongly correlated features, resulting in a sparse model where only a few features significantly contributed to the prediction results, enhancing model interpretability and generalization.

### Development and validation of models

The final selected features were used for model construction. Our study employed three mainstream machine learning algorithms, including logistic regression (LR), support vector machine (SVM), and Naive Bayes. Models were constructed separately for pathomics features, radiomics features, and radiopathomics features. Class imbalance was considered when computing metrics. Metrics such as the area under the curve (AUC), accuracy, sensitivity, specificity, positive predictive value (PPV), and negative predictive value (NPV) were calculated. Sensitivity and specificity were defined with the positive class being defined as stage III gastric cancer. Model performance was compared using a comprehensive analysis of AUC values, delong’s test, and decision curve analysis (DCA).

### Statistical analysis

Clinical baseline features were subjected to t-tests, chi-square tests, or Fisher’s exact tests using SPSS software (version 25.0, IBM). T-tests were applied to continuous variables with homoscedasticity, represented as x ± s, while chi-square tests or Fisher’s exact tests were used for categorical variables, represented as ratios. A two-tailed p-value < 0.05 indicated statistical significance. ICC, Spearman rank correlation tests, z-score normalization, Delong tests, and LASSO regression analysis were conducted using Python software Receiver operating characteristic (ROC) curves and clinical decision curves were plotted. The study utilized an Intel Core i7-13700KF CPU, an NVIDIA GeForce RTX 4070Ti GPU with CUDA 12.2.79, and 64GB DDR4 memory for machine learning tasks, with Python 3.7.12, scikit-learn 1.0.2, and Jupyter Notebook 6.5.4 forming the software environment.

## Results

### Clinical and pathological characteristics of patients

This study included 202 cases of gastric cancer, comprising 125 males and 77 females. The cohort was divided into a training cohort of 141 patients and a validation cohort of 61 patients. Statistical analysis between the two sets showed no significant differences in age, sex, tumor location, differentiation grade, and Stage Groupings (categorized into Stage I– II and Stage III). Additionally, it is worth mentioning that GURZU et al. proposed a new Dukes-MAC-like staging system, which has demonstrated potential prognostic and predictive value. It could improve postoperative treatment strategies for gastric cancer, especially in early-stage patients [30]. This study incorporated it into the baseline statistical analysis, and its distribution in the two cohorts showed no significant differences. The clinical and pathological characteristics of the patients are summarized in Table 1.

**Table 1:** Clinical and pathological characteristics in the training and validation cohorts

Characteristics	Training Cohort (*n* = 141)	Validation Cohort (*n* = 61)	*P*
Age, mean ± SD	55.1 ± 12.076	55.25 ± 10.779	0.935
Sex, n (%)			0.346
Male	84 (59.6)	41 (67.2)	
Female	57 (40.4)	20 (32.8)	
Location, n (%)			0.467
Antrum	88 (62.4)	37 (60.7)	
Non-Antrum	53 (37.6)	24 (39.3)	
Differentiation degree, n (%)			0.87
Well differentiated	5(3.5)	2 (3.3)	
Moderately differentiated	39 (27.7)	14 (23.0)	
Poorly differentiated	96 (68.8)	45 (73.8)	
Stage Groupings for pTNM, n (%)			0.196
Stage I - II	71 (50.4)	26 (42.6)	
Stage III	70 (49.6)	35 (57.4)	
Dukes-MAC-like staging system, n (%)			0.898
Stage A1 (T1N0)	21 (14.9)	6 (9.8)	
Stage A2 (T1N1-3)	4 (2.8)	4 (6.6)	
Stage B1 (T2N0)	8 (5.7)	4 (6.6)	
Stage B2 (T2N1-3)	14 (9.9)	7 (11.5)	
Stage C1 (T3N0)	7 (5.0)	0 (0.0)	
Stage C2 (T3N1-3)	19 (13.5)	4 (6.6)	
Stage D (T4N0-3)	68 (48.2)	36 (59.0)	

### Feature selection for models

**Fig. 3:**
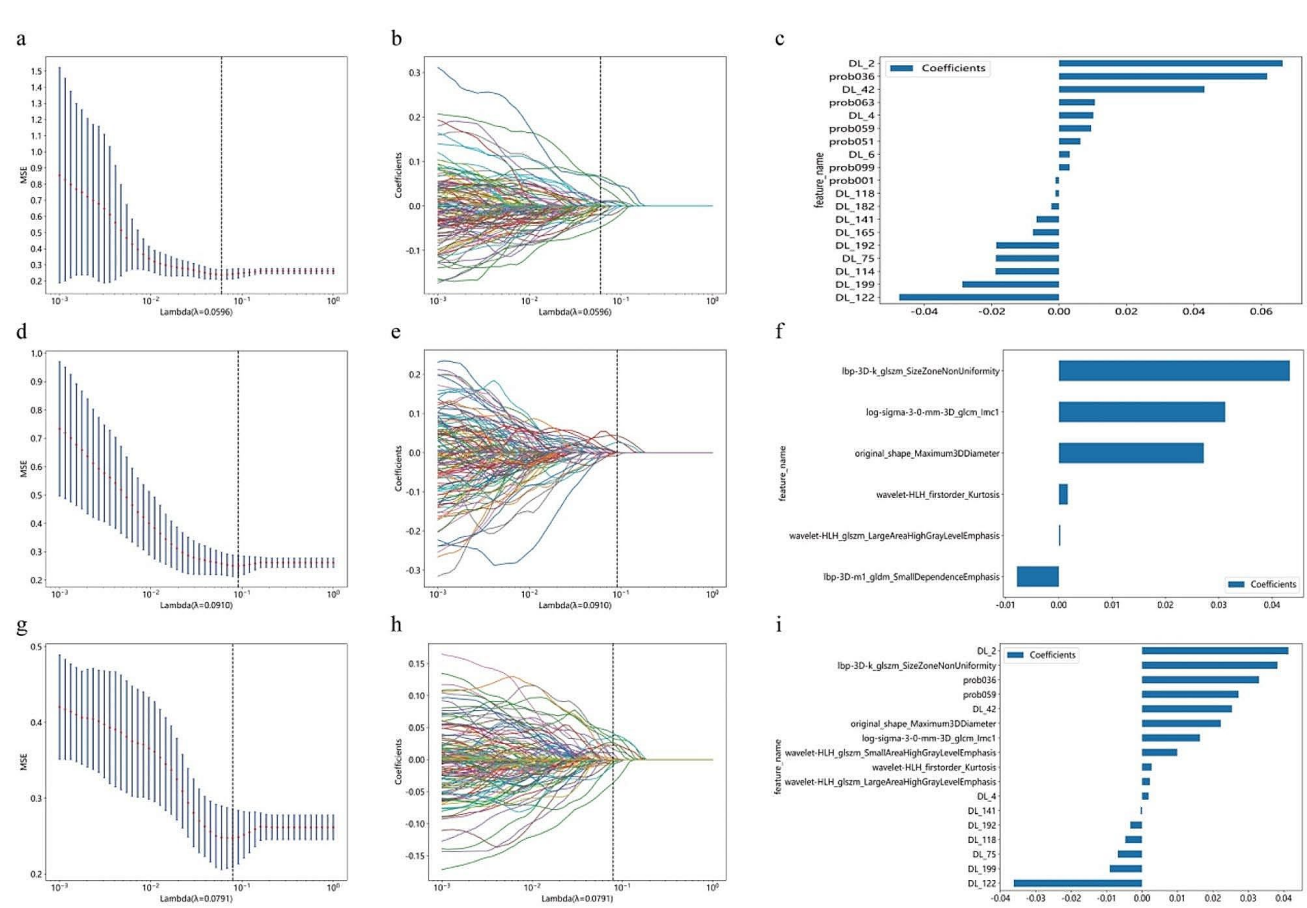
The procedure of feature selection utilizing the Least Absolute Shrinkage and Selection Operator (LASSO) regression model. The features with non-zero coefficients retained after selection. Feature selection for pathomics (**a-c**); Feature selection for radiomics (c-f); Feature selection for radiopathomics (**g-i**). Optimal λ values are chosen based on 10-fold cross-validation and minimum Mean Squared Error (MSE), represented by vertical dashed lines (**a, d, g**). Display LASSO coefficients for different λ values, where vertical dashed lines indicate the number of features corresponding to the optimal λ value (**b, e, h**). Following the application of LASSO regression for feature selection, exclusively those features exhibiting non-zero coefficients were retained (**c, f, i**)

During the feature selection process, we selected the λ value with the minimum Mean Squared Error (MSE) (Fig. 3a, d, g) and fitted a Lasso regression model based on the optimal λ value (Fig. 3b, e, h). The pathological features comprised two parts: 210 deep learning features (see Appendix 1) and 101 Term Frequency-Inverse Document Frequency (TF-IDF) features (see Appendix 2). After feature dimensionality reduction, a final selection of 19 pathomics features was made (Fig. 3c). Simultaneously, the 1834 radiomics features (see Appendix 3) were reduced to 6 features with non-zero coefficients (Fig. 3f). Finally, both sets of features were merged, resulting in a total of 2145 features (combining 311 pathomics features with 1834 radiomics features), which were further reduced to 17 radiopathomics features through dimensionality reduction (Fig. 3i).

### Construction of pathomics, radiomics, and radiopathomics models

The pathomics features obtained through the aforementioned selection process were utilized to construct the pathomics models, radiomics features were employed for constructing the radiomics models, and radiopathomics features were used in building the radiopathomics models. In total, 9 models were established: LR_Pathomics, NaiveBayes_Pathomics, SVM_Pathomics, LR_Radiomics, NaiveBayes_Radiomics, SVM_Radiomics, LR_Radiopathomics, NaiveBayes_Radiopathomics, and SVM_Radiopathomics.

### Validation of pathomics, radiomics, and radiopathomics models

**Fig. 4:**
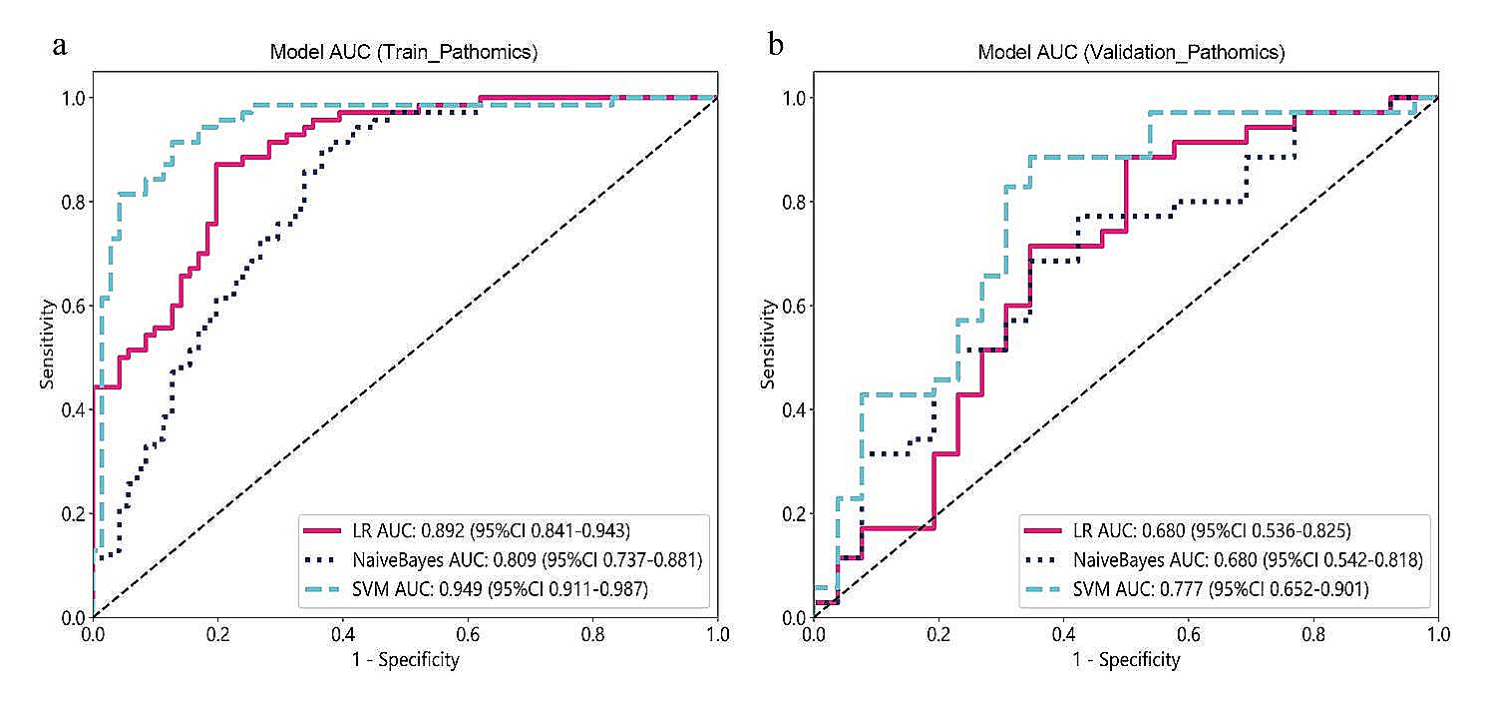
The receiver operating characteristic curves of the LR, NaiveBayes, and SVM in the training (**a**) and validation (**b**) cohorts, respectively LR, logistic regression; SVM, support vector machine; AUC area under the curve

The metrics for both the training and validation sets are displayed in Table 2. Among the pathomics models constructed using machine learning algorithms such as LR, NaiveBayes, and SVM, SVM_Pathomics exhibited the highest AUC values, both in the training cohort (0.949) and the validation cohort (0.777) (Table 2). The ROC curve results for the pathomics models in both the training and validation cohorts are depicted in Fig. 4. Delong tests were performed between every pair of models in the validation set of the pathological models (Table 3), indicating significant differences between the ROC curves of SVM_Pathomics and LR_Pathomics (*P* = 0.016) as well as NaiveBayes_Pathomics (*P* = 0.048), suggesting superior predictive performance of the SVM_Pathomics model compared to LR_Pathomics and NaiveBayes_Pathomics.

**Table 2:** Performance of models for predicting discrimination between stages I-II and stage III gastric cancer in training and validation cohorts

Model	Task	AUC (95% CI)	Accuracy	Sensitivity	Specificity	PPV	NPV	Precision	Recall	F1 score
LR_Pathomics	Training	0.892 (0.841–0.943)	0.837	0.871	0.803	0.813	0.864	0.813	0.871	0.841
	Validation	0.680 (0.536–0.825)	0.721	0.886	0.500	0.705	0.765	0.705	0.886	0.785
NaiveBayes_Pathomics	Training	0.809 (0.737–0.881)	0.766	0.914	0.620	0.703	0.880	0.703	0.914	0.795
	Validation	0.679 (0.540–0.818)	0.689	0.771	0.600	0.711	0.652	0.711	0.771	0.740
SVM_Pathomics	Training	0.949 (0.911–0.987)	0.894	0.914	0.873	0.877	0.912	0.877	0.914	0.895
	Validation	0.777 (0.652–0.901)	0.787	0.886	0.654	0.775	0.810	0.775	0.886	0.827
LR_Radiomics	Training	0.742 (0.663–0.824)	0.688	0.857	0.521	0.638	0.787	0.638	0.857	0.732
	Validation	0.720 (0.588–0.852)	0.705	0.743	0.654	0.743	0.654	0.743	0.743	0.466
NaiveBayes_Radiomics	Training	0.752 (0.673–0.831)	0.695	0.843	0.549	0.648	0.780	0.648	0.843	0.733
	Validation	0.733 (0.607–0.859)	0.705	0.714	0.692	0.758	0.643	0.758	0.714	0.735
SVM_Radiomics	Training	0.799 (0.726–0.873)	0.745	0.800	0.690	0.718	0.778	0.718	0.800	0.757
	Validation	0.712 (0.579–0.845)	0.705	0.771	0.615	0.730	0.667	0.730	0.771	0.750
LR_Radiopathomics	Training	0.904 (0.858–0.951)	0.830	0.914	0.746	0.780	0.898	0.780	0.914	0.842
	Validation	0.747 (0.617–0.878)	0.770	0.829	0.692	0.784	0.750	0.784	0.829	0.806
NaiveBayes_Radiopathomics	Training	0.861 (0.801–0.921)	0.787	0.857	0.718	0.750	0.836	0.750	0.857	0.800
	Validation	0.748 (0.624–0.872)	0.738	0.714	0.769	0.806	0.667	0.806	0.714	0.758
SVM_Radiopathomics	Training	0.953 (0.920–0.986)	0.901	0.914	0.887	0.889	0.913	0.889	0.914	0.901
	Validation	0.851 (0.745–0.956)	0.787	0.771	0.808	0.844	0.724	0.844	0.771	0.806

LR logistic regression, SVM support vector machine, AUC area under the curve, PPV positive prediction value, NPV negative prediction value

**Table 3:** Delong tests were performed between every pair of models in the same validation cohort

Model	Task		Delong Test	
LR_Pathomics	Validation		vs. NaiveBayes_Pathomics	*P* = 0.984
NaiveBayes_Pathomics	Validation		vs. SVM_Pathomics	*P* = 0.048
SVM_Pathomics	Validation		vs. LR_Pathomics	*P* = 0.016
LR_Radiomics	Validation		vs. NaiveBayes_Radiomics	*P* = 0.633
NaiveBayes_Radiomics	Validation		vs. SVM_Radiomics	*P* = 0.639
SVM_Radiomics	Validation		vs. LR_Radiomics	*P* = 0.837
LR_Radiopathomics	Validation		vs. NaiveBayes_Radiopathomics	*P* = 0.982
NaiveBayes_Radiopathomics	Validation		vs. SVM_Radiopathomics	*P* = 0.052
SVM_Radiopathomics	Validation		vs. LR_Radiopathomics	*P* = 0.013
SVM_Pathomics	Validation		vs. SVM_Radiomics	*P* = 0.439
SVM_Radiomics	Validation		vs. SVM_Radiopathomics	*P* = 0.058
SVM_Radiopathomics	Validation		vs. SVM_Pathomics	*P* = 0.036

LR logistic regression, SVM support vector machine

In the radiomics models constructed using LR, NaiveBayes, and SVM algorithms, the AUC values for LR_Radiomics, NaiveBayes_Radiomics, and SVM_Radiomics were 0.720, 0.733, and 0.712 (Table 2), respectively. According to the Delong test results (Table 3), there were no significant differences between any pair of models in the validation cohort of the radiomics models, indicating comparable model performance among LR_Radiomics, NaiveBayes_Radiomics, and SVM_Radiomics.

**Fig. 5:**
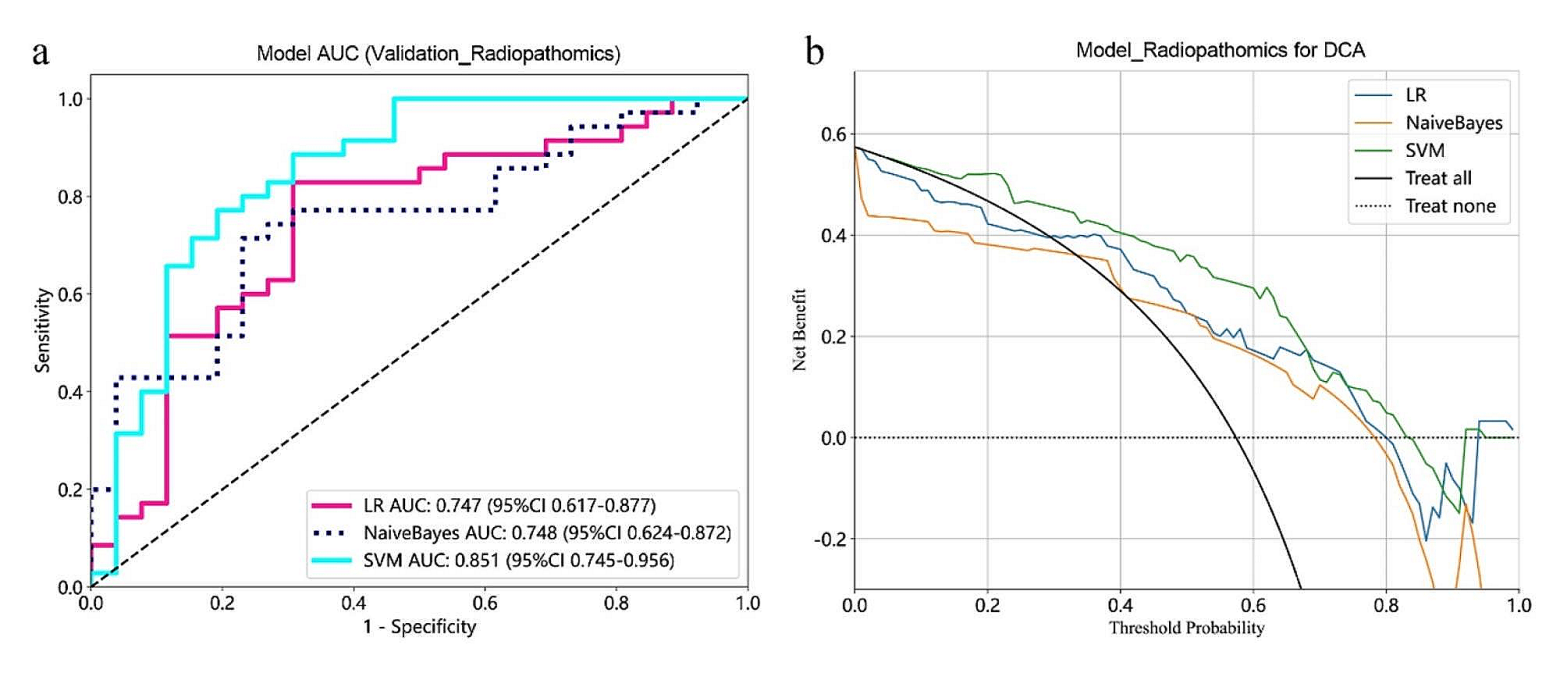
Receiver operating characteristic curves (**a**) and Decision Curve Analysis (DCA) (**b**) for Radiopathomics models based on LR, NaiveBayes, and SVM algorithms on the validation cohorts

For the radiopathomics models constructed using LR, NaiveBayes, and SVM algorithms, SVM_Radiopathomics exhibited higher AUC values in both the training cohort (0.953) and the validation cohort (0.851) (Table 2; Fig. 5a). The Delong test between SVM_Radiopathomics and LR_Radiopathomics yielded a *P*-value of 0.013 (Table 3), indicating a statistically significant difference in AUC values between them. However, the Delong test between SVM_Radiopathomics and NaiveBayes_Radiopathomics yielded a *P*-value of 0.052 (Table 3), suggesting a less significant difference in AUC values between them. In addition to AUC values and Delong tests, DCA is also an important indicator for evaluating model performance. The analysis of DCA results showed that the net benefit of SVM_Radiopathomics was superior to that of NaiveBayes_Radiopathomics (Fig. 5b). In summary, the predictive performance of the SVM_Radiopathomics model was better than that of LR_Radiopathomics and NaiveBayes_Radiopathomics.

### Comparison of SVM_Pathomics, SVM_Radiomics, and SVM_Radiopathomics models

**Fig. 6:**
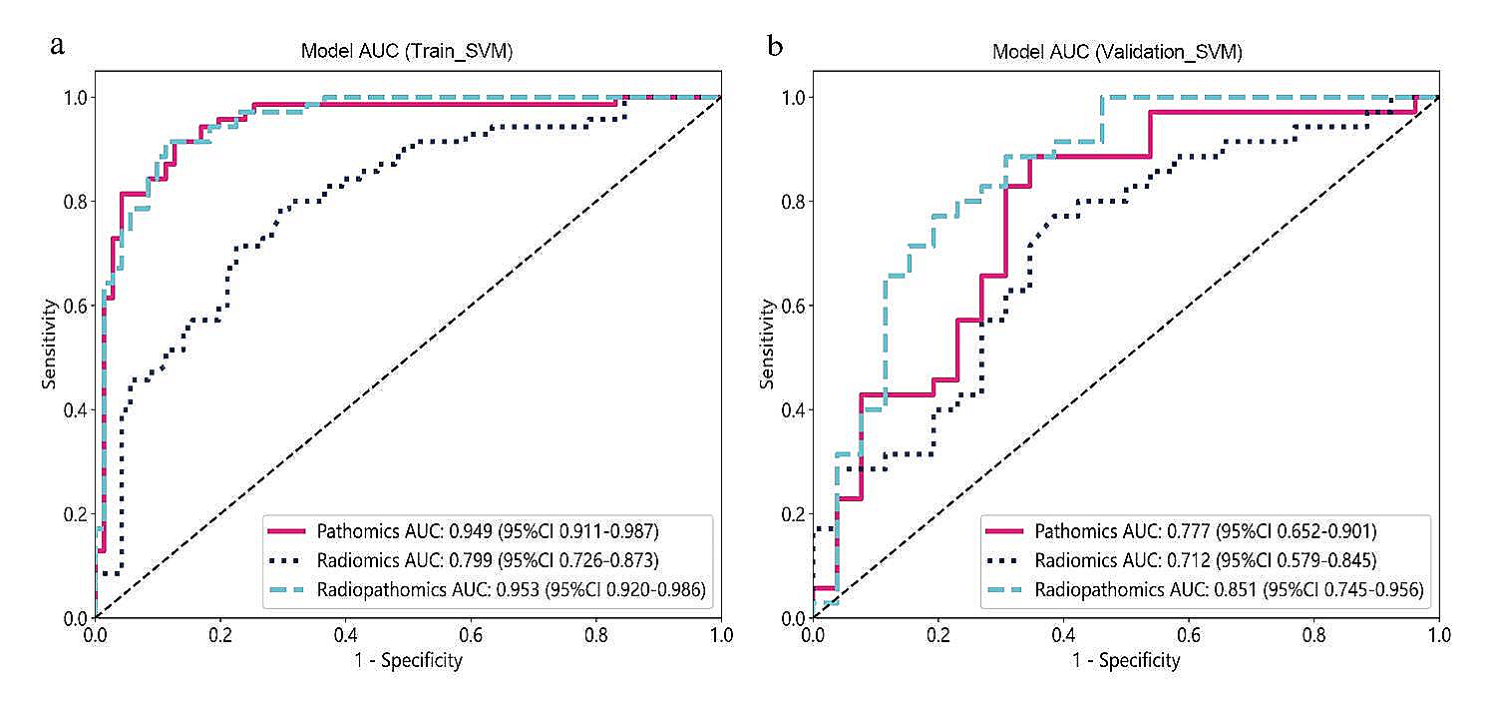
Receiver operating characteristic curves for the SVM-based pathomics model, radiomics model, and radiopathomics model were used to predict the discrimination between stages I-II and stage III gastric cancer in the training cohort (**a**) and the validation cohort (**b**) SVM, Support Vector Machine; AUC area under the curve

**Fig. 7:**
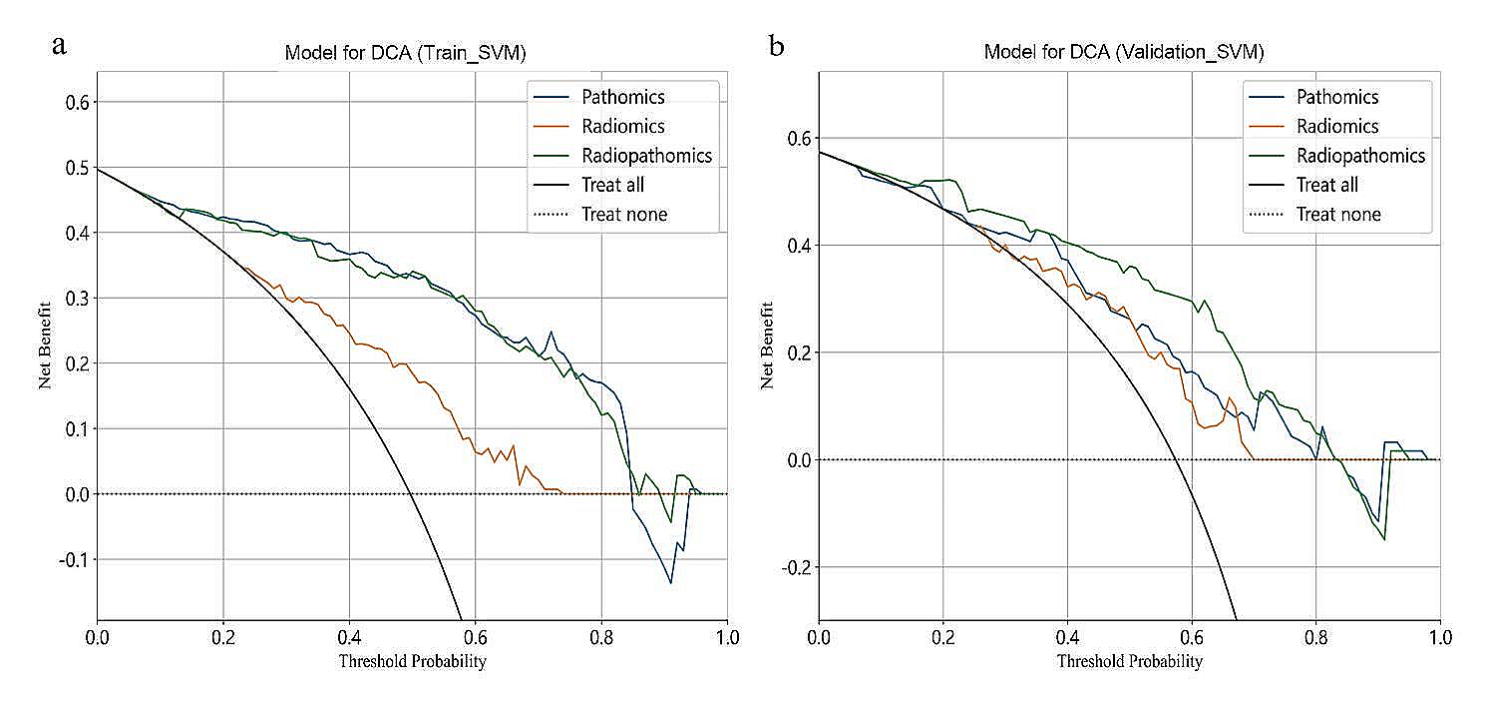
Decision Curve Analysis (DCA) for three models in the classification of stages I-II and stage III gastric cancer within the training (**a**) and validation (**b**) cohorts. The graphical representation clearly illustrates that the radiopathomics model yields the highest net benefit for both datasets

Considering that SVM algorithms generated the best SVM_Pathomics and SVM_Radiopathomics models, an objective evaluation of the discriminative performance of these three models was conducted. The ROC curves for these models in both the training and validation cohorts are shown in Fig. 6. In both cohorts, the AUC values of SVM_Radiopathomics were higher than those of SVM_Pathomics and SVM_Radiomics. Delong tests were performed between every pair of models in the same validation cohort, showing a *P*-value of 0.036 between SVM_Radiopathomics and SVM_Pathomics (Table 3), indicating a statistically significant difference in AUC between them. Although the Delong test between SVM_Radiopathomics and SVM_Radiomics yielded a *P*-value of 0.058 (Table 3), Decision Curve Analysis demonstrated that the net benefit of SVM_Radiopathomics was higher than that of SVM_Radiomics (Fig. 7b). In conclusion, compared to SVM_Pathomics and SVM_Radiomics, the performance of the SVM_Radiopathomics model was superior.

## Discussion

In this study, we developed radiopathomics models using LR, NaiveBayes, and SVM, integrating pathomics features based on pathological tissue slides with radiomics features from CT scans for the classification of stage I-II and stage III gastric cancer. The SVM_Radiopathomics model achieved promising results, demonstrating high predictive efficiency and robustness. This approach may represent a promising new method for assessing the pathological staging of gastric cancer.

While machine learning has tremendous potential in the field of pathomics, the application of AI in gastric cancer primarily focuses on tumor diagnosis, molecular prediction, and prognostic assessment [24, 31–33]. In this study, we developed pathomics models for predicting pathological staging based on digital HE-stained images of gastric cancer. The performance comparison of three pathomics models suggested that SVM_pathomics outperformed NaiveBayes_pathomics and LR_pathomics. The extracted pathomics data is likely to belong to a high-dimensional and nonlinear space. LR is suitable for linearly separable and non-separable problems; for complex data distributions and nonlinear problems, LR may not perform optimally. NaiveBayes is suitable for simple problems and high-dimensional text classification, with fast computation speed, but its assumption of conditional independence may limit its performance in certain cases. SVM is suitable for high-dimensional and nonlinear problems, exhibiting strong generalization capability.

Although the SVM_pathomics model in our study demonstrated good performance, there was a tendency towards overfitting. To mitigate overfitting and ensure the generalization ability of the final selected model on the test set, we implemented a series of measures. We employed 5-fold cross-validation to evaluate the model’s performance. By training and evaluating the model on multiple subsets, we gained a better understanding of its generalization ability and avoided overfitting on a single training set. By assessing the model’s performance on independent validation data, we could objectively evaluate its generalization ability and practical applicability. The primary reason for overfitting in the pathomics model may lie in the high dimensionality of the pathomics features, leading to good performance on the training set but poor performance on the validation set. Incorporating radiomics features can provide additional information, helping to reduce the model’s reliance on pathomics features and thus mitigate the risk of overfitting. Furthermore, combining radiomics and pathomics features may offer a more comprehensive and accurate feature representation, thereby improving the model’s generalization ability and reducing the likelihood of overfitting.

The radiopathomics model demonstrates good performance in predicting the pathological staging of gastric cancer, while machine learning based on the integration of pathomics and radiomics features has shown promise in other cancers. Wang et al. developed a combined radiomics and pathomics model to predict postoperative outcomes in colorectal cancer patients with lung metastasis. This combined radiomics-pathomics nomogram performed excellently in predicting overall survival and disease-free survival [34]. Wan et al. and Feng et al. similarly developed and validated comprehensive models that integrated radiomics and pathomics features for effective prediction of pathological good response in locally advanced rectal cancer patients after neoadjuvant chemoradiotherapy [35, 36]. In summary, the radiopathomics model is an approach that combines radiomics and pathomics data for analysis and prediction using machine learning and artificial intelligence techniques. The radiopathomics model holds significant potential in the medical field, providing deeper insights and support for clinical diagnosis, treatment, and research.

However, there are some limitations to our study. Firstly, this study is a single-center study, which may introduce potential result bias and requires validation with data from other centers. Secondly, the models we constructed did not incorporate clinical features such as tumor markers. Additionally, due to the limited data volume and the consideration of model balance, we set the outcome as a binary classification (stage I-II and stage III). In the future, multi-class prediction research can be considered as data expands. Lastly, the absence of comparable previous studies hindered our ability to effectively compare our radiopathomics model with other methods. Nonetheless, we remain vigilant about advancements in related fields, and should relevant reports emerge in the future, we will consider conducting comparative analyses.

In conclusion, our study proposed and validated a radiopathomics model based on pathological HE slides and CT images for distinguishing between stage I-II and stage III gastric cancer. In our study, the radiopathomics model based on the SVM algorithm exhibited the best classification performance. This approach may become a potential method for precision treatment and personalized medicine in gastric cancer.

### Electronic supplementary material

Below is the link to the electronic supplementary material.

Supplementary Material 1

## Data Availability

All datasets generated for this study are included in the article/Supplementary Material.
