# Correction: Development and validation of a Radiopathomics model based on CT scans and whole slide images for discriminating between Stage I-II and Stage III gastric cancer

**DOI:** 10.1186/s12885-024-12240-7

**Published:** 2024-04-15

**Authors:** Yang Tan, Li-juan Feng, Ying-he Huang, Jia-wen Xue, Zhen-Bo Feng, Li-ling Long

**Affiliations:** 1 Department of Pathology, The First Affiliated Hospital of Guangxi Medical University, Nanning, Guangxi China; 2 Department of Radiology, The First Affiliated Hospital of Guangxi Medical University, Nanning, Guangxi China; 3 Department of Pathology, The First Affiliated Hospital of Guangxi University of Chinese Medicine, Nanning, Guangxi China; 4 Key Laboratory of Early Prevention and Treatment for Regional High Frequency Tumor, Gaungxi Medical University, Ministry of Education, Nanning, Guangxi China; 5 Guangxi Key Laboratory of Immunology and Metabolism for Liver Diseases, Nanning, Guangxi China

**Correction: BMC Cancer 24, 368 (2024)**

10.1186/s12885-024-12021-2

Following publication of the original article [1], the authors noticed a typesetting error, whereby the equal contributions statement was erroneously omitted. The equal contributions statement should read: Yang Tan and Li-juan Feng have contributed equally to the study as co-first authors.

The original article [1] has been corrected.
