# Supplementary material for: Development and validation of a Radiopathomics model based on CT scans and whole slide images for discriminating between Stage I-II and Stage III gastric cancer
Source: BMC Cancer. 2024 Mar 22;24:368. doi: 10.1186/s12885-024-12021-2 (PMC10960497; doi:10.1186/s12885-024-12021-2)
Supplement: Supplementary file 1 — Supplementary Material 1 [file 12885_2024_12021_MOESM1_ESM.docx]

Appendix 1 After conducting transfer learning on pathological images, parameters of ResNet-18 were adjusted. The modified ResNet-18's penultimate layer extracted features from pathological image blocks in both the training and testing datasets. Each sample generated 210 distinct deep features, listed below and separated by the Tab key. Copying and pasting this into a blank Microsoft Excel document will allow for viewing.

ID DL_0 DL_1 DL_2 DL_3 DL_4 DL_5 DL_6 DL_7 DL_8 DL_9 DL_10 DL_11 DL_12 DL_13 DL_14 DL_15 DL_16 DL_17 DL_18 DL_19 DL_20 DL_21 DL_22 DL_23 DL_24 DL_25 DL_26 DL_27 DL_28 DL_29 DL_30 DL_31 DL_32 DL_33 DL_34 DL_35 DL_36 DL_37 DL_38 DL_39 DL_40 DL_41 DL_42 DL_43 DL_44 DL_45 DL_46 DL_47 DL_48 DL_49 DL_50 DL_51 DL_52 DL_53 DL_54 DL_55 DL_56 DL_57 DL_58 DL_59 DL_60 DL_61 DL_62 DL_63 DL_64 DL_65 DL_66 DL_67 DL_68 DL_69 DL_70 DL_71 DL_72 DL_73 DL_74 DL_75 DL_76 DL_77 DL_78 DL_79 DL_80 DL_81 DL_82 DL_83 DL_84 DL_85 DL_86 DL_87 DL_88 DL_89 DL_90 DL_91 DL_92 DL_93 DL_94 DL_95 DL_96 DL_97 DL_98 DL_99 DL_100 DL_101 DL_102 DL_103 DL_104 DL_105 DL_106 DL_107 DL_108 DL_109 DL_110 DL_111 DL_112 DL_113 DL_114 DL_115 DL_116 DL_117 DL_118 DL_119 DL_120 DL_121 DL_122 DL_123 DL_124 DL_125 DL_126 DL_127 DL_128 DL_129 DL_130 DL_131 DL_132 DL_133 DL_134 DL_135 DL_136 DL_137 DL_138 DL_139 DL_140 DL_141 DL_142 DL_143 DL_144 DL_145 DL_146 DL_147 DL_148 DL_149 DL_150 DL_151 DL_152 DL_153 DL_154 DL_155 DL_156 DL_157 DL_158 DL_159 DL_160 DL_161 DL_162 DL_163 DL_164 DL_165 DL_166 DL_167 DL_168 DL_169 DL_170 DL_171 DL_172 DL_173 DL_174 DL_175 DL_176 DL_177 DL_178 DL_179 DL_180 DL_181 DL_182 DL_183 DL_184 DL_185 DL_186 DL_187 DL_188 DL_189 DL_190 DL_191 DL_192 DL_193 DL_194 DL_195 DL_196 DL_197 DL_198 DL_199 DL_200 DL_201 DL_202 DL_203 DL_204 DL_205 DL_206 DL_207 DL_208 DL_209

sample_01 -6.051733132 -0.541134815 1.035103813 -0.860154454 -0.411204778 -0.911991634 -0.882903338 -0.121513767 -0.176395547 0.297687044 0.561687022 -0.344707693 1.629086306 -0.41790439 -0.303221389 -0.096458222 0.47868314 -0.1109184 0.290683878 0.407976874 -0.11075023 -0.280775014 -0.265773164 0.11088229 0.027931109 -0.032214658 -0.831914149 -0.282751472 0.391312756 -0.461251542 0.59877925 -0.306181259 -0.324065873 -0.286732353 0.156757981 -0.192393491 -0.254400598 -0.341869681 -0.129499123 -0.193739978 -0.083750399 0.027798372 0.273955306 -0.037101463 0.051427325 -0.090622216 0.147604337 0.07312445 0.116358756 -0.085586592 0.039518571 -0.144376242 -0.059574573 -0.175343893 -0.18581895 0.120766574 0.056906347 0.270472518 0.211471938 0.125868604 0.30910942 0.006091814 0.16543368 -0.141959773 0.039977632 0.021466592 -0.007575947 -0.099529316 0.165371435 -0.226770046 0.067589699 0.00575654 -0.012187267 0.089296463 0.118933633 -0.216341498 -0.049918493 0.175719035 -0.073295234 -0.001618987 -0.043067719 -0.052507971 0.210424933 -0.185894836 -0.20471526 -0.094425168 -0.03908332 0.025840114 -0.125169853 -0.00690339 0.046653944 0.021657316 0.053386545 -0.020164608 0.246615401 0.006573175 0.022074377 0.075789409 0.108564467 -0.004726464 0.091155841 0.006658992 -0.052813161 -0.041258434 0.004717708 -0.015125766 0.252501026 0.144700106 0.156583929 0.092903499 0.024854852 -0.02554383 -0.054841329 -0.018773191 -0.133721425 -0.111728951 -0.03950964 0.080585757 0.094255654 0.066550826 0.128346306 0.10122956 -0.01465603 0.089015805 0.038224121 -0.098293874 0.010170275 0.116693859 0.014388789 -0.066342724 -0.061459745 0.060400071 -0.108076577 -0.038906414 -0.084543929 0.012879931 0.026857922 0.005661969 -0.00931691 -0.063581932 0.062624963 0.002432487 -0.030710449 -0.143434166 -0.092538184 0.011512347 -0.033707698 0.033588034 0.038571921 -0.065146918 -0.01668413 -0.034969757 0.103106336 -0.050572858 0.041979432 -0.041306752 0.021310905 -0.039762648 -0.000889063 0.013503927 0.061940426 0.043658072 0.031545051 0.027472452 0.002953987 0.021761926 -0.07638235 0.019271147 -0.029785204 -0.009253528 -0.03105313 -0.077921347 -0.054950605 -0.064931981 0.042784696 0.034218481 -0.045446014 -0.036391256 0.027480489 0.001861841 0.093188263 -0.037428691 -0.010585594 -0.066766208 -0.083471243 -0.012358919 0.015691313 0.057462877 0.021672126 -0.05844881 -0.070104389 -0.022439749 -0.033733433 0.050575134 0.002413134 -0.031849921 0.004676592 0.017467605 0.017486679 0.014724005 -0.021914858 -0.015546823 -0.070276015 0.020868543 -0.031942516 -0.033232271 -0.0115785 -0.018106193 0.042863021 -0.015887193

sample_02 4.859105686 1.805553523 2.763217435 -1.336763163 -0.865081081 -1.575752368 -1.151032738 0.493844287 -0.084592776 -1.043699554 0.861734938 -0.427778156 0.621123501 -0.424747643 0.3493401 0.662702032 -0.668945893 2.175673614 2.18766665 0.223925237 -0.801465622 1.057356799 -0.779141851 -0.015942089 -0.201052451 -0.494856806 0.253143144 -0.528707486 0.451409711 -0.499279604 -0.489343746 0.540429274 -0.942229938 -0.826175471 1.242859076 -0.003866704 0.352999264 0.371168289 -0.108833944 -0.231955809 -0.160750516 -0.266467886 -0.456106878 -0.632112596 0.355890722 -0.386935251 -0.249045634 0.246399337 0.246693487 -0.036504484 0.27635933 0.357742734 -0.304390631 -0.462921495 -0.179808373 -0.208443688 0.178548928 -0.340109937 -0.084950553 0.059629902 0.00299064 0.148533041 -0.352257375 -0.366793843 0.07931291 0.225574566 -0.135321258 0.183284593 -0.016226462 -0.059803519 -0.246668867 0.076692425 0.422774761 -0.000477399 0.017542869 -0.057056526 -0.106587844 0.318313021 0.166458186 0.038688144 -0.187173404 0.117815086 -0.122884646 -0.129149477 -0.068311188 -0.376001011 -0.001754894 0.205652192 0.241047447 0.285138393 0.033272369 0.086553883 0.046877162 0.057903437 -0.093283398 -0.076398522 -0.094184609 -0.058587909 0.017940909 -0.062745212 0.049785142 0.106969371 0.094356429 0.035544787 -0.047723897 0.027315927 0.075375607 0.078516539 0.068461704 -0.032513631 -0.087872465 0.079019691 -0.021908905 0.046199219 -0.031689295 -0.025981686 0.077277473 -0.008706957 0.253734305 -0.102513436 0.002325876 0.127111201 0.050372254 0.025539481 0.005055506 -0.054342717 -0.131813816 0.021090863 0.029486606 0.079444698 0.071808189 0.152228276 0.077750694 -0.057722623 0.007768409 0.097592045 0.123426981 0.005306692 -0.022863069 0.00876703 0.053326943 -0.087047184 -0.015557546 -0.019670723 0.033991115 0.035508706 -0.007679793 -0.074418445 -0.07284036 -0.003406175 0.041181644 -0.083983215 0.052864451 -0.001958957 -0.056176924 -0.033465289 -0.10963774 -0.135914017 0.089225815 -0.067461744 0.02733697 -0.009865243 -0.111428576 -0.031088224 -0.06388882 -0.044794023 0.015857154 0.048188378 0.040590085 -0.057058726 0.062077865 -0.0241908 0.029997313 -0.144367869 -0.025896842 -0.070196375 -0.017881688 -0.01050513 -0.0648514 0.006249193 -0.03743553 0.020428907 0.041879013 -0.006537738 -0.093483447 -0.033325478 0.048768524 -0.003598051 0.092787612 -0.010216464 -0.001553571 0.00994093 -0.06539527 0.014016342 -0.125325039 -0.084686856 -0.063811916 -0.009560276 0.043349282 -0.046107084 -0.001751471 0.038762708 0.076582542 0.036893692 -0.023866532 -0.057011912 -0.010779843 0.009135125 0.093665853 -0.01172915

sample_03 1.50059838 5.110342039 2.432823294 -2.623639796 1.098417669 0.272233481 0.383463283 1.752780905 -0.179789701 -0.69300449 0.192259336 -0.947343322 -1.389055579 0.267948463 1.159143516 -0.504349545 -0.385841099 -1.804507348 0.482609669 -0.203162786 0.081555532 0.280784417 0.305709427 0.243119559 -0.289015635 -0.543980775 0.643921664 -0.50289669 1.186907149 -0.667745923 0.408747998 0.324140412 -0.432928401 1.026228709 -0.296890022 -0.307241142 0.128186612 0.164263761 -0.276516677 0.009308592 0.017079971 0.308176656 0.082033086 0.273847785 0.210780309 -0.023072949 -0.191646087 0.431694783 -0.107788902 0.332433513 -0.318368552 -0.343276487 -0.274206886 0.279278492 0.142155612 0.329328015 -0.054886233 -0.077321526 -0.163647978 0.032862986 0.293705018 -0.12031266 -0.259732804 -0.495016422 0.239419219 -0.107934662 -0.174094347 -0.129555912 -0.196446639 0.27836224 0.132181038 0.198515307 -0.161890739 -0.059232755 -0.031844648 0.091372924 -0.017751656 -0.307600827 -0.017151107 -0.037311509 0.180523539 -0.014519459 0.135659468 -0.08517055 0.101726447 -0.07229639 -0.336451833 -0.108339477 0.120988473 0.153208848 0.010574599 0.128409351 -0.233398742 -0.103624669 0.127602209 -0.098635258 0.024082593 -0.160548256 -0.04990245 -0.228000081 0.151094916 -0.204742228 -0.08714461 -0.081129367 -0.234081429 0.068089387 -0.023935438 0.114643855 -0.053009346 -0.007012597 -0.15998512 0.050859373 0.133447445 0.172419171 -0.053099082 -0.092414563 0.074688336 -0.041425206 0.090418375 0.111747181 0.059800137 0.000510342 0.024074036 -0.007245911 0.279070996 0.007882328 0.048218652 0.00319669 -0.010053493 0.021398299 -0.078963707 -0.007406267 -0.096713164 -0.113012644 -0.14971007 0.080018218 0.206585187 0.108266235 -0.060852896 -0.059778372 -0.064144471 -0.108397695 -0.005631155 0.1033236 -0.053080118 0.112475748 0.005522373 -0.103139419 -0.056290881 -0.011919503 0.063252524 0.051257432 0.018843996 -0.155893756 0.127815752 0.02510573 -0.12303014 -0.109860184 -0.030826128 -0.051401932 0.04523707 -0.023996403 0.197701753 0.061554877 0.077216517 0.032032734 -0.0996335 0.127802482 -0.083521728 0.062211596 0.108996391 -0.105474491 -0.028280351 0.025497929 -0.050704423 -0.088378989 0.022831822 0.045202935 -0.117675254 -0.071777296 0.00117104 0.048406943 -0.002607055 -0.040737302 0.088435275 0.074860186 -0.136826264 -0.039592832 -0.002087379 0.025551707 -0.051656306 -0.001858779 0.04463234 0.031091971 0.097890805 0.03918381 -0.074228223 0.006741992 0.129894804 0.012347033 -0.097416012 -0.091294064 0.003538601 -0.009276432 -0.035261111 0.092074416 -0.012932955 -0.021569218 0.052309559 0.048216114

sample_04 2.975125211 -0.774822018 -0.266762581 -0.202285914 -2.038572958 0.323340294 -0.99517954 0.759573219 -1.984677935 -0.483751985 0.631644452 0.088287772 -0.191743151 -0.326464658 -0.261267564 1.040358491 -1.207408889 0.086566928 1.18767913 -0.650705409 0.140054029 0.557098402 -0.524288442 0.203103115 -0.217839584 -0.008768962 0.143597672 0.528018423 0.014849491 0.047144885 -0.30739364 -0.046682972 0.447762444 0.39817175 0.855783143 -0.177888512 -0.338560269 0.603969115 0.477233679 -0.415053498 -0.153064453 -0.070035566 0.537672719 -0.584623346 0.080044604 -0.166578063 0.145742277 -0.556180046 0.065511037 -0.086279377 0.246496831 0.096318032 -0.362200434 -0.149891541 -0.236321931 -0.16230033 0.088404375 0.303929901 0.034259346 -0.033508216 0.057770076 0.058716849 -0.001960338 0.16585044 -0.061745903 0.243950338 0.240216209 -0.279582735 0.155581284 -0.009101058 -0.299100676 0.200897342 0.012304013 -0.05943583 -0.083132006 0.017590144 0.029450744 0.130307429 0.151918669 -0.026996802 -0.049710255 -0.181267869 0.115486342 0.279202302 -0.220770965 -0.194512586 -0.056943428 0.048634825 -0.033102916 -0.006235147 -0.016310593 0.046717419 -0.021492763 0.008725134 -0.016754287 -0.009747617 0.164941298 -0.076828316 0.098597251 -0.025980303 -0.073973401 0.12651071 0.008044049 0.054548996 -0.014298897 0.137598799 -0.039064269 0.103758167 0.083067965 0.028379949 0.009646975 0.083897686 0.028671299 -0.069127892 -0.097740257 0.118745852 -0.156221371 -0.319111651 -0.023016628 0.012530428 0.068805189 -0.091561043 0.004718436 0.002935992 -0.105877094 -0.087524946 0.004230328 0.014520847 0.03719323 -0.028254693 0.07262502 0.117158221 0.034224885 -0.002199413 0.24037864 0.06947989 0.049196008 0.067118422 0.112827545 0.027198361 0.105800692 -0.029499344 0.098967903 0.119172573 -0.118434945 0.178172227 0.075673658 0.04712648 0.090139665 0.041749557 -0.013647642 -0.073127485 -0.026771095 -0.038674162 -0.011404118 0.019224754 0.041933544 -0.091346684 -0.067273757 -0.032584338 0.011376028 0.060116104 -0.01125623 -0.08593081 0.050444252 0.060088252 -0.008853832 0.042454723 0.022952217 -0.00073768 0.016637485 0.085769272 0.020269149 -0.01846468 0.085937727 -0.001256774 0.115233761 -0.064202677 0.025272719 0.043216684 0.015147642 -0.024112858 -0.032111497 -0.00825339 -0.017383463 -0.041643973 -0.058211192 -0.032306272 -0.056627251 0.004448555 0.06278551 -0.014188927 0.001336694 0.071748286 -0.04679163 -0.052453393 -0.015235906 0.029770844 0.016228776 -0.059218916 0.065546181 -0.030455661 0.024731834 -0.07809037 -0.04784218 -0.02686063 -0.008139959 0.007686156 -0.007395806 -0.031157285

sample_05 -5.241078001 -0.379225111 -1.314094848 1.221041866 -0.346135874 0.019869931 0.935157123 -0.135203469 -0.409847554 -0.816002649 -0.869694124 0.313010115 0.540305327 -0.380320163 -0.228728366 0.669430374 -0.029962799 -0.07672711 0.553038382 0.16760634 -0.157907647 -0.178442231 -0.001129834 -0.378194327 0.084637987 0.055372896 -0.305327751 0.407082051 0.12097816 0.255351445 0.131257326 0.394919793 -0.197370488 -0.027666472 -0.26792204 0.3182308 -0.127388575 0.196931507 0.193687524 0.083885898 0.019668613 0.347482266 0.03983149 0.168547032 0.078963847 0.085651536 0.080735479 -0.040576285 -0.029993558 0.023464642 -0.109849473 0.0622857 -0.058482048 0.210053143 -0.057616219 0.005004721 -0.135501815 0.159005941 0.265526577 -0.089643827 0.162854095 0.194378807 -0.183248063 -0.07850786 -0.087357904 -0.031363496 0.047120967 -0.056839245 -0.219151843 0.063822045 0.032746185 0.00304697 0.152088944 0.121439827 -0.019951002 -0.120918045 -0.084495476 -0.035574837 -0.034518904 -0.09341191 0.087870527 -0.013105307 0.030656483 0.031413102 0.042678731 -0.08936869 -0.028532431 -0.058777073 -0.033230543 -0.118873547 0.036353722 -0.220975132 0.170059845 -0.014237311 0.08171652 -0.059103761 -0.042525506 0.007203347 0.016140743 -0.134084412 -0.082242824 0.03668486 0.06911144 -0.0806878 -0.13016494 0.118833433 0.007239205 -0.186000777 0.028862736 0.019283425 0.006691391 0.002053954 -0.032887627 0.034612835 0.040528918 -0.000242352 -0.009737407 -0.002392528 -0.020286523 0.046640092 0.034539584 0.027100064 -0.060853675 -0.01716573 0.053614636 -0.092919806 -0.106559229 -0.038702182 -0.053620936 -0.040360033 0.071018241 0.10541602 0.008385474 -0.054532665 0.13269097 0.009743735 0.023714329 -0.095184851 0.027765671 -0.028097103 -0.018231699 0.009731006 -0.026399958 0.052514969 0.011065085 0.073169587 0.090686862 -0.055864224 0.007447298 0.013122986 0.035891107 -0.027159997 -0.06093755 -0.004333527 0.013473114 0.029669088 0.026262689 -0.022194817 0.035819235 -0.084775422 0.02950677 0.066686222 -0.005104362 -0.070698639 0.002647317 -0.006299056 -0.109504242 0.004758841 -0.030884653 -0.071373728 -0.0083466 0.02355085 -0.087063824 -0.02799399 0.032387494 -0.005497134 0.042725567 0.017308093 0.06150773 0.018333573 0.031376403 0.014575334 -0.018636944 0.023092736 0.020417581 -0.014270491 -0.029054112 0.093109265 0.003864085 0.027009353 -0.00122985 -0.024232464 0.005959532 0.025141913 -0.003455118 0.01618168 0.021176637 0.011419655 -0.021268128 0.032398073 -0.014006846 -0.036607699 0.001574946 -0.005798878 0.083086236 0.000541479 0.010217359 -0.003979297 -0.044023423 0.054146461

sample_06 -1.751820091 0.481905136 -1.252748865 -1.552060783 2.435198979 -0.527399675 2.398151222 -0.381640602 -0.729674786 -0.425704166 -1.77082438 -1.193513568 0.595136376 -0.262649136 -0.608247934 0.44866726 -0.144818551 -0.019844611 -0.859058829 -0.425445454 0.648437314 -0.029277188 -0.435867018 -0.136867543 -0.157273425 0.267064294 0.230282832 0.304207419 -0.014851728 0.321469041 0.115386701 0.030441237 -0.598650537 0.53930214 0.266047721 -0.509813934 0.37400144 -0.089716914 -0.197657561 0.609155357 -0.181381799 -0.210497644 0.072857657 0.3286364 -0.268456065 -0.209044013 0.136020625 -0.054411879 -0.04153704 -0.024178614 0.05697736 0.146900461 0.002441806 0.04041017 0.161334505 0.230880489 0.250902164 -0.168380088 -0.063243049 0.351546228 -0.144731634 -0.063230106 -0.363733386 -0.042644092 -0.086896332 0.171800689 -0.094391685 0.07257217 -0.006624234 -0.248349474 0.090820193 0.20442389 -0.03899099 -0.24898067 0.139426062 -0.001438738 0.012052306 -0.035419191 -0.163492811 -0.020955298 -0.098089174 0.003890251 -0.053337541 -0.094349852 -0.024270585 0.08211512 -0.035390422 -0.01858168 0.090619532 0.20933543 -0.074309035 0.078407254 -0.038412196 -0.116993007 -0.036381927 0.102955772 -0.187673653 0.136410323 -0.03282315 0.044280444 0.053064547 0.041233807 -0.04082133 -0.03937215 0.151158407 0.138096586 0.162167909 0.044389239 -0.111695549 0.181674219 0.043903029 0.024671822 0.050532324 0.074210034 -0.063258308 0.003661845 -0.038216767 -0.117289127 0.014874051 0.002305405 -0.046837085 0.045469229 0.014078868 -0.002939244 -0.099396898 0.106140095 0.081002206 -0.037229269 0.097098773 -0.045970628 0.06756012 0.003241425 0.085401631 -0.011526062 -0.072230728 0.124199161 0.068801189 -0.141005026 0.068086775 0.081301292 0.088334551 0.017996112 0.059494714 0.060317505 0.069723112 -0.065198816 -0.071641145 -0.076515875 0.110158527 0.110876741 0.032984167 0.018325295 0.045198861 -0.046960144 0.016138519 -0.034239435 0.04342406 -0.072282266 -0.069835279 0.057690473 0.00394245 -0.090271118 -0.045517775 -0.043225268 0.057411013 -0.010984671 0.020216404 0.082804632 0.074200689 -0.001261045 0.009249449 0.099358709 -0.077343543 0.071549717 0.063801037 0.007095707 0.025943308 -0.02706793 -0.000412131 -0.012828158 -0.04951933 0.052437734 -0.08078008 -0.010198457 0.031645381 -0.008982459 0.046435926 -0.082995129 0.037198526 0.006743272 0.042255017 0.027059117 -0.030670654 0.048517527 -0.01645392 0.004458982 0.035089315 0.075030917 -0.055089578 -0.008481115 0.069372666 0.009181334 0.042993064 -0.061677354 -0.072787895 -0.020010689 0.003935277 -0.008085152 -0.086379341 -0.021834684

sample_07 -6.554748459 -0.245758947 1.675041538 0.206865172 2.271853854 -0.402796237 -1.366583054 0.255986967 0.383106404 0.960851376 -1.087694715 -0.035685793 1.190410653 0.071156192 -0.006764368 -0.084450721 0.439222173 -0.258717182 -0.53255065 -0.325801551 0.548963115 0.202475476 0.74446667 -0.598912629 0.010414863 -0.839001433 0.296962447 -0.292962023 0.625060244 -0.419675982 0.477998741 -0.265919598 -0.148812422 -0.146844457 0.228833219 -0.059039517 -0.23588871 -0.159822912 0.0935612 0.032688978 -0.196695421 -0.177660442 0.12399358 0.153337455 0.290771867 -0.143952087 0.064892106 -0.220575827 0.157560218 -0.068710274 -0.073297249 0.076675936 -0.058564404 -0.00431227 0.026587852 -0.131466531 0.303660998 0.047362416 -0.036816806 -0.010913279 -0.091766483 0.018297012 0.235386092 -0.14966472 0.001917536 -0.095121519 -0.055943011 -0.141515182 0.133305912 0.006093142 -0.069455245 -0.069609567 -0.28035255 -0.064423989 -0.195415192 -0.120574394 0.00048174 0.025225645 -0.03138293 0.171012831 0.029618439 -0.152335885 0.055893931 -0.053752519 -0.046037537 -0.009495745 0.060572729 -0.081394876 -0.089389862 0.061284803 0.033677875 -0.004944538 -0.005672843 0.049812891 -0.075413614 0.122055216 0.078085537 0.004341117 0.037390954 0.038961716 -0.030673038 0.073032674 -0.068757769 0.147679134 -0.071929471 0.015939673 0.029795757 0.104814871 0.022817476 -0.069729015 -0.116073965 0.100367546 -0.014577847 0.010525813 0.035643485 -0.048201098 -0.00928382 0.005406065 -0.041895917 0.02143918 0.02380902 -0.045202404 -0.009680483 -0.020448302 -0.074823823 0.040788434 -0.040354319 0.005808998 0.120000193 -0.019598783 -0.080702789 0.002933292 0.017325875 -0.057580806 -0.02122133 0.071115797 -0.00542859 -0.004855679 0.058027752 -0.029273922 0.056339982 -0.002216683 0.053275919 -0.05234213 0.0050194 0.046755146 -0.024885628 0.024781827 -0.005394568 -0.010809435 0.03032876 -0.021420536 -0.055904466 0.003784697 -0.042960578 0.043014995 -0.006980034 -0.033058539 0.017284929 -0.023024377 0.035204028 0.022932631 -0.025771351 -0.018561746 0.013148597 -0.024121502 -0.002451123 -0.024947553 0.013112177 -0.02380452 -0.058614218 0.044535963 0.004107191 -0.04297261 0.058962188 -0.015134901 -0.007569762 -0.030000671 -0.063813091 0.008521414 0.000587136 0.003508669 -0.035811964 -0.015549103 -0.020642148 0.010989126 0.054965296 -0.004563944 0.003801161 0.009838442 0.024822738 -0.050913434 0.006105878 -0.025632802 -0.010252512 0.052230833 0.008624847 -0.021360121 0.037245032 -0.039189149 -0.004121201 -0.018161625 -0.000338689 -0.054559526 0.013275552 -0.028439963 -0.030257241 0.00127545 -0.009955128 -0.009818072

sample_08 0.010822246 -1.94312711 -0.692729328 -2.874233538 0.73323456 -1.200201654 -0.713776584 0.563688362 0.154996023 1.014021403 -0.089371519 -1.296776321 0.140511069 0.360046608 -0.056238795 -0.192238142 0.214557161 -0.129589605 -0.501250979 0.459355357 0.078612545 0.363763408 -0.624077716 -0.303959981 -0.218426636 -0.122834056 0.488791844 0.231148905 0.082137689 0.46614866 -0.096035831 0.310139696 -0.226356636 0.283932524 -0.023915732 -0.158756756 -0.205856658 -0.102142378 0.106434414 0.28964296 0.33988808 -0.402873336 -0.401532091 -0.127406571 -0.514927569 -0.321719374 -0.161212547 0.12076444 -0.110930251 0.543477678 -0.295287582 0.060158232 -0.36480881 0.285743954 0.097139701 -0.142697819 0.190613646 -0.035529803 -0.076860523 0.120113274 -0.242608593 -0.225577066 -0.023975927 0.059484305 -0.089120277 0.061363599 -0.217886606 0.058346145 0.257343163 -0.094960405 0.012523574 -0.049788173 -0.056008314 0.015591025 -0.028997239 0.102018053 0.07367543 -0.102630675 -0.046010068 -0.114762765 0.075224247 0.048539379 0.005839044 -0.031864912 -0.009679758 -0.016471632 0.01893489 0.035617521 -0.001363228 -0.061690875 -0.036947387 -0.063062591 0.089338243 -0.243663471 0.002370222 0.159443497 -0.055767165 0.025835343 0.103622308 -0.176715804 0.040172452 0.072911049 -0.01552882 0.055176887 0.193406357 0.125847222 -0.194674415 -0.015484638 0.201567939 0.035039419 -0.031940153 0.101147453 -0.08379998 -0.154381434 0.127425363 -0.053385262 0.107957191 0.061820773 -0.01790447 0.001550611 -0.033423926 -0.048866426 0.131176849 0.05436968 -0.145822862 -0.079119276 0.036504008 0.051813111 0.021095817 0.053841291 0.13473706 0.071811043 -0.007884675 0.026261567 0.013179314 0.045370781 -0.030559923 -0.02708722 -0.023326206 -0.050208777 -0.005140045 0.075549361 -0.005325583 -0.022647628 0.074037249 0.042375268 0.113765947 -0.010484432 -0.01820821 0.023098987 0.099076438 -0.040784132 -0.047411024 -0.063171896 0.030400914 -0.035125645 -0.033894942 0.065601073 -0.074871772 0.039922198 0.043616271 -0.018367932 -0.067015903 0.001770097 -0.072132364 -0.048291565 0.017346688 -0.002446168 0.033167708 -0.018141349 0.054251067 -0.02151453 -0.066006396 -0.074256488 -0.066925521 0.029179213 0.044357223 -0.015548871 -0.055631333 -0.005718382 0.008958672 0.048573838 0.057246135 0.025691105 -0.036857341 0.002147121 -0.01271739 -0.041652979 0.068407523 -0.065040935 0.074445887 -0.009560331 0.039202291 0.001186786 -0.063756376 0.080525399 -0.000563395 0.056012498 -0.034858147 0.003174093 0.084551805 0.014813208 -0.016499068 -0.049785843 0.100505594 0.026740421 -0.037634558 -0.0133195 -0.004914151 0.010000794

sample_09 -1.642187601 -2.626581327 -1.904493519 -1.434650701 -3.587677892 -0.923151025 -0.110075421 0.35792213 1.873337114 0.360033278 -0.587380049 0.837232859 -0.869190074 -0.027691064 0.321645057 -0.595015705 0.904503089 -0.081487984 0.088533588 0.478877197 -0.126687825 -0.651618592 0.003210568 0.3185239 0.110949411 -0.343566464 0.287092951 -0.115011821 0.692740582 0.307074477 -0.894765148 -0.428317901 0.296464028 -0.203395334 -0.497464223 -0.308874266 -0.014620445 0.150603523 0.303992408 -0.092094588 0.462050526 -0.139479556 0.142546967 -0.113500816 -0.346633523 0.112795284 -0.124024376 0.122337207 -0.100500415 0.098838114 -0.093733822 0.131213262 -0.17800999 0.218118091 0.009075172 0.124095611 0.014935438 0.057365634 -0.213250948 -0.083938782 -0.021762958 -0.206725662 -0.332990343 0.355355423 -0.169188592 -0.317926197 -0.042816334 -0.312806904 0.195679839 0.013189724 -0.328193487 -0.35533799 -0.308746079 -0.098794433 -0.123883792 0.132579608 0.075691483 0.058090393 0.226979789 0.198255772 0.020498817 0.02077285 -0.413009809 0.12770925 -0.02035218 -0.008057787 0.009858057 0.09132075 -0.060589515 0.360025702 -0.315671296 0.133777715 0.018853373 -0.189609645 -0.007057186 -0.163811444 0.181732627 0.189605879 0.048034366 -0.022052488 0.174310893 0.076245917 -0.039348125 0.004146211 0.060164171 0.044142528 0.02199928 -0.233634407 -0.0858594 -0.073442005 -0.065418234 -0.000146913 -0.1141973 -0.204198089 0.07911005 0.025471524 -0.101594603 0.02258458 0.238439173 0.095418279 -0.03917599 -0.005337093 0.016152695 0.002481643 0.041027035 0.097575217 0.138250836 -0.080392871 0.035297721 0.166627841 0.011136536 0.000795918 0.014584794 0.036298314 -0.053669148 -0.008008975 0.06449422 -0.123162492 -0.074440677 -0.024953717 0.124927729 0.052324419 -0.003004285 -0.008772725 0.055758795 0.050516134 0.075691674 0.039390296 0.069926426 0.073139573 -0.102645995 0.01718801 0.069874186 -0.020683412 -0.032964637 0.129844726 -0.080969639 -0.061270178 0.001587099 -0.034873879 0.068079156 0.055779115 -0.122664071 0.021323706 0.072844031 -0.000391895 -0.046590032 -0.017180257 -0.006039022 -0.047056978 0.037795642 0.046004879 0.069273063 0.00088007 -0.084314797 0.044532576 -0.029322061 0.069408871 -0.153391833 0.01887757 -0.087770775 0.034554961 0.049277072 0.07083195 0.111263359 -0.09089127 0.080300933 0.075807803 -0.087134519 -0.030634641 -0.055397121 -0.17390596 -0.060875593 -0.035818832 0.045871531 0.006354711 0.062994631 0.111202066 -0.074195245 0.003244552 0.043582935 0.107831646 -0.012386307 0.051051254 -0.033733087 0.007545694 0.02319308 0.049534176 0.05095731 -0.057956496

sample_10 8.718530399 0.853949572 -4.558544707 -0.120062256 4.306704357 0.394510606 1.909492507 0.535590165 1.138412067 -0.346892528 -0.494538987 -1.078657452 -0.229511592 -0.946276291 -0.329251188 -2.392199556 1.051451205 -1.001180479 -2.296205477 0.736853726 0.962524398 -1.397857304 -1.237064005 -0.382042831 1.025062513 0.904079387 0.185174744 0.658361479 -0.476074135 -0.064714109 0.222543801 0.366472615 0.506669587 -0.06646075 1.015730307 -1.090291736 -0.139790347 -0.248727613 -0.341415311 0.200538375 -0.435246339 -0.356641656 -0.131829169 -0.126821594 -0.381817642 0.182036051 -0.500331285 0.495122053 0.251926273 -0.238762164 0.143420638 0.292691668 -0.39913922 0.10174968 0.009634499 -0.17735426 0.60120586 -0.152958646 -0.099813382 0.523184602 -0.283361393 -0.057386742 0.06289831 0.066426941 0.208805028 0.245524505 0.017489509 -0.142854522 0.244782593 -0.188792241 -0.394360096 0.114927056 0.094625365 0.38790511 -0.249512454 -0.014694869 0.382522803 0.110952997 -0.089071868 0.068963494 -0.021882754 -0.001744089 -0.118359639 -0.203320332 -0.052180186 0.17608001 -0.371683822 -0.183455987 0.01462554 -0.285328247 -0.147596605 -0.260684217 -0.077881709 0.022997084 0.010774759 0.01594255 -0.105687035 -0.008869114 -0.121132553 0.058942841 0.133938642 0.199307396 0.021225558 -0.253317853 0.130792407 0.235080781 -0.054943438 -0.308209758 -0.020979339 0.119039802 -0.239860052 0.120507639 0.044520289 0.255149621 0.009378319 -0.037565917 -0.090014404 -0.008522471 -0.209278583 0.028449518 0.062510544 -0.091805878 0.117322602 0.201018338 0.116631912 0.043747771 -0.016969743 0.230355607 -0.223033778 0.037100748 -0.055866991 0.101487317 0.198196287 -0.029862921 0.111838295 0.029229025 0.266633978 0.144671972 -0.067875346 -0.059447412 -0.09401749 -0.022448227 0.022178361 -0.146707864 0.002071897 0.004638519 0.121987282 -0.007511869 -0.130129442 -0.138306191 -0.070451038 0.047554919 0.153100622 0.059926357 -0.066498135 0.132171416 -0.132300998 0.057641684 0.171792654 -0.02065218 0.055905728 0.102239913 0.042292566 0.222234622 -0.053506637 -0.000702391 0.156739502 0.09889258 -0.078415793 0.118300677 -0.067610672 0.07555928 0.025878861 -0.056419327 0.122655896 -0.003405531 -0.085499061 -0.097930923 0.163377198 -0.070584114 -0.086004101 -0.074454439 -0.040487289 -0.034399724 -0.048232699 -0.025803952 -0.025357662 -0.04294212 -0.028640418 0.06808112 -0.072886917 -0.095664291 0.064462992 -0.018676802 -0.056105624 0.084970053 0.057313623 0.002028458 -0.001704664 -0.016838643 -0.020303832 -0.066246673 0.012343976 0.095252115 0.062915078 0.124936774 0.075560773 -0.000939655 0.096710735 -0.033535238

sample_11 11.29868935 3.517679623 -6.670252896 0.102261036 3.376379596 -0.15553749 -2.058297757 -0.966138534 1.955257193 1.001225928 -0.159092649 -2.767970913 1.121689232 0.028993341 1.766460226 -1.183822288 1.781777519 0.721101377 -1.940704968 -0.043027094 1.198771833 -0.8836696 -1.168866279 0.898778063 2.264272338 0.269801425 1.515186798 -0.791055909 0.171405037 0.211711288 0.077585001 -0.217090788 -1.084325249 0.026918549 -0.285408822 -0.090024069 -0.210691216 -0.151933675 -0.955006403 -0.693577027 1.138261273 -0.730546803 0.280003907 -0.592550611 -0.388281686 0.723013761 -0.398278972 0.138202823 0.071546916 0.146281167 -0.375479847 0.544672764 -0.079467911 0.363202852 0.379471526 -0.342785352 -0.079451724 -0.448619655 0.196728818 0.327716235 -0.047301172 0.056529672 -0.007743548 0.445629181 -0.818405327 0.320909339 0.313294255 0.397430329 -0.288649236 0.093132915 0.309645527 0.841222581 -0.169149668 -0.047095693 -0.239397484 -0.248999271 -0.08154536 -0.058990762 -0.210985161 -0.134124317 0.154807417 -0.119969567 -0.113476999 0.104356281 0.202443813 0.068590367 0.206531056 0.184786537 -0.164014291 -0.069410577 0.257521469 0.090039345 -0.060751992 0.183143119 0.12745064 -0.006229234 0.033493582 -0.374764536 -0.465752033 -0.029308618 -0.203409463 0.362816699 0.326947287 -0.395875737 -0.336968323 0.400158875 0.286905856 -0.303156948 0.406208969 -0.028496536 -0.203385932 -0.019531555 0.281007204 -0.217710143 -0.199724821 -0.041888471 0.253704344 0.025191129 0.121456936 -0.151562751 -0.220883058 -0.163158052 0.165914226 0.134781409 -0.159162744 0.037000007 -0.074952423 0.517071956 0.130282182 -0.011480592 0.317448315 0.040946324 0.036397181 -0.135888774 0.144615193 -0.136135126 -0.070403902 -0.038058623 0.240630904 0.041951703 -0.068162247 0.01681946 -0.006842239 -0.147463924 0.037455707 0.006110354 0.035028387 -0.152009093 0.151186471 -0.214069459 0.149508504 -0.198109444 0.012407634 0.037824725 -0.05495922 -0.082493687 -0.015820407 0.062245789 -0.02362477 -0.238477818 0.134377461 -0.010737897 0.153873061 -0.057063004 -0.256982633 0.21398754 -0.084561593 0.030278984 -0.090229653 -0.080772137 0.204154706 0.201346139 -0.171857018 -0.097663347 -0.036535362 0.269619187 -0.058755938 -0.044599605 -0.008006138 -0.057300612 0.034833415 0.133176121 0.112716057 0.030180994 -0.011899145 -0.047836641 0.018957846 0.108349861 0.075866005 0.067692265 0.079468131 -0.053182544 0.047492806 0.223007485 -0.054153549 0.077744701 0.039894772 0.109708165 0.026403756 0.017907666 0.036011317 -0.005853587 -0.072029626 0.011189884 0.043211962 -0.053362709 0.234127197 0.134831842 0.163303959 0.076725011

sample_12 -3.748363434 -1.607388704 1.088091366 -1.480688563 -0.350198288 -0.310515372 -0.82905765 0.569964599 0.274393525 0.396187054 0.425911911 -0.465420009 0.527913185 -0.009871868 0.337614792 0.152752641 0.197700365 0.377249899 -0.012792295 0.122118373 0.475156438 -0.029371691 -0.273429241 -0.230952273 0.104404717 -0.084393979 -0.552907659 0.046739671 0.315935388 -0.114727452 0.333692892 -0.414403904 -0.291445916 0.02236375 0.194289844 -0.255292233 -0.016945988 -0.21366829 -0.206348538 0.023004687 -0.138798037 -0.057805794 -0.116393551 -0.073029348 -0.090004661 -0.284587754 -0.070492999 0.119304246 -0.008819218 -0.063254278 0.025643851 -0.140883429 -0.026615093 -0.02833171 -0.009989612 -0.071789437 -0.118801102 0.111338836 0.094887535 -0.074581176 0.104817157 -0.056048091 0.105765452 0.041085561 -0.075807742 -0.161144402 -0.008311567 -0.083964469 0.24827687 -0.026967845 -0.02913985 -0.074184375 -0.087959378 0.099208574 -0.071741376 -0.023088332 0.034892293 0.085819056 -0.142786524 -0.020406706 -0.092791956 -0.062341415 -0.063170679 -0.072385683 -0.050450224 -0.067096319 -0.05425681 0.038132907 -0.06149159 -0.063953619 -0.066934962 0.138389682 -0.036282132 -0.06616697 0.05936287 -0.055245924 0.024324634 0.039293197 0.028265064 0.065318512 0.037933465 -0.032287356 0.009275598 0.067405469 -0.002638159 0.017141766 0.148190958 0.037061986 0.047853832 0.146373374 0.015595649 -0.211332465 -0.011107107 -0.056125495 0.07035885 0.000750309 -0.000176371 -0.016278288 0.084730277 0.026174637 0.099032433 0.034071362 0.025647139 0.108762768 0.055050855 -0.047511425 0.067003219 0.054327977 0.037125877 -0.045163379 0.016547144 -0.009629624 -0.102672679 0.04591048 -0.009094205 0.094835848 -0.03422319 0.020703714 0.009345306 -0.069065095 0.057013892 0.010278375 9.87E-06 -0.124757315 0.032217944 -0.036874921 -0.030280657 0.038890892 0.076643805 0.024529582 -0.031888139 -0.040521216 -0.007896155 0.05452392 0.051352307 -0.03277666 0.038794399 0.009662241 -0.07517769 0.100981147 0.053833079 0.052617656 -0.065741565 0.021318575 -0.035279945 2.96E-05 -0.007890915 0.021351154 -0.002637059 -0.053988688 -0.006286244 -0.014569446 -0.009361508 -0.058494615 0.021087618 0.002174653 -0.00881242 0.009282382 0.024620767 0.000191412 -0.003598698 -0.056144732 -0.015099738 0.014442319 -0.080570226 0.00394345 0.026478117 -0.017920777 0.032330024 -0.049208182 -0.068070016 -0.024742996 -0.004531804 0.017836547 0.009524807 0.049710515 0.014103891 -0.016055447 0.008599938 0.006099756 0.011726711 0.023984982 -0.043939892 0.010874662 0.034092337 -0.045010501 0.042691554 0.009799438 -0.020492759 0.01881307

sample_13 -4.065152418 -3.258793229 0.234700596 0.46037208 -0.006573664 1.358084627 -0.327133525 1.823530744 0.017260954 -0.909298041 -0.093357279 -0.686896113 -0.631730262 0.473698644 1.005906908 0.697641335 0.977508828 -0.180422775 -1.063468548 0.139717792 0.184475122 0.733679872 -0.059251774 -0.488965327 -0.250053679 -0.406301929 0.289704509 -0.040073823 0.149180988 0.278303298 0.373600336 -0.229828631 -0.619756383 0.366787113 0.153298375 -0.274769362 -0.17410415 0.145404315 0.147600021 0.077490528 0.232256941 0.258074504 0.056801174 -0.023465613 -0.190348303 -0.087883185 -0.21807352 0.18109352 -0.107045876 0.272745895 -0.000431588 0.070740452 -0.148466894 0.003148042 0.028745271 -0.26780102 0.01396033 0.063294863 0.13672337 -0.101473045 -0.180035902 -0.110160106 0.099072349 -0.168188749 -0.301052614 -0.130690077 -0.046390351 -0.029506263 0.102331834 0.21103012 0.051010373 -0.165260263 0.084429595 0.215989112 -0.095563908 0.131491237 0.064437924 0.010613 -0.245088796 0.086176705 -0.04726448 -0.115253125 -0.025540381 -0.137716463 -0.086478364 0.042739858 -0.030229646 0.08472948 0.013418633 -0.166986154 -0.027687273 -0.159536114 -0.071654472 -0.069625392 -0.155599189 0.069028572 -0.034683728 0.082357055 -0.04119397 0.099954587 0.224712835 0.08426198 -0.040542512 0.008279342 0.011887559 -0.014474336 0.077160889 0.022760073 0.127903107 0.091151874 0.187797367 -0.146847018 0.062044566 0.225889148 0.078229511 0.086694264 0.072036241 -0.090936454 -0.146802269 -0.070198116 -0.065508521 -0.033635487 0.12366629 0.009240708 0.067003488 -0.010482602 -0.007221592 -0.121673492 0.022932033 -0.041507905 0.045693943 -0.064054409 -0.006530598 -0.078097502 0.138941285 0.032224995 -0.033328624 -0.042409992 0.03395448 -0.113131359 0.01409337 0.03791382 -0.030498375 -0.115242889 0.029315778 0.065399532 -0.07124354 -0.056145411 0.097062625 0.082784342 -0.000746778 -0.055484259 0.160163921 -0.041291871 0.097196826 -0.070177043 -0.074580362 -0.072270442 0.064451635 0.057097651 -0.012496289 -0.009843117 -0.085256787 -0.026438849 0.029711807 -0.00149013 -0.010255371 0.034410565 0.057972228 0.027835182 0.0530125 -0.030330772 -0.032635608 -0.059675274 -0.030968462 0.038372349 0.035459842 0.102860102 0.022038783 -0.008657403 0.05171801 -0.096478866 0.111242258 -0.042895119 -0.011531752 -0.083516991 0.030453743 -0.0429619 -0.021974843 -0.04733133 0.003332352 0.017352192 0.013539858 -0.024541322 -0.06185317 -0.005154394 0.006061911 -0.056812288 -0.081670594 0.056865793 0.045579093 0.00317771 0.028370453 -0.055892188 0.012646221 -0.01562194 0.01836314 0.003005839 0.004313758 -0.0212031

sample_14 0.061250473 7.402024592 3.468284668 -1.917645056 -1.238985772 1.472622594 1.470242379 -0.36045463 0.578096657 2.478497954 -1.871386583 -1.350882221 -0.428293012 0.029453002 1.191524018 -0.67119896 -0.920303081 0.834830029 -0.672160383 0.090401365 -1.00851864 -0.142544597 -0.169489004 0.109998748 1.175638126 -0.692608165 0.427611707 -0.53367903 0.844620942 -0.234400786 -0.540419569 -0.03398629 -0.653222382 -0.239071664 0.127691865 -0.560560564 -0.292492414 0.352446616 -0.309666208 -0.44182307 -0.096182441 0.385132325 0.080699456 -0.107534094 0.025211764 0.271104212 0.508378014 0.241474441 0.36809921 0.42129103 -0.000115186 -0.049831006 0.330888143 -0.39785861 0.557971222 0.776054682 0.191270226 0.006232424 -0.27715206 -0.005476675 0.164899665 0.506400276 0.655604783 0.232871214 0.372137974 -0.345858489 -0.319908772 -0.870036649 0.16501439 0.06641872 0.15493897 0.410991927 0.104933707 -0.329806289 0.284823606 0.139738185 0.097726718 0.072250365 0.070082815 0.165673147 -0.237540509 0.17811795 0.295688902 0.340249808 0.379938962 0.205365432 -0.061285367 0.487162483 0.087300333 -0.156675643 -0.046827028 -0.185627484 -0.022012378 0.072922069 0.221731733 -0.305694548 -0.127469261 -0.053789561 -0.124236323 -0.081841347 -0.294774781 -0.271859342 0.038448104 0.212461929 -0.004107431 0.057266982 0.033962755 -0.172570935 0.279608961 -0.28990182 0.117087365 0.134090508 -0.251590837 0.324916852 -0.096658203 -0.098045186 -0.176927382 -0.021081523 0.197844975 -0.088674044 -0.182179691 -0.08319018 0.010849017 -0.052095548 -0.292991946 -0.032571415 0.072325785 -0.149263004 -0.366410244 0.030245706 -0.241106585 0.224786088 -0.059447096 -0.040146437 0.034534975 0.078673744 -0.144367859 0.071394032 -0.066551019 0.16742081 -0.22312458 0.14637723 -0.065958426 0.137493794 0.126848983 -0.209855058 0.055375406 -0.18786189 0.085230782 -0.086202166 -0.169902817 0.125814136 0.100506722 -0.073395554 -0.026538819 -0.053193734 0.156337142 -0.016415121 0.212358812 -0.078318411 0.042768876 0.120452066 -0.129442511 -0.156581289 0.00358863 -0.047702924 0.151517672 -0.038731126 0.007217778 0.25405857 0.101909097 -0.007522049 0.155010846 0.00283465 -0.043786506 0.077475863 -0.059022224 0.031388451 0.092553859 0.108183279 -0.078993996 0.09271588 -0.13860659 0.019441679 -0.035893683 -0.087627918 0.092317319 -0.081805935 0.019529249 0.146591816 -0.045141124 0.178563421 0.183679707 0.070977743 -0.093821731 -0.124226569 -0.002620422 0.005487 -0.110142041 -0.058789361 -0.022056838 0.008577462 0.104201177 -0.105242106 -0.004186854 -0.061727746 -0.162398303 0.077770109 -0.034315097 -0.013493059

sample_15 -6.104138449 -0.341480388 0.65020252 -0.016810762 0.031206519 1.051936406 -0.88808497 0.981183242 0.341862496 0.637267211 0.150281745 -0.505878223 0.897420222 0.609181112 -0.441406159 0.850707151 0.385673254 -0.408504551 -0.236859097 -0.237368515 0.951228244 0.206014164 -0.493190484 0.440036594 0.413575833 0.327015774 -0.481417849 -0.234607548 0.422910279 -0.08677906 -0.123920208 0.426269054 -0.175913512 -0.145254387 -0.24263499 -0.62700475 -0.595412441 0.134501839 -0.100908855 -0.152110468 -0.063328656 -0.163102835 0.038364665 0.083103637 0.122073139 0.124019892 -0.090778707 0.126303646 0.36205153 0.047275114 0.034397607 0.001719114 -0.007428582 -0.019365447 -0.042062636 0.020883039 -0.008707604 0.20650918 0.149561379 -0.135756893 0.161319961 -0.171680756 0.026919448 0.128119141 0.018282793 -0.140706217 0.053847876 -0.191233662 0.307707579 -0.212267821 0.048558954 0.000180281 0.071066503 -0.115117924 -0.088696719 -0.353490634 -0.078380007 0.172038282 0.152401612 0.129057211 0.060761845 0.099071576 -0.046003982 -0.037772091 0.017357228 -0.042511724 -0.045913033 0.062792271 -0.193363152 -0.007311417 -0.008829452 -0.112325774 -0.225782431 -0.175000391 0.164259958 -0.0231511 0.060200729 0.230998784 0.083174398 0.033694823 0.076456775 0.065022249 -0.108602641 0.06693635 -0.128365248 0.064845228 -0.020742284 0.056016405 0.00172395 0.208804716 -0.012629954 0.069899155 0.008351826 0.064958934 -0.011624659 -0.114225228 -0.018711997 -0.04227468 0.024297461 -0.002777626 0.194973727 0.070351847 -0.032105655 0.067035621 0.188214592 -0.072809866 -0.037451467 0.034352932 0.034396274 -0.133866543 -0.098465816 0.04544514 -0.032010098 -0.032117548 0.124564721 -0.034966345 -0.016829952 -0.181574997 -0.102432554 -0.021055593 0.089082849 0.042935764 -0.066878952 -0.044292593 -0.105179373 -0.027318904 0.111638477 0.110238915 -0.045412325 -0.100632228 0.064222419 0.037513469 0.084209727 -0.054232374 0.012379851 0.062873158 -0.026938763 -0.049774372 0.005622586 0.00515237 -0.022574933 0.034100294 0.031056878 0.079900895 -0.009118335 -0.109741451 0.116831365 -0.116916946 -0.02313009 0.040366508 0.006103267 -0.031639539 0.021913955 -0.003280218 -0.01541126 -0.08414885 -0.023885203 0.01835952 -0.012191368 -0.112804052 0.022690881 -0.019885187 0.054906071 -0.104236647 -0.015634989 0.004419011 -0.002850337 -0.012786316 -0.007797256 0.151850094 -0.011687524 0.012683183 0.018732003 0.011876145 -0.032378041 -0.043457893 -0.013443589 7.46E-06 -0.091810601 -0.010261488 0.02364848 0.01594997 0.090513891 -0.007709988 0.028711232 0.007406573 -0.022286645 0.031096795 -0.050132977 -0.053395329

sample_16 -5.012957783 0.076545334 -2.600812312 -3.619075551 0.87115627 -1.328385329 0.459286928 0.847676629 0.256918085 1.605195842 -0.914568979 -0.405327431 0.726341085 0.581611166 1.058386842 0.369992319 1.240663456 0.057959912 -0.954629949 0.777440122 -0.305829234 -0.259824194 -0.198860308 0.305914808 -0.513034083 0.559842603 0.278914494 0.396898853 0.08824157 0.087261835 0.192342111 0.603426428 -0.07903168 0.731977434 0.450540262 -0.108998952 0.014467674 0.011066017 0.127503244 -0.102309897 0.584400501 -0.310545586 0.028126822 -0.413831971 -0.209901715 0.004564533 0.142532554 0.114829489 -0.115267665 0.699269322 -0.174819995 -0.083629991 -0.675339855 0.022676066 -0.344016214 -0.540835148 0.36987967 -0.116893368 0.178523809 0.415845358 0.125689933 0.124125759 -0.422123542 0.184427385 0.222826072 0.123870831 -0.363303204 -0.377943929 0.384649109 0.145802437 -0.424132007 -0.354802231 -0.3872366 0.279120464 -0.177826056 0.002128434 0.141149063 -0.105954692 0.00131449 -0.063759679 0.050769987 0.047851642 0.132143072 -0.046815024 0.128440292 0.082337226 0.02680763 -0.021810605 -0.202566685 -0.076939874 0.096221031 0.123169226 0.017727571 -0.110638934 -0.288862434 0.012626499 0.072145561 0.07998321 0.021845537 0.221595629 0.041091665 -0.074044945 -0.016473646 -0.146482662 0.174800718 0.055941674 0.034827386 -0.194366486 0.259326945 0.104452062 -0.300830015 0.029248553 -0.076310987 -0.17040666 0.008425984 0.117789875 -0.19730802 -0.032609628 0.068304989 0.080904574 -0.246187535 -0.006099252 0.108865311 0.010382862 -0.047839641 -0.020958175 -0.01917281 -0.13308916 0.045501637 -0.079200789 -0.036610298 0.089834923 0.021470126 0.033810031 -0.018061952 -0.163661293 -0.207619914 -0.103656863 -0.100272876 -0.134291742 -0.06801068 0.024589445 -0.054398279 0.247487233 0.040744276 0.159482216 0.080879874 0.078781127 -0.106022401 -0.048682449 -0.007596867 0.058427312 -0.003589533 0.132162657 -0.001745244 -0.094521808 0.088341336 -0.042020567 -0.018455096 0.037235574 -0.173609558 0.013249527 -0.100604319 -0.107592964 0.097373674 0.009006021 -0.036502362 0.073281864 0.01699695 0.011221939 -0.018602223 -0.038820433 -0.036837446 0.02371627 0.005103143 0.077368749 -0.082330911 -0.054423452 0.039266326 -0.038632964 0.050375329 -0.02338392 0.048199973 0.04334801 0.008881249 -0.00275565 -0.022586507 -0.083370989 0.060151481 0.028194978 -0.012307363 0.02425982 0.006750038 0.074628072 -0.177763301 -0.071830622 -0.094564751 0.043388255 -0.102737147 0.081254964 -0.013704063 -0.12473024 -0.097252734 -0.011217641 -0.166451317 0.086143142 -0.053141533 0.0859569 0.066804514 -0.002747741

sample_17 -0.882663116 0.634017267 -0.907457445 -4.187571686 1.681784784 -0.39539717 -0.86453052 0.513303723 0.889611542 0.600531025 -0.429677306 -2.409754789 -0.429777233 0.903986823 0.183194046 0.222083445 0.449116059 0.111166779 -1.60841317 -0.134620487 -0.391879714 -0.208216339 -0.238201703 0.679319446 -0.270929909 1.136432439 -0.215864401 -0.05590076 0.010693379 -0.512678337 0.104521085 0.437935332 -0.154713511 1.37787191 0.721285028 -0.177521142 -0.455844253 0.218614132 0.673071314 0.183051448 0.305291263 -0.570837078 0.198181379 -0.096023075 -0.383186421 -0.329189143 -0.091452726 -0.047738791 0.225707726 0.281578869 -0.288550096 0.025470601 -0.24386058 -0.095739287 0.153948083 -0.2248886 0.196102985 -0.271476884 -0.118346967 0.154411459 0.086015721 0.256145505 -0.070807974 0.243036578 0.189438078 0.180093621 -0.492295465 0.275062278 0.00358146 -0.09744753 0.08155382 -0.527311218 -0.116744628 -0.14270239 -0.409902258 -0.135633364 -0.148874271 -0.20438261 -0.066779784 0.037848891 0.155005032 -0.149242963 -0.129081745 0.092688975 0.261313066 0.196368358 -0.152991117 -0.159347752 -0.127600374 -0.012801385 -0.054713394 0.152088044 0.069747151 -0.526125276 -0.126921397 0.207233904 -0.119778314 0.165108387 -0.000999392 -0.040407504 0.077720496 0.157313811 -0.087553872 -0.408812681 0.096836186 0.191441173 0.029569166 0.044727586 0.146472715 0.167901058 0.04570863 0.320484681 0.077263783 -0.016258341 0.089802354 -0.162284027 -0.049178654 -0.145865563 0.009513563 0.16152809 0.01906247 0.041788065 0.012071579 0.054951975 0.002354956 -0.012876012 0.011500755 -0.044265869 0.073626459 -0.067172432 -0.157312059 0.111917145 0.153412906 -0.096850888 0.047402923 0.096912416 0.035736264 -0.116006166 -0.030954844 0.047067779 -0.074968297 -0.09505553 0.117799612 0.117260426 0.202448997 -0.092041548 -0.017056649 0.164049951 0.037516319 0.014876739 -0.01894056 0.047710548 0.036411859 0.053495416 -0.016269919 0.001737483 -0.159686767 -0.139589699 -0.050692644 -0.039899394 0.025537474 -0.13272259 0.148073395 0.081886005 -0.096195499 -0.072454723 -0.029829478 0.063813837 0.021194913 -0.101755517 0.070950065 0.085560115 -0.020204583 0.062508658 -0.085971156 0.043375538 0.075110725 -0.15082687 -0.032604489 0.109695823 -0.011340608 0.05598851 -0.055849272 -0.016562089 0.195802332 -0.091955273 -0.124236627 -0.114455218 0.056098032 -0.026415747 0.035435585 0.155701114 0.020695126 -0.011372453 -0.03086781 0.06326529 -0.069395144 -0.011493507 -0.08137004 -0.024755614 0.101984634 -0.039376697 -0.027291507 0.037709279 0.02254112 -0.105510967 0.03691325 0.05217612 0.019386654 -0.014249442

sample_18 -0.429062922 -2.128167371 0.561462805 -2.919121648 2.786027493 -0.19613399 0.39571509 0.57681438 -0.105187037 0.863030876 -1.062862669 -0.813680034 -0.226715492 0.434406942 0.174429198 0.443167299 0.647205285 0.079785292 -0.701280966 0.47652793 0.864258737 0.014977563 -0.288480736 -0.27670278 -0.019796638 0.111913605 0.264902104 0.285613607 0.004062002 -0.116294951 0.388443348 0.168267868 -0.181216931 0.558432567 0.078873018 -0.265753114 -0.144786949 0.153630394 0.089191955 0.114750323 0.385762904 -0.193797099 -0.267052193 0.086852544 -0.231463415 0.046884045 -0.041639206 0.177667252 -0.067183813 0.274210704 -0.020210388 0.025722912 -0.023142561 0.022389206 -0.107635783 -0.004422556 0.144567399 0.041971567 0.018725927 0.355173864 -0.09310859 -0.067726106 -0.111451583 0.166270451 0.033212804 0.224580571 -0.090014034 0.142602948 0.166861902 -0.166043891 -0.10553311 -0.156669897 -0.089203205 -0.128714782 -0.075513553 -0.068277462 0.026785641 -0.169014472 -0.139826812 -0.049840799 -0.094240526 0.108557345 -0.000790685 0.002262097 -0.062804012 0.076101332 0.055526971 -0.025090723 0.061737165 0.039467768 -0.14570031 0.077075755 -0.05039013 -0.07139015 0.015391861 0.116730073 0.018360122 0.043705387 0.087377582 -0.039461304 -0.050654896 0.025504521 0.006380679 -0.107932303 0.230241889 0.14133713 -0.115582143 0.018561762 0.085496397 0.22969852 0.001794767 0.115927774 0.028787259 -0.037156553 0.045017638 0.050522179 0.042847604 0.002961442 -0.000754106 0.038224204 -0.008605766 0.067846515 0.014466279 -0.047723079 -0.048594926 0.011701547 -0.030913073 -0.014276985 0.032870769 -0.041482382 0.045531131 0.014743376 -0.002823848 0.067438212 -0.065084246 0.013328573 -0.002221527 -0.047795559 0.006052456 -0.023617883 0.021797659 0.029100425 -0.042259336 0.024887434 0.036271115 -0.006964644 -0.009243675 0.045844441 -0.00240326 0.015754254 0.019920619 0.019805654 0.049770491 -0.015455879 0.14369579 -0.064029416 -0.015985493 0.007325786 0.013789273 0.043145617 -0.028893682 0.013910171 0.071197678 0.000133189 -0.015656915 0.051861038 0.078745512 0.019963216 -0.016370916 -0.040348212 0.029524838 0.022136014 -0.05624789 0.047308502 -0.002637553 -0.030924982 -0.047078007 0.039104763 -0.03555857 -0.025502868 0.037636655 -0.065719368 -0.012144228 -0.007497377 0.041340434 -0.087237406 -0.038978609 -0.070217736 0.003986169 -0.020106487 0.003440007 0.050239366 0.019973507 0.00795912 -0.048251077 -0.012166203 0.016951966 -0.034749388 -0.024821157 -0.035944769 -0.003837268 -0.009768544 -0.005206808 -0.011496784 -0.106997214 -0.06132642 -0.013283118 0.047474022 -0.057875218 -0.047149829

sample_19 2.126205012 -0.426068766 0.182476436 -0.799730877 0.59403421 -0.46618509 2.055151191 1.203780278 -0.134731136 -0.374758693 -0.974129387 -0.775759688 0.239107913 0.021133266 -0.468501651 0.01010265 -0.329858313 -0.317054897 0.147840315 -0.471287134 1.326492271 -0.397160377 -0.543771008 0.541550064 0.343716996 -0.158319208 -0.180970312 -0.018246067 -0.292061569 0.529589813 0.239182889 0.331418667 0.232047972 0.311249321 0.096553583 -0.322802 0.292232922 0.173172322 -0.332452145 -0.053452966 0.182128841 0.00705425 -0.124697176 0.454467426 0.181926045 0.066252557 -0.166704644 0.205825023 -0.116504296 -0.283459887 0.017503781 -0.094789573 0.088377786 -0.242019321 -0.070523871 0.430932998 0.080315186 0.345382666 0.213006038 -0.061169524 -0.178521583 -0.151880709 -0.153485336 -0.06223788 0.132872004 0.045870572 0.206321087 -0.053065268 -0.21412394 -0.113262005 -0.045217672 0.0804754 -0.211272521 -0.135076858 -0.132834123 -0.036095527 -0.057176438 -0.042716513 -0.083668717 0.021023471 -0.047463858 -0.132794862 0.133667897 -0.025396968 -0.110794407 0.037620374 0.010216324 -0.119635932 0.091228451 0.199860356 -0.06668499 0.153761675 -0.063048853 0.042516756 0.137565212 -0.037933135 -0.053395359 -0.058594975 0.008569115 0.027418075 -0.030784378 0.127599838 -0.074023861 -0.085223083 -0.009187021 0.052826277 -0.155302751 -0.064870348 0.123512233 -0.10827092 0.121405427 -0.118085752 0.105845958 0.041728716 0.053305355 0.010348166 -0.116500912 0.013143975 0.033161047 -0.087119765 -0.11572303 0.036356199 -0.118797607 -0.067718573 0.027831571 -0.067663242 0.201080536 -0.104637189 0.068065985 -0.154602109 0.035109878 -0.052288561 0.027764364 0.061704249 -0.020116034 -0.015247673 0.086316019 -0.042690104 0.023774152 0.062182065 0.036305505 0.025744991 0.032869376 -0.113187834 -0.015114871 -0.028157906 -0.016367316 0.113588772 -0.129004364 0.062313526 -0.062799023 0.006020068 0.034086304 -0.010491015 0.039244143 -0.001326933 0.084225592 0.026599235 0.033932319 -0.003983939 0.100884289 0.016989185 -0.041451651 0.001014383 0.042201747 -0.067270276 0.073998574 0.037053374 0.080282032 -0.055066048 0.02282656 0.042982707 -0.015162123 0.057777873 -0.023368795 0.006752714 -0.032227278 0.003881662 0.01365508 0.003528119 0.057401053 -0.105345255 -0.022851504 -0.01531627 -0.062563176 0.023382051 -0.017751683 -0.024635316 0.005248455 0.003494979 -0.011279316 -0.036187581 0.046611046 -0.014095272 -0.005969101 -0.026575227 0.029972785 0.020592552 -0.050809613 -0.055607318 -0.070398437 0.022750855 0.02689911 -0.068162002 -0.016160426 -0.046389965 -0.004164875 -0.000120043 0.042258315 0.003047677

sample_20 1.269969007 -1.827664753 2.86574584 -1.324002782 1.096584633 -0.34562326 -0.286837069 0.553262902 -0.258048504 -0.172953542 0.561175913 -0.844369297 -0.241539029 0.470727868 0.900562906 0.517880568 -0.058940064 0.18175939 -0.340412816 0.077382491 0.235347143 0.658589882 0.094685891 -0.285861044 -0.128866938 0.28680319 0.110200777 0.060495836 0.394539709 -0.121118188 -0.090380896 -0.191347833 -0.262266902 0.211324658 0.158062224 0.067850418 -0.347883659 -0.04653846 0.018554284 0.029028226 -0.124826437 -0.163677127 -0.357384961 -0.191310764 -0.112451789 -0.325177504 -0.067406012 0.037842414 0.009639319 0.184823604 -0.047829517 0.073021818 -0.159282704 -0.009756334 -0.074770654 -0.116858411 0.022895881 0.038413932 -0.032744561 0.089491165 -0.081047181 -0.034287278 -0.089111954 -0.234960327 -0.094265724 0.112174704 -0.043056519 -0.010929941 0.026430142 -0.030166707 -0.028934442 0.012291631 -0.1001242 0.074078135 0.048359677 0.036214117 -0.039304127 -0.037175294 -0.066357845 0.015999653 -0.095857067 -0.033063142 0.025595999 -0.105774672 0.035841512 -0.064710148 -0.064897952 0.064138934 0.038426048 -0.010573808 -0.165965225 0.05137039 -0.008460944 -0.195651764 0.04689626 0.026815281 -0.00619726 0.026899075 0.091943124 -0.070424656 0.041813155 -0.013575424 -0.037022236 -0.133390315 0.080827855 0.012093768 0.088287747 0.092149594 0.059975669 0.115624557 0.038808994 0.098658472 -0.017230544 0.070811305 -0.027397982 0.001815079 0.024379314 -0.01697221 -0.037805828 -0.039574959 0.132866153 0.007761694 -0.044080241 0.015918069 0.002064511 -0.113910248 0.002833601 0.075294646 0.025959753 -0.061018 -0.027659815 0.081525716 0.025841176 -0.063105966 -0.01531075 0.048715113 0.063483412 0.08584621 -0.035674563 -0.067813355 0.055415776 0.012517095 -0.014793488 -0.003995633 0.066507998 -0.057206986 0.00741037 -0.023033467 -0.016375732 -0.032087329 0.082060804 -0.023736677 0.00271549 -0.052956712 0.003542593 -0.08309212 -0.06594533 -0.08642263 0.035885619 0.002988816 -0.035626841 -0.006438098 -0.01684428 -0.029302796 -0.058827796 0.054079666 -0.013466133 0.022859523 -0.023134732 0.020391103 0.032213389 0.041449577 -0.006420021 -0.046473927 0.027575179 -0.015117001 -0.005778787 0.039256266 0.038261776 -0.002281354 -0.02691694 -0.024449806 -0.019725387 -0.007914101 0.023192589 -0.023880644 0.004429039 0.037558651 0.00311039 -0.035917837 -0.005697725 -0.008947924 -0.020944482 -0.014926515 0.00573196 -0.004751072 0.024241936 0.012795563 -0.052181137 0.029775822 -0.003775032 -0.043758682 0.039016369 -0.035419143 0.005060143 -0.069498798 0.049025243 -0.001016648 -0.034237431 -0.034408732

sample_21 3.597901589 -3.336741915 1.600158869 -1.225122369 1.657507786 0.50255929 -0.744085464 1.419778474 -0.166559117 0.732583403 -1.110326142 -0.465858196 0.067435899 0.805841526 0.921267536 0.627160419 -0.416229796 -0.442894474 -0.469900376 0.74580639 0.653137952 -0.130013659 -0.211706989 0.111578888 -0.481923898 0.678539477 0.24553888 0.514801756 0.078303001 -0.235110145 -0.179395616 -0.245461396 -0.007279964 0.526790065 0.025282866 0.60558027 -0.008727868 -0.11738567 0.40514713 0.218891511 0.061970689 -0.194077843 -0.155399246 -0.017593128 -0.208656806 0.28574126 -0.116969803 0.425706028 -0.008317129 0.248993694 -0.064697429 -0.054702362 0.054045213 0.04946614 0.11649313 -0.060780788 0.186426041 0.012430882 -0.431238035 -0.06412637 -0.124624555 -0.105175764 0.147179857 0.105620235 -0.080460403 0.016539229 0.059416313 0.077063955 -0.003833471 -0.1945722 0.170130396 -0.227024346 0.184967113 -0.151341043 -0.132355115 -0.052380904 -0.244070517 -0.1581766 -0.25401179 -0.127703168 0.109729159 -0.117555403 -0.260653758 -0.098208348 0.059199466 0.146319534 -0.024811245 0.18356664 -0.003890627 -0.046103842 -0.073882659 -0.118012472 0.058752349 -0.257629697 0.124191129 0.094363983 -0.087537419 -0.015004441 0.10006914 -0.159293755 -0.031686225 0.107752779 -0.108548112 -0.126113331 0.003137775 0.265171299 0.08962458 -0.048606497 0.197637121 0.030945157 0.064889002 0.037031704 0.051827315 -0.078595052 -0.023807405 0.093775614 0.003357033 -0.064227031 0.01548372 0.098700127 0.004277653 0.087284511 0.020672397 -0.008334328 -0.051035677 -0.000537366 -0.044177541 0.06228436 -0.012009443 -0.024298922 0.020294087 0.051363007 -0.030790382 -0.031009431 -0.014570402 0.052904667 0.083539935 0.03822171 0.112911324 -0.115435068 -0.010905082 0.003121068 -0.181086044 -0.078132366 -0.075559619 -0.02977998 0.058618541 -0.039923547 0.015879642 0.043868189 -0.018696534 0.016693212 0.096285804 0.057490366 -0.009493267 -0.039044345 -0.092047476 0.044913117 0.025876366 -0.001403753 0.101533136 -0.018546933 -0.002363353 0.03588975 0.004542793 0.045307092 0.000592064 0.050083991 -0.112295878 0.079129902 0.026514639 0.001822204 -0.015635874 -0.002186075 0.073726183 0.004778217 -0.035814024 0.028454862 0.046361594 0.016029982 -0.110748059 0.031748463 -0.045238418 0.054247022 0.076288471 -0.014901186 -0.056966624 0.038094604 0.02159118 0.014780882 0.124285914 0.056531239 -0.051790555 -0.08976921 0.107106611 0.038505817 -0.031559614 0.034990671 0.064221872 0.055491321 0.03529862 -0.08545339 -0.062150751 -0.035442091 0.028676984 -0.019520531 0.033004351 -0.029763607 -0.003590681 -0.017942165

sample_22 2.429218092 -0.077387685 -1.709221142 -2.127346013 2.184184962 0.36004619 0.780895336 1.51310209 -0.516925637 1.256327856 -0.954518985 -0.651752582 -0.434980944 -0.063742534 -0.023404079 0.107248578 0.377196808 -0.492187502 0.342949822 0.169439416 0.728433238 -0.490759789 -0.083232374 0.954334639 -0.428476046 -0.37372814 0.636155377 0.103609065 -0.258610999 0.282104907 0.207121947 0.196748255 0.11430115 0.128840688 -0.499467862 -0.258866795 0.293212701 0.151971099 -0.078072128 0.028431536 0.36771181 -0.226089539 0.2949358 0.004257245 -0.458202934 0.075370388 0.137201271 0.432301865 -0.099761643 0.25311503 -0.04019268 0.271155845 -0.172689729 -0.289079625 0.328009968 0.059951589 0.198127841 0.254535429 -0.003689657 -0.135111248 -0.093140478 -0.122792509 0.0778949 0.043740722 0.058764921 -0.127762964 -0.253803778 -0.104241165 0.029174296 -0.090945535 -0.092539685 -0.086286454 -0.162141092 -0.063241019 -0.032843832 -0.018603714 0.111181718 -0.043121405 0.103853395 -0.029640948 0.130118691 0.057737385 0.116879804 0.002408922 -0.010823252 -0.080872506 0.020565368 -0.075463329 -0.159266043 0.073847558 -0.088506297 0.06417938 0.048529885 0.137116238 0.177547718 -0.092486896 -0.003687702 -0.222855385 0.246463332 -0.018220814 -0.185066866 -0.020280647 -0.182099067 -0.030963046 -0.040039368 0.010177484 -0.089132633 0.03205576 0.118393178 0.009082982 -0.22122017 -0.089173924 0.029232829 -0.116073286 -0.099649328 0.027803725 0.062570612 0.038105301 0.230060751 0.033683491 -0.082920047 0.064135755 -0.037433079 -0.067416766 0.019573722 -0.034732001 0.096667921 -0.025566226 0.035478089 -0.014691368 0.08140454 -0.024906331 -0.036134882 -0.10792289 -0.098022003 0.024183603 0.058002373 0.023700097 -0.008543744 0.025375763 0.046343109 0.045065584 0.009168291 0.010700062 -0.111545026 -0.018369483 0.028762859 0.031118073 -0.066244522 0.064114523 0.054809109 -0.050991435 0.009734166 0.066078952 0.042718831 -0.080857969 0.046654712 -0.043905204 0.038807386 0.015999783 0.008562181 0.027293788 0.096112811 -0.112739452 0.004246668 -0.120375601 0.028324145 0.013272017 0.005784209 -0.069368846 -0.019898408 -0.074190482 0.042504596 0.032551286 0.002067802 -0.026414712 0.040272775 0.019294207 -0.0300579 0.121332773 0.007241641 0.013321775 0.033060896 0.004425931 0.02610543 -0.026354418 -0.004620419 0.002421495 0.062369816 -0.016846204 -0.03263946 0.01488382 -0.029648391 0.02131014 0.00362783 -0.000955377 -0.010962194 0.012102791 0.015890649 -0.0266176 -0.053131608 -0.062941289 -0.007130249 0.029222355 0.021991954 -0.038205898 -0.019382175 0.034054318 0.065455721 -0.034037886

sample_23 -3.164652603 -2.226270645 -1.007542607 -2.302817029 0.634352831 1.639258173 -0.531993578 1.388213993 0.344076249 -0.378912361 -0.339652616 -0.016283744 -1.500577768 0.926729869 0.707296221 0.869401659 1.375355399 0.031940024 -0.167957534 0.906321836 0.032996812 0.219603812 -0.054764691 0.210365087 0.263140369 0.141527229 0.352293864 -0.032599571 0.078721593 -0.199905597 0.020041494 -0.198187764 -0.254635554 0.096730878 0.119588995 -0.219695437 -0.078914879 -0.070955734 0.237177747 -0.206016808 -0.060116659 0.08199598 0.013509887 -0.486301405 -0.064319935 -0.142392371 -0.333106349 0.22269831 -0.12246529 0.003971394 0.189725832 0.013710699 0.056127637 -0.134355941 -0.012472854 -0.241716162 0.267957218 -0.06050836 0.208274128 -0.103471041 -0.027133562 -0.125544653 -0.061758954 0.271778511 -0.119375142 -0.204805233 0.175666336 -0.166342135 0.196599343 -0.07115769 -0.126301421 -0.143771127 -0.068739674 0.041783503 -0.071076684 -0.014156724 -0.03464736 0.029405797 0.023438413 0.056017002 -0.051769714 0.072364336 0.064327426 0.030731738 -0.023038068 0.112878278 0.003625583 0.034005053 0.008596818 -0.090783139 -0.124797256 -0.032917496 -0.092009703 -0.055471046 0.11389758 0.011535927 -0.044700525 -0.081401596 0.122526072 0.017655819 0.288193109 -0.049239614 -0.053044516 -0.053221211 0.045139747 0.04166006 -0.095456893 0.010064626 0.038956076 0.201715045 0.119284056 0.005356818 -0.006691359 -0.044266147 0.071401457 0.09987288 -0.033841795 0.009667828 0.079710771 0.05474952 0.119659472 0.046297458 0.206118404 -0.015776384 0.042465756 0.106066176 -0.045612843 -0.174724599 0.022149955 -0.088398106 -0.001575055 0.070520768 0.056753723 -0.037808358 0.020568124 0.040943454 -0.059776783 -0.084600466 -0.001420571 -0.110796954 -0.010277192 0.113315267 0.005074956 -0.046634609 -0.046015717 0.131029426 -0.011267721 -0.065662246 0.050885964 0.015404869 0.013537974 -0.05764306 0.087992125 -0.013100284 0.039101029 -0.009616933 0.058001367 -0.073052623 0.09723035 -0.088514286 0.17004577 0.020262529 -0.039429123 -0.004643105 0.010520888 0.044484874 -0.063389724 0.066080379 0.009146073 -0.01668497 0.065044007 -0.122338613 0.034773167 0.022729678 -0.140145564 0.080502101 0.003981021 0.042989813 -0.085471966 -0.050019093 0.086587548 -0.04750868 0.010774124 0.004164376 0.023740941 0.039935671 0.011219565 -0.004144894 -0.012740905 -0.017690916 -0.004703551 0.030282889 -0.019754248 -0.001151046 -0.015404436 0.022357064 -0.0315338 -0.078227969 -0.05278454 -0.021777583 -0.020915534 0.064270719 -0.033860194 -0.01142609 -0.019087865 0.022932377 -0.038679969 0.068710434 -0.008128561 0.078382306

sample_24 5.432873932 3.02050547 -7.013341544 -0.407487097 2.406380159 3.798861762 -1.320304996 3.28937667 1.829681589 1.000982629 -0.636336289 -2.17164995 -0.263723424 -0.769919047 0.557640073 -1.141987306 1.091897926 0.99950678 0.98315672 -0.985131316 -0.507027608 -1.225985196 -0.658623215 2.094684518 0.776788129 -0.075217025 1.565002149 -0.227872419 1.127739446 0.964402078 -1.15669031 -0.84759779 -0.365125229 -0.208429439 -1.073349682 1.015203628 -0.375186341 -0.818565301 0.218375273 -0.023655915 0.532669388 -1.108800017 -0.003083764 -0.51828718 1.130153085 1.212778126 -0.218376692 0.544727308 0.427444258 -0.243720932 -0.021523589 0.486152563 -0.152232842 -0.523537692 0.685459038 0.149545603 0.159039795 0.604079977 0.634576527 -0.143925142 -0.354034083 -0.402349362 -0.191557822 -0.201541656 0.056140431 0.014002061 0.009787173 0.434492828 0.207226562 -0.305730287 -0.069121062 0.257519006 -0.374180326 0.376547914 -0.053442798 0.446476647 -0.051498334 -0.053809258 0.078157176 0.257530588 0.200835459 0.50945597 0.358285556 -0.185280185 -0.036119973 -0.024638856 0.445063377 0.042392676 -0.241545285 -0.180029021 0.124318723 0.231434526 0.471882316 -0.256025691 -0.167761732 0.220068463 0.011251109 -0.434313433 0.102437197 -0.066171151 -0.08071497 0.094957296 0.240068979 -0.216717979 0.126477207 -0.031919925 -0.278859363 -0.343294407 -0.005050231 0.37197578 -0.220091549 0.295169779 0.127398748 0.195315137 -0.081241282 -0.101957999 0.469268949 -0.023654588 -0.035022278 0.102829098 -0.233097118 0.255805296 0.21532394 0.088564578 0.032665037 -0.153517825 -0.166520404 -0.082156185 -0.283417692 -0.103402496 0.082554173 0.323234091 0.157059378 0.053253952 -0.08661944 0.230206643 7.67E-05 0.150822819 0.057495932 0.069456166 -0.065368815 0.326935233 -0.078363272 0.02532574 -0.31608004 0.334128637 -0.145003501 -0.126506368 0.350727935 -0.102591328 0.041610951 -0.083916456 -0.171482561 -0.025202573 0.119971485 -0.155967859 -0.236415357 0.220843226 0.065687746 -0.304706458 0.240120094 -0.123656016 0.192410282 0.138200393 -0.06603956 -0.187992939 -0.090531271 -0.088227695 0.001635877 0.020578313 -0.098593878 -0.1483962 0.002565104 -0.166787449 -0.04824472 0.076535748 0.013698558 0.182620421 0.157763043 0.198654728 -0.21696797 0.081183804 -0.225443647 -0.038740921 0.170930383 0.148364632 -0.287315587 0.062814998 0.037718113 0.086036383 0.046719322 0.19713833 -0.094603096 -0.082868557 -0.063068666 0.052058845 -0.127386309 0.079149407 -0.091978373 -0.133034017 0.113547804 -0.240053792 0.059004957 0.121455212 -0.00495853 0.156956292 -0.009001881 -0.154176091 0.02591681 0.048986283

sample_25 -6.528825568 1.638307058 -0.735817602 0.11500312 1.889463361 -1.0468063 0.441716826 -0.685480723 0.300300718 1.126259468 -1.02239756 0.101596892 0.260981481 -0.231361673 0.160221185 0.596581624 0.078830004 0.042812999 -0.906282259 -0.189833739 -0.2029434 0.031960471 -0.552645622 -0.273777359 -0.087574406 0.218378192 0.2941136 0.010089271 0.330598349 -0.068352492 0.445986278 -0.103268045 -0.305935919 0.525945995 0.351958473 -0.219859117 -0.091087769 -0.161156989 0.076317047 -0.100723175 -0.393332091 -0.263286482 -0.188121819 0.267425168 0.38484082 -0.182757071 -0.097442196 0.083283411 0.243795523 0.20800291 0.013536061 -0.170752527 -0.030823809 -0.019521223 0.060583106 -0.023536738 -0.037934579 -0.048103034 -0.076293 0.07724643 0.131289852 0.077928627 -0.354410462 0.005209127 0.081506575 -0.018502478 -0.180989677 -0.124151037 -0.004263662 -0.005651296 -0.038285835 -0.10889715 -0.157377802 -0.056710268 -0.041032781 -0.060208523 0.006173557 -0.006312913 -0.053843183 0.08923684 0.060583417 0.034876574 0.057031989 -0.106413537 0.004404192 0.019278439 0.121135967 0.147502776 0.003314597 -0.081458981 -0.066224966 -0.02004612 -0.117209886 0.016605588 -0.094807878 0.08350351 0.114397283 0.175932391 0.054589637 0.118375881 0.012294008 0.109159687 -0.091065878 -0.029351205 0.070258053 0.057097779 0.033167118 0.123925592 -0.046832338 0.129160183 -0.001917157 -0.004619654 -0.024275035 0.010953023 0.055154719 0.066012615 -0.031366107 -0.017253842 0.01016084 0.100001582 -0.125354019 -0.127774048 -0.07128643 0.149510042 -0.028251932 0.101184074 0.02287875 -0.099906606 -0.020208395 -0.049879619 0.071879683 0.048176829 0.010354969 -0.009115226 0.045437696 -0.074988598 -0.027309389 -0.035213663 0.089941236 0.014445579 0.004847839 -0.099398796 0.013353941 -0.016915707 0.012030363 0.05373324 0.080181656 0.052729625 -0.005867721 -0.027632523 0.00999769 0.01256846 -0.034509135 0.044392233 0.087659894 0.019332578 0.042627207 -0.001897965 -0.044827836 -0.045936907 0.016531427 -0.091396938 -0.03125814 -0.028566362 0.025571552 -0.044297475 0.009843871 -0.019109129 -0.019284352 0.001536956 0.031483599 -0.033635378 0.049224519 -0.007357974 0.075006445 -0.031212108 0.026388674 -0.007338938 0.005116778 -0.044076279 0.009405177 0.091325568 -0.040137297 0.022723484 -0.023584833 -0.0257674 0.051111579 -0.00351867 -0.022203069 0.052868308 -0.028656654 -0.018038294 -0.006206635 0.061011412 -0.011186092 -0.009514719 -0.034920895 -0.035380611 -0.031902059 0.035202447 -0.018067542 0.025191569 0.007308962 0.001574031 -0.047640633 0.017374227 -0.025413233 -0.028506163 0.006818184 0.01647527

sample_26 -1.989794257 -2.82589488 -0.338981557 -1.77460414 -1.284597196 -0.686291709 -0.406680147 -0.094006351 -0.183018933 1.437022572 -0.663962637 -0.469949126 -0.406950227 0.093003848 0.846788835 -0.004290384 0.227716278 -0.005538473 0.025694293 0.457160658 0.193003042 -0.024237622 -0.289005043 -0.079147323 0.035417489 -0.164304075 -0.068151335 0.178302797 0.31418007 -0.076552249 0.092295834 0.142427193 -0.017168741 -0.086080404 0.037341628 -0.181295515 -0.140661188 0.038839133 -0.196873447 0.326900751 0.461148362 -0.221737327 0.058803284 0.030492932 -0.276108318 -5.12E-05 -0.322694415 0.193879998 -0.089655205 0.005403479 -0.42552636 0.087391031 -0.051983628 0.190712032 -0.031161403 -0.10777853 -0.097306274 0.056402203 0.135074288 0.029022541 -0.215563811 -0.084526463 0.048034605 0.142826303 -0.208035187 0.001819643 -0.260738253 0.123622291 0.353617282 0.09390892 -0.05829076 -0.065960693 -0.136580345 0.04406464 -0.139873097 0.090855714 -0.061670566 -0.10175391 0.004141563 -0.059714507 0.009069312 0.048467234 -0.03347591 -0.207308058 -0.036053079 -0.005621573 0.150643066 0.069237976 0.132502787 0.006391158 -0.066483963 -0.065212598 0.02147844 -0.052808372 0.008938105 -0.005877734 0.033621694 0.147429392 0.054913655 0.001446289 5.02E-05 0.049905853 0.06318549 0.044652927 0.074510817 0.044144696 -0.048327667 -0.043535416 0.08823112 0.082701276 -0.083573557 0.002480745 -0.031721305 0.026165911 0.06908035 0.038724764 -0.035538848 0.06512114 0.010492367 0.066410279 -0.133846726 0.046486604 0.05406464 -0.035485721 0.028533243 -0.048593116 -0.023723347 0.003657727 0.018122041 0.023600519 0.042953669 -0.078777283 -0.080977364 0.045456086 0.041797297 0.058064794 0.036108509 -0.053188335 -0.055344419 -0.013004578 -0.012395251 0.067739704 0.060291641 -0.004692955 -0.04494488 0.027600773 -0.018798221 0.056766136 -0.010812813 0.075476341 0.024010241 0.021712185 -0.010659616 -0.02941789 0.073904958 0.022700534 0.072753701 0.029969116 0.059556329 -0.026256002 0.027635148 0.004990344 -0.065190755 -0.054148971 0.001332527 -0.074832847 0.054763758 0.05115147 0.047906375 0.020989002 0.012163302 -0.030189565 -0.036671586 0.050938337 -0.077939292 -0.01370305 0.014798702 -0.032695755 -0.06137931 -0.039312085 0.067242858 -0.008917783 0.021350631 -0.008026209 -0.029380131 0.009300062 0.02456678 0.044087447 0.047972982 0.022792215 -0.006105503 -0.022254557 0.028017426 -0.02187637 -0.053251821 0.02320306 -0.015569341 -0.010610817 0.001451702 0.043150442 -0.015342712 0.044943424 -0.033400225 -0.012044211 0.041182317 -0.0166567 -0.06941688 0.029553569 -0.009304579 -0.028984774

sample_27 -5.584270701 -0.015269232 -1.366619898 -2.398657088 0.976856987 -0.220134525 -0.087968331 1.061155702 -0.562063076 0.973572711 -0.996439681 0.279934562 0.36064092 0.423506535 0.615095328 0.746542895 0.480842317 -0.401720036 -0.384793368 0.223325014 0.271438903 -0.117433777 0.145120588 0.478099703 -0.055906511 -0.376177272 0.507128817 0.208738225 0.004226579 0.051332262 -0.346199311 0.542931446 -0.060909733 0.002147146 -7.97E-05 -0.403327608 -0.046480264 0.168008238 0.13273526 -0.159986403 0.25171777 0.245979563 0.155124908 -0.453382366 0.180783934 0.071551616 0.14427218 0.023482502 0.0593549 -0.10589052 -0.043299717 -0.089724924 -0.095106243 -0.018734679 -0.020953472 -0.216633389 0.192962295 0.053290934 0.103434632 -0.055272781 -0.011023679 0.115925825 -0.031183916 0.164929735 0.080122012 -0.044754588 0.013026544 -0.212716527 0.216394145 0.010152637 -0.052596212 -0.319541956 -0.165295205 0.080846651 -0.078665818 -0.043091998 0.035611027 0.167542592 0.212346126 -0.016139759 0.006508213 0.021670877 0.041144491 -0.087587797 0.095894679 -0.101383821 0.147876739 -0.007008058 -0.026717286 0.016426962 0.057256848 0.006589042 0.085956198 0.015579275 0.1066025 -0.153251849 -0.003414985 0.002208062 0.057241842 0.164822353 0.004402262 0.069208843 -0.094695878 0.041013389 -0.035069476 0.068711864 0.024188704 0.038653893 0.036302869 0.099731732 -0.047884721 0.130615246 0.009042161 -0.078521842 0.117120694 0.108736085 -0.035989754 0.092105139 -0.118784297 -0.022302806 -0.066583251 0.018035445 -0.005562912 -0.099104793 -0.002959155 0.16863236 -0.06313556 0.093722986 0.01073843 -0.060465291 0.007521669 0.18425206 0.014127789 -0.048825533 -0.05402713 0.031292995 -0.061077228 -0.025743515 -0.272202218 -0.003938475 0.08063448 -0.006411596 0.032196962 0.150468229 0.052116033 0.035285402 0.026368616 0.006515492 -0.108927981 -0.088664429 -0.016299842 0.074882437 -0.062245779 -0.034017127 -0.040391295 -0.002451891 0.050239831 0.099690637 -0.008409722 -0.022552874 -0.051589215 0.038648488 -0.06487555 -0.080308765 0.046103482 0.041211773 -0.048748084 -0.00955653 -0.034931112 0.021565411 0.02975051 0.003872738 0.003111528 0.040482644 -0.06512734 0.038996435 0.058315885 0.063229443 -0.065601304 -0.033019421 -0.008464494 -0.052157006 0.031621347 -0.017430366 0.053792475 0.074506059 0.074351016 0.034815836 0.014020507 0.02920365 -0.051823995 -0.020969787 0.009735647 0.021164455 -0.070321962 0.007742617 -0.015946845 0.021845794 -0.041023178 -0.019932323 0.079936793 -0.011584566 0.051988888 -0.008091109 -0.03196023 0.020035312 0.004244656 0.002779011 -0.068184569 0.085460847

sample_28 -6.561506502 -3.052507801 0.29897363 4.03896772 0.865503791 3.131983455 -1.494343762 0.786008262 -1.487289702 -1.358977604 -0.83976477 -1.219533172 -1.539612554 0.856650613 1.27189833 1.453335542 0.574011548 0.452660864 -0.641300563 -0.011234865 0.953857703 0.029893298 -0.96857494 0.506171961 0.201196109 -0.579939785 0.649137116 0.542476455 0.325056321 0.225938169 -0.497114088 0.549255798 -0.17476246 -0.251380831 -0.390549548 -0.452006477 0.628125588 -0.023553277 0.285144756 -0.460827879 0.214864735 0.704455384 0.597086628 -0.097071855 -0.120785722 -0.369886186 0.205882616 -0.220196972 0.112820617 0.383808985 0.072843047 -0.25016012 -0.201394926 0.160576772 0.09124747 -0.525575477 0.270502081 -0.024321473 0.722744879 -0.237245963 -0.337112719 0.306749379 -0.113954406 0.238690623 -0.230840727 -0.368373657 -0.025311182 -0.365731511 0.092610071 0.013049205 -0.375875769 -0.190187532 0.586683233 0.491497995 0.273430778 0.069237394 0.026119637 0.01166707 0.11488705 -0.237166059 0.13912049 0.109234994 -0.317470336 -0.336833347 -0.078001699 0.222320552 -0.204760465 0.079534618 -0.084063334 -0.163662845 0.109182864 -0.346227918 0.306636748 -0.138835541 0.020958899 -0.08110524 -0.036367659 0.077555411 -0.187064629 0.152935593 -0.235922466 0.150143698 0.154264686 0.077733723 0.068567326 -0.052417197 -0.06908217 0.05678273 0.013122924 0.075514435 0.228228823 0.030114684 0.230719501 0.271100675 0.171403841 0.137699625 0.18302503 -0.16285545 -0.079704936 0.224164553 0.071661639 0.071674654 0.048937568 0.115880657 0.202196902 0.149663059 0.126028874 -0.005898859 0.150960002 -0.197144285 0.003424261 -0.026902824 -0.108743066 -0.055551263 0.195102667 -0.142168102 -0.141659969 -0.235102514 -0.181166837 -0.069906908 -0.339815744 -0.022745312 0.044698714 0.094498175 0.046661505 -0.009195686 -0.099113399 -0.331729822 -0.067290288 0.03073356 0.002029034 -0.034552103 -0.08306147 0.049457594 -0.136209872 -0.098141784 -0.189185795 -0.092001334 0.014483378 -0.050321804 -0.028164718 0.042469761 0.073152535 0.076134066 0.053511209 0.0806735 0.208202544 -0.037491381 -0.02358568 0.124856294 0.128812147 -0.186504686 0.1005734 -0.07484748 0.003271059 -0.044057875 0.029941496 0.232849734 -0.029537996 -0.114145352 0.310140227 -0.016020323 0.033734215 -0.083721425 -0.079485924 -0.063183772 -0.029691964 0.014178844 -0.180755805 -0.05234192 -0.055975247 0.017601055 0.018624952 -0.031532588 -0.018848761 -0.154330587 -0.065889263 -0.105293755 -0.002141 0.029240328 0.095005137 0.05902092 0.029929711 0.042707745 -0.109751026 0.068272429 -0.127042648 -0.060755959 0.09343688 0.082503393

sample_29 5.074962021 -1.050198503 0.380580506 -0.064919168 -0.77993176 -2.207650227 -0.56694819 2.505050057 1.78929197 -0.665773284 -0.784393773 -0.260448038 -1.035655062 1.232564446 -0.000426478 0.089739715 0.183809438 -1.010299865 -0.142455777 0.967123537 -0.245667777 0.655663769 0.267999899 -0.038863127 0.571198695 -0.147851281 0.355830024 -0.575396256 -0.086851254 0.514127268 0.524398642 -0.493532352 -0.156553685 0.23905458 -0.179433542 0.064847659 -0.403687473 -0.347589999 -0.252993023 -0.356439293 0.179665136 -0.316889317 -0.293807313 0.153899331 -0.119483179 -0.05326725 -0.268418677 -0.20569031 0.114707239 0.074737522 0.116326065 0.099520245 -0.055511169 0.189190065 0.158682764 -0.075231646 0.172259267 -0.080462807 0.206488423 0.222399817 0.009969483 0.050779737 0.293325381 0.155997814 -0.129537719 0.135005564 -0.11903213 0.166453744 -0.106066895 0.111332196 0.212314823 -0.206355304 -0.058390954 -0.183016532 0.116004041 0.058872922 0.003703815 -0.226913755 -0.392066539 0.098865205 0.104072222 0.002718211 0.09624076 0.17395717 0.031516214 0.011035791 -0.109427179 0.047901082 -0.088591029 0.012676713 -0.114465026 -0.201181255 -0.002216442 0.040475964 -0.15624537 -0.00263903 -0.011724787 0.137148592 0.097229793 -0.12176648 0.149773641 -0.04324967 0.052396805 0.046295812 0.195698845 0.130891233 0.121102361 0.133402497 0.031649653 0.167443367 -0.141373729 -0.059552219 -0.083135271 0.110138037 -0.057912678 -0.01939705 -0.047282177 0.162150277 -0.117674306 0.053988796 0.065679331 0.01083232 -0.064378231 0.078407351 0.095444869 -0.052627432 0.150420338 -0.130051306 -0.085389628 -0.058687597 0.065646622 -0.054771929 0.03299937 -0.1655133 -0.089411531 0.091451189 0.040302 0.129938735 0.02175176 -0.087691719 -0.022774745 -0.122183921 0.00630475 -0.11973933 0.115464786 0.103840881 0.043472657 0.048651989 0.016719739 0.087851764 0.005958013 0.019786405 0.004753864 -0.120881312 -0.019744121 0.009495928 -0.142562965 0.107229438 0.008022844 0.051877209 0.166826448 -0.030193061 -0.065417222 -0.149119878 -0.091895042 0.065687453 -0.081728779 0.021555787 -0.014986432 0.122917513 0.073854275 0.043269759 -0.090172859 0.004938625 0.078319854 0.060519156 0.008524836 0.041241841 0.086778312 -0.045502174 0.073865276 0.008629257 0.093621028 -0.056183185 -0.013531229 -0.124680385 -0.09104039 0.036179795 -0.090225258 0.073279591 -0.020312111 0.036747093 0.024029549 0.006525081 -0.009146885 0.067487749 -0.00019548 0.12917531 0.023568146 0.099288599 0.033472834 -0.063043133 -0.091362657 -0.004728257 0.025617547 0.013572717 -0.023548219 0.01169419 -0.073885757 -0.03088119

sample_30 0.54090971 8.132349174 -1.337715245 -0.012223553 -0.681303122 -1.43380525 0.441628759 2.362793457 4.759612372 0.921742965 5.65278508 -1.143828539 -0.091127922 -1.583494073 1.519346972 0.316868009 0.082217309 2.411792444 -1.324361744 2.300111014 2.237324078 -2.412610532 3.087556736 0.186168571 -1.328906815 -0.851078326 0.077390315 0.74933291 -0.188291153 -1.273890567 1.270117269 -0.138502222 1.60167887 -1.405485253 0.511643214 -0.565288707 0.769452089 0.375982485 3.088975253 -0.782260816 -0.413874331 0.75719544 -0.512648895 1.762639082 0.055123916 0.382194042 1.208529061 1.0302904 -0.63516293 1.257458263 0.098149477 0.947103165 0.984182737 0.654066522 1.725145729 -0.842070901 0.441764343 -0.990328624 -0.231730442 -0.992734705 2.027555095 2.104596432 0.283557241 0.068286891 0.122437691 -0.227638181 0.897622991 0.27325896 1.13126661 0.01431464 2.342218829 0.281751143 0.644799177 -0.085827024 0.051651107 1.554039025 0.480977464 -1.092416713 0.47004583 -0.306872162 -0.257798514 1.277144375 -0.193922074 -0.279901 -0.324875328 0.032946018 -0.040999251 0.275242264 0.736497795 0.451013346 0.028193844 1.204656817 1.042665041 0.100909288 0.288061211 0.99022749 0.284949289 0.741927434 0.439822132 0.395077802 0.238677085 -0.201567861 -0.212610989 -0.179543832 -0.096169556 0.073522605 -0.85953945 0.773764051 -0.47348465 -0.164815223 -0.3705953 0.302517091 0.533712301 0.560390755 0.321397816 0.16077208 -0.428995143 0.093903958 0.188396685 -0.190090863 -0.13819684 0.081419481 0.042023677 -0.402674382 0.439217361 -0.19380055 -0.144253931 0.105491213 0.115234814 -0.264628985 0.896960588 -0.083206403 0.979418484 -0.315128864 0.409807228 0.189706708 -0.142111425 -0.488919186 0.409945626 0.412734455 0.389306754 0.698753308 0.116904046 -0.022201789 0.072714753 -0.352680584 0.069560575 -0.05037606 -0.106566477 0.237429533 0.299539918 -0.184330395 0.087692688 0.197765181 -0.372041048 0.606235999 0.457267954 -0.273641571 0.566589891 -0.046061042 0.125886891 -0.212779047 0.491269778 0.431306895 0.248433145 -0.126031927 0.011670412 -0.048576282 0.089650381 0.559027036 0.207725184 -0.154297324 -0.103959173 0.339037514 0.174208882 0.061555595 -0.07565017 0.385107796 -0.172409213 -0.542987797 0.062415025 0.404388019 0.403043687 0.208968709 0.19837656 -0.03603551 -0.157114748 0.048118641 0.010215111 0.186574045 0.502366769 -0.107767842 0.609993976 -0.222664709 0.333036459 0.679047692 -0.131069354 0.246808009 0.399835556 -0.131344796 -0.639348669 0.083517915 0.341014271 0.599809755 0.458349394 -0.358085672 -0.147935102 0.004458507 -0.075371144 -0.136900368

sample_31 12.15201502 3.776058991 -7.150068125 1.021463175 0.052652074 0.740561102 -4.090803006 -2.206616509 -1.341126895 3.569078725 -0.176002992 -2.244673685 0.926324861 -0.307674343 -0.88059155 0.420668024 2.356971758 1.182073827 0.892491954 -2.717539904 0.388864101 -0.319955052 2.05606107 1.453470049 1.109191764 0.239816734 0.074714566 -0.006225651 0.604419389 0.066173265 -0.654157442 -0.47725338 0.010612581 1.230696809 -0.50988898 0.648144319 -0.035713368 0.248156789 0.290569918 -0.420252429 0.049476764 1.242415819 0.577945949 0.019581954 0.133997866 0.084576259 0.258451585 0.173365042 -0.614746031 0.334872991 0.303227053 -0.189882529 -0.498412755 -0.139062612 -0.169462203 0.139596181 -0.30226134 -0.20308021 0.227373898 -0.349535085 -0.245731826 -0.365342199 0.104945029 -0.289567455 0.560746425 -0.066868856 -0.51000792 0.305078068 0.043361287 -0.007679846 -0.04501069 0.009040237 0.220616092 0.303220531 0.159589035 0.165038268 -0.24485656 0.076355307 -0.097595207 0.026101708 -0.140814021 -0.257171559 -0.079896262 0.400307294 0.385294382 -0.265985119 0.256085748 -0.062555052 0.320738374 0.010656277 0.176512898 0.194714059 -0.247848143 0.196311694 -0.175152479 -0.108418455 0.097389558 -0.138059787 0.292956399 0.04916319 -0.018906882 -0.25305117 -0.085813035 -0.036036219 -0.026691771 -0.088948839 0.089741427 0.096700316 -0.087171044 0.133150966 0.200546084 -0.128759208 -0.173189606 0.253534362 0.199493363 0.12737802 0.274102988 -0.022104884 -0.127089731 -0.04144627 0.001725137 0.208703318 -0.069936926 -0.002095745 -0.080927541 0.051982392 0.075128823 -0.112012449 0.092217321 -0.040670876 -0.001115257 -0.043865908 -0.046898538 0.21311306 -0.116033325 -0.023596433 0.051115534 0.032399305 -0.008511294 -0.033536985 -0.055063625 0.006953247 -0.025210384 -0.204517133 -0.094187382 0.075094637 0.013675388 -0.025394529 0.032652756 0.071297661 0.076013866 -0.004121197 -0.011906221 -0.206247118 0.127987546 -0.080908821 -0.203765726 0.22163718 0.094722878 0.105475848 -0.030853266 0.088547731 -0.06065224 -0.074618719 -0.113483492 0.001976119 -0.026603173 -0.078370623 0.059207543 0.028840864 -0.019277422 0.011384702 -0.046437566 0.014653847 0.058591199 0.00099541 -0.028461874 0.052283102 0.015587996 -0.05211989 0.082059769 -0.057369377 -0.002203693 0.056450663 -0.014416611 -0.055284815 0.143926637 -0.016753723 -0.003011795 0.132184262 0.006015934 -0.095082611 -0.081672541 -0.083335392 0.015811606 -0.032227184 0.058218967 -0.064465246 -0.036460462 0.041751335 -0.05060154 0.024052348 -0.025422708 -0.023756113 -0.020178445 -0.031193639 -0.119175467 -0.05592349 -0.011212809 -0.055674305

sample_32 3.54964241 -1.849717684 -0.494923161 -1.687054804 1.877796646 -1.110775577 1.837908571 1.988832951 1.15098632 1.381308684 1.334698505 -0.30712046 1.226320588 -1.120587974 0.626015793 -1.01353354 0.707319342 -0.091277326 -0.878219053 1.19108834 0.544113907 0.118059069 -0.549174181 -0.265296971 0.801124812 -0.280314012 -0.437926137 0.041919459 -0.34830735 1.221179477 -0.91659352 0.709411024 0.722630381 0.263837055 0.225810037 -0.310418183 0.133147379 -0.063094899 -0.292864028 0.076738013 0.500075486 -0.374406525 -0.239327857 0.234811491 -0.793456854 -0.327228977 -0.087176959 0.487759555 -0.122606643 0.139969968 -0.195148599 -0.056415365 -0.870780698 0.310142002 -0.535425402 -0.206155386 0.364859986 0.064552761 -0.163722582 -0.005310889 -0.188723 -0.053641857 0.148907259 0.120290153 0.204395161 -0.146611531 0.115478839 -0.297979548 -0.047660307 -0.071981054 0.114237189 -0.253665914 0.253832062 0.123048854 0.220801817 0.011456813 0.084384212 0.033130084 0.460202455 0.0353053 -0.19021005 -0.199909245 -0.051828015 -0.00875649 -0.036764009 0.262836627 -0.269772386 -0.146802082 0.022758092 -0.066824978 0.045139349 0.070992144 0.039923767 0.144326788 -0.1668588 -0.055476196 0.040587305 -0.137394221 0.109154953 0.202105206 -0.164367643 0.044430201 0.289839625 0.344457582 0.131931513 -0.398107106 -0.038180725 0.070677088 0.081834578 -0.256584075 -0.135017833 0.091872748 -0.046963124 0.028158324 0.11608515 0.024848274 0.106306978 0.141596463 0.035353152 -0.039256205 0.159768154 -0.115373383 -0.139334964 -0.030454544 0.082690119 0.049410344 0.066181387 0.043739846 -0.054957175 -0.078745254 -0.054956681 0.133663518 -0.025194844 0.077022741 0.049170256 0.024050927 -0.085742235 0.065257466 0.04747796 -0.061751502 -0.075418775 -0.040725987 -0.055886439 0.038673606 0.030145789 0.077719115 0.080310413 0.060501805 0.143373222 0.047561496 0.012340938 0.030947668 -0.076785392 0.065730392 0.019020632 -0.037139721 -0.078435072 -0.024990592 -0.097076712 0.108563847 0.073680543 -0.027960628 -0.137947173 0.026673268 0.031005945 -0.009344848 -0.096248928 -0.009656887 -0.096620517 -0.00106397 0.007253494 0.107094254 0.020169659 -0.074881472 0.070352772 -0.063838716 0.12911475 0.118993698 -0.03821311 -0.088575633 0.004150641 0.119311461 0.064079136 0.023746145 -0.035607958 0.071282131 0.042581987 -0.030754935 -0.117784024 -0.068313824 -0.058465336 0.023625232 -0.11116341 0.009264268 0.000710777 -0.048366721 -0.039826026 0.031090713 0.064633548 0.089552932 -0.053615621 0.082013641 0.005664719 -0.094757003 0.022696999 0.010568163 0.009978405 -0.121677077 -0.013370873 0.034501199

sample_33 1.568042961 1.615563909 1.037369492 -1.352331504 -1.434198277 0.476250828 -1.84348328 0.40851273 -1.66257767 -0.159533073 0.850717052 -0.202890908 0.842752538 -0.464897171 0.642141832 -0.09653577 -0.758203389 0.336954737 0.519744094 0.32661171 0.384022245 -0.027283732 -0.333639976 -0.020129935 -0.738318099 0.754319095 -0.346898047 0.913443339 0.467921586 0.356988443 0.242681051 0.226830943 0.400843831 0.751662618 0.389797969 -0.177386998 0.426276392 -0.059102781 0.024002653 -0.138744774 -0.314981831 -0.115228442 0.612589989 0.330616061 -0.500762452 -0.106349347 -0.202630437 -0.126506057 -0.096719888 -0.233582177 0.022730718 -0.159748253 0.219703683 0.295301715 0.341596778 -0.137262232 0.036812086 -0.033780665 -0.501371738 0.078718599 0.196560691 -0.002589009 -0.089002893 -0.231433487 -0.1530894 0.406080938 -0.010392366 -0.153167631 0.098682718 0.115845716 -0.162919126 -0.145401669 0.039116852 -0.179981265 0.001734997 0.046572677 -0.339049564 0.090201105 0.129445719 -0.190219508 0.200476817 0.229954961 -0.169702752 0.25125144 0.17159485 -0.021570996 0.029085937 -0.138190183 -0.128241104 -0.131937208 0.002156023 0.125036728 -0.140765079 0.128996383 0.209774472 -0.140645375 0.055529006 -0.10396138 -0.27137861 -0.042417037 -0.052948002 0.11347069 -0.046343384 0.018819408 -0.02061377 -0.056422043 -0.035867515 0.103884336 -0.185955684 -0.120179408 -0.009671469 -0.084570663 -0.040711453 -0.068073178 -0.073742595 -0.030664335 0.019086209 0.141666459 -0.024111273 0.158781822 0.060421118 -0.022774826 -0.135606135 0.032546432 -0.101183516 -0.067708559 -0.021040081 0.155387779 0.035258188 0.077790213 -0.072294365 -0.073464571 -0.066757923 0.02688538 0.034384167 0.067882973 0.123771684 -0.134367256 0.04842908 -0.011869926 0.103206362 -0.103003053 -0.082496384 -0.009041105 0.019550349 -0.01504676 0.037161846 -0.0618256 0.018959758 -0.047130445 0.104083148 -0.007893689 0.022485346 0.016881092 -0.056432197 0.073620368 0.106858196 -0.039968476 -0.026134466 -0.022272098 0.036414348 0.088458428 0.02242278 0.003360179 0.092750793 0.04454208 0.128796513 -0.001057672 0.058334771 -0.01959167 -0.03604136 0.0307628 0.02546462 -0.042044318 -0.068880123 0.022289677 -0.060799959 0.060366636 0.034446347 0.03636981 -0.057600236 0.058662442 0.006547624 0.105150274 -0.049677167 0.054875012 0.05500159 0.045870323 0.014942961 0.08923976 -0.048843779 -0.056874023 -0.010295564 -0.101962566 0.01914865 0.003842528 -0.052714987 0.107125731 0.035036073 0.03984061 0.06477467 -0.035874106 0.049102053 -0.058990583 -0.07910289 -0.069461532 0.024492083 -0.032794999 -0.021613148 0.089282556

sample_34 -5.491958271 0.559375429 2.286785462 -0.422039318 1.367971248 0.623432617 -1.436994272 0.778806077 -0.707289145 1.984012425 -0.637444188 0.357668486 -0.685697448 -0.090413131 0.877784688 0.936505886 0.075268256 -0.706374425 -1.085147574 -0.931216105 0.303123419 -0.326625124 0.045462903 -1.017462743 0.082063083 -0.243291921 0.442417635 -0.399241543 0.31699412 -0.267011841 0.10952433 0.047201105 -0.287466017 -0.26086794 -0.236464724 -0.192997821 0.613638713 -0.034571571 -0.175044182 0.100496832 0.040835155 -0.124757183 0.019021448 -0.407805289 0.297329032 -0.076260704 0.366121682 -0.076763399 0.154675423 -0.317229086 0.001097767 -0.304067371 -0.065964095 0.035604684 0.10672112 0.14703904 -0.166430041 -0.15849315 -0.047680698 -0.090781457 0.095651543 0.180589432 0.227449043 0.150735639 -0.02683017 -0.016661849 -0.037451959 -0.131994672 0.215474897 0.132603841 -0.037019874 -0.09976699 -0.073487439 0.051364653 0.183041588 -0.081304997 0.112558154 0.173701533 0.116359859 -0.002898482 0.044703213 -0.094275769 0.005289779 -0.054793121 0.008252594 0.019389153 0.084114365 0.064655885 0.105510069 -0.014669488 -0.008759315 -0.10089743 0.073410499 0.019396561 0.102159373 -0.123719139 0.032506944 0.052195479 0.00775942 0.038676527 -0.023349963 0.071402545 0.136761256 0.034485267 0.045122252 -0.072759551 -0.067632311 0.007428599 -0.091539919 -0.001682493 0.095620662 -0.060884254 0.029939063 0.139056074 0.070841873 0.071568135 0.037441499 0.202036888 -0.051392641 0.026600701 -0.075843209 -0.036034314 0.058321499 0.012335973 0.011185765 0.084978736 0.051730812 0.002632455 -0.060927526 -0.065858596 0.119211595 -0.070561357 0.152678465 0.014397195 0.039645896 0.02232141 0.025944979 -0.030804927 0.029293532 -0.03543608 -0.028450109 -0.152039824 0.008876859 -0.017879496 0.107491277 -0.053458617 0.033408869 0.024648688 -0.00710859 0.008396868 0.04036404 0.125494508 -0.011697981 -0.004338949 -0.04273233 0.047523594 -0.015331568 -0.034556872 -0.032265491 -0.01084574 0.09846859 0.00460499 0.052465505 -0.025642369 -0.099411565 -0.020099967 -0.06114823 0.085934904 0.001540432 0.057493846 0.088410833 0.029132908 0.003679122 0.051211622 -0.031503621 -0.018865666 0.052840899 -0.010335126 -0.064436561 -0.025040618 -0.014498014 -0.049139994 -0.02919757 -0.079917959 0.115174135 -0.031647684 -0.002993688 -0.00836552 0.015114627 -0.012681212 -0.054278315 -0.037892287 0.004244901 -0.109248361 -0.055875473 0.018936636 0.003347717 -0.029585421 0.059466917 -0.01955824 -0.018131092 0.011126643 0.045665709 -0.054546619 -0.015721587 0.027817167 -0.0389655 0.007494267 -0.010932135 0.045281313

sample_35 6.470034757 -1.836045885 1.202209779 -1.247311263 -0.947571475 -0.890124964 0.038607261 0.159624295 -0.643812792 2.312974853 0.726405883 -1.151492383 -0.93810754 0.423348022 -0.041180933 0.702587491 -0.316207354 -0.463385757 0.719627886 -0.789325797 -0.279709415 -0.117813065 -0.259025421 0.16909776 -0.747243263 -0.12415084 -0.10087617 0.381052217 0.12273799 -0.041676885 0.474032921 0.100513523 0.881085857 0.7440982 0.092994987 -0.583245495 0.272385593 -0.009648553 0.446282341 0.374372286 -0.033213039 -0.057807328 0.747334724 0.374490842 -0.312937842 0.020361216 -0.139317232 0.256664482 -0.126725602 -0.212523482 -0.842877402 0.033413618 0.481488544 0.077373202 0.223953729 -0.077064373 0.025885678 -0.080794946 -0.224650918 -0.060264555 -0.197194589 0.045795737 -0.086835142 -0.201159338 -0.186169607 -0.162688549 -0.073961769 -0.109088973 0.005086687 0.226107929 0.010957386 -0.046166125 0.011304929 -0.02780326 0.19813145 -0.136121213 -0.084121889 0.045788203 -0.119189123 -0.258522939 0.220183907 -0.02976397 0.03270943 -0.16256981 -0.209881007 0.075355526 0.019233693 0.073782098 -0.23625382 -0.302344333 -0.004760116 0.021221129 0.073290864 -0.090996493 0.144761123 0.032799359 -0.050753633 0.027452954 0.047778523 -0.108724608 -0.204891875 -0.039397927 -0.147014665 0.133312643 0.026264942 0.034019394 0.04440849 -0.050052616 -0.02284924 0.198351016 -0.041724525 -0.043038319 -0.237242287 -0.126999051 0.002947007 -0.082445073 0.085353842 0.130832798 -0.080322612 0.057405346 -0.062322201 -0.028985543 -0.005270045 -0.007020114 0.025132892 0.100814285 -0.084907218 0.118194822 -0.09232716 0.104284361 0.022532161 -0.071945536 -0.125631303 0.020107161 0.048635475 0.038574091 0.113612448 -0.015548646 0.02843027 -0.004310893 0.011855818 -0.015894441 -0.060721429 -0.154604964 -0.043003275 -0.088089038 0.042775104 0.029319295 -0.116646893 0.076921949 0.11343714 -0.01526462 -0.138393682 0.163848766 0.104657358 -0.010093448 -0.065048116 0.034370572 -0.101739189 -0.129113269 -0.038766707 0.26215422 -0.133578785 -0.044961995 0.099652547 -0.150173935 0.056124055 0.046238078 -0.035002315 0.013829652 -0.021218742 0.05032292 -0.000420946 0.15815626 0.017242347 -0.01233659 0.036119215 -0.001042045 0.006867564 -0.052762336 -0.035150279 0.013633951 -0.099860138 0.030576358 -0.061068849 0.073145761 0.004254361 0.032214378 0.030410999 0.129331909 -0.020233576 -0.114243402 -0.139963474 -0.037910095 0.02998547 -0.035432967 -0.036429057 -0.013214476 0.027889226 0.021945194 0.08215263 -0.00495326 -0.101470606 -0.037272844 0.127227317 -0.006836798 0.074659601 0.183187194 -0.023425818 -0.033106961

sample_36 -1.251652502 3.82649093 0.180370745 0.155988455 -0.842626068 0.182494759 -0.348417801 2.019830022 0.133055118 -1.145608999 0.542580177 -0.048538377 0.16846626 -0.334332251 -0.344349084 0.015552319 -1.914436756 -0.517918579 0.898893801 0.338203982 0.072775023 0.309981221 -0.124100866 0.009542409 0.712300104 0.191969024 0.287430991 0.173435983 0.416584821 -0.490643421 -0.701165343 -0.037752613 -0.287508199 0.085902544 0.176826512 -0.135307232 -0.244718576 0.494514343 -0.063422669 -0.183336047 -0.227884427 0.508541353 0.361965757 -0.221940103 -0.213453118 0.002113206 -0.02014591 -0.234540541 -0.286146439 -0.164750113 -0.036642771 -0.134449885 -0.429492013 -0.041463847 0.107163361 0.039108361 0.287734313 -0.133275528 0.008090339 -0.265390861 0.20151692 -0.199163687 -0.456062616 -0.227499216 -0.0751192 -0.064043318 -0.080147767 0.218575716 0.076684329 0.032795203 -0.095783373 0.005957176 0.168920568 -0.072848568 -0.477945691 0.108120659 0.158648885 -0.186698123 0.185601897 -0.02736478 -0.10748394 0.011592714 0.171714655 0.065960225 0.269213078 0.177922743 -0.002448444 0.215171139 0.050030537 0.239086269 -0.083341952 0.053484795 -0.215963111 -0.103410746 0.121510146 0.015493904 -0.103490473 -0.070754849 0.149418568 -0.143842618 0.066378097 0.152537199 0.141714905 0.242067436 -0.081854619 0.022911187 0.00641673 0.036106454 -0.060708724 0.290109926 0.06713822 0.070474023 0.099416899 0.118466154 -0.038398338 0.077881246 0.021701088 -0.14317345 0.042796006 -0.058406265 2.05E-05 -0.09471396 0.009600211 -0.004924037 0.025155912 -0.036844034 0.084157172 0.00714448 0.124733484 -0.071111486 -0.046592735 0.000645426 -0.03035753 0.146701959 -0.105086779 0.095825531 -0.049959018 0.042183942 0.20064357 0.026827542 -0.075864982 0.045969927 -0.131430987 0.040738022 0.018061542 -0.079361727 -0.104789778 0.032932244 -0.070255112 -0.071316043 0.04204901 -0.123455745 -0.055198311 0.006357466 0.020354175 -0.028323847 0.01165425 -0.09894619 -0.017374779 0.085044058 -0.040852186 0.074205813 0.033792116 -0.030874574 -0.130377761 -0.102008519 -0.052805969 0.012829418 -0.022069524 0.035301772 0.082481224 -0.021969156 -0.028965479 0.148738219 -0.078001166 0.041461247 -0.015779335 0.031641461 0.019271984 -0.081447021 -0.02991293 -0.051413537 -0.014224829 -0.068402266 0.049912842 0.096731187 -0.000974285 0.043655461 0.085346163 0.060170937 -0.000933345 0.066935668 0.022117397 0.022856166 -0.112349113 0.075970485 -0.067878918 -0.022695498 0.055352636 0.007324817 -0.076912671 0.007429475 0.045736714 0.059130673 0.053466437 0.033897979 -0.02157083 0.025240445 -0.013759721 -0.004167361

sample_37 0.918864142 -1.876232355 -0.277675987 -0.028938034 -3.719691939 -0.545359954 -0.813250968 1.75528847 0.958720395 -0.021176726 -0.001817124 0.201000319 1.079624229 0.142629471 0.154270881 -0.048940634 -1.388488741 0.034452203 1.030147666 -0.206412245 0.556413423 0.124167399 -0.862719832 0.447319115 0.18559182 0.42949791 0.527864744 0.332980137 0.501714841 0.523377067 0.252924431 0.218768338 0.320026458 0.031897449 0.378998848 0.16394878 -0.021420455 -0.185887157 0.195048817 -0.094207057 0.246412304 0.137292465 -0.275034618 0.021936241 0.034589893 -0.237820615 0.219961973 0.205451656 0.307092174 0.058125102 0.149342419 -0.27916178 0.013590534 0.109912792 -0.070365446 0.091846111 0.115132447 0.111677539 -0.184207453 -0.018614778 0.029817793 -0.317683037 -0.14103766 0.263072356 0.041399728 0.106571243 0.283060161 0.137815958 -0.078867378 -0.314622394 -0.014461837 0.02243304 -0.173278076 0.03178235 -0.081817311 0.004905261 -0.071684943 -0.07766763 0.100126311 0.094632825 -0.016777507 -0.125991618 0.018405533 0.008072263 -0.019559027 0.032693239 0.064782658 -0.058240715 0.057301599 0.06329259 -0.255199682 0.043566314 -0.036722965 0.090103213 0.02303611 0.075347697 -0.01485863 -0.042656286 0.189249124 0.046244329 -0.118444997 0.041995683 0.091925491 0.049450537 -0.037035689 0.147001427 0.11106862 -0.030405775 -0.128152334 0.204335055 0.192937321 0.008676334 0.126863136 -0.042954041 -0.001638766 -0.027297999 -0.018531485 -0.02051138 0.126632807 -0.098708298 -0.048948649 -0.125382739 0.125127795 0.02146639 0.143299468 -0.086928263 -0.222777034 0.012334304 -0.094161998 0.025012386 -0.001895483 -0.128090173 0.013614909 0.025112573 -0.017490458 0.0191415 0.153943241 0.126783488 -0.086109423 -0.077253707 0.032501685 0.017635565 -0.090173024 -0.094943726 0.031478179 -0.059993627 -0.036277205 -0.059238623 -0.07378821 -0.086694383 0.064962484 0.066697804 -0.045044542 0.022843385 -0.084646744 0.083579651 -0.061788727 0.055525689 -0.021182906 0.027402926 0.108033394 0.154495285 0.116603617 0.010223924 -0.074667063 -0.01319805 0.0629434 0.067612527 0.111562487 -0.08770661 -0.041151796 0.030629321 0.042307631 -0.082553508 -0.159921023 -0.003632669 -0.02587886 -0.060501751 0.045704627 0.018997564 -0.043929722 0.026979445 -0.021235517 0.029776517 0.059387736 0.064582643 0.000124547 -0.020549472 0.005783309 0.01234132 -0.047486881 0.077422209 0.019376011 0.016115657 0.001278018 0.056364081 0.066691547 -0.031719193 -0.036596789 -0.000910183 -0.031580201 0.070526951 -0.050212073 -0.101596403 -0.010849265 -0.041709381 0.051079883 -0.01342843 -0.036411305 -0.022477784

sample_38 -3.391477901 -1.665586657 0.893243115 -0.368100141 -4.673501335 -1.132272833 -1.673710288 0.407622516 1.411091833 -1.203685629 0.184227032 -0.123240981 0.152814208 -0.836828805 -1.245081573 -0.906115157 -0.013765966 0.142428752 0.182538893 -1.296910735 1.197285325 0.29832007 -0.688330363 -0.332237692 -0.295424376 0.450956957 0.079540588 0.069327945 0.286384929 0.211800622 0.365361727 -0.503062198 -0.479805345 0.161656593 0.426270465 -0.476012851 -0.258748341 0.477935513 -0.23071054 0.340938961 0.048214768 0.286639398 0.009630226 -0.241153436 -0.22043172 0.041552817 0.123920955 0.382029458 0.459658078 -0.245571712 0.575008228 -0.506041453 0.118382998 -0.052033144 0.027044042 0.055654554 -0.411343774 0.184118883 0.204786639 0.180221907 0.2501964 0.039017986 0.349432518 -0.175826257 0.105790651 -0.13284319 0.178909552 -0.396318844 -0.064841273 -0.089609081 0.034964371 -0.15976543 -0.001300069 0.060613402 -0.159992936 -0.259198765 -0.013422672 0.161237317 0.177131128 -0.259811932 -0.107643647 -0.066314927 0.364639086 -0.098600345 0.113981087 -0.025725912 -0.057408491 0.143659582 -0.067099275 -0.187782353 -0.151043297 0.045835096 0.166313174 0.076068585 0.105353726 -0.072370382 -0.220670338 -0.11837499 0.448944654 0.323193124 -0.09579091 0.168046499 -0.227826065 0.070564903 -0.352859509 -0.061281477 -0.100733267 -0.11183612 0.003970421 0.043882733 -0.020531801 -0.139486071 0.097881591 -0.011249063 -0.071642498 -0.112461516 0.020039147 -0.215417823 0.144183462 -0.091281115 0.135134702 0.076889258 -0.105115646 0.031810904 0.092562001 -0.14790724 -0.167052648 0.199567785 -0.064363011 -0.03104171 0.017188896 -0.022728109 -0.0812324 -0.10500128 0.071611528 0.003234715 0.052998002 -0.011134463 0.061078725 -0.055605734 -0.046846716 0.005641526 -0.007839409 -0.033783421 -0.085323472 -0.170554605 -0.011803373 -0.154435311 -0.03339852 -0.196575141 0.023767831 0.026317351 0.083703297 0.044045212 0.054998765 -0.003883642 0.199916324 0.002136918 -0.070665264 -0.052978618 -0.148270207 -0.099601453 0.067377747 0.089486672 0.048699207 -0.0271709 -0.065946364 -0.12841498 0.132856322 -0.090524744 -0.112452377 0.056806806 -0.022982867 -0.019711499 0.084672878 -0.001051075 0.064711341 0.017506795 0.207312049 -0.207387745 -0.039859477 -0.015563989 0.007798292 0.097715228 0.000449882 0.117851504 -0.003392813 0.089109811 0.107764485 -0.091279758 -0.010976736 0.149327331 0.052717448 0.024806455 -0.044796764 0.011223943 -0.060225824 -0.085421017 -0.134955603 -0.118828891 0.0104921 0.087819664 -0.021635219 -0.162729352 0.062464123 0.011658095 0.094877718 -0.054276696 0.019971847 -0.103760051

sample_39 1.440699413 -1.476765037 -2.644977538 -0.179976911 0.685474226 0.201951983 2.198505351 -0.62153023 0.270276323 -0.607068566 0.006785183 -0.739065533 1.615488014 0.464904275 -0.441805788 -0.228638818 -0.771976549 0.20111391 -0.241991829 0.582577845 0.006064701 -0.315274918 0.096123075 0.60499178 -0.182029726 -0.592957538 0.352072537 0.125384307 -0.351136855 -0.35633208 -0.586317373 0.500582298 0.809380876 0.068764046 0.097602839 -0.330412357 0.132105494 -0.187094724 0.145215187 -0.132363104 -0.122663795 -0.264712563 0.104263182 -0.203952726 -0.094000833 0.315657349 0.135664135 0.077808188 -0.060776489 -0.077643409 0.181399419 -0.119012677 -0.758809866 -0.121485197 -0.036644402 0.074182961 -0.216413459 0.075067801 -0.113478331 0.011468848 -0.051872688 -0.113191664 -0.011289711 0.428145264 0.034698253 -0.203562014 -0.056285756 -0.328952324 -0.142214836 0.046386678 -0.07936709 0.068781666 -0.1210144 0.202048028 -0.1130425 0.10102369 0.144891147 0.025093141 0.346514369 0.101395664 0.040114561 -0.101307404 -0.136163304 0.045958652 0.096005953 -0.059204766 0.119972537 -0.214320041 -0.095686766 0.157692655 -0.359857127 0.179945102 0.048326029 -0.120380719 0.08547941 -0.166411386 -0.060141777 0.069603533 -0.013504117 -0.002889902 -0.056907151 -0.21015484 -0.150285887 0.031421495 -0.022705042 -0.129572499 -0.052923283 -0.012285563 -0.038088331 -0.031426996 0.072627263 -0.050807877 -0.186650928 0.056623353 -0.073226998 0.004461279 -0.110056337 0.039769593 -0.013653235 -0.042622051 -0.031288891 -0.042620598 0.037926958 -0.001675131 -0.00264927 0.000383499 0.033040048 0.00319315 -0.057942375 -0.003498608 0.081678954 -0.022351787 0.005716148 0.098897356 -0.157775976 -0.021231592 0.182703344 -0.078648418 -0.067335867 -0.013621284 0.01449721 0.027675844 -0.207249634 0.037851113 0.049806053 -0.055805767 0.055942204 0.017135236 -0.066573955 0.007511423 -0.033227376 0.029124745 0.075716471 0.017313254 -0.096847206 0.098697151 0.03228365 0.139221911 -0.129878548 -0.063542054 -0.125688926 0.022972056 0.039403779 -0.011415534 0.015652337 0.011228286 -0.03631663 0.013048195 0.064034232 -0.051383805 0.039841892 0.041452513 0.004815594 -0.007912406 0.045516596 0.039031791 -0.022315789 0.000491274 0.028738266 -0.012036764 -0.015968835 -0.026926294 0.017050789 -0.045406056 -0.029203494 0.093579975 -0.036443354 0.040129708 -0.014845549 -0.012605021 0.000980439 0.012284489 -0.053601743 0.005208086 -0.038286113 0.031662968 0.086040268 0.013119489 -0.076576197 0.009176099 0.055617074 -0.01144742 -0.000912544 0.009649835 0.086491157 -0.007237288 0.013552595 -0.04453742 0.016220007 0.098536301

sample_40 0.825349194 -6.537105774 2.852459689 -0.173575532 -1.486897158 -1.936854019 1.421586133 -1.249426719 1.16956734 -0.246209268 1.060621762 -0.67315124 0.837356758 0.157810912 1.390014808 -0.517657001 -0.296013535 -0.280087366 0.853094924 -0.228614498 0.233832083 -0.124279948 -0.062780968 0.417491289 0.980834823 -0.675913788 -0.691940104 0.203781226 -0.126636206 0.18094852 0.158074806 -0.62697576 0.366604169 0.003707371 0.082481276 0.305412337 -0.017817291 0.301507334 0.220735894 0.267067545 0.013418993 -0.097394003 -0.273285891 -0.582456685 0.377611334 0.098345838 0.068282543 0.138072583 0.041316599 -0.298892441 0.364135221 -0.121201541 -0.037252588 -0.120961169 0.017142948 -0.13769134 -0.110365356 0.081797047 -0.06965518 0.27703533 -0.152112237 -0.272291617 -0.077207293 -0.012066284 0.046692827 -0.146491223 0.013888495 -0.074012407 0.129095812 -0.229496454 0.204117701 -0.075516112 0.090951394 -0.120277673 0.216281126 -0.047389663 -0.116122273 0.006095348 0.17727268 -0.073292521 -0.006550756 0.071355391 0.016712241 -0.234393562 0.210130623 -0.242682917 -0.075068616 0.024134528 -0.017363796 0.049936495 0.121227397 -0.05030265 -0.004079768 -0.012797498 0.12867764 -0.166821972 -0.078637863 0.077610776 -0.12743807 -0.016415749 -0.062461295 0.143857981 -0.142712679 -0.108193278 -0.138790275 -0.042653436 0.074419984 -0.093586132 -0.090159467 0.122947156 0.019478712 0.093266263 -0.00640039 0.044189941 -0.108596863 -0.031897767 0.046086048 0.036424062 -0.033307372 -0.062488284 0.014988317 -0.00993496 -0.027361619 0.160866787 0.037144087 -0.051619598 -0.053202681 -0.053559404 0.038500488 -0.030083321 -0.025845254 -0.064945335 -0.020619106 -0.021116221 0.041686438 -0.130139166 -0.041377813 0.038361528 -0.076925179 0.070257625 -0.05842886 0.005696122 0.057456959 0.067309907 -0.160833621 0.06392298 0.039422045 -0.013587628 0.056044719 -0.109824825 0.028507853 0.02142158 -0.041375092 0.053984985 -0.040664175 -0.042514381 0.038483608 -0.114525136 -0.097526308 0.064068432 -0.051318433 -0.064893827 0.024403377 -0.02602423 0.012287272 0.052680384 -0.05491136 0.036188625 0.044239392 0.060135421 0.035397906 0.086459258 0.000991122 -0.038235448 0.108307829 -0.043507728 -0.035128554 -0.084487863 -0.03981202 -0.002834111 0.106480528 0.004814796 -0.029401794 0.007241415 -0.023636003 -0.013932488 0.011218989 0.081608722 -0.074216191 -0.020191819 -0.04512646 -0.041149713 0.052620828 0.00258295 0.005198363 0.026545434 -0.023417667 -0.072440053 -0.040986901 0.018882529 0.013853328 -0.018901695 0.069140653 0.024147968 -0.062285341 0.008415757 -0.006693993 0.015150024 0.008437795 -0.023781965

sample_41 -6.788564523 0.255009105 -0.984389014 1.399374374 2.69187864 -1.010927692 0.391484316 -0.849854962 1.081694514 0.677021918 -0.194226188 0.831020455 0.862073811 0.991382954 0.410123713 -0.88216028 0.461702501 -0.447122119 0.939269539 0.169654486 -0.351871687 0.670455979 -0.012393688 0.512748661 -0.603710792 -0.077951194 0.116131928 0.313291825 0.175213619 0.110392359 -0.241442959 0.331356583 0.28317293 0.317667897 0.543490004 -0.08649381 0.006089583 -0.513251277 0.272940576 -0.154446963 -0.198162675 0.492977022 0.098345131 -0.188645252 -0.048227778 0.004249159 -0.26669282 -0.000318372 -0.012061295 0.09819567 -0.268881636 0.131538838 0.239224194 -0.11667943 -0.013995477 -0.057913571 -0.100232663 0.007350483 0.257626838 -0.178851493 0.04538168 -0.293222825 0.058394259 0.173732817 -0.144301395 0.10252776 0.172478491 -0.029748453 -0.029537469 -0.056953551 0.041408436 0.144593754 0.069183247 -0.205173612 -0.042173277 -0.023700321 0.079467978 -0.052557149 0.090370765 -0.128680157 -0.04769512 0.004090017 0.039552896 -0.065099936 0.020251004 -0.102186193 0.006064545 0.107344365 0.081380575 -0.265638436 -0.030794758 0.005286749 0.07988185 -0.074775593 0.057312211 -0.047194019 -0.051706364 0.121993437 -0.085622805 0.021791088 0.026491351 0.035557469 0.071960572 0.025417623 0.061401723 0.007566887 -0.065043984 -0.062077938 0.061744083 -0.022577481 -0.005065382 -0.003788999 0.104386275 0.004733576 -0.009244609 -0.027114628 -0.050522288 -0.101145431 0.043237355 0.018481893 0.01169461 -0.04939508 -0.195024929 0.008782612 0.080542991 -0.047586191 -0.020934073 0.077771298 0.02787265 -0.048336041 0.076060588 0.039226104 0.00036361 0.035749858 0.013137451 -0.020469037 -0.073860708 -0.023423731 -0.138330393 -0.027838998 -0.030724894 0.033583509 -0.004757459 -0.035753622 0.008119254 -0.054438097 0.054852255 0.063920409 0.039578106 -0.050742555 0.069851444 -0.062466781 0.010608727 -0.046493504 -0.00133968 -0.046003976 -0.039126639 -0.002689583 0.012841047 0.024438069 -0.044201155 -0.004162474 -0.045613444 0.01777258 -0.050072131 0.081167008 -0.051109823 0.098584015 0.027771677 0.047336094 -0.071711043 0.043965872 0.044025865 -0.042129386 -0.048759167 0.063374381 0.013643612 0.028572316 -0.003962783 -0.006929724 -0.090723494 0.053586291 0.009989401 -0.053125786 -0.109876282 -0.052099709 -0.023776774 -0.021150735 -0.007369988 -0.020057189 0.013995836 0.082884913 0.027295101 -0.036683503 0.046702883 -0.031837935 -0.008927514 -0.067053079 0.007238281 -0.024329808 0.016586028 0.088575942 0.013718029 -0.011033379 -0.016335753 -0.040084544 -9.74E-05 -0.013775468 0.021363861 -0.01932707

sample_42 4.682381932 4.210611203 -0.072428295 -1.313834763 -0.30615719 -1.559589372 -0.880284315 0.035187881 0.212420245 -1.386617508 1.102727955 -1.756313145 0.885859454 0.304086673 0.816367641 -0.039834485 -0.797524878 -0.5248123 1.270869613 0.635593569 -0.375325781 0.467840617 -0.607664627 0.292044868 -0.191130294 -0.071696574 0.541149855 0.298521354 0.286913483 0.161917816 -0.012261142 0.524076367 0.025998292 -0.385091034 0.076736048 -0.594953467 0.588660393 0.251011372 -0.477403597 -0.596619419 -0.35689249 0.027091407 0.262575326 0.142402238 -0.183423106 0.001098159 0.244877704 -0.662562577 0.348006318 0.167675638 -0.240968487 0.122227013 -0.52255247 -0.000947091 -0.07115471 0.303730043 -0.162785407 0.010406122 -0.392584237 -0.046808929 0.10002785 0.061495613 -0.049259703 0.031495473 -0.238448871 -0.167720119 -0.014604759 -0.405135725 -0.127375932 0.006441798 -0.032752232 -0.082179949 -0.240693486 -0.025736044 -0.305272306 0.064734744 0.136712115 0.186916808 0.16161371 -0.102989276 -0.172696618 -0.036488295 -0.161729704 -0.014232888 0.069370981 0.210456545 0.223143965 0.076841236 0.035115554 -0.04533567 -0.091054232 -0.004263884 -0.14207348 0.035005649 -0.043166367 -0.063395733 -0.146292691 -0.027202015 0.12729906 0.146871945 -0.172904345 -0.058651152 -0.294409971 -0.004063146 0.033031081 -0.065758896 0.001398448 0.083387625 -0.093444888 0.02826559 0.099271076 0.029337795 -0.03848619 0.029399479 -0.144874321 0.127445224 -0.040725755 0.038348981 -0.141910727 0.071138764 0.048140646 -0.024493482 -0.123553478 0.098513007 -0.010358788 -0.010770795 -0.037523734 0.021541083 0.086517616 0.0479437 0.037681079 -0.08113286 0.086689618 -0.094474149 -0.020560465 -0.062523291 0.019771554 0.112741788 -0.019025623 -0.037761677 0.103313397 -0.064515594 -0.106925097 0.009140904 0.081651754 0.04922463 0.03321566 0.025994076 -0.007871495 -0.022809015 -0.00766009 0.062006729 0.075890147 -0.012040706 -0.030535302 -0.002729461 0.075925819 -0.072190453 0.006637855 0.040904742 0.016241552 0.063867807 -0.031259725 -0.098129676 -0.05278196 0.036687934 0.059589102 -0.005879867 -0.005614252 0.047809736 0.025456681 0.070080167 0.010135204 0.021584393 -0.07140759 -0.052738746 -0.150396808 -0.07724397 0.040181798 -0.063226071 -0.086728554 -0.096471756 -0.051237507 -0.016481069 -0.064259742 0.083221225 0.10187724 0.065576084 -0.018478446 0.017470341 0.032721367 0.024581574 -0.073437046 -0.050589449 0.054831045 0.002719025 -0.040467806 0.000672759 -0.037255926 0.021627017 0.032049555 -0.024000048 0.003703633 0.064160206 0.110929011 -0.06247666 -0.028122111 0.01831125 -0.008858332 -0.005713644

sample_43 -1.455225018 -1.583157434 3.297341175 -2.923157686 0.70351941 -1.329098701 -0.992467104 -0.468295572 -1.206760715 0.190520808 0.955288648 -0.207221576 -0.408582156 -0.586013184 -0.128250811 -0.407281959 0.027896541 0.629385031 -0.72965617 -0.219121655 -0.835298048 0.072167118 -0.175385975 -0.378081781 0.04856526 0.035700235 -0.161577339 0.113964045 -0.171200489 -0.010759878 0.236720343 -0.1266597 0.163689553 0.191019395 -0.290472834 -0.09510008 0.013384101 -0.032991328 -0.359250673 -0.00269011 -0.007949599 -0.255889739 -0.073029788 -0.002657462 -0.04310299 0.123138376 -0.046497912 -0.01660888 -0.0631331 0.091480574 -0.102446824 0.191488967 0.153749793 -0.024501519 0.222149163 0.096819998 -0.272026929 -0.189792463 0.028447505 0.16324406 -0.078075584 0.215417591 0.093918119 0.058329632 0.096682315 -0.045691338 -0.024681503 0.02125779 0.137058864 0.122440282 0.032597847 0.029997179 -0.150954173 0.05117275 0.177960077 -0.01922174 -0.021452169 -0.030544036 -0.048266546 0.044912955 -0.12783851 -0.007477695 0.156834288 -0.046944937 0.126532817 0.094687933 -0.01587002 -0.152997483 0.210911692 0.014426325 0.095979484 0.012418902 0.049422913 -0.010697778 -0.031275293 0.021009452 0.112535642 0.145213716 -0.023311487 -0.16565802 0.06722096 -0.0511325 0.152187888 -0.053943032 0.039171563 -0.010586344 -0.046112426 -0.001556122 0.006660277 0.016001537 0.052343968 0.047165746 0.162678562 0.042263992 -0.001923802 -0.002232456 0.04954349 -0.081669182 0.016262472 -0.025391266 0.005318049 0.00462821 -0.174249835 0.019666089 0.107097876 -0.15257595 -0.029499051 -0.02830337 0.110291222 0.042851255 0.01993581 -0.024921408 -0.032967671 0.011785705 0.006446843 0.037710701 -0.026873092 0.015243453 -0.055237252 0.027396681 0.055356835 0.028174891 0.001834082 -0.018561499 -0.074605405 0.017182764 -0.075618791 -0.045428314 -0.083702408 0.067752057 0.004611193 -0.048970188 -0.074982494 -0.035829219 -0.000337936 -0.004416048 -0.092812886 -0.007732059 -0.013916441 0.075103514 -0.06788856 -0.018818627 0.063661153 0.025663663 -0.05209582 -0.002456156 0.128086265 0.00533371 -0.001088301 -0.059762565 -0.013557033 -0.043091755 0.007435197 -0.03801956 -0.014967214 -0.04598006 -0.044894558 -0.0117893 0.019587919 -0.068074597 0.019831555 0.008723324 -0.023021055 0.064518569 0.055269803 0.009447811 -0.040190204 -0.015041192 -0.022365509 -0.021987367 0.016059326 0.037048583 -0.066508429 0.0163241 -0.001823616 0.052621016 -0.02064814 0.050196743 -0.059299747 -0.108181279 -0.06766118 0.00508126 0.06974528 0.034569003 0.005982172 0.000204484 -0.026300344 -0.069808341 -0.063620087 -0.060780576

sample_44 -1.622014555 -4.298238889 0.307866572 0.789659325 0.503406473 0.687835902 0.647044993 0.252195308 -0.050087538 -1.325305329 -0.513860628 -0.054041254 -0.540409389 0.207235075 0.032046802 -0.277671346 0.355884585 0.452720347 -0.739388953 0.671503355 -0.364433548 -0.166447054 0.669725011 -0.181719514 -0.091924684 -0.574865715 -0.038857278 -0.426938701 -0.83183809 -0.208767537 -0.096818895 0.014144284 -0.246787077 0.487314131 -0.231490942 0.299371318 0.488618022 -0.034286549 0.1161392 -0.217127552 -0.102149183 0.233309582 0.079689164 0.2048816 0.310400431 0.140747677 0.061210487 0.185924345 -0.448623717 0.025834027 0.13274332 -0.0789678 0.021247304 -0.154723604 0.207944395 -0.103899221 -0.114085042 -0.163929816 0.003434458 0.146020698 0.031107409 0.254630548 0.119074148 0.156765237 -0.084153973 -0.188011597 -0.101622214 -0.018732337 -0.141418537 0.166763145 -0.192011581 -0.192854173 -0.002933732 0.122090043 -0.221511361 0.105495284 0.073145951 -0.006693252 -0.113631253 0.096499577 0.10449576 -0.041726143 -0.107320906 -0.150284373 0.00438317 0.119563448 0.066283889 0.144616809 -0.029765952 -0.08734796 0.029678577 0.057002397 -0.039423004 0.117717843 -0.082575546 0.022759137 -0.142954925 -0.012745805 0.073954337 -0.065888931 0.125925244 -0.117753431 0.001662498 -0.014535346 -0.033471673 0.149786514 0.046404247 -0.011904752 0.136382584 0.180904976 0.040756535 -0.115577409 -0.096408605 -0.012938377 0.004099434 0.069438753 0.041338992 0.07738263 0.008336406 -0.130475082 0.104593062 0.008752048 -0.013503045 0.033730905 0.029022888 0.0036493 -0.054196575 0.012249961 0.083153831 -0.06301423 0.004380904 -0.044137364 -0.045426902 0.002077141 0.156464984 0.084866868 0.038783139 -0.043104752 0.066706395 0.042482795 0.054127583 0.014446601 0.013450362 -0.009295538 0.044690005 -0.016257062 -0.062520589 -0.0727258 0.007212644 0.063333821 -0.034140663 -0.085464207 0.087576195 -0.034062952 -0.011288249 -0.112556952 0.070798619 0.068633379 0.060355636 -0.009114223 -0.035097767 0.036883923 -0.006377898 0.002719004 -0.066656736 0.004944776 -0.011617346 -0.046475471 -0.027737042 0.080380217 -0.054426337 -0.06049385 0.083370509 0.046283925 -0.013594255 -0.040855455 -0.013919753 -0.034898526 -0.057060284 0.007975807 0.014548998 -0.086743829 0.017067625 0.067590516 -0.067407685 -0.008876209 -0.003541975 -0.012908708 0.023719048 0.002327648 0.023580936 -0.014490729 -0.051589759 -0.022592541 -0.009587235 0.01027633 -0.057926335 0.022017517 0.009428704 -0.006508266 0.02403361 -0.027693638 -0.034296781 0.054289337 0.046493119 0.023023046 0.007441301 0.099304233 -0.03579327 0.035041447

sample_45 -0.20681909 1.072987664 3.315832666 -0.866738028 -0.884545674 -0.454319887 -0.084012406 0.418817945 -0.977344229 -0.760464805 0.726823627 -0.389880949 -0.090615561 0.392541849 -0.465611677 0.272684104 -0.598812861 -0.418483468 0.637437743 0.087462587 -0.827296684 0.147254685 0.233285885 0.063285053 0.227565388 0.340738487 0.119035962 0.464781054 0.178733796 -0.223359528 0.177758223 0.168181289 -0.254502135 0.053826119 0.108835109 0.115942554 0.738347977 -0.420451063 -0.327802348 -0.246276025 -0.228630018 -0.166523433 0.151808064 -0.031567763 -0.018778883 0.186827167 0.43339533 -0.180455547 0.240089136 -0.084929135 -0.031819497 -0.032664491 -0.178017748 0.130388382 0.20854079 0.141403946 0.1910811 -0.248642547 -0.057591045 -0.076190663 -0.115538423 -0.203761611 -0.410288884 -0.153153575 0.060774334 0.323253434 0.104605849 0.019540017 0.281399961 -0.053614148 -0.115178089 0.031483035 0.118820796 0.080401119 -0.058345671 -0.031533947 -0.099026049 -0.2012966 0.1005313 -0.051636769 -0.032035745 0.148335058 -0.329991613 -0.016338584 0.050595557 -0.07309409 0.106536982 -0.065497074 0.13039852 -0.062195535 0.190569195 -0.065298591 0.004633401 0.07534312 0.022016164 -0.03226611 0.008494494 -0.105533822 0.113944497 0.031611596 0.185217588 0.048944978 0.181148895 0.043570604 -0.176441996 -0.18133231 0.007768943 -0.094542373 -0.014265384 0.036396921 0.008747021 -0.096919132 0.021126292 -0.022885093 -0.13405158 -0.079468003 0.031026171 -0.007401694 -0.085269205 -0.098382215 0.007272063 -0.128267953 -0.01976253 -0.025885984 0.007240555 -0.026854972 0.059492864 0.185854087 -0.084105944 0.09235035 0.132173716 0.025483765 -0.009862646 0.076312339 0.090515743 -0.008046808 -0.031249314 0.032578309 0.110664402 0.132527391 0.096801126 0.010124762 0.080581146 -0.072222499 0.034410756 0.046719695 0.044360024 -0.056285277 0.119685776 0.064643224 -0.063293201 0.0722933 0.018277505 0.037042049 -0.040976967 0.057775481 -0.019836801 -0.017686056 0.007703074 -0.08893258 0.030643254 0.071331877 -0.059086832 -0.032448689 -0.026551825 0.009707366 0.071101915 -0.026519474 -0.096781749 -0.045749914 -0.026304768 -0.024502945 -0.016781327 -0.130661843 0.040924531 0.049976082 -0.038867071 -0.024686377 0.019541602 0.04945815 0.009302267 -0.040917997 0.051056825 0.020201651 0.123287158 -0.026426041 0.027389016 0.014372373 0.015402044 -0.00385856 -0.010778658 -0.069095272 0.045481646 0.044502439 -0.023042587 -0.004342718 -0.123358282 0.034146718 0.014529213 0.061139784 -0.011380113 0.007795322 -0.041071669 -0.035233588 0.053308184 0.02165744 0.063940786 0.030920879 0.077917799 -0.002830825

sample_46 -5.919593295 0.921539064 0.337158352 1.836053681 1.932192174 -1.308391657 0.250526104 -0.6103395 0.591418534 -0.119434918 1.368195304 0.010499841 0.838839791 0.46675027 0.240079755 -1.550470326 -0.181860284 0.45485194 0.6700433 -0.418638144 -0.46196792 0.23183043 0.1027289 0.601474359 -0.559983648 -0.233899522 -0.704798545 0.143482585 -0.181964409 0.174719777 -0.163498973 0.080392691 0.256193377 -0.111263509 -0.232939009 -0.080805351 -0.250508632 0.187103173 -0.12612447 -0.077793359 -0.323193231 0.280617493 -0.124503041 -0.019515491 -0.141269915 0.312428197 0.158043761 0.204542125 -0.256326322 -0.110264311 -0.095131837 -0.07425044 0.091014988 0.114135217 -0.003975046 0.103929166 -0.119226511 0.011743453 0.140105995 -0.154968122 0.096331531 -0.27951137 0.093950071 0.085844952 -0.147248672 0.103508918 0.070966706 0.189811287 -0.245123627 -0.125808929 0.024985771 0.263842173 0.113791758 -0.065777205 -0.144899953 -0.133239978 0.027844341 -0.079830349 0.043784147 -0.057689599 -0.007530629 0.136463381 -0.119168244 -0.025201534 0.03138808 -0.214109335 -0.147389083 0.091561444 -0.002108508 0.087535522 -0.009032978 -0.062843154 0.004359333 -0.096600507 0.086301951 0.116920175 -0.180298862 -0.142948234 -0.053857165 -0.107562648 0.065396684 -0.155485701 0.130747267 0.012914575 -0.081602577 0.079078471 -0.069466617 0.018625119 -0.034715671 -0.042860283 0.02364105 -0.016774178 0.084485505 -0.012278154 -0.130264255 -0.125344855 0.07941859 -0.087481883 0.013001767 0.045126341 0.051753277 0.045419789 0.059058946 0.108848659 -0.034245516 0.017777924 0.185808103 0.016095754 -0.11855324 0.094112983 -0.204737129 -0.042305544 0.038091151 0.036045979 -0.045620017 -0.131307986 0.030023942 -0.030679345 -0.062964705 -0.09695776 0.061435508 0.007690542 0.121585268 -0.067729799 -0.055126078 -0.072325801 -0.000416091 0.076431868 0.04693236 0.025715225 0.008403477 -0.026923772 -0.033349462 -0.062913777 0.013758047 -0.109723703 -0.039332042 -0.061339754 0.010251498 0.010644056 0.051579753 -0.014397323 0.067920002 0.02846055 -0.058955152 -0.062492111 -0.016824107 -0.044695859 -0.04046131 0.044943264 0.005536329 -0.047499802 0.03878613 0.079270854 0.007561249 -0.009838558 -0.0307133 0.038639211 0.052461811 0.02794937 -0.084668232 -0.074499591 0.007011005 -0.058236218 0.01628155 0.013584233 -0.028333522 -0.003695179 0.023687495 -0.007004263 0.007691508 0.056934305 -0.062287534 -0.051605542 -0.01882569 -0.007088453 -0.021405215 -0.052242716 -0.000946269 -0.015591467 -0.044626878 0.016179539 -0.035777598 -0.026718091 -0.034483361 0.036749786 -0.008184398 0.005847708 -0.098304922 -0.030449387

sample_47 -0.998601999 -3.27770285 -0.591924979 -0.071046427 -1.238484064 -1.153022299 1.501487513 -1.047834748 0.603369239 -0.392992719 0.825253856 -0.7637428 0.399154943 -0.039920266 -0.016267683 -0.363271507 0.613896544 -0.023797026 -0.077188654 0.264487347 -0.361355851 -0.532825842 -0.083762167 -0.352698227 -0.130534478 -0.244022025 -0.280109458 -0.078320898 -0.435011392 -0.053143747 0.007114466 0.079794967 0.295004026 0.022862819 0.111285224 -0.024149068 -0.231506918 0.029614048 0.106695819 -0.056620935 -0.165203113 -0.158387502 -0.078820649 -0.164917749 0.022136539 -0.064755292 0.100153226 0.135624572 0.000792725 0.116480166 0.172558484 -0.334025309 -0.117232805 -0.125918267 0.103857528 -0.079364778 -0.110536084 -0.149589373 0.051001974 -0.266627121 -0.065909688 -0.060773625 0.014754716 -0.124521678 0.052391503 0.042761783 0.030970629 -0.118936238 -0.014497539 -0.033894396 -0.057978952 0.153818398 0.040344129 0.254142796 0.209070838 -0.056526661 -0.022519686 -0.123704746 0.057770506 0.013677843 0.118046903 -0.00366065 -0.020288004 0.039494436 0.033948192 -0.069298215 0.01644779 0.233868903 -0.091111771 -0.065117032 -0.006370252 -0.090736429 0.079438873 0.008127351 0.053825918 -0.125903756 -0.050217127 -0.092731242 -0.068248369 -0.068009509 -0.054139897 -0.062071587 -0.10402964 -0.078927049 -0.070013442 0.022314913 6.26E-05 -0.055213758 0.02048278 0.002637441 -0.010759439 -0.057209116 -0.105062389 0.002295646 -0.085580253 -0.059311081 0.079276832 0.023720597 -0.04927936 -0.058664087 -0.032525313 0.09432283 -0.00362579 0.01474947 -0.032641335 0.008707103 0.044455397 -0.123590895 0.028491696 -0.015934923 -0.031428953 0.061892707 0.02511333 0.031805235 0.034944159 -0.069198211 0.024590638 -0.002968057 -0.119528052 0.15174491 0.011060169 -0.0074616 -0.12223853 0.040660882 -0.018541794 -0.004888499 -0.03500598 -0.096923904 -0.000361158 -0.007442256 0.068949063 0.081675132 0.006613851 -0.041368874 0.003423385 -0.023903052 -0.047816057 -0.136151657 -0.026705278 -0.030689706 -0.099172114 -0.068822243 -0.010556091 -0.00297251 0.008249536 -0.065001174 0.001908759 0.045115039 0.064802749 0.024119934 -0.017720008 0.034335894 0.023625483 -0.028432516 0.060637534 -0.064021224 -0.041598674 -0.011751141 0.002134666 0.032639408 0.048313676 0.023445815 0.001609098 -0.103146537 -0.021862505 -0.078718768 0.00311206 0.059347881 -0.043819802 -0.042981997 0.061307766 -0.017455857 0.009500957 -0.057714889 0.008958538 -0.02694251 0.006294843 -0.048391807 0.085409517 0.030216331 -0.043928319 -0.002934907 -0.016029759 -0.007927197 -0.073319704 -0.013381268 -0.026226538 -0.007854386 0.008732942 0.038750108

sample_48 -2.981060612 0.230488764 -0.414347603 -2.394519835 1.500142751 -1.671570185 0.939015834 0.467368033 -0.466638073 0.955098407 1.066327815 -0.453868118 0.82744414 0.5940054 0.268765938 -0.905490705 0.131620461 0.17483024 -0.563265006 0.022375806 0.02434721 -0.036681793 -0.235241585 -0.455495054 -0.464521511 0.145058261 -0.297759235 0.34285294 -0.385970428 0.299022594 -0.147201115 0.382718625 0.10267439 0.098725428 -0.274243101 -0.167854788 -0.093252961 0.07570294 -0.306717948 -0.193245333 0.340513123 -0.170518298 -0.272030945 0.012964546 -0.096088773 -0.076028499 0.077567418 -0.093122974 -0.168657684 0.216129011 -0.040004181 -0.054584206 -0.0191052 -0.037846074 -0.265327091 0.057180476 -0.025616876 -0.093130787 -0.144595672 0.018506838 -0.175207247 0.142751054 -0.172948692 -0.185347654 0.007399445 0.124971054 -0.091761034 0.028034392 0.098410517 0.187432138 0.000298854 0.019418332 -0.07732573 -0.057460304 0.123645164 0.139561132 0.068424375 0.041977358 -0.029290385 -0.013441616 -0.105857054 0.057832242 0.003826406 0.018790225 0.121253375 0.056349505 -0.09119559 -0.023976272 -0.119361699 -0.020474975 0.122092503 -0.145635075 0.005765529 0.060478759 -0.125600247 -0.061535642 -0.069491484 -0.050769385 -0.045709785 -0.078639711 -0.092102573 -0.113239681 0.122248537 -0.03596877 0.039724438 -0.00100022 0.009939556 0.043538641 -0.185528844 -0.077305575 -0.099695764 -0.022122858 0.028216445 0.045735012 0.064385317 -0.037338592 -0.020047226 -0.070156037 -0.072085791 -0.030817912 -0.055703608 0.085877836 0.025967105 -0.093202253 -0.040446117 0.004364522 0.125955367 0.116887581 0.119363772 0.002767582 -0.069267564 -0.14797639 0.04583137 0.067023053 -0.075315774 -0.00849702 -0.082305253 0.062586324 -0.070933382 -0.022093158 -0.006398966 0.050405139 0.033152775 -0.06204269 -0.031914475 -0.0620861 0.077973446 -0.04024401 -0.086713849 0.087316533 -0.033686206 0.087297665 0.016238429 0.040325777 -0.02465174 0.01104218 0.024528408 -0.022074374 -0.025046998 0.081054592 -0.020270418 -0.003568063 0.027915672 0.037848536 0.062597478 0.025645352 0.034985864 0.007268769 -0.106740393 -0.042063082 -0.044840257 -0.003310401 -0.017365216 -0.076010292 -0.021460839 0.010898934 0.004455929 0.027788809 -0.01750981 0.009390288 -0.038233515 0.037677529 -0.033369628 0.068352682 -0.028120447 -0.031285756 -0.064027855 -0.062506084 -0.062940123 -0.040311153 -0.018744253 0.080253055 -0.025236235 -0.007012409 -0.040553905 -0.036130714 -0.039003581 -0.024616305 -0.033287561 -0.072588706 0.003835243 0.021491408 -0.055846061 -0.040592585 0.03534803 0.012419927 -0.005626783 -0.023515057 0.031471167 -0.045792538

sample_49 -2.409991303 -1.731612408 1.400767981 0.017864479 1.350831926 0.249965699 1.163577917 0.161998414 -1.460878145 -0.383447696 1.387481121 -0.121667515 0.414255976 0.285326449 -0.076625652 0.248981573 -0.284869475 0.461192576 -0.040458647 -0.385584448 0.086772801 -0.183911472 0.174943957 -0.509190303 -0.10310558 0.197536617 -0.095509016 0.34867676 -0.403652722 0.167111141 -0.434756643 0.06002153 -0.480597375 -0.137041859 -0.100572175 -0.239799356 0.069947753 0.076134317 -0.039409426 -0.095546817 -0.070055659 -0.246968605 -0.020805822 0.316169145 0.041792819 -0.095875264 0.423325226 -0.003679736 0.056924253 -0.047070945 0.175515804 -0.231612546 -0.040653209 -0.065576354 0.147860867 0.133673753 0.082106196 -0.057655917 -0.111993204 0.048601581 0.069922049 -0.244349795 -0.097777816 -0.017472484 -0.108215706 0.048102376 -0.130156895 0.077876357 0.104505337 -0.05121682 -0.023227418 0.097507986 0.062663334 0.058322757 0.067473523 -0.000410819 -0.086974515 -0.009065606 -0.039541141 -0.056133147 0.019751849 -0.068414906 -0.086926774 0.118573628 0.22960204 0.082257199 -0.068878587 0.084004981 0.057486091 -0.104568995 -0.212289219 0.029012192 -0.036660285 -0.056303727 0.08829944 0.041552895 0.022086064 -0.01747986 -0.007086396 -0.046379295 0.143114233 -0.035129552 0.181094138 -0.146608031 -0.056815649 0.11201621 0.079862034 0.138962751 -0.086449216 -0.007766186 0.055784414 -0.080850491 0.047133198 -0.006467385 0.008110171 0.004040573 0.029471016 -0.032469169 -0.021641567 0.125729595 -0.068793451 0.040462563 0.050562266 0.046058415 -0.049643112 -0.003963669 0.054841325 0.110644575 0.033762418 0.067306187 0.077553081 -0.041054196 -0.104516955 0.036189568 -0.012541472 -0.024935142 0.042224837 -0.010404443 0.119748691 -0.049401123 0.018937019 0.018948399 0.038594435 -0.028246822 -0.082707919 0.023918974 0.066472399 -0.041556758 0.098007611 0.073003622 -0.012034546 -0.005382932 0.039325623 0.004350259 -0.075367713 -0.054200638 0.006890772 0.006231349 0.068049273 0.043140888 -0.020407736 -0.02876169 -0.01083221 -0.043443572 -0.008729577 0.000678058 -0.01513818 -0.003931247 0.058572057 0.035397375 0.003282614 0.080012454 -0.018666034 -0.058546616 -0.009941846 -0.051577853 -0.040403279 -0.007337822 0.020944684 -0.065557187 0.00749238 0.021004102 0.009847021 0.021595135 -0.070875458 0.008578564 -0.080248435 -0.012281677 -0.035410114 -0.079700587 -0.041786931 -0.009769211 0.019490337 0.035533926 -0.007611912 -0.038826695 0.009372326 0.030410009 0.010747115 -0.018220816 0.032227928 0.048991889 -0.053387209 0.017150703 -0.003242113 -0.004528706 0.002682212 0.012721286 0.00254078 0.00221178

sample_50 -3.968687509 -0.558399406 -0.204678175 -2.599755258 1.663447922 -0.550512089 0.721538781 -0.023950757 -1.684962189 0.593870788 1.044567875 -0.567185054 0.019024868 0.577389512 0.202568645 -0.055689108 0.088438363 0.082786502 -0.414777279 0.032718996 0.631693904 0.205317965 -0.177756497 -0.118958525 -0.127941583 0.23032131 0.084950686 -0.436506021 -0.251588441 0.273551148 0.037302504 0.330639992 -0.17403372 -0.118783612 0.021622215 -0.432076783 -0.250872819 0.039013831 0.064575554 -0.158385777 0.166230214 -0.075038603 -0.034370812 -0.049911624 0.003815549 -0.273866161 -0.018879181 -0.188107119 0.163666893 0.207871313 -0.084747003 -0.082901148 0.081180741 0.334187788 -0.17668744 -0.031221983 0.015088991 0.08703631 -0.17728487 -0.117757034 -0.026603571 0.066120589 -0.184819049 0.01235417 0.037745258 0.111725205 -0.160179453 -0.054242317 0.270316772 -0.039141257 -0.09513731 0.064005893 -0.123385864 -0.105707885 0.134530088 0.030713536 0.010812358 0.076998064 0.036275073 0.05504509 -0.007798142 0.15467181 0.106801182 -0.165402135 0.048064832 -0.073112022 -0.013665103 0.016383084 -0.080788618 0.065300477 -0.003765147 0.065761502 0.004090683 -0.028873405 -0.145902839 -0.140141643 0.031407366 0.040022262 -0.002715556 0.043273221 0.058591119 0.056111445 0.137662892 -0.038661315 0.071090296 0.021025702 0.000678239 0.131961914 -0.1115775 0.074675866 0.038928258 -0.027027768 0.09304656 0.09262014 0.045884542 -0.020001836 -0.022544938 0.047833377 0.055664615 0.122339049 -0.058429686 0.09873239 -0.015767634 -0.024990802 0.023552072 -0.018983656 0.037672144 0.071696253 0.109784602 0.056180062 0.041567507 -0.062158372 -0.124221374 0.024670887 -0.072140121 -0.007740081 -0.065045357 0.115159462 -0.032030032 -0.014556042 -0.040997139 0.020105735 0.036875082 0.004772405 0.042123349 -0.010486473 0.059309547 -0.057741042 -0.101232008 0.03619014 -0.073871152 0.099966835 0.08081531 -0.049853724 0.020222957 -0.006429421 0.014592572 -0.006468553 0.052036854 0.097391417 -0.002871849 0.020719866 0.01453646 -0.025351621 0.010986575 0.067818133 -0.015401151 0.020479353 0.023521016 0.024378265 -0.044840453 0.004666018 -0.075595245 -0.00816618 -0.030793315 -0.057092462 -0.045965336 0.063021805 0.019090318 -0.057051316 -0.014192198 0.004429251 -0.02179325 0.022218084 -0.032839642 0.025821838 -0.066056437 -0.03286344 0.02944359 -0.02962283 -0.09941771 0.059998007 0.022621091 0.049559824 -0.009173506 -0.002909554 0.0055073 0.005750217 -0.020436234 0.01056254 -0.016858328 -0.023842353 -0.004191431 -0.010370007 -0.003720567 0.030314714 0.052856227 -0.009748975 0.024708737 -0.058716304

sample_51 -6.847114086 -0.22098698 1.155222778 3.520808089 0.748981366 0.096016772 -1.319911417 -0.185902964 -0.003944429 -0.401551346 0.334262884 0.01440321 0.324684788 -0.276417755 -0.288406409 0.329909664 0.277688598 -0.022425704 0.233826198 -0.254050099 -0.120528893 -0.054402803 -0.295698754 0.487735116 0.688691222 0.039381232 -0.190057244 0.047916095 0.191068857 0.007912321 0.101411465 -0.20772032 -0.053008777 -0.394809623 0.011259144 -0.237382543 -0.510667976 -0.305287389 0.102716218 -0.068975162 0.066568395 -0.123456553 0.027030562 0.276883004 -0.232643011 -0.163375846 -0.340829871 0.334946998 0.237905701 0.157866752 0.164088154 0.009461135 0.038373424 0.047661927 -0.199871609 -0.120893951 0.01862768 0.048368836 -0.065049887 0.105313291 0.089434891 0.061228704 0.066651037 -0.079970844 0.039582776 -0.188029435 0.087074725 0.058636457 -0.047683339 -0.16293469 -0.032254543 0.094003296 0.094102067 0.097781089 -0.14521182 -0.319614284 -0.197883922 -0.030103701 0.071434679 -0.117907401 0.121682872 -0.221183118 0.137953199 -0.055125604 -0.046119907 -0.062025126 0.003231199 0.056063643 -0.16336649 0.2055312 0.037819496 -0.222486307 -0.133575875 -0.088128929 0.105516711 0.09296502 0.015900653 0.008493241 -0.032968118 -0.026078343 0.013280124 -0.051124958 -0.097999043 -0.134568822 -0.062344278 -0.048394401 -0.021956201 0.070666617 0.01020271 0.095713084 -0.103818219 0.199798214 0.051064044 0.050801717 -0.038407835 -0.039773029 0.032257752 -0.026017067 -0.006829235 0.024440362 0.225251979 -0.045155154 -0.098301556 -0.029371026 0.063191831 -0.088324762 0.098340819 -0.025016412 -0.152836422 -0.045886382 -0.029377534 -0.073386588 0.011176475 -0.020435037 -0.044807539 -0.030570263 -0.07887901 -0.003011584 0.09094703 -0.106611578 0.063171824 0.014309863 -0.003859543 0.033850031 -0.072288443 -0.00698358 0.132868822 0.074967195 -0.078079565 -0.13030828 0.105693846 -0.10929744 -0.040184711 0.007958895 0.008359048 0.067321617 0.063183554 -0.101259177 0.027170836 -0.04471573 0.056233453 0.053827788 -0.021739552 -0.017550874 0.077395243 -0.072840709 0.104536687 0.017992287 0.040665879 0.025207277 0.031533619 0.030426064 -0.015007046 -0.004140422 -0.030023671 -0.024485066 0.023537554 0.098291777 -0.046826095 -0.021507009 0.039788129 -0.028195659 -0.069568556 -0.015696002 -0.070508054 -0.034648696 -0.032284438 0.085381378 0.048128518 0.080785953 0.026033999 -0.013131289 0.016874607 0.042737346 0.0173289 -0.078300246 -0.006819787 0.028378451 -0.018338099 -0.03301199 0.034762316 -0.003917546 -0.015189836 0.002005836 -0.014587506 0.019086651 -0.042098339 0.088207197 -0.027232004 0.036571946

sample_52 -0.809789941 -1.807757259 -2.390336594 -1.714913221 -2.251597528 0.772171311 -1.171314088 1.720911282 0.478337932 0.532554436 -0.202141254 -0.15892371 -0.973409046 0.334056015 0.247654876 -0.374838764 0.691642088 -0.074415235 0.155908624 0.15314667 -0.193449741 -0.088275631 -0.551534691 0.025787101 -0.392383446 -0.220595422 0.09013923 0.381714349 0.04055434 0.139935965 -0.944040185 -0.296005654 -0.29405115 -0.055169619 -0.598628372 0.218661498 -0.04743652 -0.654214931 -0.262272435 -0.10120865 -0.107190663 -0.195301749 0.053865604 0.124593104 -0.122800881 -0.408101723 -0.430067306 -0.065590899 -0.278451943 -0.129473467 -0.117527891 0.077526474 -0.18995486 0.003254813 0.227115155 0.163896457 -0.035679438 0.002064974 0.073843099 -0.103319024 -0.002634593 0.009300968 0.051017107 -0.036760874 -0.28051178 -0.137484221 -0.139667341 0.07487348 -0.008165393 0.103630921 0.05854117 -0.000735354 -0.022558308 0.219898146 -0.015579816 0.079235924 0.185959499 -0.116312693 -0.129043293 -0.065550185 0.188003607 0.243454549 0.066248377 0.175850796 0.109858525 -0.067970307 0.005771838 -0.022152425 -0.056818273 0.082731562 0.038091094 -0.053586832 -0.051027885 -0.012446427 -0.091679003 0.136504277 0.032083747 0.060436155 -0.00728584 -0.053724536 -0.123301202 0.001166166 -0.061960359 -0.034732929 0.18040704 -0.031884938 -0.178490197 -0.01008893 0.178071147 0.05824317 -0.130171646 -0.185154013 -0.105831397 -0.145673644 0.029017567 -0.118435866 0.095456027 0.044890701 0.1485594 -0.033085511 -0.099502556 -0.10380697 0.250946603 0.033283472 -0.088946444 0.037316918 0.062175671 -0.088829519 0.077895604 -0.023118609 0.02898775 -0.061846749 -0.022675664 0.018102538 -0.061572623 0.0715364 -0.002596011 -0.064721506 0.067093598 -0.326388369 0.054447574 -0.000755818 0.074950639 0.063286675 -0.006697507 0.098783102 -0.032043942 -0.043239701 0.250233927 0.026113265 0.045413788 0.036420389 -0.073339964 0.109286745 0.101237588 -0.058240652 -0.031702258 0.023646532 0.062953522 0.020237313 0.164364835 0.039772653 -0.113603299 -0.102305098 -0.045430568 -0.111248686 -0.077110665 -0.055326068 -0.044912576 -0.019389155 -0.032406833 0.045150848 -0.090143896 0.015027985 0.006272284 0.054393954 0.081396016 0.033966827 0.03031306 0.05693102 -0.080237761 0.066055929 0.069285366 0.019564466 -0.05502788 0.152091189 -0.069503543 -0.057308642 -0.010408502 -0.049056114 -0.020161525 -0.067128042 0.033923376 0.012172018 0.059203182 0.020645317 0.021391211 -0.023478424 -0.063337639 0.018566 0.058341227 -0.013976971 0.034910167 -0.016759274 0.04232468 -0.036556978 0.005599592 0.002594265 0.011143232 -0.027456267

sample_53 -1.32784229 -4.457310717 -0.594085372 1.607744964 -2.864591611 0.269728505 1.19043797 -0.161962648 0.38961001 -0.694422236 0.414364693 0.016540483 0.782844517 0.123964831 0.130833205 -0.221434306 -0.316370254 -0.67337921 -0.177606399 -0.190138067 0.070054769 0.237002812 -0.124562726 0.10981468 0.405768877 -0.174484189 -0.2265142 -0.302480861 0.416097123 0.326343698 0.253461415 -0.082267069 -0.048487779 0.29639217 0.251879936 -0.174582316 -0.19663883 -0.025890066 -0.08772236 -0.017010556 0.150763736 0.003927198 -0.123269579 0.161927128 -0.200944944 -0.061132973 0.069909518 0.161863563 0.089945133 0.226817489 0.078923777 -0.09685914 -0.212963838 -0.278043921 -0.063654205 0.054175274 -0.118375781 -0.16969896 -0.110188745 -0.130725076 -0.044611709 0.020788468 0.158752557 0.166403468 -0.077822036 -0.062649054 -0.042245145 -0.042529054 0.10228947 0.030459123 0.0945337 0.162415422 -0.106881663 0.067292379 0.003874838 0.043706961 -0.098472935 -0.149215058 0.022310992 0.00608222 0.020096793 -0.078110872 -0.011744385 0.12000851 0.073272747 -0.189663003 -0.010606085 0.115355533 0.076014179 -0.125000564 -0.08563353 -0.082986699 0.092851744 -0.076483178 -0.039860576 0.007092583 -0.075377291 -0.074529683 -0.0506706 -0.046116167 -0.064487237 -0.006488925 0.014213899 -0.000715345 0.133449192 0.039307967 0.041975402 -0.067957424 -0.112604264 0.082644384 -0.049858385 -0.01653707 0.040059609 -0.035455792 0.007780639 -0.064276801 0.037667903 0.022656469 0.013077895 0.015831831 0.022275857 0.05050966 0.021956847 0.031707056 0.009296288 -0.030590637 -0.082302357 -0.124391222 0.018498172 0.047708994 0.023126922 0.009175952 -0.012415479 0.100072866 -0.027712285 -0.010311224 0.028959799 -0.030679427 -0.03280441 -0.01059736 -0.106639376 0.029516979 -0.091743429 -0.065037062 -0.057108501 0.022527649 0.010137122 -0.008828469 0.016056122 0.015155713 -0.022482895 0.041994266 -0.105413322 -0.024249849 -0.019202697 -0.028456405 -0.059904234 -0.024684447 -0.08717515 -0.041436289 0.006532529 0.008392929 0.010859624 0.049987266 0.015297304 -0.014280663 -0.059136452 -0.031850845 0.023276103 -0.00320568 0.027351146 0.007402258 -0.010203278 0.010133639 0.024018657 -0.012938804 -0.024863183 -0.014078465 -0.026942825 -0.009931276 -0.00052778 -0.009202926 -0.003943383 0.003967869 0.062075165 -0.077734576 0.0352237 0.02766951 0.025796745 0.048450075 0.00507582 0.014469395 0.043783337 -0.07007116 0.052037901 -0.003220377 5.87E-06 0.004248874 0.097980205 -0.023045527 0.010586377 0.008552088 -0.008176278 -0.019976461 0.008632915 -0.012206696 -0.023539965 -0.004779371 0.056702916 0.043027348

sample_54 -5.043250665 -1.672192407 -0.105369994 0.038296853 -0.910308583 0.711718198 -0.995466554 1.236851955 -0.464587131 -0.391422739 -0.205935887 -0.504157207 -0.940854237 0.271224547 0.997077798 0.773813549 0.957916307 -0.292370692 -0.840395673 -0.211320868 0.235016395 0.012741826 -0.313361705 -0.157960382 -0.379301957 -0.279351105 0.239548427 -0.072865931 0.008112153 0.141573438 -0.024231171 -0.145305941 -0.523544929 0.050884073 0.074816318 -0.514137603 0.212342495 -0.004106152 -0.027826179 -0.080822108 0.037857555 0.21368717 0.097170011 -0.283700078 -0.018662237 -0.186567793 -0.170191624 -0.140062256 -0.103719512 -0.046886362 -0.036578831 0.081351113 -0.021166218 0.143771971 -0.114337505 -0.28566235 -0.149249115 -0.027624339 0.250897895 -0.156355427 0.003447896 0.017568829 0.243021688 -0.011205526 -0.143363252 -0.104140051 0.019840994 -0.155926511 0.153892042 0.186773438 0.046405721 -0.124866776 0.090831439 0.254183337 0.014388303 0.017598833 0.037687796 -0.015698901 -0.077624364 0.029259174 -0.141390138 -0.018901045 0.005313283 -0.187197921 -0.091284221 0.060499078 0.055899496 0.079770267 -0.028448469 -0.18627063 -0.042804963 -0.112913269 0.017062224 -0.02873923 0.071147487 0.126116876 -0.024304303 0.03711167 -0.146262394 0.056845981 0.052089532 0.09153496 -0.123614864 0.102649626 -0.026457046 -0.100750391 -0.034164538 0.018610455 0.052826979 0.103142716 0.010859656 -0.085217863 -0.0145955 0.233021512 0.0686453 0.017873759 0.071959361 -0.049369759 -0.127142754 -0.050175606 -0.042271034 -0.085232263 0.087633318 0.031869461 -0.00689942 0.118082198 0.042404155 -0.091347362 0.081620458 -0.04788496 0.053921404 -0.049331176 -0.002340705 -0.070268198 -0.000404927 -0.016394118 0.029053381 -0.010422912 -0.017350144 -0.112466238 -0.074096688 0.024406956 0.031787751 0.01544813 -0.005722276 0.01484016 -0.105105272 -0.068065366 0.107526554 0.052099727 -0.042349738 -0.012348878 0.029611637 -0.001689933 0.062185368 0.01678334 -0.058897458 -0.044837807 0.061569623 0.01612266 0.063089686 0.001554704 -0.059461492 0.044434781 0.011951704 -0.046230907 -0.015071997 0.023049957 0.045746803 0.012156282 0.099277125 -0.008704845 -0.013351233 -0.07831774 -0.041967017 0.04484653 0.049936535 0.050198383 0.017328466 -0.032282525 0.079372971 -0.061716441 0.044035093 -0.004100392 0.054910366 -0.028190559 0.030697855 -0.009628976 0.038432073 -0.041501423 -0.040298313 -0.014333948 0.099878677 -0.019828825 -0.024703794 0.023889974 -0.012352643 -0.071656417 0.035241753 0.002752733 0.013664404 0.077369661 -0.027684498 -0.048190649 0.003188735 0.014841396 -0.037687501 0.049479931 -0.037188703 0.032355271

sample_55 -0.11865483 -3.394008882 -1.06320115 -2.199691497 -3.850711678 -1.407302552 -0.420050939 -0.734488129 0.732280096 1.52057333 0.067297116 0.121171355 -1.473498183 -0.422600365 -0.174680076 -0.005136171 1.080616166 -0.226005866 0.679358344 -0.881317101 -0.027938467 0.164647553 -0.075020257 -0.178026057 -0.456095679 0.682109083 0.190570829 0.097192756 -0.176308868 -0.262070021 -0.767111801 -0.030987509 -0.069601026 -0.634383848 -0.006193268 0.039990449 -0.149747376 0.236649564 -0.012215896 0.601721302 -0.008207014 -0.007268692 0.385553629 0.066245284 -0.396561603 -0.14202701 -0.002123504 -0.011755184 0.044613695 0.001298784 -0.36028946 -0.03874917 0.26786134 0.101070989 0.322736145 -0.053817471 0.059607361 -0.137174882 0.227335407 0.155719888 -0.236995335 0.104536828 -0.321069994 -0.012369811 -0.356773169 -0.188413483 -0.243722302 -0.110691209 -0.445663356 -0.121740778 0.018281455 0.041639264 0.054153574 0.076232953 -0.144381397 0.007445138 0.033154045 0.031603413 0.074647767 -0.012324761 0.028328654 0.066047613 0.139379065 0.126927708 0.045873575 0.080419353 -0.036712773 -0.029692475 -0.0218437 0.144216083 0.139992836 0.071766898 -0.116466141 0.103300268 -0.042013387 0.182319605 -0.046894536 0.198946438 -0.016263355 -0.001170063 -0.196200983 -0.138478198 0.013343524 -0.048438951 -0.036478559 -0.01720096 -0.162363431 -0.002433686 -0.025415202 0.053745482 -0.177331765 -0.106497938 -0.124637238 -0.012102262 -0.062920151 -0.032742058 -0.156928267 -0.025630934 -0.025396668 0.000804747 0.023513666 -0.15324378 0.080430947 -0.053400102 -0.04500487 0.077576061 -0.004383849 0.018056977 0.019633195 0.096757045 -0.018342084 -0.084436482 0.100438145 0.070643023 -0.007035467 -0.028903191 -0.056123064 -0.07404092 0.114560104 -0.06985833 0.027870089 0.019603288 0.001088806 0.01460232 -0.000385005 0.051990415 0.10063263 -0.035653871 0.088872497 -0.050714446 -0.018044349 -0.118752764 -0.029574076 0.052278546 -0.017366561 0.055109825 0.055440942 0.083839348 -0.121951098 -0.037731795 0.060288847 0.080601432 0.023917572 0.051079825 -0.006411538 -0.0636849 -0.042109008 0.053493685 -0.136976948 0.041221359 0.100830136 -0.037235068 -0.079501476 0.12984836 0.083405638 0.059427205 0.086583326 0.072365942 -0.007429282 0.01930685 -0.053144826 0.005248578 0.022344834 0.026499791 0.023505793 -0.021742023 0.058838462 -0.015365386 -0.017740156 0.04326229 -0.025412195 0.012956095 0.038600123 -0.056043869 0.058212164 -0.020593902 0.075578016 -0.07587811 0.035144849 0.09104314 0.029962247 -0.090961286 -0.01264888 -0.003981481 0.037714981 -0.003180457 0.012065679 -0.045284126 0.006958501 0.025043892

sample_56 -3.436602885 -0.641947277 -0.279170046 -3.019650739 -0.057357158 0.608063643 -0.805974414 1.328598476 0.205467378 1.191226452 -0.411584211 0.378580827 -1.156795843 0.085322643 0.780026034 0.551039264 1.037846012 0.081704596 0.157433149 -0.394229484 0.421346814 -0.022942777 -0.307520184 0.177606175 -0.54056484 -0.035933012 0.568970054 0.184319751 0.069663826 -0.256726231 -0.295401765 0.168884844 -0.203361943 0.232992418 -0.362249094 -0.004453673 0.533073138 -0.376460818 0.096273714 0.019178145 0.211377446 0.244732411 0.576348963 -0.421897056 -0.085180446 -0.484211057 0.111732691 0.054055388 -0.047190245 -0.364096139 -0.048337384 -0.03095246 -0.077245031 -0.092864075 0.161068809 0.01582559 0.099019205 -0.033926892 0.283597376 -0.009434788 0.116495082 0.31701144 -0.046228656 0.284394575 -0.304009364 -0.126897516 -0.030074522 -0.204208988 0.050197322 0.183999028 0.15855873 -0.219400118 0.069599963 0.063090136 0.046715512 0.046872525 0.025017955 0.090599645 0.009684061 -0.01918148 0.122298066 -0.073006324 0.008130216 -0.061851509 0.128310669 0.104411936 0.014920144 0.057890614 0.086533891 -0.153682583 0.036058155 0.015012659 -0.003145365 0.145150934 0.195677671 0.023335478 -0.07479045 0.111137027 -0.141280913 0.123140143 -0.007277644 -0.188930658 -0.112662833 -0.064125532 0.003450114 -0.219034394 -0.049125202 0.134733716 -0.147562548 0.109183909 -0.076080925 -0.110347264 -0.214207541 0.0144846 0.193190072 0.032922522 0.08316047 0.138539477 0.114627057 -0.056676362 -0.073576316 0.039234445 0.289979638 0.076481679 -0.015072674 0.178890461 -0.042407532 -0.019678352 0.031588037 0.01469858 -0.044605038 0.091949188 -0.01792921 -0.053034118 -0.136768425 -0.066218823 -0.04182681 -0.084919716 -0.146473743 -0.151689042 -0.10862237 0.053462093 0.109457992 0.094152129 0.003516818 -0.027547921 0.060528076 0.066246394 0.103668325 -0.134702458 -0.011408735 -0.084901361 -0.0964289 0.069668589 0.123741357 0.053771338 0.016705958 0.096409216 -0.037709759 -0.04064397 0.140403237 0.104166637 -0.003137197 -0.059334997 -0.057770475 0.009446621 -0.112161255 0.081348468 -0.076956812 -0.041937602 0.163284497 -0.060980353 -0.034747373 0.157959037 -0.083842599 0.012273927 0.125404625 0.089561892 0.029468683 -0.036194598 0.014792332 -0.063884834 -0.059926308 -0.049713503 0.028684509 -0.007173318 -0.067905815 -0.047305502 -0.003599735 -0.042803146 -0.047605892 -0.003301671 -0.045433667 0.03960044 0.005759264 0.055056912 0.008142238 -0.056492032 0.020958914 0.053308577 0.001491875 0.143882011 -0.006564441 0.020459468 -0.046971236 0.027368632 0.016465411 -0.019223281 -0.037337462 0.091779242

sample_57 1.873954134 -0.230010223 4.130770942 -1.95228044 -0.037756413 -0.246388403 -0.332254598 0.038509483 -0.759086089 1.473958793 -0.835224857 -0.643018391 -0.979222374 -0.025332079 0.478474972 0.310069242 -0.262278584 0.474359727 -0.230757685 0.072814742 -0.255155536 -0.180580133 0.192930454 -0.244612659 -0.116385417 0.519865675 0.370187177 -0.22824045 0.138379132 0.177438388 -0.153999422 -0.029776704 0.013766973 0.079992674 -0.13289558 0.244167834 0.024323202 -0.606968548 0.227773821 0.193546074 -0.212541045 -0.111697443 -0.247186716 -0.168899337 0.194252513 0.374828141 -0.208355337 0.48801997 -0.038051089 0.251726315 0.033986302 -0.219762891 -0.037521457 0.024744168 0.041138635 0.165338595 0.156767611 -0.210017962 0.177939664 0.221962972 0.10094187 -0.026092721 0.173361505 0.122111116 -0.129585078 0.071166635 -0.323157101 0.214793328 0.097369763 0.132103128 -0.011119719 0.136496415 0.034847471 0.023084115 -0.027433109 -0.073033185 -0.199152079 -0.029292599 0.016536178 0.018149205 -0.03437517 -0.016795809 -0.000154613 0.203267537 -0.171629309 0.085197729 -0.120708149 0.118891885 -0.109231217 -0.113760787 -0.095273428 -0.122385431 0.139371733 -0.057064511 0.103418689 0.062856625 -0.123613339 0.100030509 0.084256349 -0.174214146 -0.032174264 0.027866325 0.045340841 -0.0183709 0.185528997 -0.020169124 -0.013275538 -0.03613289 -0.02434743 -0.097793161 0.103584703 -0.106810839 0.059796783 0.106517449 0.063873498 0.022278754 0.083322122 0.072410879 -0.042961858 -0.02986939 -0.027246599 -0.172621248 -0.066234428 -0.005182218 0.015357017 -0.037640772 -0.008719243 0.020677043 0.015338374 -0.127346361 0.010299826 0.011924044 -0.034064795 0.12231067 -0.016175818 0.022699516 0.059901589 -0.021081675 0.026340562 -0.003821017 0.01500044 0.045061014 0.019700874 -0.112844656 -0.00776432 -0.077201857 0.093654309 -0.047543379 0.063668533 -0.031794597 -0.019173543 0.030313772 0.02556668 0.025505106 -0.06371483 0.007075788 -0.015152778 -0.025613235 0.066106537 0.083929532 0.005050305 -0.060741086 0.113269194 0.016055111 0.077732205 0.035861913 -0.010953355 0.065348025 -0.039014181 0.029780956 0.11136893 -0.056871417 0.0685638 0.021189972 -4.44E-05 0.045521412 0.015197526 -0.016163869 0.072146545 0.031833553 -0.087220737 -0.102880868 -0.02333884 0.036131032 0.017347801 -0.016737888 -0.01404903 0.042931796 0.025025039 0.020805989 0.005609419 -0.005454702 0.066129876 -0.0228714 0.052990476 -0.077886849 0.099090751 -0.004590046 0.072279986 0.028610184 -0.011080309 0.089410066 -0.003981533 0.003881431 0.033626966 -0.027354802 0.044647027 0.009827645 -0.013757325 -0.009427612

sample_58 -1.619291564 -2.435112397 3.474277039 -0.488120485 1.611164813 -0.475067576 0.599506295 -0.029320453 -1.090577679 -0.724616493 1.353686292 -0.265770346 0.41406713 0.100766238 -0.641466711 0.04050885 0.158973519 0.160969485 -0.612868032 -0.408088504 0.317743842 -0.006074698 0.605064285 0.051533146 -0.172709628 -0.032322338 -0.184999463 0.032402764 -0.256112715 -0.05566427 -0.1341896 -0.316251359 -0.172353429 -0.133028591 -0.350232289 -0.048085731 -0.294441391 -0.193327082 -0.195798851 -0.2720302 0.182228888 -0.153918867 0.000428874 -0.022849113 0.138077508 -0.036862142 0.27466301 -0.047961278 -0.03278418 -0.126033597 -0.010547838 -0.117446275 0.0430764 0.082748077 0.107667715 0.202182877 -0.21016121 -0.007923052 -0.352478042 0.04564015 -0.165222168 -0.143535162 0.081817999 0.068856202 0.061353064 0.054868428 -0.149362132 0.042953785 0.121580772 0.102816156 0.015435961 0.075919693 -0.037669151 -0.077795911 0.037417466 0.07547309 -0.054560115 0.172316011 -0.011079732 0.053024927 0.032596079 -0.060834764 -0.069263437 -0.037811548 -0.060721192 0.018786612 -0.007852635 0.10485609 0.058756641 0.014500516 -0.128534658 0.002257252 -0.003987854 0.068616687 -0.059529792 0.017479215 0.127490551 0.012694109 0.063902202 -0.011850982 0.169047802 -0.067685823 0.162431263 -0.104410009 0.049164719 -0.040621036 0.021476656 0.039040998 -0.019139915 -0.027929317 0.006530248 -0.016075883 0.153529274 -0.038469517 -0.073556205 -0.004616089 0.018359446 -0.141651862 -0.051710931 0.001347029 -0.01046225 0.053276122 0.010742758 -0.083962706 -0.031729063 -0.051517597 0.017409902 0.09149565 0.0532213 0.1242662 0.092069189 -0.028458258 -0.015822805 -0.095215892 -0.068396248 0.053360564 -0.004942573 0.066290456 0.04572346 0.000559223 -0.001114756 0.06253583 0.011935965 0.046398584 -0.053706668 0.063072624 0.045020366 -0.089893075 -0.000715682 0.045743533 -0.049545648 0.011051397 0.0311952 -0.034119516 -0.017835816 -0.019213432 0.074774271 0.056878518 0.050879384 0.062604361 0.018706346 0.034896903 0.033000137 0.033735142 -0.027837011 0.017332138 0.046576199 -0.03668597 -0.055582453 0.043661914 -0.038600033 0.04524324 -0.069118269 0.022632068 -0.007945069 -0.076663858 -0.051952007 -0.011966488 0.119315473 -0.052796824 0.04907396 0.053534794 -0.006986542 0.066470745 -0.02105929 0.005537836 -0.032736807 -0.06048533 0.015282778 0.000428812 -0.052093961 0.022766291 -0.020716365 0.050096251 -0.025691276 -0.005024248 0.016367743 0.048447653 -0.030983644 -0.002764044 -0.014075253 -0.054438455 0.024090853 0.075400429 -0.000790221 -0.03265148 0.047927761 0.042487714 -0.046797358 -0.020401143

sample_59 -5.317623182 -0.632941848 2.325618639 3.934172264 0.933358801 0.128141839 -1.12772799 -0.234339467 0.084191186 -0.762184544 0.262587761 -0.994033985 0.612811544 -0.566807717 -0.484509815 0.122489123 0.147577759 0.349203794 -0.112080685 -0.417330089 0.134177488 -0.025874075 -0.156598066 0.602534717 0.002139578 -0.125987936 -0.910391837 -0.213943263 0.009054763 0.103645174 0.179577762 -0.235080045 0.146682151 -0.438244014 -0.091126263 0.046568569 -0.584391015 -0.119263808 -0.361723663 -0.126615011 -0.083490031 -0.192381301 0.000269256 0.288921262 -0.255234098 -0.038025119 -0.00790232 0.190296774 0.301952649 0.170117344 0.128619868 -0.025966967 0.224936792 -0.126501028 -0.109215512 0.245994794 0.031701076 -0.130503545 -0.061785975 0.152520281 -0.030555811 -0.155397914 -0.165142483 -0.044717094 -0.071142361 0.000482941 -0.10013692 -0.019220263 0.136140456 -0.004319712 -0.206123023 0.152901235 0.044827418 0.098536133 -0.04213404 -0.127315404 -0.077725115 -0.069384917 -0.041577457 0.172604984 -0.050052341 -0.085089948 0.068754621 0.062084827 0.028752809 -0.009620159 -0.148914097 0.149138568 0.009597027 0.206875629 -0.082225757 -0.045628556 -0.135871022 -0.068586808 0.132876584 0.066032689 -0.001258051 0.010090018 0.075641341 0.120327799 -0.00181683 -0.05755402 -0.141004216 -0.036444473 0.057869238 -0.087886212 -0.003525384 0.150679324 -0.09443754 0.029435099 -0.10213014 0.022762093 0.051052577 -0.105701169 -0.0272271 -0.073749737 0.053884555 -0.021276984 0.124399485 0.099445123 0.165278098 0.093416221 0.018058417 -0.009574151 0.017377491 -0.080525176 -0.001339258 -0.046768361 -0.122155387 -0.044820331 -0.101713371 -0.025856884 -0.005789694 -0.051730216 -0.035726899 -0.036087456 -0.06802978 0.007101285 0.037316054 -0.064245396 0.021543933 0.04475026 0.041586475 -0.080066231 -0.02684714 0.016961595 0.065624666 0.077318787 0.03002528 -0.035722815 0.001775816 0.045978902 0.014768768 0.091196052 0.050114376 -0.029665357 0.075936527 -0.013957242 -0.008990401 0.003803255 0.126738868 0.082944174 -0.074646445 -0.004866915 0.023960005 -0.019494373 0.067360779 -0.0003574 -0.069045208 -0.012967877 -0.029036572 0.000912481 -0.05197673 -0.049688114 0.045264929 -0.078110296 0.026224442 -0.0175032 -0.064018729 -0.057849808 0.013780551 -0.034425565 -0.015518091 -0.029953483 -0.001616363 -0.038307892 -0.030896054 -0.023673809 0.05492349 0.040667149 0.021222255 -0.01699884 0.031355596 0.037786859 -0.022898912 -0.013687122 -0.049734397 0.095242782 0.023879604 -0.034828123 0.026716046 -0.034738766 -0.05327371 0.03938081 0.005449303 -0.046168776 0.005045178 0.094233101 0.019626116 0.097847819

sample_60 2.451623289 3.338692487 1.698184472 -0.36357122 -0.018146999 -3.207681773 -0.024667907 -0.428578433 -0.461255775 -1.152623881 1.897521983 -0.038183538 0.498010857 0.043691946 -0.301539152 -0.134805607 -0.006189496 0.910294753 0.348415461 0.111593648 -1.086394986 -0.049815264 0.226656494 0.104146877 0.169071511 -0.446099259 -0.139278079 -0.523303868 0.325615923 -0.155390818 0.443376935 0.438733995 -0.065647238 -0.476587194 -0.511400273 0.011273671 0.119276098 0.038038278 -0.683963081 -0.172372512 -0.031444127 -0.011121448 0.318708753 0.175986716 -0.449535039 -0.396473833 0.007932013 -0.341915832 0.318748375 -0.043779254 0.387269059 0.465758143 -0.04599921 -0.395941833 -0.004272537 -0.009501074 0.007977609 0.037660166 -0.148817972 0.106407802 -0.361107055 0.114866581 0.10150755 0.074664639 -0.390518337 0.336222754 0.20916057 -0.020174773 -0.064965092 -0.100791239 -0.049624746 -0.170504093 0.2665947 0.189478077 -0.288598859 0.055833002 0.129057338 0.089274107 -0.028143068 0.320329508 -0.187566376 -0.132592666 -0.026172591 -0.282276886 -0.121945649 0.011206378 -0.03038363 -0.093284614 -0.036588645 -0.061534163 0.181738708 0.093276714 0.026283801 0.145137734 -0.080599128 0.002147834 -0.022357764 0.009819333 0.026841827 0.402540356 0.15538301 -0.098382552 -0.079219958 -0.158639849 0.042663724 0.286552841 -0.035399619 -0.039523259 -0.00093832 -0.139702422 0.098888169 0.06359711 -0.028016637 -0.025713137 0.003302333 0.017294206 -0.117915359 0.068301527 -0.077841826 -0.011612752 0.087304979 0.000154312 -0.092242143 0.125072668 -0.036513452 -0.019354666 0.087297176 -0.120429024 -0.075970326 0.010219132 0.041561839 -0.014645973 -0.09564002 -0.081633204 0.029730597 -0.058583042 0.037715796 0.016845177 -0.041556528 -0.019300409 0.021368642 0.01458998 -0.03227798 0.017899143 -0.05699828 -0.02199261 -0.008961766 0.0042969 -0.112101563 0.021260384 -0.009002407 -0.022904436 0.002419921 0.088448144 0.058754049 -0.052417148 -0.035257626 0.012873121 -0.069117681 0.103380339 -0.004579365 0.000470644 0.077463359 -0.004033868 0.018817251 0.014486798 0.025762013 -0.016791273 -0.086617215 0.009737196 0.007290176 0.021790418 0.05936329 -0.015768977 0.002277193 -0.019377375 -0.012053307 0.031494765 -0.043008266 0.00149885 -0.055448789 0.064838379 0.005590434 0.038238708 0.027494141 0.044163373 -0.02641695 0.032459133 -0.059125123 -0.037802237 -0.045575942 -0.099935144 0.04085223 -0.047484486 0.045530843 0.014251219 -0.007540571 0.019183797 0.048527071 0.000415498 -0.016303676 0.021983923 0.045152462 0.017617515 0.009917799 0.009468174 -0.045054858 0.008511924 0.014572167 0.014055469

sample_61 -5.957045489 -0.780272852 -0.486050131 0.910532496 0.992860885 -0.428605206 1.16439707 -0.379193806 -0.375136987 -1.181853991 0.794792139 0.139479859 0.467142284 -0.237502285 -0.086798069 0.381879158 0.291121653 0.003779932 0.254543397 0.17131343 -0.191574053 0.579868612 0.392081229 0.763168685 0.085217103 -0.851425512 -0.039235327 -0.028347106 -0.465500781 0.244294357 -0.131139687 -0.09185622 -0.092766927 -0.406523137 0.356575068 -0.155539848 -0.243628822 0.000763938 0.503429889 -0.5185055 -0.102210692 0.42037733 0.527386974 -0.356087582 -0.189177689 0.359465017 0.359525617 0.240760496 0.032562821 0.164308507 0.150051363 -0.175843064 0.12212425 0.087964628 -0.104712374 0.209000575 -0.144798114 0.250411023 0.125203832 -0.44184181 -0.135993854 0.148192524 0.085687483 0.005368424 -0.055100429 0.144011347 0.058366474 -0.157910981 -0.07732723 -0.211013597 0.131798686 -0.220649039 -0.181569734 0.118426222 0.190462293 0.017979855 0.13446581 0.029249306 -0.010892545 0.070897329 -0.054276634 -0.248482837 -0.335465821 0.015311935 -0.047263131 -0.207734276 -0.018065284 -0.052766257 -0.074581352 -0.023517375 0.023149437 -0.082287267 0.145575128 -0.22509319 0.004556853 -0.045779344 0.193918129 -0.038862295 0.14900137 0.007108997 0.053118784 0.070851684 0.118992718 -0.023251731 -0.089061793 -0.023859918 0.135929828 -0.021283397 0.037035922 0.039604545 0.152401105 -0.150618616 -0.037069195 0.044815979 -0.088324379 -0.130487282 0.096575949 0.13772523 -0.045534432 0.011707904 0.09913499 -0.036828416 -0.020061325 -0.182756667 0.009575999 0.000513811 -0.147412116 0.002126014 -0.167843219 -0.002349017 0.033808197 -0.020005726 -0.056879195 0.089615209 -0.196610544 0.010896455 0.082358915 0.075298713 -0.005931444 -0.041482674 0.002058044 0.020498453 0.19235489 0.040236732 0.095736135 0.018890643 -0.047396271 -0.21055981 0.08344799 -0.093127728 -0.038567946 -0.007408058 -0.010019719 0.03307283 -0.125814522 -0.001659482 0.022635578 -0.050185928 -0.111019772 0.101512571 0.087860669 0.08700706 -0.022776641 0.142928318 -0.015787095 0.079432856 -0.005338223 0.045248962 -0.02410394 0.100669441 0.063979495 -0.111645739 -0.017159046 -0.007873568 -0.073661476 -0.026042208 -0.131668691 0.005469683 0.088235743 -0.109359807 -0.046039183 0.075711387 -0.053133683 0.113707141 0.022925957 0.080860057 -0.001369499 -0.084982282 -0.025415715 -0.040036295 0.054701173 0.001437197 -0.066327986 0.098613595 -0.083275841 0.01666054 0.139507141 -0.095114157 0.045308278 -0.065881329 0.036376245 -0.045679291 0.092102862 0.062415079 0.033590592 0.086303331 -0.083288254 0.040609837 -0.045623103 0.046098183

sample_62 10.89999277 0.224900059 -2.799415458 1.898766605 -1.378952904 -1.899135614 -2.040411176 -1.055944683 -1.206664122 1.033261124 -0.765323163 0.799606456 0.344023275 1.842890875 -0.890359027 1.197412947 -0.111747256 0.464512189 -0.804886992 -0.066556613 0.09408084 -0.204206252 -0.231859683 -0.083837408 0.190785529 -0.324622074 0.294478042 0.06018093 0.061688028 0.246970489 0.213303666 -0.091940333 0.167788817 0.022159743 -0.121152686 0.284041365 -0.210321407 -0.157133009 0.370431236 -0.126203921 -0.689668113 0.518863155 0.09856623 0.43769113 -0.004755327 0.283934612 0.013126337 0.065014566 -0.230361464 -0.181131695 -0.066141372 -0.316616005 0.108841259 -0.092077579 -0.00297233 0.040668381 0.102023365 -0.206748794 0.044596931 -0.046710876 0.001353916 -0.190119319 -0.262048994 -0.052601223 0.187108722 -0.158804307 0.025014163 -0.165960625 0.090059978 -0.197247468 0.222456978 0.009611942 -0.171413408 -0.036448343 0.001040366 0.000718621 0.152136575 -0.120584405 0.000121278 0.131280631 0.150562723 -0.05276993 0.100623143 -0.232074716 0.064634202 0.069109726 -0.208412904 -0.084781349 -0.274702664 0.100403385 -0.124612807 0.099809717 -0.052556802 -0.050027645 -0.064747675 0.047870978 0.030901321 -0.215168658 -0.208906621 -0.057611082 -0.078759733 0.109437631 0.228622146 0.000365412 0.091231478 -0.245258401 -0.035149038 -0.012819779 -0.017821568 -0.038328209 0.020267656 -0.241213253 0.033550035 0.058616991 -0.07191276 -0.007250366 0.022927402 0.012793071 -0.015310059 -0.15879692 -0.000711578 0.0136394 0.058324282 -0.05926449 -0.045404677 -0.031064514 0.005073142 0.014927922 -0.038203082 -0.045870191 -0.026692458 -0.05512854 -0.030983219 -0.045628642 -0.044907268 -0.004072775 -0.009510502 -0.071042265 0.029017471 -0.08182168 -0.084147416 -0.033190772 0.062576613 -0.004976935 -0.026565138 -0.026423776 -0.053116391 -0.00244423 0.057555016 0.060506187 -0.112300883 -0.057099881 0.11539608 0.019382483 -0.009781154 -0.038252018 0.017644346 -0.054883822 0.062915325 0.150914195 -0.037541404 -0.006929545 0.042790483 0.006651077 0.060256805 -0.018632677 -0.071479149 0.041773195 -0.093779802 -0.011072171 -0.068403969 0.003487928 -0.003613043 -0.014406406 -0.006376885 -0.038054741 -0.008624693 0.030254203 0.00892141 0.047490772 0.048059745 0.01808941 0.033869974 -0.049542124 0.012222592 -0.005773155 -0.020068407 0.025080439 0.093109962 -0.024321145 0.036480379 0.080687995 0.034406511 -0.009914275 -0.042272577 0.020199807 -0.04169665 0.014116913 0.033730299 -0.040432296 -0.048691201 0.036593714 -0.022498891 0.045580174 -0.040197852 0.020287887 0.008274922 0.009772114 -0.027211213 0.086893016

sample_63 0.817651686 -1.739824622 -1.266228723 -0.184034144 1.492531442 -0.037403079 0.467564356 0.873017323 0.401586947 0.036860888 -0.796957001 -0.384964601 -0.002950827 -0.020775543 -0.171287279 -0.558327839 0.402662051 -0.060442353 -0.507645034 0.158307229 -0.481802438 -0.006763768 -0.127015979 0.139702261 0.117849184 -0.696756291 0.119132199 0.099880209 -0.265938937 0.017895319 -0.194501186 0.088696722 0.153391174 0.057836385 -0.222087895 0.159349743 0.11292778 -0.305327812 0.053526328 -0.089239954 -0.011922558 0.044209278 0.148593316 0.125120989 -0.023383184 -0.158902033 -0.173971194 0.161839079 -0.256023378 -0.187860942 -0.077428911 0.030196895 0.034236054 -0.094515536 0.139325541 -0.014161986 0.114933146 0.036272527 0.007531211 -0.068939642 0.032570759 -0.011092607 -0.071475367 0.147002706 -0.147377268 -0.030325344 0.064646153 -0.02668862 -0.117710426 0.08428021 -0.134772509 -0.154997064 -0.061191768 0.053056527 -0.056950944 -0.170882206 0.148486357 -0.114954116 -0.066648339 -0.046963846 0.158134033 0.243802284 0.095833798 -0.063160591 0.091892613 0.024045936 0.089381029 0.033784432 -0.009337254 -0.021576106 -0.085494477 0.014843278 -0.052656693 0.062341044 -0.086807398 0.07750749 -0.040381848 -0.059532879 0.097158731 -0.055696142 -0.104938169 0.031511346 -0.015445432 0.088750902 0.056473011 0.038173064 -0.043800406 0.089886349 0.022882706 0.005386821 -0.050551297 0.02277538 -0.029488988 -0.051960237 0.040223541 0.057212856 0.073264515 0.023745833 -0.052019248 -0.003113388 0.044285642 -0.038365247 0.056020639 0.04980859 -0.014344703 0.021831386 0.025240746 -0.018051233 0.004537057 -0.003875531 -0.009899947 -0.039886805 -0.008954839 -0.06565117 0.001928064 0.002747531 0.050747929 -0.042284388 -0.022571134 -0.028433591 0.026246224 -0.015925436 -0.037592682 -0.050979111 0.002043329 0.073168461 -0.025948975 0.036943017 0.022755923 -0.026079729 0.001671942 0.014422617 0.050415945 0.012441696 -0.008816034 -0.010307746 -0.040625337 -0.045254941 0.033072936 0.005510045 -0.021483664 0.002217166 -0.028598012 -0.00120665 0.002116272 -0.034019188 -0.037364657 -0.02973616 0.019662352 0.027107512 -0.03027826 0.028727333 -0.015356856 0.030398502 -0.011142167 0.026280895 0.00512157 0.007592345 0.00899968 0.006216749 -0.070472265 -0.009983954 -0.007573108 -0.012626218 -0.008799721 0.018834003 0.008864037 -0.046053303 0.001428862 0.03562111 -0.015121901 -0.022827761 0.018268217 0.002620689 -0.003662438 -0.001246096 0.000732817 -0.018671085 -0.009621269 -0.01413882 0.046645035 -0.051992467 -0.036401712 -0.016268956 -0.018238983 0.012235725 -0.004748699 -0.037530964 -0.002894353 0.006352405

sample_64 5.339752248 -0.087479647 1.796068914 -2.420212582 1.598060788 -0.307780109 -0.640723188 -0.114319403 -0.887614259 0.312648423 -0.126375571 -0.227469592 -1.120444734 0.002640496 -0.375039515 0.051784031 0.620875647 -0.194146029 0.044631113 -0.037795474 -0.382138104 0.13054713 -0.118903256 -0.37583751 -0.24014518 0.572719057 -0.026242826 0.115754588 0.090194414 -0.298971309 0.293365085 0.342573294 0.042891354 0.265259732 -0.445518206 0.019673037 0.188969681 -0.260661875 -0.116789984 0.075168365 -0.033550098 -0.030627705 -0.016757783 0.183950412 0.065779027 0.257033228 0.061311454 -0.082576258 -0.169580975 0.354863845 0.024436592 0.150940674 0.103624218 -0.381611921 0.013595589 0.166573028 -0.073144561 -0.22396433 0.11943875 -0.110640792 0.160993229 -0.061481907 0.184717983 0.033986112 0.199705654 -0.127098092 -0.276441054 0.186451093 -0.137051265 0.113862121 0.12617657 0.051598666 -0.151513446 0.124890812 0.144848286 -0.083536808 -0.050936711 -0.030925572 0.023152175 -0.102832622 -0.006362143 0.100329127 0.030149666 0.087269599 0.099404691 -0.089287568 -0.139973005 -0.071619773 0.01739506 -0.008182183 0.03555266 0.13335473 0.127871964 -0.134743284 0.046609786 -0.07898301 -0.06711734 0.019926056 -0.057804482 -0.06341907 -0.143900697 0.024868805 -0.104521496 -0.10685986 -0.011536653 0.091097694 -0.111627445 0.033571727 -0.027774602 0.003625023 0.008409523 0.048468944 0.060918111 0.097941061 0.032599117 0.015438803 0.13353257 -0.141340202 0.067495344 0.035251442 0.007245284 0.037902269 0.058780181 -0.026453196 0.064691771 -0.068154805 -0.102101948 -0.027406466 0.060025031 -0.101415562 -0.046211684 0.009820844 -0.0753213 0.079281016 0.062466252 0.002826755 -0.038756074 -0.013674905 0.026253592 0.043615832 -0.007265498 -0.076635764 -0.024591673 0.074844499 -0.043086486 0.029352619 -0.045124384 -0.129921672 -0.028536202 -0.101244146 -0.107514253 0.009354023 -0.006094814 -0.058951361 0.042263923 0.150644023 0.004585663 -0.073976391 -0.089516905 -0.014448857 -0.032706638 0.010967212 0.101219397 0.01051847 -0.010831255 -0.009895928 0.101236457 0.05460219 -0.057972718 -0.043213845 -0.023192294 -0.016414592 -0.047239445 -0.055307774 0.086532311 -0.019745163 -0.019100315 -0.024512255 -0.064195358 0.102248435 -0.027241229 -0.003001086 0.007197016 0.039234014 0.036255131 0.05659623 -0.062775729 0.005960888 0.036808676 -0.028188756 0.050247784 0.075789522 -0.015160829 0.000906656 0.002186915 0.075787723 -0.027884896 0.053455804 -0.081733397 -0.068056863 -0.036692754 0.005653866 0.093552567 0.000914596 -0.039062123 0.052889888 0.024264898 -0.048163354 0.000309124 -0.064873691

sample_65 -2.45949569 2.577574182 -1.134039509 -1.142762119 1.081501677 -0.374824818 -1.343319804 1.038897892 0.303535098 0.072591467 -1.044041069 -0.241179509 -0.545917977 0.648947366 1.215200282 -0.139823184 -0.32694347 -0.687535927 -0.703826148 -0.002298654 0.428991643 -0.489518358 0.107344011 -0.379834329 0.013188235 -0.679577227 -0.022665831 0.079912191 0.705556998 -0.204775244 -0.035787716 -0.027490113 0.307225169 0.121948879 0.29575749 -0.077876359 0.303449769 -0.193354162 0.083463511 0.106242471 -0.144618 0.355180486 0.075717346 -0.018850699 -0.091868503 -0.064412394 -0.037868685 0.170542063 0.008256485 -0.163544044 -0.14292216 -0.141283899 -0.121261299 0.191878176 0.213905088 -0.096397629 0.057549791 -0.29043079 -0.00383035 -0.0713653 0.306309183 -0.193550333 0.127790575 0.161433538 -0.073372357 -0.037627878 -0.039664794 -0.08743635 -0.029596823 -0.168034406 0.133866506 -0.011268104 -0.126200257 -0.138831149 -0.229060755 0.253664732 0.108843945 -0.046893566 -0.045071532 -0.103445203 -0.141093349 0.031740184 -0.007898797 0.102958843 0.024554873 0.201658875 0.1158194 -0.213623129 -0.137712068 -0.234352124 -0.03630365 0.085254139 -0.070735491 -0.051219949 -0.121534483 0.051164202 0.076796645 -0.117932821 -0.010073954 -0.082492091 0.080709179 0.101106846 -0.154686069 -0.048718157 0.088298686 0.018784708 0.026703118 0.037290665 -0.124276578 0.042981844 -0.11284751 0.008401363 -0.032228981 -0.01824922 0.01609642 0.080367303 0.009236606 0.070800117 -0.085111239 -0.013046762 0.107707276 0.083669214 -0.080040786 -0.023594838 0.045296516 0.103822651 -0.07321638 -0.03141588 0.05425104 -0.062579846 0.0194914 0.071722682 -0.096010068 -0.155081127 -0.116196985 0.096527 -0.015328044 -0.038445527 0.01032048 -0.058562878 0.008945307 -0.006973686 -0.033394367 -0.010349772 -0.046265536 0.086032236 0.039976395 0.089132542 -0.009320448 0.00275041 0.086227834 0.007793929 -0.095011472 -0.050565102 -0.031646272 -0.005701106 -0.039792699 -0.011910945 -0.046799147 -0.082441764 -0.036494828 -0.004750668 -0.047139874 -0.037436784 -0.003087212 0.011060148 -0.00024002 0.054847442 -0.056711454 0.030010828 0.040425056 -0.048584812 -0.038492563 -0.012489155 -0.002934866 0.078077256 0.072360151 4.95E-05 -0.030886482 -0.036158818 -0.053351248 0.028249838 0.021877978 -0.032896257 0.096788811 -0.003145775 0.02172655 0.124482552 -0.018483789 0.003278698 0.027818422 -0.04301139 -0.075127341 -0.055987227 0.055564829 -0.005701664 -0.085055964 -0.025963074 -0.06152308 -0.013942563 -0.014883409 -0.049477645 -0.017296156 -0.024617358 -0.012107152 -0.048751486 -0.001607305 -0.017737794 0.022468425 0.014437784

sample_66 12.69635728 -2.549664541 -0.25254507 0.72557414 2.153677582 -1.552063958 0.683670218 0.358030799 -0.692151962 0.246681807 0.274222746 0.631157974 -1.518236267 0.284110964 -2.806226016 -0.071942347 1.784824065 -1.122259149 -1.346764642 -0.148774881 -0.328532453 -0.56641106 -0.102617479 -0.117830047 -0.134555244 -1.180082383 -0.744900146 0.347611633 -0.113353699 -0.754721783 0.274591235 -0.289904743 -0.537239445 0.396328966 -0.25693441 0.229328351 -0.411114091 -0.307222776 -0.500378314 0.237547683 0.025570019 0.092843636 0.066997831 0.078460027 0.381482794 -0.400079099 -0.687652119 -0.036592492 -0.16188017 -0.216739899 -0.027371939 -0.012932038 0.144361785 -0.240087809 0.088184463 0.095626921 0.18098566 -0.174619398 0.557055894 -0.325987899 0.314640642 0.764286112 -0.043986627 0.099388803 0.002848702 0.093551915 0.069550409 0.375321224 -0.297651928 -0.088354929 0.111349215 0.303518417 0.165094198 0.002449853 0.189685385 0.08922537 0.128885916 -0.278394399 0.073294986 0.142888299 0.409121318 0.648068948 0.165364677 -0.189852475 0.154998264 -0.219980361 0.413596693 0.249793869 0.176029076 -0.173706578 -0.218291233 -0.194409538 -0.051882267 0.152909959 -0.105153817 -0.004392514 -0.295000166 -0.031180096 -0.037357556 0.037223645 -0.140187584 -0.083885739 0.05822662 0.14143036 -0.025211245 0.053298617 0.027032249 0.176950261 -0.050488638 0.07749162 0.171077249 -0.027277671 -0.093792395 -0.026703651 0.019769596 -0.022014864 -0.070254537 0.111565146 -0.068636914 0.19701314 -0.061835429 -0.053903138 -0.040242275 -0.202875688 -0.035303244 -0.180641772 0.008707835 0.043755769 -0.005855457 0.021067508 -0.043128127 -0.119032234 -0.153356438 -0.006547822 0.113292572 0.097321224 -0.126107012 -0.2196744 -0.095754172 0.012554862 -0.054192495 -0.076503785 -0.098819515 -0.074100782 0.153216138 -0.020517701 0.077218756 -0.109839266 -0.078800575 0.024134091 0.031893467 0.038917589 -4.72E-05 -0.01015246 -0.033817107 0.100406869 -0.014745941 -0.032211979 -0.058290953 0.070641646 -0.107549685 0.032540522 0.006860938 0.081006158 0.085196862 -0.001106322 -0.063927151 0.06195951 0.13918678 0.016699211 0.060032015 -0.051196159 0.124615608 0.187129058 -0.094886277 0.071873906 -0.143476904 -0.058445125 0.004653501 0.032385121 -0.015459269 -0.116840249 0.02296196 0.031702821 -0.086775063 -0.035109145 -0.113933365 -0.006140934 -0.118934812 -0.010676665 0.074060866 0.151134167 -0.015253257 0.029284592 -0.000331626 -0.108219593 0.026131612 0.110987796 -0.114842456 -0.023354713 0.052741668 0.05150989 0.14973732 0.054847994 0.028718249 -0.060212727 -0.010258985 0.026319529 0.077525352 0.046618091

sample_67 2.396402923 -3.763269019 1.87336751 -2.067939022 -0.657002971 -2.620146372 0.093395091 0.114305105 1.279763412 0.079329956 -0.13319886 -0.30351921 -0.546463635 0.464824495 -0.438835168 -0.786284426 0.206379297 -0.005489577 0.115332649 0.347884516 0.011863799 -0.089647562 -0.357223816 -0.103559953 0.325899389 -0.504505477 -0.107822228 0.018095796 -0.204790561 0.428567511 -0.204754192 -0.295491916 0.223121631 0.132645057 -0.452042467 0.608581112 0.235228904 -0.012944529 0.160785645 -0.014413643 0.306208855 -0.22033637 0.031142969 -0.114384939 0.114421678 -0.029613631 -0.152797519 0.149978528 -0.086298957 0.056212306 0.261376973 0.038532213 0.085051922 0.066520544 -0.008952177 0.040518074 0.016094559 -0.099881217 0.057122242 0.00628279 -0.099930039 -0.020928872 -0.085443564 -0.009830989 0.026891015 -0.093656554 -0.214659862 0.031786861 -0.007772291 -0.025884656 -0.051925642 -0.252658656 0.16535235 -0.007214523 0.18410813 -0.005268906 -0.090346125 0.011455639 -0.079856962 0.1310643 -0.000951649 0.088423423 -0.090599042 -0.017640385 -0.110988479 0.097653058 -0.003222664 0.086602486 0.018792323 0.051294408 0.151290507 -0.102649891 0.010752763 -0.061000467 -0.114675939 -0.076033988 0.071813616 0.240082397 -0.013531864 -0.147645064 0.04490968 0.046193081 0.032178406 0.037474911 -0.05233244 0.078428228 -0.041634107 0.082787962 -0.031102274 -0.009806001 -0.031975153 0.085250216 -0.013986462 -0.109602336 0.000917675 -0.019237702 0.003149522 0.068109399 0.138067721 -0.018123879 -0.058732259 -0.061814132 -0.069247508 0.122789913 -0.035460871 0.021588577 0.051206407 -0.077008035 -0.016612138 -0.074780351 0.018113884 -0.039610324 -0.010481937 -0.017797236 0.000152296 0.08027496 -0.004478504 0.092165444 -0.004459195 0.063477506 0.007533559 -0.012729605 0.033650057 0.029215664 0.097444278 0.004503693 0.034745589 0.021653137 0.071560494 -0.019125552 0.019541643 -0.001694518 -0.051913241 -0.015080887 -0.033843165 0.022343188 0.029706204 -0.041249374 -0.036076588 0.04623815 0.000407023 0.006071875 0.032125344 -0.058721823 -0.007110931 0.059606229 -0.005721528 -0.017189909 0.018795503 0.01299449 0.00784758 -0.026035095 0.019856742 0.047561963 0.016042226 0.039840008 0.017997043 0.061055941 0.01626055 -0.035480344 -0.057246835 0.033453854 0.038931734 0.004963774 -0.040890377 0.005776242 0.025737332 -0.094532741 -0.01563561 0.042332476 0.071449966 0.001267958 0.020811341 0.030494497 -0.027622955 0.048735164 0.029694186 0.002094601 0.013001797 -0.04260508 0.019904897 0.024318066 0.043529055 0.067432157 -0.023530745 0.004891358 -0.033694747 -0.021525692 0.050354515 0.02013435

sample_68 -3.378909308 -1.171337405 2.346396073 -2.129512695 2.013789725 -0.686326597 -0.131576728 -0.280857513 -0.762261857 0.298935869 0.434578453 -0.171902135 0.449510172 -0.450080217 0.217305858 -0.040688074 0.049206898 0.466193661 0.340201358 -0.099874241 -0.032705465 0.050959046 0.46244097 -0.756551549 0.220940878 -0.069376372 -0.0805258 -0.001291367 0.040549722 -0.136546532 -0.166207818 0.190826328 -0.195694265 -0.187469928 -0.281332181 -0.117478549 -0.045601654 -0.015472784 -0.087527099 -0.22273357 -0.040801168 0.061649427 -0.178047489 0.131866059 0.172111915 0.028786699 0.204900013 -0.185327187 -0.118003193 -0.061160173 0.180317247 -0.078874638 0.230342682 0.03621221 -0.033796512 0.105364893 -0.216415794 -0.026773285 -0.149295182 -0.030376967 -0.021040935 -0.001400132 -0.000239125 -0.112897776 -0.039518953 -0.062896656 0.126444457 0.1167561 0.208095604 0.05543451 -0.02532275 0.068095703 -0.231289331 0.130551947 0.052916059 0.025224775 -0.009487544 -0.105936727 0.029295773 -0.053493697 -0.027827113 0.134704583 0.029847936 -0.050148236 -0.144629497 0.077368753 -0.101116941 -0.130243397 -0.043789442 0.093910694 -0.011630398 -0.095601208 -0.002070076 -0.059595145 -0.038738043 -0.073607306 -0.010513511 -0.163695021 0.047023655 0.00239058 -0.003210228 0.074023601 0.138936763 0.0561546 -0.056010147 -0.046751349 -0.063619096 0.084918926 -0.037996675 -0.018691293 0.088031476 -0.005092651 0.047399014 -0.012057721 -0.093431709 -0.02348168 0.018307499 -0.084654284 -0.09540109 0.020205859 0.042761431 0.036198635 -0.051613677 -0.043002974 -0.043553619 -0.079032308 -1.90E-05 -0.014807825 -0.061698639 0.079393624 0.092606602 -0.094756888 0.057732987 0.020467294 0.014949465 -0.030050008 -0.059929939 0.075929284 0.003110368 -0.039532807 0.031871327 0.041168918 0.034630166 0.003325266 -0.027434567 -0.050988132 0.022016442 -0.042586178 0.050162299 0.028264562 0.016102897 0.066978718 -0.042152564 0.079117924 -0.060093442 -0.016410715 -0.027209335 -0.01167405 0.019615126 0.070915727 0.004430478 0.023749382 0.033725446 0.035931179 -0.005936254 0.051806976 -0.019101219 -0.032053748 0.016771429 -0.026259452 0.033766741 0.008111656 -0.020303565 -0.013252714 -0.057562406 0.070197235 -0.081404938 0.015523133 -0.01180193 -0.005085637 -0.00698365 0.005269341 -0.01456336 0.005960225 0.01463278 0.050498324 -0.041630145 -0.028081793 -0.010325669 0.006061419 -0.018309686 0.046617697 -0.010508742 -0.047950905 0.058217357 -0.00514277 0.028303934 -0.003506919 -0.042408195 -0.030376656 0.035705994 0.030552989 -0.033154089 0.012145993 -0.012998518 0.040381675 0.056084062 0.051810464 -0.000779084 -0.004794501

sample_69 6.122489214 -0.593843048 3.767859997 -1.276714463 3.111016166 -0.792957651 0.049099459 1.42119179 -0.308773369 -1.01455636 0.155123087 0.735922346 -0.436572803 -0.648131023 0.178060362 -0.2396761 0.268993537 0.305057096 0.548752318 0.583080956 0.358046744 0.273089489 -0.052209414 -0.027750212 -0.139593525 0.399147397 0.082124493 -0.18558421 0.32153167 -0.386830092 -0.208551238 -0.188937603 -0.519570977 0.087864589 0.065240309 -0.007482175 -0.052071586 0.419888212 0.09282077 -0.322596439 -0.056990534 -0.08990804 -0.40806114 0.023902841 -0.367074742 -0.059786547 0.235940507 0.031743857 -0.202024288 0.178566343 0.26800581 0.029122651 0.112242167 0.05934446 -0.056903489 0.020438738 -0.104038719 -0.350211315 -0.017637435 -0.10461412 0.062718967 -0.107912654 -0.112349808 -0.043111751 0.121017286 0.068204369 0.223946017 0.002800691 -0.028550844 -0.154614463 -0.209368847 0.091437596 -0.262591524 -0.036250968 0.197395549 -0.029431089 -0.205204577 -0.028861323 0.006313486 -0.1570049 -0.141462435 -0.146918722 0.055917088 0.182960968 -0.05211354 -0.123791162 0.037741525 0.082451027 0.16445879 0.079154121 -0.051552635 -0.008711113 -0.012579757 -0.142222142 -0.069567762 -0.125249998 0.090703076 0.071501765 0.226257226 -0.035024695 0.057095122 -0.000736749 -0.027630035 0.06029985 0.066625148 -0.120612579 0.006699567 0.044655967 0.013025627 0.046048423 -0.093866634 0.034005892 -0.002713677 -0.09017926 -0.164056593 0.009343206 0.026616807 0.023192119 0.044381463 -0.09558262 0.077173524 0.056016943 -0.054608222 0.07350583 0.053840449 0.052792289 -0.024824303 0.037467617 0.006504043 -0.030849468 -0.092252903 0.005591776 0.053600689 -0.108925263 0.057974256 0.032442379 0.094314014 0.012665797 0.001982119 0.027209459 -0.015339932 -0.023726627 -0.017641961 0.027571767 0.048263867 -0.0064517 -0.055655162 -0.077713521 -0.003095625 -0.055120871 0.013345954 -0.001095206 -0.087420263 -0.093179484 0.045592401 -0.0167993 0.015855198 -0.041026549 -0.024241626 -0.046115916 -0.012416622 -0.024984679 -0.000621643 0.015849051 -0.068588929 -0.048813291 -0.022948945 -0.046626876 0.047296206 -0.068141174 -0.019040337 0.019008307 0.004840364 -0.081532786 0.044698173 -0.062595524 -0.007095712 0.01384096 0.072490901 0.022870453 0.063695186 -0.005036569 -0.028014468 0.06310318 -0.025308878 0.042820005 0.075120173 0.056254599 -0.020346603 0.029314936 -0.032802273 0.044950069 -0.026470153 0.036513711 0.047242644 -0.044170798 -0.037298542 0.003186165 0.027793284 -0.078470043 0.017152779 -0.047177121 -0.011152361 0.003426692 0.006524322 -0.067410027 -0.057052817 -0.054761578 0.014792538 -0.029480441

sample_70 5.684588596 -0.951810539 3.263708704 0.952660178 -1.340050435 -2.609950542 -0.633055796 2.069553845 2.034018526 -1.724701055 1.048013375 0.067153754 0.561176706 0.333293516 0.352452408 0.416632221 -0.21207883 0.788266348 0.097011151 0.329955059 -1.053603759 0.856476948 -0.269353329 0.230197437 0.466590671 -0.076606186 0.510699152 0.251809094 0.096448629 -0.01526321 0.253322569 -0.819863209 -0.59122212 -0.249831529 0.289478383 0.176764293 0.354227741 -0.246613624 -0.240377006 -0.376235621 -0.099424195 0.034527799 -0.028555646 -0.039690356 0.341990166 0.040298695 0.301599283 -0.061543577 0.373880009 -0.376150575 0.149296032 -0.130385817 -0.037976366 -0.289290757 -0.062683796 -0.220579158 0.280908348 -0.179369513 -0.066218227 0.123544087 0.201489732 0.000513797 -0.229232047 0.002367219 0.238376406 0.200001706 -0.2553039 0.382724486 -0.002772333 -0.14226434 -0.084603092 -0.007496286 0.315346713 -0.029012257 -0.047002013 0.172228704 0.140839346 0.147731329 -0.195245863 0.191314519 -0.232249928 0.033194777 0.052842562 -0.188965026 0.178582858 0.032354098 0.190040943 0.006625547 0.113117046 0.034144274 0.104952522 0.106283175 -0.110077092 0.157303948 -0.188713956 -0.137650668 -0.124669686 0.024139353 0.12367727 0.071106005 0.192148236 0.115655444 0.049022778 0.230253177 -0.09162772 0.003407884 0.179154675 -0.022400434 -0.056692943 0.099596779 0.060381193 -0.005429996 -0.185588063 -0.024653053 0.146152346 -0.094440042 -0.075584038 -0.025232288 0.014522041 0.082663802 0.027175789 0.033075544 -0.100043434 -0.039798703 -0.087292163 -0.095774198 0.113065611 0.00233711 0.152175429 -0.047585014 0.140774902 -0.026287352 -0.163389928 -0.138682783 0.002133703 0.008027798 -0.054632657 0.149536902 -0.103071433 -0.027948978 0.059884349 0.05464821 0.139531082 0.052038524 0.202431446 -0.02160371 0.024044342 0.178715167 0.035409149 -0.096015687 0.119113402 -0.118017193 0.091907073 0.007673409 -0.003874272 -0.097960881 -0.089465649 0.067072884 0.069218213 0.054382384 -0.000917517 0.132517721 -0.066631925 -0.057105903 -0.07204884 0.067954816 -0.029222973 0.058321351 -0.043798065 -0.061230037 -0.04140576 -0.137656353 -0.12335931 -0.145490653 0.085652728 -0.08534805 0.139766962 0.070775431 -0.074577905 0.104849644 -0.004262731 -0.019435784 -0.054710646 0.109419122 0.079906293 -0.079050361 0.140519556 0.001133348 0.068846292 -0.021069853 0.049751371 0.016038893 -0.059308643 0.028265537 -0.03043976 -0.045721817 0.028664895 0.013035898 0.05772652 0.079314161 -0.152160726 -0.071885884 -0.024002604 0.039247154 0.121565419 -0.015129975 -0.015320384 0.033282142 -0.046546847 -0.129280241

sample_71 -6.995746159 1.850288211 -1.124113818 0.708468715 1.989716889 0.107123144 0.273676245 -0.312973003 0.154114063 0.72394199 -0.716334145 1.136561976 -0.410281216 0.58975417 0.011014804 0.616566201 -0.050835244 -0.604138712 -0.60407173 -0.067345174 -0.080600791 0.347750576 -0.615642227 0.01896147 0.183762287 0.313217843 0.400764857 0.245177742 0.032538837 -0.084316424 -0.073476979 -0.178152523 0.049362715 0.314012112 0.138995162 -0.337351577 0.106587863 0.005565191 0.109442306 -0.112525202 -0.260646308 0.155993061 0.076233988 -0.050533917 0.463711116 0.097093244 -0.104033681 -0.00773996 0.089814835 -0.016303552 0.012192708 0.1652644 0.257159656 -0.214292274 0.163191805 -0.026629669 -0.061772814 -0.158588183 -0.090916418 -0.029091926 -0.008575699 -0.125144744 0.01124716 0.12805687 0.00496656 0.084055797 0.007344336 0.083362871 -0.082215153 0.026374137 -0.033811793 -0.152232249 0.186235265 -0.010444844 0.136273108 -0.128076724 0.107668686 -0.071649309 0.150285411 0.106316004 -0.066876912 0.056503713 0.064765937 0.101251459 0.122623111 0.058357264 0.106902081 0.11051436 0.105116264 -0.184086661 -0.036990468 -0.024299116 -0.046120311 0.039466795 -0.098940168 -0.054412697 0.064305269 -0.022866164 0.050484066 0.024284213 -0.025695359 0.008590951 0.056441682 0.093501408 0.004949678 -0.004420403 0.032418221 -0.067444015 0.029010808 0.022464624 -0.048480826 -0.034630308 -0.088542942 0.035017271 -0.003867407 0.121240347 -0.02151044 -0.051881199 -0.090216055 -0.091549349 -0.00223876 -0.03509573 -0.090931417 0.045901739 0.090665453 0.161475066 -0.109317111 -0.070712075 0.025102246 0.043276956 0.194643335 0.074847443 0.02934305 -0.163974604 -0.024532015 -0.073300248 0.03924768 -0.074469576 -0.106441394 0.103954428 -0.007326227 -0.039004752 0.003574288 -0.010881143 0.068018318 0.010648382 0.073667157 0.000381945 -0.007458685 -0.082390555 0.097611267 -0.001777407 -0.009778231 -0.024174274 -0.004581977 0.085210764 -0.074439868 0.036747627 -0.0205199 0.055121729 -0.035174932 -0.012059333 -0.020662455 0.01583842 0.020346802 0.037876999 -0.074597323 0.086226651 0.06627269 0.000179174 0.077847952 0.01553052 0.044698108 0.025651108 0.008333249 0.024557838 0.096800102 -0.01740604 -0.028036831 0.008293592 0.044226209 0.065267904 0.019106273 0.037230195 -0.014824906 0.104972762 0.030942872 -0.077780942 0.000189231 0.015657509 -0.041963916 -0.067443056 -0.016457591 -0.019680575 0.018908569 -0.013211568 -0.000261806 0.059273711 0.000337546 -0.006823193 0.000114509 0.039162452 -0.025248813 -0.001544726 -0.059985004 -0.010233975 0.004376682 -0.048058923 -0.038552772 -0.026061884

sample_72 2.051808255 -1.527840081 -1.689996052 -1.541829237 -1.816093627 0.744054608 -1.257990371 0.754980794 -0.21730185 0.717284958 -0.424283363 -0.831092436 -1.041102491 -0.15832052 0.136532147 0.460181451 0.809545706 0.158577132 1.085200254 -0.512364951 -0.347092781 0.41559667 0.094586025 0.291296462 -0.809398941 0.372205035 -0.145201544 0.414676282 -0.064858271 -0.416696841 -0.390671503 0.223939162 -0.508453933 0.196730571 -0.363156099 0.719090425 0.215032646 -0.255913855 0.220228208 0.26381186 -0.185478589 -0.173113802 0.084276523 0.013879872 0.080691067 -0.207519435 -0.422370821 0.189888722 0.046184987 -0.245236697 -0.012186597 0.152377259 -0.039621969 -0.147199496 -0.06548013 0.068209263 0.357404746 -0.083420067 0.115456772 -0.039078151 0.117892052 0.039385825 0.235733774 0.038414071 -0.203848836 0.054703979 -0.192390058 -0.013865522 -0.211326993 -0.209892953 0.052397718 0.063476883 0.22676564 -0.092169749 0.050321811 0.032049694 -0.031694721 -0.011811619 0.12718395 -0.052544935 -0.111903182 -0.048022624 -0.005427713 0.122953224 0.177987548 0.037645514 -0.106567086 -0.041278916 -0.111730064 0.249452038 0.094737424 0.151374468 0.041915778 0.105434817 -0.021474062 0.27750066 0.04381472 -0.049710554 0.101274921 0.027217357 -0.012617461 0.009258317 -0.061331578 0.0571082 -0.066385855 -0.091651215 0.040599107 0.133549231 0.104514381 0.037117449 -0.039457588 -0.089337239 -0.132180042 0.06719498 -0.117609375 -0.027180281 0.023712593 0.148227632 0.008371007 -0.093678952 -0.069453513 -0.118634439 0.163431209 -8.84E-05 -0.003973753 0.005113011 0.193271196 -0.024673781 0.081601971 0.155083374 -0.06530524 0.010919315 0.093961453 -0.065561916 0.011464136 -0.072841361 -0.126640193 -0.04662188 0.186633421 -0.071763957 -0.044591346 0.052900606 0.02721882 0.047330635 0.001448993 0.028943644 0.150438357 -0.02392522 0.090293039 -0.007530526 0.002165175 0.033902268 -0.134294047 0.042593047 0.026162086 -0.018790055 0.083287255 -0.009322117 -0.066840026 0.103818378 0.015874396 -0.016020449 -0.050344333 -0.032263464 -0.028635208 -0.0172695 -0.004653297 -0.028414988 -0.046584394 0.049115221 0.123082362 0.004296477 -0.046731995 0.059009099 0.099342365 0.144320865 0.022482881 0.004343824 0.084397559 -0.008915212 -0.0572922 0.034727169 0.075568987 -0.068083448 0.007539588 0.070165565 0.028433114 -0.002880932 -0.009465914 0.011174409 0.018399531 0.036834524 0.073159146 0.029265168 0.097933689 0.010359913 0.042996732 -0.054696788 -0.038685312 0.049301999 0.055733246 0.00644968 -0.005147951 0.014249129 0.026018585 0.01733969 -0.003002856 -0.024569265 -0.039459419 0.042734624

sample_73 2.355338744 -1.804938931 5.301770252 1.619020708 0.486838207 -1.503891252 1.279455433 0.240355883 0.132779631 -1.064259604 0.693193297 -0.622030227 0.862949753 0.475160615 -1.128003376 0.985936973 -0.813864934 0.212698304 -0.172764755 -0.577904346 -0.037251588 0.525700105 1.205801469 0.786489498 -0.08926801 0.517760625 -0.248152102 0.13387354 -0.077326085 -0.094528847 -0.038658254 -0.416398465 0.208253592 -0.145618743 0.256741871 -0.286939163 -0.524431058 -0.333343171 -0.747701553 -0.119806001 0.279369933 -0.511368236 0.366979493 -0.203490386 -0.326201872 0.262050703 0.343452388 -0.360441859 0.341059998 -0.340024687 0.150129639 -0.023302917 -0.097648183 -0.173173828 -0.05909713 -0.012338154 -0.007856588 -0.012918639 -0.156021168 -0.05258426 -0.210677542 0.088290976 -0.165143748 -0.013534632 0.261370138 0.06007724 0.215331513 0.259351011 0.194885636 0.028282201 -0.284757527 0.362468412 -0.069460027 0.310260595 -0.297702871 0.275989594 -0.09617957 0.23073224 0.197847583 0.060986272 0.085474686 0.204132839 -0.023350648 0.036586299 0.204454735 -0.046951169 0.042874759 0.006381106 0.066608658 0.008057538 0.134619697 0.178752053 0.180913495 0.201913832 0.262943421 0.080773021 0.015518375 0.034906687 0.006543712 0.095946376 0.110637229 0.064076842 -0.025942011 -0.036004756 0.223458359 -0.075799194 -0.129903004 -0.028721543 -0.100927228 0.097658381 0.053442941 0.026922039 0.130481037 -0.064645577 0.073355792 0.026138827 -0.07178465 -0.157647566 0.135069352 -0.115882965 0.058312471 -0.071977678 -0.012572509 0.038911033 -0.064199381 -0.068279721 -0.034941999 -0.052621138 0.046873803 0.059455436 0.006968363 0.020173511 -0.004172287 0.035735736 -0.108752468 -0.181127424 0.025510332 0.125587761 0.064930279 -0.058923295 0.026266015 0.02949763 0.052064179 0.073000109 -0.05610417 0.112318688 -0.010176089 -0.039682357 0.051551966 0.1039552 0.025549356 0.071164849 -0.056388048 -0.030075085 0.050348808 -0.017493769 -0.033976154 0.027474155 0.083279216 -0.066974185 0.076333844 0.074051942 -0.041371504 -0.025411656 -0.114560249 0.017015858 -0.008711728 -0.026126042 -0.08122822 -0.050161818 0.057829894 0.027315733 -0.079947747 0.06825139 -0.045285535 -0.012497061 0.104738849 -0.022133414 -0.003975789 -0.028464829 0.054834914 0.075905163 0.099812441 0.025445124 0.033769664 -0.002467983 -0.051032266 0.07824815 0.026464684 0.060535847 -0.047945015 -0.107902533 0.00143947 0.019419452 -0.006942481 0.004131568 -0.018397332 0.096306976 -0.10558829 0.009968582 0.014335152 0.014813855 -0.060668048 -0.025955175 0.047628215 0.081389316 -0.07918426 0.067941758 -0.023879963 0.031253919

sample_74 8.058314229 1.56914789 2.491845977 -1.804252678 3.053181782 -0.933621985 0.870232602 0.855563387 -0.296008375 -1.335200336 0.623293991 0.052411038 -0.643440596 -1.230189321 0.049157572 -0.716408312 0.148262488 -0.019088447 1.102828978 -0.013571805 0.706934961 -0.20266326 -0.304435794 0.034808143 0.521104315 0.395329905 0.151186284 -0.604639667 0.080858014 0.097478322 0.053104054 0.680516151 -0.022912799 -0.43873545 -0.263335333 0.024550854 0.159144747 0.450870821 -0.356826323 -0.274471995 -0.528163814 -0.151486101 0.34678126 0.255987349 -0.1640222 -0.463361811 -0.048817132 0.197721069 -0.506628961 0.191978467 -0.122772021 0.148539952 0.184498977 -0.35889617 -0.118203351 0.004182542 -0.041400791 0.282586146 0.010837587 -0.171002126 0.161519789 -0.165714147 0.047566721 0.13283992 -0.029375516 0.003487656 0.033857298 -0.173772306 0.044580807 -0.161257456 -0.300722965 0.043473585 -0.391969063 0.43234059 0.152426333 0.04198592 0.038814636 0.191116598 -0.104521888 -0.211033325 -0.15782681 -0.230356099 0.114937501 0.067080642 -0.122796003 -0.122803194 -0.155878859 0.005407347 0.113019476 -0.112602109 -0.061690336 -0.01856962 -0.028061319 -0.044819563 -0.13663991 -0.215660325 0.147940228 0.021016832 -0.067608913 0.137292481 -0.011440995 -0.052752184 -0.196072917 -0.029213108 0.016000731 0.087983804 -0.152658774 -0.023256248 -0.142592274 -0.029721293 0.040817861 0.067835697 -0.187975413 -0.08733967 0.044883585 0.08165114 0.023777999 -0.05016711 0.025199252 -0.022718473 -0.127082046 -0.054447135 -0.106128246 0.064809355 0.049443692 0.089597651 -0.012613662 -0.085049634 -0.09914653 -0.007917001 0.032878851 -0.070760903 0.09194512 -0.031577496 0.121305833 0.060615679 0.094404854 -0.021278691 -0.047311915 -0.081550963 -0.046175643 -0.005498355 0.055559381 0.05792767 -0.07814353 -0.025572826 0.016166633 -0.008084753 -0.064439165 -0.048275856 -0.028839689 -0.035063706 -0.055786572 -0.073625176 -0.044017222 0.001767522 0.066376057 -0.060893463 0.033664528 -0.008248962 -0.024110207 0.02008677 -0.050621378 0.044026923 0.026760858 0.053056215 0.080322382 0.024193428 -0.030965965 -0.037306078 0.02998365 -0.011613349 0.001799445 0.01228773 0.088972289 -0.006006535 -0.076811501 0.043548617 -0.007173263 0.111976577 0.034384503 0.073602696 0.071076683 -0.055610719 -0.028210487 0.003160173 -0.041734376 0.002473435 -0.016740421 -0.014250801 -0.034934311 0.024789208 -0.040971157 0.011315005 0.045634622 -0.015472357 -0.04098674 0.06012831 0.020567273 -0.057491886 -0.027713112 -0.028169639 -0.039023171 0.015775642 -0.046150536 -0.0387038 0.021451054 -0.036808913 0.043187206 -0.006934204

sample_75 8.058342294 -4.27734303 6.11098846 2.079801897 0.705701917 -1.67710869 1.976676283 0.784011061 0.105794347 -0.348791701 0.437037775 0.582807279 0.61973169 -0.595892966 -0.410124407 1.158894577 -0.673377845 1.058962256 -1.344856017 -0.574551568 0.137949928 -0.320364117 0.622033705 1.095174409 -0.722004801 0.583360301 -1.280145163 0.858791069 0.256289045 -0.136204946 -0.915641772 -0.00181686 0.650776487 0.324039848 0.38561028 -0.436594281 -0.065289926 -0.425688886 -0.420813398 -0.392502073 0.275095873 0.211960746 0.154400347 -0.65108057 0.129195665 0.259129544 -0.036220099 -0.169758979 0.306225841 -0.040230443 -0.647444691 0.002121927 -0.071314534 -0.142777378 -0.072426899 0.174159999 -0.227536119 0.042436168 -0.497162278 0.395930535 0.096519034 -0.001696546 0.025926664 -0.336133755 -0.111592827 0.125435473 -0.178861999 -0.016478627 -0.082157512 0.518556332 0.012169049 0.157832983 -0.101996999 -0.051140938 -0.067878769 0.063038834 -0.094204341 0.082855975 -0.14525789 0.131240502 0.075175647 0.051553339 0.286800418 0.100305664 0.111318162 0.234375574 0.045208801 -0.027199779 0.158158026 -0.20941145 0.033290812 0.154143881 0.067655934 0.067968805 0.100796448 0.105336392 0.211274303 0.010758499 0.021908512 0.319794337 -0.123054005 0.088132829 -0.155044119 0.128576 0.016074463 0.05971413 0.087678707 -0.223172183 0.093190038 -0.086546 0.043585763 0.030063343 0.137330417 0.013749347 0.154980608 0.109469242 -0.049853135 -0.127581822 -0.019035309 -0.009060984 -0.152672061 0.191299888 0.141569466 -0.005323341 -0.053265856 0.057599778 0.151469874 -0.057580439 -0.014412922 -0.116544765 0.001379897 -0.16639525 0.056884124 0.0317013 -0.016571703 -0.009800499 -0.06964114 0.120745159 0.025841493 0.022994682 0.02533241 -0.062941455 -0.105182861 -0.012385291 -0.029707152 0.055643764 -0.060169565 -0.060824789 0.042648509 -0.030369636 0.109331726 -0.026252268 -0.012477661 0.001454825 -0.005889079 0.026115769 -0.047130677 0.08187144 0.115225106 -0.175812612 0.041474549 -0.056892906 -0.111803497 -0.076147298 -0.022369973 -0.027285409 -0.161101359 0.005182356 -0.124367502 -0.060987676 0.003291094 -0.041824166 -0.089444959 -0.129029479 -0.154782083 0.015035953 -0.069827045 -0.018023875 -0.008267621 0.05088487 -0.10553336 -0.000496354 0.02683434 0.084474889 0.031745801 -0.04367039 0.068554631 0.064817179 0.021421651 0.02981019 -0.035986566 -0.060553742 -0.052767668 -0.089370706 -0.003873616 -0.011836816 -0.020093392 0.092505892 -0.040767547 0.029148372 0.034095576 0.027823277 0.025442988 -0.025142237 -0.06346729 0.110172997 0.004759454 0.00570231 -0.055565341 0.054470612

sample_76 4.71094707 2.626603723 1.316836393 -1.042438434 -0.791344209 -0.507106519 -0.722301218 0.176179913 -2.275722754 -0.812313383 1.711144792 -0.568497438 0.085160445 0.515732219 -0.107562026 0.089777895 -0.231304754 -0.108182397 0.465622388 -0.195923652 -0.330578878 0.307291495 -0.800480421 -0.247589756 -0.483584984 0.156993385 -0.469385867 0.206093001 0.622726684 0.139101616 -0.144562862 0.237899196 0.211741999 0.505444398 0.965679514 0.276123814 -0.002401037 0.316000659 8.86E-05 0.010940778 -0.258343128 0.015538733 0.447989572 0.457445386 0.073844979 -0.221451042 -0.108550172 -0.289808964 0.140785843 -0.503641874 -0.157681837 -0.012199403 0.009873871 0.055322659 0.075056271 0.094769889 0.168546571 -0.26530271 -0.409242833 -0.093849041 -0.006884278 0.136690029 -0.477848057 -0.228414416 0.047853185 0.038933881 0.279294508 -0.053336634 0.234391107 0.186941174 0.009022896 0.106252441 0.065428483 -0.033648439 -0.011079206 -0.184573105 -0.082239017 0.04752768 0.048292239 -0.041333906 0.03340785 -0.096833915 -0.236302077 -0.071936178 -0.000196233 -0.1702392 0.150905667 0.007850793 -0.077206021 0.03735416 -0.094455028 -0.047731112 0.120602588 0.084901371 -0.010599169 0.003612838 0.106312578 -0.038737853 -0.076041936 -0.036249147 0.134549519 0.048128392 0.144941873 -0.154164163 -0.00423351 -0.077004201 0.069977427 -0.10797325 -0.006009074 -0.101343568 -0.109824668 -0.087291794 0.044874458 -0.05569169 -0.085576938 0.209824441 -0.040081414 -0.037027927 0.041949819 0.001827218 -0.053075683 -0.057584205 -0.158830921 0.017351658 0.107934748 -0.009598725 -0.010745156 0.067889986 0.061685993 0.18447648 0.100102049 -0.137143975 0.005560949 0.072794631 -0.108171657 -0.058438334 -0.083944827 0.023245688 0.142352854 0.138466866 0.101359381 -0.035790038 0.015042136 -0.10045448 -0.065021999 -0.084999812 0.005567509 0.009507439 -0.031043491 -0.04820485 -0.00656759 -0.032752442 0.012936826 0.059220882 -0.089233331 0.020811879 -0.108417848 -0.03445712 -0.107890387 -0.086963442 -0.03625519 -0.032904845 -0.034061646 0.021410412 0.023547249 -0.040642774 0.11506213 -0.05740928 -0.056680562 -0.064320934 -0.12815628 0.080847738 -0.073538272 -0.001546444 -0.044145624 -0.115748008 -0.031187624 0.103355352 0.007942054 -0.118868691 0.072333608 -0.087160716 0.02697377 0.023700952 -0.000391404 -0.011236336 0.012201692 0.014899062 0.074186591 -0.049000366 -0.04223041 0.012323104 -0.018962883 -0.04736964 -0.034516346 -0.012582559 -0.076346972 0.063542266 -0.050861251 0.036413536 0.080972856 0.013433677 0.026100113 -0.035754426 -0.022356861 -0.110469972 -0.08372918 -0.046855124 0.002914846 -0.036751683

sample_77 -7.749477399 3.663405045 0.720820643 3.62622655 2.013765583 -1.387991183 -1.875245208 -0.377970115 0.520240976 1.680913202 0.551755108 1.085188066 -0.273497022 -0.543501373 -0.081191374 -0.801235885 -0.471366399 -0.806531432 0.066459938 0.94313458 0.101544431 0.964018154 -0.346750167 1.28187832 -0.282718323 0.290030819 -0.741316376 -1.015756754 -0.639407371 -0.566207787 -0.595174968 0.462308585 0.584059196 0.163405515 -0.478662191 0.365971609 -0.106549995 0.518930863 0.241387273 -0.337717087 -0.370583303 -0.228221265 0.077539732 -0.055888737 0.429803351 0.243845952 -0.33377544 -0.639833577 -0.200451023 -0.386379654 0.101376823 0.006846532 0.206161317 -0.354877901 0.3495034 0.178014618 0.102840831 0.169556802 -0.271310132 0.38220834 -0.077209913 -0.278561623 0.42711183 -0.011239107 0.146286728 -0.022724479 0.161472187 0.554358761 -0.031177425 -0.389851753 -0.219729239 0.334596163 0.335841815 -0.109149256 0.403274585 -0.04123064 0.308625788 0.105117202 -0.242469995 0.12903265 -0.272008826 0.182309241 0.00573095 -0.155440996 -0.022729626 -0.074585391 -0.325392514 -0.03563583 0.110308402 0.16199173 -0.205740506 -0.042916693 -0.04895225 -0.00760684 0.14441371 0.137877387 -0.094650949 0.060323723 -0.124099454 -0.180265296 -0.136719977 -0.087924206 -0.03671699 -0.072552588 -0.15161493 -0.121374807 0.245417622 0.087924712 0.117705653 0.030270211 0.14071251 0.074791159 -0.178526118 0.057460025 0.001686656 -0.055875266 -0.04182292 -0.308538505 -0.093524766 0.056252358 0.169048142 0.087396076 0.071865927 0.019932529 0.218629099 -0.103227982 0.055233214 0.019025074 -0.024404664 0.04590526 0.062734297 -0.040224962 -0.123003012 0.057160283 0.046644913 -0.010290401 0.100362603 -0.119859449 0.05403432 -0.163997903 0.078045062 0.169969356 -0.047037776 0.058062735 0.031110518 -0.042782205 -0.038917145 -0.076855973 0.018338667 -0.101513832 0.047555765 -0.088952064 0.099072096 -0.003440591 0.019819167 -0.023201431 -0.021517293 0.081726013 -0.121904283 0.170208881 -0.004299865 0.089899678 0.008866786 0.183081646 -0.061801686 -0.12303042 -0.082285938 0.048192745 0.135449563 -0.013531463 0.050267512 0.205221779 -0.263896009 0.016101816 -0.009231244 -0.035631934 0.107694389 0.086110424 -0.204405196 -0.159583189 0.018084356 -0.127815025 0.089015554 -0.019336384 0.077290606 0.089466037 0.076057825 -0.006652402 0.130998271 -0.040358989 0.066970605 -0.033478795 -0.01731441 -0.028316695 0.122143572 0.034476815 0.035746479 0.112989528 0.033214428 -0.044925073 0.011882148 0.002070497 -0.030465293 -0.087536342 0.01592713 0.014517883 -0.090916977 0.139144451 -0.010666717 0.004890447

sample_78 0.203864205 -1.683502795 3.826449112 -0.620011906 0.935671139 -1.380651861 0.92461925 -0.187774379 -0.904643484 -0.916161744 1.130328526 -0.04815349 0.826311646 -0.756672136 -0.810201818 -0.373669911 -0.176597739 0.656729267 0.022414476 -0.269082458 -0.325220309 -0.29771829 0.755738666 0.329303482 -0.029712791 0.226047524 -0.691063949 0.409486218 -0.221912318 0.105707914 -0.140330042 -0.135601725 -0.185794647 -0.174144012 -0.270068739 -0.106993629 0.127398576 -0.028432708 -0.15935503 -0.109624505 0.297888872 0.189887459 -0.016009789 0.043920076 -0.049375481 0.140047565 0.311553786 -0.323456714 0.136101424 -0.252209229 -0.242658522 0.027428466 0.057395494 0.009036733 0.173418521 0.25195669 -0.017948701 -0.075029341 -0.369867231 0.138676734 -0.259057773 -0.08084576 0.227228932 -0.033384937 -0.180175712 0.029585678 0.017245824 -0.021888598 0.176163144 0.113822105 -0.109210895 0.165680827 -0.088435842 0.097545466 0.026438368 0.139705667 -0.003259835 0.081301593 -0.094089353 0.066724402 -0.034373731 -0.047575625 -0.026055266 0.046016049 0.244187455 0.072340805 -0.157955432 0.010500143 0.077060423 0.085771115 0.050777845 0.084365044 0.003721114 0.09822323 -0.049089643 -0.020398272 -0.025602559 0.003884897 -0.070271965 0.109667722 0.064553298 -0.023942778 0.042463438 -0.081293745 0.036362959 0.093199063 -0.132025874 -0.082581864 -0.057924455 0.124118453 0.071481235 -0.062488605 0.176049521 -0.073229338 0.03791579 -0.014598648 0.029031535 -0.057545832 0.038782353 -0.084908391 0.015178732 -0.027898415 0.075340032 -0.043811621 0.008722468 0.01320599 0.054869483 0.015697001 0.023177649 0.096651553 -0.01030564 -0.02935491 -0.006323165 0.007333502 -0.097682737 -0.007530884 0.037211048 -0.008729782 0.01355449 -0.048710944 0.050070755 -0.03346187 0.075509823 -0.043477829 -0.051774376 0.036036953 -0.027712629 -0.100820487 0.033339274 0.113809551 -0.043383583 0.062004224 -0.002718545 0.011091167 0.027773906 -0.015604788 -0.013577885 0.010039516 0.082274106 0.031083891 0.031042779 -0.014863377 -0.019157021 -0.024343541 0.005450004 0.002981597 0.051290549 -0.013752928 0.022626182 0.005830419 -0.004638925 0.005857187 -0.071504067 -0.065190528 -0.0224466 -0.039084642 0.094417461 -0.00126095 0.069302894 -0.072220749 0.091245452 0.044117019 0.021670726 0.01456608 0.045795591 0.044459871 0.018380056 0.000183601 -0.039270325 0.01199642 -0.043716821 0.000177412 -0.007365319 0.025378048 -0.017603774 -0.027993891 -0.025699605 0.065948915 -0.04580385 -0.011952339 0.02002752 -0.031575967 -0.002024077 0.029344582 -0.012645522 0.021016169 0.000945241 0.028230307 0.015533995 -0.009731695

sample_79 -3.102744964 0.495105139 2.627466957 0.840243393 0.233769908 -1.056616297 0.132533307 -0.723061512 -1.287824789 -1.405122523 1.664757344 0.294006346 0.415988597 -0.520015918 -0.724267799 -0.781856676 -0.495315092 1.026249131 0.327384792 0.002884764 -0.271986772 -0.36881316 0.547559896 0.99654791 0.300597108 0.531546909 -0.33979543 0.170963625 -0.48165015 0.101342274 0.16832257 -0.11376152 -0.129031456 -0.589301272 -0.318282955 0.004241524 -0.123181504 0.457592641 -0.483326809 -0.037354384 0.262952183 0.153473973 0.359468596 -0.123601611 -0.055159975 0.09108293 0.54946893 -0.094827323 -0.06179972 -0.015450461 0.104292835 -0.028742786 -0.19464932 0.142882181 0.152372455 0.305074329 0.127268899 -0.186341482 -0.477301015 0.025803332 0.090198958 -0.08590516 0.13564924 0.061380196 -0.181941243 0.186455199 -0.045288334 0.293450204 0.04107796 -0.016103347 -0.271670412 0.19280753 -0.131104183 0.256576827 -0.134421711 0.034732792 0.060607512 -0.081053856 0.058846764 0.008488437 0.021068569 0.181714395 -0.103504591 0.054073793 0.112065475 -0.023028909 -0.165775989 -0.137796169 -0.043721285 0.064860182 -0.127614544 0.032566534 0.192841595 -0.040423956 -0.073523141 0.125464776 -0.187676605 -0.05615125 -0.046119193 -0.02965873 0.165363942 0.004228886 0.150695654 0.021287803 -0.155334763 0.037877817 -0.038953537 0.147261726 0.010975662 -0.098070776 0.108981581 -0.065324306 0.004058637 -0.237575358 -0.008447126 -0.217038364 0.024193474 0.064239686 -0.10632235 -0.039853848 0.028017631 0.078175744 0.07533557 -0.019772031 -0.003267299 -0.136331699 -0.028253529 -0.005332723 -0.13415933 0.154241811 -0.037470628 -0.019876989 0.047883664 0.007701256 -0.178270393 -0.037680674 0.06437113 -0.079422452 0.062342274 -0.221438695 0.079314764 0.004313286 0.210622269 -0.05780093 -0.007918494 0.084756111 0.062027411 0.031894419 0.061975264 -0.041847798 -0.054118989 0.055644001 -0.002135506 -0.017787488 0.04505638 -0.01561367 -0.059227434 0.076645337 0.049035522 -0.050096522 -0.041530429 0.104734434 0.001952344 0.037120099 -0.012230492 0.022308496 -0.036512718 -0.012071618 -0.045172593 -0.002526141 0.046503964 -0.061385108 -0.068286851 -0.047630834 0.05740887 -0.050466888 0.055752939 0.050808927 0.071503644 0.012084032 0.041271985 0.070945337 -0.004401379 0.019109026 0.066268039 -0.054499601 -0.042190041 0.00290961 0.027180626 0.103927882 -0.068543347 0.020915976 -0.044381038 0.045981723 -0.025048003 -0.038989415 -0.081322895 0.132106079 -0.141189646 -0.029822339 0.014589457 0.032255209 -0.000922872 0.076936408 -0.083195938 -0.009267559 -0.121511972 0.019103362 0.007815126 0.022648044

sample_80 -6.641491745 -0.232013598 2.505481655 2.454908098 3.131856336 0.481308557 -1.769224811 -0.018945567 -0.21359517 1.035575098 -0.610721499 0.388938695 -0.065013931 -0.009319468 0.304049992 0.303325455 0.596266342 -0.264780787 0.061422257 -0.470310025 0.73412406 -0.517146298 0.806193416 -1.004315307 0.060182595 -0.577160164 0.979431879 -0.605552582 0.099168308 -0.044282168 -0.129427491 -0.204460777 -0.086893089 -0.196756497 0.146509395 -0.16834769 0.585202069 -0.160618681 0.120067886 0.160243794 0.494225979 -0.443049854 0.271647294 0.248993734 0.345196286 -0.355134854 0.197260699 -0.544065493 -0.069715842 -0.419579104 -0.13902957 -0.036781112 -0.260078375 0.030560701 -0.143471272 -0.19646575 0.146783363 -0.156094869 -0.111916264 0.064102252 -0.061623157 0.335941809 0.108797894 -0.118450027 0.045002401 -0.154984322 0.149205605 -0.133393368 0.076055322 -0.079834943 0.007865215 -0.039420342 -0.266309739 0.070771962 -0.043990244 -0.020138787 0.075395682 -0.01728753 0.027109541 0.056271165 0.144334924 -0.108876021 0.039186309 -0.158073221 -0.115321836 0.066961844 -0.111870475 -0.045836241 0.055275342 0.233012819 -0.015725077 -0.230823945 0.039336578 -0.028529573 -0.222422857 0.166409047 0.059789607 -0.042726409 -0.092583329 0.14183811 -0.115209627 0.087060475 0.09300356 0.046276895 -0.146772366 -0.01382027 0.024464426 -0.138398124 -0.160058908 0.057550948 0.106826797 0.082363248 -0.124446757 -0.046333882 -0.146854368 0.052042872 -0.112502731 0.086718863 -0.09831173 0.088541124 -0.049540844 0.004262022 0.199269132 -0.081327789 -0.10834037 0.068841879 0.001806024 -0.095537767 0.103482022 0.075895306 -0.007195973 -0.165800337 0.19490126 0.016274357 -0.160156802 0.031957916 -0.013942126 0.066427458 -0.119251723 -0.064559267 0.030411798 -0.014218515 0.087017714 0.019928903 0.096115264 0.062895769 0.009817744 -0.050028175 -0.018887487 0.008343375 -0.133502069 0.08562883 -0.074749547 0.041690787 -0.056976034 0.064072724 -0.006913285 0.002898475 -0.039984829 -0.036259143 -0.074950606 -0.037092041 0.083477467 0.086911869 -0.048787051 0.006679631 -0.036300849 -0.111784472 0.025310534 0.000379287 -0.056513623 0.109552045 -0.066093273 0.112840866 -0.047912228 0.028683304 0.060810353 -0.066503796 -0.103996312 -0.053173651 -0.024428986 0.0025533 -0.038881914 0.060547193 -0.034034966 0.0715297 0.041447974 0.037709626 0.027270401 -0.000559614 -0.020621043 0.067844825 0.062319874 -0.005698796 0.120189854 -0.011031788 0.005846333 -0.032179801 0.031709901 -0.054451384 -0.047567475 0.01111925 -0.073561969 -0.010716622 0.04333734 0.083354884 0.002096827 0.015817574 0.015027282 0.041627316

sample_81 -5.835962749 0.666139875 1.21238546 1.384542835 -0.129699459 -0.404215855 -1.447834577 0.108760035 -0.493929917 -0.312884769 1.341279216 -0.169123276 -0.25166058 -0.609978841 0.288328264 -0.262336124 0.31280933 0.931284656 0.045084331 0.24767543 -0.156913281 -0.756461334 -0.688866989 0.24106519 0.10035726 0.519619789 -1.017704259 -0.685548951 -0.35210811 -0.475344485 0.44452892 -0.755487129 -0.492047383 0.140381833 0.00493103 -0.60284934 0.133330734 0.141401031 -0.146062752 -0.146779234 0.052933538 0.197662647 0.325292408 0.017921452 0.348424757 -0.085097997 0.241779254 -0.016729315 -0.331638747 -0.15451065 0.110083076 -0.339890623 -0.101056017 0.044852783 -0.022021483 -0.20905114 0.033456243 -0.096635277 -0.018865303 -0.135887253 0.348440171 0.026503043 0.081961724 -0.167313744 -0.049131667 -0.212630096 0.015981275 0.075594962 0.109308241 -0.121300281 0.01429138 0.098656895 0.129815231 0.043720901 0.151540496 -0.024173958 0.066014777 0.366408897 -0.078528885 -0.046192069 -0.255400656 -0.112658327 -0.064457675 -0.416410813 -0.133108786 0.17617636 -0.025616284 0.10643521 -0.2591083 0.103581392 -0.201390776 0.222873688 0.126077123 0.153841161 0.13933556 0.180666744 0.000154454 -0.009946031 -0.08858073 -0.133343297 -0.037170605 -0.088294281 -0.202551023 0.100915774 0.057262824 -0.076692671 0.206353984 0.040912098 0.065386776 0.085025478 -0.029122438 0.009957619 -0.131194697 -0.081692914 -0.068964474 -0.046497899 0.002305223 0.154698399 0.160184649 0.139424074 -0.091191482 0.175993997 -0.017129124 0.010143968 -0.060406394 0.014932204 0.166716021 0.210201041 -0.010983819 0.062469236 0.072461633 0.008034372 -0.058449549 0.043989337 -0.165943799 0.014215766 0.102010387 0.044243395 -0.001714664 0.100447484 0.059992282 -0.042968209 -0.064588694 0.023493986 0.099886029 -0.159619929 0.022265504 0.023047261 0.059759635 -0.122472317 -0.178858101 -0.078728805 0.090158634 0.031582925 -0.054719657 -0.105485816 0.109246563 0.062412369 0.029191598 -0.01466373 0.198262232 0.041368379 -0.066385068 0.082067084 -0.019393035 -0.039369961 -0.113997558 -0.002575833 -0.002213025 -0.001422553 -0.109936432 -0.17653479 0.02445867 -0.069949951 0.000549247 -0.002309962 0.010250961 0.087828365 0.049653568 -0.060248891 0.1097566 -0.001767898 0.038892349 0.036987378 0.028316098 0.056003994 0.045573332 -0.106259773 0.100832173 -0.206477937 0.021055684 -0.038080278 0.004954925 0.073727603 0.020642924 0.031618315 -0.045989356 -0.037464248 0.081944511 -0.027596558 0.003215105 -0.094851263 -0.034344331 0.004490277 -0.07777832 -0.020475416 0.018333015 0.079687533 0.067367195 0.07085414

sample_82 1.008908872 -0.688665991 2.822178848 0.743312066 -1.614289293 -1.99715167 -1.062977549 -0.291918297 0.351343694 -2.003629159 0.174363522 -0.331448045 -0.578939997 0.535502915 -1.573731063 0.191324323 -0.508147727 0.05389249 -1.034117128 -0.075058529 -0.528516768 0.609113264 0.518785181 -0.343505205 -0.034896873 0.604196602 0.204038194 -0.085571668 -0.122316664 0.194598757 0.331876891 -0.044500008 -0.523202062 0.630941074 0.643852735 0.147193253 -0.086572324 -0.106131218 -0.606190824 0.384972054 -0.267956957 -0.367135434 0.17622426 -0.004366757 0.040186593 0.019307269 0.254778431 0.367342049 0.15552315 -0.107875675 -0.171710121 0.134337766 -0.095776759 -0.273539703 0.127803555 -0.475624536 -0.174221785 -0.199088482 0.110111777 -0.270865706 -0.086263982 -0.179408923 -0.133787972 0.055624947 0.409514143 0.131782418 0.043289866 0.131092628 0.020879281 0.045729095 0.11282411 0.000873431 -0.027374098 0.050738746 -0.143971462 0.384624583 0.045103595 -0.004050325 0.03720082 0.290657105 -0.209028625 -0.182139822 0.049980983 -0.041560072 0.1365892 0.056073192 0.07681224 -0.138654455 -0.013797811 -0.229783237 0.012825908 -0.08808792 0.170861721 0.020979635 -0.062904633 0.055761895 -0.216995064 -0.032160727 0.087811209 0.133757238 0.170536711 -0.026664693 0.178673182 0.014922231 -0.055040595 -0.136093263 0.183582857 0.018103009 -0.090877052 0.112798817 -0.025233387 -0.121338426 -0.086204668 -0.068585269 0.009436591 0.061831627 -0.030175951 -0.156686986 0.129927991 -0.135781785 0.015354518 0.019365964 -0.02227862 0.008487891 -0.04389993 -0.005649936 -0.047458173 -0.012615919 -0.07010654 -0.041959964 0.04192892 0.115546567 -0.010299133 -0.056959478 -0.082177545 0.081465339 -0.160010998 0.176327624 -0.037594067 0.129446164 0.017133188 0.071300778 0.089978106 -0.091822828 -0.02174114 0.056747801 -0.099907215 0.096818206 0.033324207 0.065192264 0.016916982 0.019624886 -0.007396569 0.030387529 0.033855163 -0.021279382 0.091633198 0.112868681 -0.001531682 -0.080805596 -0.125263213 0.05051473 0.015917999 -0.058770156 -0.095896029 -0.022924803 0.019224231 -0.088727366 0.034376348 -0.078891264 -0.005517385 -0.056399332 0.038098098 0.011183002 -0.02313328 -0.021780498 0.05003908 0.06367917 -0.044824242 0.164488233 -0.061519135 -0.06716004 0.070300709 -0.032487945 0.011160368 0.025236431 -0.027968435 0.083518512 0.03077519 0.087114823 0.121663578 0.019838055 0.004800194 0.016994684 0.015598138 0.145480943 0.017849223 0.098577811 0.03620357 -0.009649956 -0.091111075 -0.079954811 0.040353427 -0.02663782 0.047545883 0.021863603 0.044423673 0.029621436 -0.035238677 0.010444972

sample_83 -5.080766201 -0.653150678 1.380709713 -1.152740562 -1.437370762 -1.320410875 -0.789832004 -0.499989776 -0.651850476 0.002087313 1.453510184 -0.107118046 0.702460187 -1.222279668 -0.098844489 -0.473068358 -0.061391293 0.754488974 0.19702595 0.279018077 -0.817283389 -0.870396896 -0.390197485 0.01203548 0.26177023 0.473648843 -1.018167742 -0.285876361 -0.040909354 -0.263627858 0.569335242 -0.842898707 -0.447663819 -0.057457131 -0.122067822 -0.29916703 0.164740952 0.011632147 -0.165091735 -0.243859266 -0.178321659 0.041706014 0.198925203 -0.172816271 0.186691556 -0.047891943 0.211523549 0.323424268 -0.143906654 -0.159538445 0.289545456 -0.328903482 -0.160368386 0.038362681 0.115766391 -0.013054741 -0.066025911 0.276855716 0.43238349 0.049763694 0.290046136 0.12934255 0.313221895 -0.090572046 -0.036487265 -0.111873099 0.192018217 -0.04624782 -0.021773731 -0.298232008 0.025050845 0.136985649 -0.073691653 0.244655031 0.159856908 -0.083670014 0.092702818 0.33659788 0.014316938 -0.301389645 -0.125804637 -0.110662724 -0.061015341 -0.205850386 -0.176870791 -0.029322654 -0.187257066 -0.044686671 -0.089154878 0.083761034 -0.128909235 0.192612667 0.101208917 0.090827211 0.206379189 -0.017857817 0.075981338 -0.18220099 0.012946492 -0.043275474 -0.085999409 -0.099452677 -0.039082685 -0.012201763 -0.071161376 0.039056012 0.172170472 0.091722269 0.071708526 0.144551093 -0.014864004 -0.058602173 -0.050233858 -0.028982935 -0.083199548 -0.039571861 -0.0393361 0.172637885 0.107748118 -0.000156734 0.039674981 0.236508747 -0.021051884 0.126518502 -0.00783779 -0.038242632 0.069452788 0.17402922 -0.056806322 0.014268891 -0.009885239 0.005546584 -0.094600714 -0.021285324 -0.182865085 -0.020016462 0.014953589 -0.037947293 -0.036449316 -0.131623228 0.05084441 -0.067408125 -0.066113919 -0.023462814 -0.030422548 -0.071674106 -0.00946964 0.052047701 0.009964615 -0.034501263 8.10E-05 -0.143481744 0.026670409 -0.019695538 -0.014902972 -0.014844723 0.025704636 -0.04717167 0.040031892 -0.038276117 0.047614723 0.040862506 0.007948297 0.086571684 0.00607232 -0.053508661 -0.085340803 -0.016670131 0.026797421 -0.023471733 -0.105373175 -0.074731058 -0.054041184 -0.056181158 0.025121611 0.04995902 -0.044918676 0.060017077 0.054173118 -0.024328752 0.132821604 0.006936083 0.039521286 0.022497135 0.006775333 -0.084054027 0.03892089 -0.017625894 0.05780081 -0.102613471 -0.141960524 -0.048101058 -0.053999954 0.028817304 0.059308057 -0.034115927 -0.008055048 -0.043943711 0.022978024 -0.056336538 -0.070109317 -0.044244005 -0.038695293 0.019114953 -0.052926853 -0.005007042 -0.038891075 0.008419395 0.031319278 0.030120825

sample_84 -5.683256167 -0.030577839 1.62725367 1.162625899 0.558370346 -1.505322569 -0.994658508 -1.039991228 -0.591822832 -0.176390619 0.913757866 0.160369669 0.364138957 -1.381748934 0.167820588 -0.598334199 0.028639754 0.21841756 -0.251972079 0.005630251 -0.656186653 -0.019063889 -0.077652156 0.509948791 -0.159246197 -0.659479142 -0.22738247 -0.332689179 -0.435096651 0.00999744 0.188374866 -0.330700291 -0.023380995 0.03224734 -0.355255097 0.104239615 -0.264003194 0.041989883 -0.123063557 -0.10829539 -0.124450935 0.025721339 0.033357626 -0.21273884 -0.065297753 0.276538285 0.162934734 0.047424708 -0.246160934 -0.187224555 0.143265089 -0.12593576 0.065885958 -0.008139976 0.014753577 0.146162092 0.021978936 0.100053899 0.000943066 -0.123325967 -0.225761368 0.210002224 0.324031078 -0.091925842 0.115841542 0.0465168 0.358888149 0.16444315 0.08969127 -0.152887997 -0.059680953 -0.066668025 0.119881418 -0.01805524 0.046895543 0.008122714 0.216258593 0.061103497 -0.085062791 0.07891634 -0.098692042 -0.121383732 0.077472183 0.031046096 -0.060103415 -0.037654633 -0.048082912 -0.06705426 -0.024371743 0.1356706 -0.118786272 0.143967215 0.093285897 0.026212538 0.088874807 -0.016785392 -0.026973788 -0.020345353 0.008414598 0.095816665 -0.045878008 -0.087628585 0.016972194 -0.034175123 0.039545301 0.008562199 0.101152842 0.024640532 0.039601622 0.100320441 0.033370522 -0.11134565 -0.072805257 -0.026736266 -0.053025291 -0.07971237 0.027784077 -0.007347082 -0.041159416 0.081087363 0.046871666 -0.02699961 0.099573012 -0.074106417 0.029892138 -0.092717969 0.05524171 0.017438179 -0.007348128 0.00755527 -0.054039119 0.000929512 0.005180395 -0.071616003 -0.089322839 -0.00457982 0.108989094 0.065140225 -0.040346013 -0.079763775 -0.005992755 -0.00393368 0.034262485 -0.010567199 0.011080814 0.000546792 -0.015234409 -0.083641306 0.088167423 0.01430762 -0.05844492 -0.021895234 0.031028991 0.065785491 0.034280679 -0.002398324 0.06440057 -0.029984064 -0.038947187 -0.047849668 0.002837721 0.047801263 -0.082394253 0.048221687 0.052645258 -0.051843331 -0.004027294 0.028890175 -0.02153681 0.007293156 -0.00056035 0.008259553 -0.070996881 0.004497681 0.02928584 -0.03487368 -0.009264896 -0.013136522 -0.011068696 -0.035868037 0.079461574 0.004566688 0.012668379 -0.006548047 0.018334689 0.032830812 0.024552117 -0.022417783 0.004979685 -0.103825322 -0.014695651 -0.034774024 -0.032735163 -0.051539818 -0.050940031 0.026750219 -0.006555123 -0.046412881 -0.00810759 -0.02588232 -0.032420618 -0.04203205 0.000768649 0.040725822 0.004363052 -0.00946626 0.041857996 0.012507881 0.010997425 0.023038891

sample_85 1.994200637 -3.981047487 1.158995906 -0.10055916 -3.205521929 -2.773831792 0.184041468 -1.09251203 0.588589596 0.065303747 1.770272721 -0.452650613 -0.010841306 0.007675021 0.101450005 -0.860296195 -0.258260356 -0.022004058 -0.115621809 -0.519313588 -0.323806455 -0.144969374 -0.419869237 -0.045148698 -0.234534424 -0.311812182 0.05615227 -0.194264908 -0.052938748 0.213391831 -0.025940533 -0.256852071 0.034776527 -0.037378561 0.376625255 -0.347752884 -0.189983587 -0.042664392 -0.060816283 0.271139071 -0.060833246 -0.298519407 -0.066484192 -0.373962236 -0.066689304 -0.329688114 0.104128117 0.133979339 0.176963354 -0.120028327 -0.159595411 -0.075235799 0.104309031 -0.259687866 0.183435586 -0.069033111 0.067794315 -0.343647623 -0.017465143 0.14883731 -0.166325493 -0.144927105 0.052814113 -0.204208754 0.053843551 0.288496092 -0.091044506 -0.040602568 0.130891093 -0.158570293 0.024242611 0.145792334 0.055487747 0.062929268 0.238732453 -0.103724901 0.015986198 0.177133398 0.162050468 -0.076665051 -0.014141436 -0.002743885 0.327861428 -0.127572794 0.232469164 -0.021303767 -0.214680556 0.143636491 0.021723028 -0.247652741 0.19819906 -0.209369889 0.112337397 -0.101649561 -0.063202183 -0.113389623 0.069289773 -0.056430844 -0.039860645 -0.084976773 -0.093887276 -0.009277739 -0.030198972 -0.029891782 -0.189225925 0.117499707 -0.036741646 -0.017519783 0.06179253 0.126521907 0.038596932 0.062232443 -0.032664275 -0.061187893 -0.016958478 -0.024724828 0.009698597 0.077470099 0.069127056 -0.116803579 0.106352617 0.085633823 -0.012152064 0.085569822 0.031360417 -0.060223465 -0.015348937 -0.109898212 -0.017085868 -0.041370903 0.001348607 -0.010299246 -0.025151022 0.059453162 -0.047016018 -0.038385457 -0.068396176 0.064698862 0.001670263 0.065008277 -0.084666212 0.039747994 0.06987638 0.009928671 -0.045502533 0.042816349 -0.057942852 -0.053997543 -0.014442263 0.005515747 0.173880581 0.032194264 -0.003872344 -0.112193226 0.009070885 -0.041599195 -0.036243415 0.003935666 -0.070605679 -0.018892103 -0.134618975 -0.217975498 -0.011820081 0.011647492 0.039795521 -0.055257624 -0.023811577 -0.010758239 0.055882831 0.067060326 -0.023865638 -0.049198533 -0.041348039 0.018170751 0.06995081 -0.013176192 -0.010033501 -0.055466439 0.014997033 -0.0554202 0.122979121 -0.026392054 0.038208609 0.019473671 -0.000445852 -0.006245361 0.029814567 -0.060962548 0.025490789 -0.066919598 0.004352495 0.030761886 0.001778848 -0.040874905 -0.045446493 0.074687779 -0.042644906 -0.062506271 0.012774639 -0.060748731 -0.150395548 -0.038144513 0.013137963 0.008792765 0.016628643 -0.049850548 0.034129944 -0.026975273 -0.006124821 -0.021450378

sample_86 0.446919875 9.142064635 5.736753066 0.054738984 -3.582679438 3.389454411 2.07686239 -1.109390179 -1.24446416 1.765277731 0.464779697 0.105517397 0.555945499 0.167440118 -0.489255376 -0.628691424 0.624073266 -0.238042673 -0.339976614 0.007206759 -0.352028373 0.276981517 -0.957702041 -0.200290478 0.298392168 -0.532556145 -0.28877448 0.444753374 -0.736839827 -0.185216916 0.065222702 0.035658397 0.053529088 -0.063845064 -0.500812249 0.403366065 0.456836249 0.1121177 -0.242101437 -0.274605923 0.123527528 0.237142794 -0.16683123 -0.585182923 -0.109981531 0.519337464 0.492822035 -0.565090245 0.08064328 0.341907331 0.314452927 0.093229893 0.304233298 0.036838241 0.212734208 -0.08032537 0.299383824 0.150617383 -0.425756985 -0.107160805 -0.040541984 0.03151238 -0.435534242 0.033322689 -0.353176895 -0.072590563 0.125216337 -0.017898394 -0.006012441 0.009978487 -0.008840574 -0.36504779 -0.268851584 0.245272967 0.129338209 -0.22685749 -0.332991574 0.134984613 0.343042979 -0.003093271 0.08487169 0.180602394 -0.121945525 0.012499656 -0.108834497 0.035908942 -0.101534915 -0.234331967 0.240318293 -0.226974605 0.046339226 0.186681988 0.203841552 -0.090392705 0.056400607 0.066710826 -0.061805303 -0.05171139 -0.130751114 0.165647519 0.142558313 0.185685291 -0.215080107 -0.016284014 0.046667648 0.096004332 -0.062270902 -0.077279486 0.063368459 0.078635145 -0.204938896 -0.064961525 -0.077094232 -0.03766915 -0.080526536 -0.056058923 0.047018151 0.093248563 0.122101088 0.056756114 0.015293238 0.15329424 -0.119903614 0.101935732 0.145202985 -0.04853826 0.060570324 -0.001023947 -0.144774633 0.195997794 -0.029470084 -0.163911771 -0.006496328 0.027829876 0.023093498 0.052990533 -0.099268183 0.02765647 0.148112733 -0.10402033 -0.009921387 -0.047236054 0.109966761 -0.072747618 0.107565689 0.09360574 -0.141239336 0.059678392 -0.015367833 -0.007893011 0.015286081 0.080332352 0.0328113 -0.121924038 0.014986264 -0.147583078 0.099396458 0.019450637 0.046702051 0.010795211 -0.050440745 -0.00223978 0.019114545 -0.047200934 -0.027565966 0.088036696 -0.166714824 -0.024449824 -0.097193545 -0.050530606 -0.07981467 -0.066174365 0.003474993 -0.153125862 0.038568319 0.055986029 -0.035958297 0.006624397 0.009851172 0.091753557 -0.033070804 -0.038464105 0.098857946 -0.08396811 0.068168732 -0.058112411 0.105694275 -0.000514509 0.035476715 -0.007241122 -0.020139291 -0.030699771 0.079716703 -0.027379951 0.073091897 0.045172698 -0.042700144 0.088132067 0.006033982 -0.015336551 0.041288151 0.004532911 -0.087396077 -0.037949168 0.129148022 0.016659444 0.144504575 -0.060207787 -0.007906594 -0.035538

sample_87 2.73515249 1.219190916 2.651481709 -2.68164308 1.176032006 -1.750999225 -0.303431176 0.271867626 -0.15469546 -1.949663937 0.761288733 -1.403488353 -0.636917022 -0.838233557 -0.323077924 -1.449654002 -0.76230948 0.76063975 1.049108914 -0.044422435 -0.509994701 0.168108328 0.004964342 0.056298559 0.465727997 0.388766506 -0.382200154 -0.518631382 -0.063616301 -0.146021107 0.205096237 0.460671898 -0.156865925 0.429255966 0.134907467 -0.051330888 0.09613375 0.341275546 -0.059497252 -0.341864394 -0.171690048 -0.061094033 0.490360248 0.142698693 -0.416820916 -0.510082666 0.206014462 -0.382270568 -0.058915003 -0.132065595 -0.0878249 0.110933982 -0.294472199 0.061760642 -0.208003585 -0.087724434 -0.078606016 0.09349799 -0.050401641 -0.210707364 0.195637967 -0.09733999 0.126149674 0.286274763 0.034769594 0.117765019 -0.193733604 -0.034682863 -0.039352379 -0.082498322 -0.042032629 -0.022893505 -0.036634273 -0.073414493 0.17389942 0.304442429 0.289365538 0.41321801 -0.123306523 -0.052741495 -0.077992011 -0.060418627 -0.229895752 0.098392433 0.120854539 -0.079129579 -0.174934103 0.037937372 0.061482457 -0.041662153 -0.139258241 -0.077327456 0.004275239 -0.034355966 -0.28404717 -0.087888216 0.333373902 0.099908753 -0.308469496 0.202720598 0.099209052 0.15511086 0.078464871 0.161877243 0.088497832 -0.152193963 0.035418733 0.242825919 -0.142300935 -0.017456422 -0.015985106 0.252840869 -0.035239268 0.019712224 0.105440216 -0.028916489 -0.054142334 0.101026494 0.070892635 0.027320251 -0.028039718 0.015443487 -0.119897172 0.126695128 0.064540303 0.211663999 -0.138464419 -0.062119003 -0.112070713 0.095108341 0.00798507 0.010310549 0.056907432 -0.27725934 0.113361029 0.237439238 0.079267568 0.037847333 -0.074211151 -0.266118571 0.127002094 0.001027894 -0.031338855 0.052801868 -0.027829196 -0.11432045 -0.008523266 0.049345975 0.017185988 -0.066024562 0.046699364 -0.096671228 0.079445778 0.064407692 0.005032628 -0.062404906 0.097591281 -0.05400853 0.019646432 0.032107398 -0.032128881 -0.127831633 -0.105177871 0.075982231 -0.127005093 0.039856971 0.038518943 -0.012083014 -0.01619417 0.020571685 -0.052008073 0.021138189 -0.005007526 0.102866363 0.00158409 -0.081326358 0.010633995 0.106913246 -0.00634216 0.001858166 0.140123944 0.016495343 0.085600569 0.02221598 -0.100444188 0.128946282 0.063486325 -0.012821145 -0.013553205 0.054747591 -0.052498637 -0.034734956 -0.017365578 0.017219722 0.09568968 -0.19050482 -0.053175712 -0.011382274 0.094738672 -0.022482476 0.058745888 -0.078152566 -0.007305934 0.093859454 -0.139844034 0.055588253 0.012655779 0.008245687 0.046252226 -0.010512007

sample_88 -7.523165358 1.613432932 0.337466068 2.683384584 2.104449942 -1.395547969 -1.26275733 -0.442413075 0.617704213 0.63704829 0.327681902 0.802675965 0.763460665 -0.12999974 0.058813219 -0.85020007 0.20819403 -0.375442725 0.630429556 -0.192898015 -0.477501748 0.686405448 -0.175109948 0.670073441 -0.277623271 -0.309474553 -0.606629408 -0.412733278 -0.093350581 -0.119041944 -0.139193559 0.175188906 0.423091668 0.225892107 0.014451676 -0.003048124 -0.258456797 -0.317907013 0.132725358 -0.05094121 -0.494033053 -0.095497637 -0.116046765 -0.027328567 -0.050106796 -0.088032428 -0.134618193 -0.074653617 -0.079566111 0.066477165 -0.166758747 0.061509148 0.287990797 0.080140668 0.055774846 0.048999334 0.110218723 0.14505669 -0.031732681 -0.03052457 0.064608728 -0.308180373 0.168360863 0.027051712 -0.039788734 0.121620274 0.064941068 0.133765527 -0.162246959 -0.261692517 -0.022748697 0.363483211 0.051318421 -0.111419146 -0.226992547 -0.129062035 0.101034146 0.11990618 -0.098371798 -0.031077794 -0.043389827 -0.077411086 0.074717125 -0.098954882 -0.005941075 -0.013967172 -0.006409099 0.163507261 0.058319581 0.048511695 0.033633757 0.076131127 -0.038637301 -0.083887739 0.062047015 0.036699516 -0.032299492 -0.003277568 -0.031339012 0.030220104 0.019286055 -0.056161137 0.006978919 -0.089480942 -0.018610232 -0.056740977 -0.002202784 -0.005455465 0.037374376 0.044909765 -0.13361341 0.126781119 0.005449674 -0.037962206 -0.0138207 -0.056770674 -0.042537503 -0.11565562 0.02410103 0.051541711 0.123669369 -0.031520809 -0.101336365 -0.013712825 -0.078812355 -0.083932849 0.031882367 0.003149642 -0.016465945 -0.061861795 0.009398174 -0.04926784 -0.079063528 -0.031107676 0.005283283 -0.02081359 0.052240247 -0.084559688 0.027874364 -0.00295887 0.055622872 0.005898956 0.00376668 -0.07354843 -0.041926604 -0.12181494 -0.00389974 0.029974711 0.014513004 -0.022952051 0.010032267 -0.000265387 0.064228742 -0.134551054 0.050050845 -0.082254059 0.05798842 -0.034278872 0.030699858 -0.02454032 0.085295306 0.022414474 -0.015786785 0.021218839 0.035266942 -0.00824819 -0.041408024 0.028911309 0.020231168 0.063348632 -0.070359472 -0.034789 0.048125804 0.057381049 0.021034593 0.013856775 0.057288519 0.093463611 0.016072657 -0.007569671 -0.00114482 -0.055380954 -0.023991951 -0.014751132 0.038005929 -0.010247953 0.005451638 -0.070993809 0.0647617 -0.066225944 0.063837912 0.00877992 0.037308227 0.027842859 0.012292442 0.004361848 -0.007106051 0.006053919 -0.01066762 0.007276374 -0.04736882 0.000442699 0.006804726 -0.052337563 0.004018742 -0.051084864 -0.019360146 0.038440896 -0.049437547 -0.030761255

sample_89 -6.505504431 -1.435046124 1.025981415 1.317780812 -1.886663649 -0.056195871 -2.306949343 0.774465675 0.341048158 -1.224671245 -0.199331209 -0.664967923 1.336709173 -0.964078118 -0.851639085 -0.69736855 0.852320161 -0.090848865 -0.296783983 -0.292070255 0.307439472 -0.260575617 -0.305164059 0.12404068 -0.246847058 -0.277519005 -0.80848999 -0.306154338 0.173383154 -0.019461514 0.704865438 -0.907960159 -0.38435267 0.014254587 0.216254151 -0.292748001 -0.756864912 0.262130638 -0.238041294 -0.211688531 0.119762057 0.353164575 0.327571938 0.552307846 -0.244782395 0.032240119 0.245883185 0.575576646 0.336195537 0.12413282 0.296521785 -0.186946796 0.262887655 -0.260906962 -0.214029779 -0.106386562 0.244437368 -0.083149417 0.242158015 0.135841177 0.183155955 -0.231135971 0.301448319 -0.068863193 -0.072463005 -0.14211495 0.126771066 -0.285026399 0.055901115 0.055995832 0.200858179 0.139422169 0.187599298 0.241810326 0.039400707 -0.282387596 -0.013406952 0.077328849 -0.024703517 -0.037910114 -0.068280492 0.08559446 0.261287152 0.030602508 -0.15729506 -0.049100686 -0.024459929 0.10765724 -0.322232432 -0.055363455 -0.215745083 0.20469835 0.063939923 -0.039629403 0.292069168 -0.061770122 -0.213634929 0.111826197 0.141990418 0.003857987 -0.037069416 -0.03555255 -0.403867127 0.108738053 -0.157588176 -0.256718951 0.168688067 -0.007216568 0.089876533 -0.026385244 -0.123684374 -0.040596569 -0.013560595 -0.108921037 0.036480529 -0.134709011 0.06517658 0.168383921 -0.005356679 0.101886666 0.211675658 0.048510506 -0.058461614 0.089145251 0.191309201 -0.008111595 -0.157618387 0.086819229 -0.038910981 0.053558929 0.005495331 0.076930278 -0.127753553 0.050619964 -0.081629724 -0.02446964 -0.108658557 -0.00622042 -0.052626626 0.02121235 -0.038542822 -0.13100421 -0.041129899 -0.071486742 -0.061499455 -0.119220581 0.018894601 0.075259764 0.089891603 -0.091050583 -0.114081842 -0.134966323 0.089081402 -0.175082126 -0.054879279 0.130576436 0.030032331 0.036814836 -0.038225917 -0.158069652 0.109514026 -0.017479534 0.06972088 -0.035221863 0.09157417 -0.043482596 -0.116603437 -0.053323581 0.059390557 -0.085004713 -0.043471423 0.015468823 0.062124466 -0.059260727 0.048446394 -0.09071994 0.070094845 0.019921348 0.132570805 0.002082156 0.046201822 -0.007658837 -0.064259055 -0.007588728 -0.096544598 0.006239358 -0.020303524 0.059564407 0.050686385 0.042241583 0.077744873 -0.040786033 0.074977424 0.109669757 0.051836696 0.031128133 0.003792698 0.048050322 -0.017774502 0.038934487 0.017880107 0.091578384 -0.083814627 0.05839562 -0.010298337 -0.074401935 0.058748726 -0.07909155 0.06274407 0.016909678

sample_90 -5.742357212 1.998440977 -1.318981012 -1.991974879 1.102043862 -1.306278887 0.593360017 0.041082228 -1.405919607 0.671776866 -0.637724213 0.402588616 0.084835627 -0.05095495 0.387845211 -0.065698715 -0.417265631 0.691885571 -0.342515955 0.032667027 0.412867577 -0.054121101 -0.332457054 0.609453947 0.097483791 0.146711749 0.272050175 -0.139374526 -0.276440244 0.079890813 0.137067509 -0.112040264 -0.431156158 0.290767925 0.131373694 -0.258700898 0.12901652 0.225591144 -0.0237937 -0.106363109 -0.11095442 0.121433094 0.277053505 -0.101534232 0.389749374 0.060494293 -0.048423698 -0.161378786 0.251571786 0.105242493 -0.168004881 -0.178947469 0.127409467 0.081256913 0.109074709 0.120610293 0.071375357 -0.057491363 -0.076122475 0.154935941 -0.173788842 0.226371326 -0.092879941 0.072440343 0.280800581 0.055471435 -0.002369203 0.078695013 0.155023476 -0.113164244 0.13088496 -0.147160132 -0.028363007 0.176104035 0.253221725 -0.05354359 -0.054848651 -0.093370372 0.054736005 0.071805683 0.109294914 0.150323881 0.025405246 -0.167293283 -0.0483727 -0.022744159 0.092158968 0.051856398 0.102664253 0.127856796 -0.020550089 -0.087511694 0.042048479 -0.032351176 -0.087992576 -0.142996261 -0.042050412 0.04354383 -0.002018774 0.182766843 -0.030102128 -0.089180131 -0.075010298 0.021283661 -0.048095233 -0.10590891 0.048284117 0.130138327 -0.102228334 -0.021818617 -0.014133713 0.008529568 -0.022652277 -0.048369506 -0.078364581 -0.011792975 0.102814564 0.077829566 -0.096885488 0.125820395 0.028689657 0.043510647 -0.053556189 -0.002929835 -0.045113202 0.076009496 -0.009045053 -0.068844345 0.005018651 0.069881861 0.064348481 0.048701124 -0.03565031 0.007840265 -0.08343836 -0.040700381 -0.005804382 0.074450502 -0.024843689 0.005454402 0.027654746 -0.083936595 -0.011690599 0.087314937 0.069696721 0.090696696 0.04290211 -0.058498272 0.002417996 -0.042752635 -0.079683966 0.061067546 0.103975022 -0.017588663 0.021592254 0.006165513 -0.031486627 0.070088848 -0.068267856 0.066957232 0.029540438 0.096378427 -0.010658802 0.004155096 -0.032173291 -0.053538921 0.068237697 -0.040791848 0.014855186 -0.024262869 0.022518494 0.02339525 0.061490527 0.109546095 -0.014690181 0.082358077 -0.037836734 0.013676194 0.010364652 -0.015025194 0.033826536 -0.009272547 0.001826879 0.036664851 -0.004430925 0.066238395 -0.026028427 -0.086430824 0.086937501 -0.021385598 -0.024643141 -0.009448 -0.001042804 -0.028607954 -0.002545574 0.033034575 0.022689516 0.069601816 0.024715195 -0.061873576 0.043893326 -0.013828693 0.042565595 -0.050444036 -0.030849182 0.052479797 -0.010991527 -0.032377324 0.00310875 -0.027634834

sample_91 -5.92288381 0.395964423 -0.93030555 1.011211435 0.922183242 -0.253836649 0.357490679 -0.037887871 0.156205402 0.209292161 -0.189763882 0.267963796 0.670358877 -0.016792363 0.381775576 0.021080723 -0.216324905 -0.459564946 0.266494808 -0.173432903 -0.220656601 0.476815877 -0.195085158 0.583439912 -0.226950188 -0.373358 -0.509748307 -0.036689444 0.06613109 0.068621098 -0.410585257 0.447686803 -0.093412734 -0.031693909 0.088738243 -0.210203607 -0.134758473 0.036936468 0.090928375 -0.432338612 -0.518628134 0.432545347 0.015279302 -0.258410901 0.06784047 0.233729812 -0.105271864 -0.109451619 -0.204490508 -0.074189922 -0.103154377 0.090830092 0.334465355 0.049875718 -0.03716768 0.083321947 -0.040815239 0.0403502 0.235101564 -0.110342555 0.207252372 -0.273062922 0.076366168 0.174525974 -0.230660519 0.032650934 0.181109957 0.102186421 -0.33135569 -0.047274843 0.211604317 0.040789302 0.079459168 -0.048927033 0.108485793 0.084381072 -0.022808899 0.021244706 0.013497951 -0.0870109 0.01169559 -0.034490028 -0.160917243 -0.028087578 0.015287573 -0.11352175 -0.025371103 -0.011127775 -0.031204981 -0.084493035 0.008301493 -0.053503648 0.037097556 -0.131272936 0.041972827 0.03583955 0.05200706 -0.063784772 0.003006146 0.002104754 -0.024294637 0.028236301 0.145677943 0.004833837 -0.01523381 0.011040997 -0.029102976 0.007997739 -0.050091997 -0.04181025 -0.006693693 -0.103153788 0.080447301 0.10744315 0.011364388 0.029773496 -0.053981244 -0.086352472 -0.039404297 0.063805046 0.008749641 -0.020959369 -0.05843804 0.072133465 0.032925147 0.002540122 0.06252152 -0.001021966 -0.077324656 -0.01985505 -0.017239415 0.066572158 -0.058683874 -0.064640298 -0.025833854 -0.028172305 0.058648525 -0.059524812 -0.065508353 -0.001573145 -0.09617729 0.000126209 0.055226662 -0.050230656 0.040825251 -0.053275073 0.028265026 0.082260232 0.036316581 -0.044404011 0.029535079 -0.050394993 0.005545129 0.014236525 -0.053780119 0.043267858 -0.057035749 -0.044678955 -0.034499971 -0.028110953 -0.023674368 0.030740395 0.014026828 0.048479033 -0.006530658 -0.004825337 -0.031763593 0.068640947 0.0769683 0.029641101 0.053085503 -0.040896309 0.012612217 -0.005419628 -0.078920012 0.016565587 0.029668251 0.10660337 0.032183351 0.024548156 0.034891429 0.026793167 0.011592225 -0.027730206 0.035161049 -0.01085428 -0.024584985 -0.02122758 0.074243449 0.011884897 -0.003006859 0.019493457 -0.015288501 -0.03662941 -0.003169931 -0.010839662 0.013939645 -0.029861462 -0.017266934 0.021136287 -0.008965947 0.046628315 -0.013071211 0.012801107 0.038780082 0.033105959 0.007726964 -0.008099847 -0.007945511 -0.015043699

sample_92 -0.088731687 -2.834037891 1.135306743 -0.77955278 -0.441043046 -1.077727731 -0.244997141 -0.008105015 -1.161183935 0.434734298 -0.516811345 -0.499743059 -1.570826098 -0.464726965 0.404739099 0.875021372 0.47622402 0.286887263 0.106376679 -0.094063821 -0.010750775 -0.054102094 0.18747554 -0.312583351 -0.706174836 -0.519494733 0.389038336 0.311204929 -0.60165202 0.041381654 0.167972656 0.118766371 -0.104048253 -0.063431718 -0.004146863 0.300546626 0.062384248 -0.052835051 -0.013094804 0.121634057 0.272448094 -0.1922773 0.330877005 -0.253362167 0.272869038 0.050585205 -0.029167906 -0.017348397 -0.019168786 -0.066796268 -0.06668854 0.031775143 -0.030605264 0.044062151 0.152710448 -0.127087963 0.035730487 -0.005786168 0.252598846 0.241628775 -0.087211778 -0.0689089 0.032931676 -0.01826335 -0.036241965 -0.202357645 -0.101877899 0.019169306 0.037828822 -0.071211389 0.006509202 0.156406949 0.131879347 0.049914527 -0.171081067 -0.061726838 -0.09760928 -0.096007637 -0.005564039 -0.016869715 -0.053409396 -0.015389063 0.141877458 -0.046144065 -0.106613636 0.014364424 0.039047005 0.105300373 -0.070282751 -0.101059567 0.025560967 -0.119139941 0.027975607 0.037611968 0.082330587 0.088806083 -0.078605579 -0.009750997 0.053602396 -0.001222433 0.038463919 -0.021140473 0.013977716 -0.010433682 0.010953669 -0.030649266 -0.079351356 0.036713607 0.130993701 0.065505012 -0.031460866 0.021680938 -0.016381411 0.068353404 -0.084610646 0.057896282 -0.052563591 -0.023166028 -0.078928545 -0.091140766 0.03239757 -0.052113859 -0.012040545 -0.059334008 -0.05794119 -0.03121331 0.096691171 0.104609772 0.027944442 -0.0752111 -0.023097143 -0.052236736 0.013267986 0.059563069 0.089619025 -0.051878155 -0.011677425 0.058393627 0.072281074 0.048410747 -0.061057651 0.0049306 0.106973535 -0.074132761 0.008956369 -0.027079501 -0.031776737 -0.054827174 0.073163543 -0.025946615 0.02659724 -0.078456325 0.010154728 0.039028515 -0.0249016 0.041959173 0.051379955 -0.005049624 0.087768447 -0.035278646 0.012042882 0.008105315 -0.020998551 0.023011567 0.059010962 0.020786251 -0.033050574 0.013542244 0.050916709 0.033788321 0.03095824 -0.036586402 -0.006859728 0.043726194 -0.004707977 -0.028240313 0.06799247 0.004311736 0.065302987 0.013902221 -0.038121925 -0.033954218 0.052044253 -0.028824793 -0.024904615 0.022927682 0.047957248 0.057587634 -0.029455705 -0.012769452 -0.017097713 -0.001460781 0.029234944 0.00986681 0.000700101 -0.024454543 0.082034481 0.01137664 -0.006882421 0.069539701 -0.007837175 0.005772072 0.03119264 0.005298255 0.017955572 0.008933707 -0.031077215 0.048716392 -0.048819989 -0.038919321

sample_93 -7.497050475 0.66052162 1.078333601 4.061513018 3.109284492 0.122015161 -0.55576936 -0.832704279 0.356653796 0.467538482 0.921119657 0.829906321 -0.301668158 1.015668968 -0.053662431 -0.221924022 0.387438028 -0.224028991 0.797299779 -0.487751972 0.3975284 0.808840039 0.166013028 0.074999782 -0.090106356 0.168114199 0.377620974 0.355056232 -0.116625336 0.198562707 -0.015044348 -0.135484352 0.033148614 -0.214697241 -0.073686094 -0.297630915 -0.099321596 -0.409651822 0.174091627 -0.203193399 0.369493984 0.065513649 0.343662963 -0.019056808 0.141358969 -0.101343816 -0.558823191 -0.01051283 0.035688023 0.085803758 -0.080797584 0.076328652 -0.141566102 0.064849046 0.042574005 0.19191833 -0.247499552 0.008024862 -0.153157661 -0.286625901 -0.093293121 0.152371739 0.089937171 0.000581744 0.019033866 0.13023284 0.161278947 -0.027831311 0.155805013 -0.50538207 0.055348811 0.056898578 -0.030816423 -0.156250966 -0.082921901 -0.079221939 -0.17026708 0.198332804 0.017736965 -0.004160011 0.089989315 0.040842516 0.059008443 -0.125792453 0.019606079 -0.066407148 -0.265943192 0.00772518 -0.101882532 0.12275231 -0.09036837 -0.003358895 0.150784586 0.133991795 0.060955264 -0.151890944 -0.020691307 0.170648366 -0.150573492 0.011440545 -0.044712104 0.050171183 0.156439864 0.10695159 -0.201340263 0.108596146 0.057309186 0.029449274 0.063206202 0.109802912 0.169129387 -0.010632834 -0.020090969 0.046704512 -0.098971111 -0.039086041 -0.101262442 -0.011430557 -0.076568032 0.130539212 0.116953222 0.00176772 -0.05036456 -0.030999872 0.054290704 0.037393304 -0.010562001 -0.054135978 -0.151508847 0.141474294 -0.069878565 0.068680307 -0.179762946 -0.015141642 -0.086790558 0.080303938 0.071501511 -0.061542427 -0.136960134 -0.027362017 -0.017708758 0.055035594 -0.017734409 0.132160788 0.013385372 -0.017758105 0.096397098 -0.033021237 0.016617372 -0.153533096 0.001920536 0.080365 -0.011689984 0.054281436 -0.077191552 0.131478542 -0.032602675 0.006602658 -0.059017686 -0.005002576 0.009866265 -0.040483698 -0.078974963 0.08393197 -0.071410194 0.034045384 -0.056766694 -0.001001856 -0.014706416 0.070791167 0.033233717 -0.005855709 -0.062509458 0.028342041 0.012682673 0.023504393 0.039619862 -0.00171061 0.072784747 -0.034748393 0.04063671 0.030010384 -0.014968962 0.023296347 0.013117597 0.04689811 -0.005235727 -0.002142855 0.040518242 0.039530805 -0.044546205 0.014148618 0.041581846 0.153243188 -0.063647597 0.019352202 0.105871147 0.01068352 -0.020555902 -0.070973484 -0.031648238 -0.046709326 0.036645273 0.002548011 -0.053221302 0.032049166 0.032981804 0.005854105 -0.05755686 0.061666952

sample_94 -1.289138105 -3.901085711 -1.328840038 -0.060953441 -4.962837565 -0.928766035 -0.413109031 0.047750705 0.811544183 -0.617431904 -1.006667667 0.639618738 -1.257096652 -0.545676442 -0.923699317 -0.940911705 1.026718676 0.096790708 -0.385514742 -0.54834069 0.126127303 0.040555059 0.336574537 0.216430419 -0.832168235 0.365370668 0.887922335 0.082451409 -0.37718848 -0.279592312 -0.241374259 0.701512606 -0.36427578 -0.094910536 0.241291726 0.876608571 -0.221645286 0.261127932 -0.305208158 -0.125395944 -0.033821509 0.035278776 -0.097518021 0.209861706 -0.066541159 0.169007607 -0.213011064 0.044669305 0.161881808 -0.076480085 -0.024935597 0.252689579 0.404947065 0.111613774 -0.17501692 0.066045072 0.10162484 -0.220342591 -0.148868861 0.133506943 -0.05939171 -0.07664921 0.215576524 -0.081292387 0.218134527 -0.249904682 -0.056746081 0.097048808 0.02019328 -0.071693606 0.03488302 0.011357917 -0.143990911 -0.113210036 -0.150308487 -0.182815815 -0.074616951 0.139888844 0.129823696 -0.25206085 -0.042202719 0.100037307 0.009410728 0.097429927 -0.169209859 0.337338139 0.159823754 -0.094720147 0.0643773 0.141876407 0.168614591 0.012093481 0.019916685 0.18147052 0.092742814 0.120686623 -0.01865401 -0.153556306 -0.005104529 -0.083697292 -0.030045632 -0.091282002 0.029430197 0.068109592 -0.087252413 0.06040345 -0.049333196 0.056149118 -0.019876076 0.072365053 0.049337068 0.001465345 -0.107946445 0.032761307 -0.014149689 0.068334063 -0.118284983 0.123783246 -0.0420944 -0.036399508 0.066430504 -0.042838258 0.021444503 0.098616938 0.052647847 0.017009437 0.16022445 0.065715417 0.004024574 0.094804437 -0.041577274 -0.047917362 -0.076209831 -0.091813006 0.111188717 -0.104347079 0.061762477 0.192000827 -0.016910328 0.015791348 0.107875966 0.014988337 0.078478847 -0.010257128 0.154347967 -0.133496101 -0.055123196 0.086705776 0.059802439 -0.020375193 -0.047915004 0.034110811 0.007244277 0.095889887 -0.039693734 -0.004241762 0.056756317 0.095855642 -0.110800205 0.00850581 0.038141386 -0.081324118 -0.019633847 0.012461375 -0.106220689 0.154464909 0.033734893 -0.021665583 0.023914443 0.163516782 0.034540449 -0.021931849 0.011222014 0.065482762 -0.029708778 0.108334085 0.094716032 0.032972953 0.047263008 0.0132249 -0.13124412 0.08396321 -0.002905315 -0.045878442 -0.03048677 0.151385199 0.018699317 -0.064927411 0.011931156 0.004102134 -0.018039812 0.079259279 -0.065487806 0.006892894 -0.035123341 -0.02004004 0.066266584 -0.021546637 0.098667496 0.09254422 0.027425082 -0.064463176 0.011788585 -0.004666956 0.006537104 0.017828955 0.031673538 0.047701867 0.075737603 -0.097540793

sample_95 -5.24717152 -3.483410906 2.700026448 0.974252331 1.320883939 0.914239907 -0.249308302 -0.451110459 -0.296566751 -0.239741181 0.346184065 -0.880186723 2.332764281 0.105398981 0.08003128 0.069347231 0.50852076 0.184927472 -0.189895473 -0.501049737 0.524884857 -0.249326265 0.178349241 0.070518626 0.104296276 -0.118273808 0.048187635 0.169834781 0.095848872 -0.141712263 0.194878233 -0.048550383 -0.220587076 -0.350662418 -0.056056302 0.180389695 -0.468293723 -0.322804407 -0.398513091 0.002458426 -0.24395755 -0.099010145 -0.159603868 0.429189736 0.13848772 0.230319829 0.290566753 0.103413566 0.248989271 0.216903812 -0.347105957 0.083481998 -0.007220429 -0.066017431 0.044567607 0.020770983 0.198326104 0.267131985 -0.160485739 0.036375345 0.02287229 -0.224983297 -0.008767927 -0.077686821 -0.05616099 0.006214817 0.024422355 -0.074606023 -0.047186315 0.01116244 -0.080212591 0.056489667 0.15869583 -0.196766418 0.00101181 -0.217237934 0.004144726 -0.052737651 0.014499151 0.131753461 0.142417861 0.212891123 -0.118098034 0.056868265 0.051997938 -0.11817972 -0.107012866 0.215315738 -0.050016185 0.036941147 -0.039149526 0.030987906 -0.183796711 -0.149215743 0.021391081 0.005433808 -0.064802432 0.023747426 0.101024452 0.026826323 0.034345905 -0.052124684 -0.211076157 0.084972983 0.024952804 0.043984774 0.017444682 0.135473527 0.006602384 0.008187259 0.009125595 -0.129827363 -0.123340191 0.005648982 0.089703818 -0.036494089 -0.016089324 0.04262072 -0.006565748 0.090815338 -0.004334998 0.104460517 0.015667537 0.064586524 0.158522087 0.00236504 -0.085826302 0.08890797 -0.074149767 0.051439309 -0.095920608 0.07339796 -0.039892578 0.003826623 0.053148098 -0.050884088 -0.054464402 -0.087355619 -0.006209712 0.005274196 0.000170864 0.099888137 0.01612424 -0.139108744 -0.004337978 0.029334827 0.017907557 0.162624665 -0.03094119 -0.064221618 0.033592521 0.027201327 0.01415344 -0.128636441 -0.019253545 0.011027714 -0.022734029 -0.063860867 0.012133724 0.045331334 0.06489626 0.067155476 0.018603034 0.067542991 -0.028384265 0.016971621 0.04011577 0.005113855 -0.083554468 0.025450109 -0.044363274 -0.049972463 -0.093033462 0.017671735 0.000494615 -0.008102932 0.007637229 -0.074904688 0.024459993 -0.082724495 -0.034007297 -0.034347603 -0.080028163 -0.075137922 -0.086191777 0.001907058 -0.037350716 -0.003849079 0.01216905 -0.009827207 -0.047423802 -0.019335978 -0.022482362 0.03911725 0.028416831 -0.06631104 0.020703791 0.017543579 0.034223286 -0.02890331 0.00863314 0.048771282 0.030166145 0.016673018 -0.050947797 -0.037720468 -0.033944067 -0.028492148 0.002733956 -0.010805613

sample_96 5.01119263 -0.897662624 3.173935951 0.049557937 0.440200582 -1.377267169 -0.432843693 0.716316089 -0.452119971 -0.593332829 -0.34655607 0.161864498 0.509047917 0.446576787 -0.486531272 1.082346351 -0.55543606 1.421286205 0.616022596 0.301629297 -0.621978567 -0.727380488 0.011976498 0.375087376 -0.33357045 0.135604836 -0.261372679 -0.1484783 0.559984002 -0.443938767 0.40555665 0.112054569 0.360315545 -0.146818086 0.551856391 -0.240007987 0.2102402 0.384023834 -0.220186774 -0.021133199 0.081832795 -0.043163024 0.150012109 0.092865978 -0.275836867 -0.087037681 -0.265596101 -0.161399581 -0.037847935 -0.221745677 -0.204316379 0.222060865 0.041839465 -0.020965391 0.310702707 -0.16089989 -0.013768628 0.358873582 0.007244354 -0.118607763 -0.026963575 0.023714702 -0.163265673 -0.089024902 -0.027320713 -0.021644751 0.23721107 0.012656872 0.154757382 -0.147137976 -0.074962529 -0.073889773 -0.034561807 0.035972871 0.186193864 -0.144497742 -0.074606585 0.092918416 -0.100410777 0.104553624 -0.037065768 -0.191163064 -0.135388765 0.023355946 -0.060179537 0.054411083 0.055266038 0.017482138 0.045191173 0.111666404 0.005369518 0.176103278 -0.083197723 0.081912277 0.15720424 -0.136570846 0.04127098 -0.135877708 0.07189316 0.082444872 -0.13621416 0.063632963 0.044885991 0.003708637 -0.074700904 -0.007885286 0.079481703 -0.006620849 -0.080599987 -0.088404509 0.040992186 -0.144950416 -0.11811452 -0.125582298 0.022536995 -0.016542231 -0.118937753 0.001537932 -0.017302043 -0.076539768 -0.014664446 -0.03242695 0.041145547 0.165346341 -0.037956109 0.069941436 -0.07776719 0.038039919 -0.028461912 -0.075396567 0.036823558 0.065320133 -0.007848869 0.057694857 0.058449288 0.024371575 -0.073608175 -0.115369028 -0.027406185 0.041070544 -0.046242598 -0.062887394 -0.005305288 0.045479052 0.01657265 -0.04621615 -0.030958816 0.03944223 0.052950887 0.050169956 0.129337848 -0.084097932 -0.058063344 -0.071251157 -0.046813109 0.050937547 -0.020629184 0.054340053 -0.028596308 -0.048758094 -0.063628694 -0.018233404 -0.079606189 -0.01069866 0.047411407 0.04247847 0.062318569 0.019517162 -0.07373708 0.002152057 -0.011971165 0.014857918 -0.043331442 -0.054566466 -0.06571218 0.049322979 0.030167556 0.002549889 -0.001811192 0.097566713 -0.02209835 -0.019250956 0.048941047 -0.012533651 0.030175418 0.038062477 -0.105703361 -0.006465932 0.010318829 -0.003920042 0.027970854 0.008995639 0.002538505 0.098386465 -0.027984769 0.069248689 -0.079597446 -0.027996484 0.001510091 0.086867863 0.052351099 0.001360433 -0.029722634 -0.077021231 -0.037298181 0.093656318 -0.002278233 -0.014426167 -0.00660789 0.020910527

sample_97 -3.77352018 -3.241374234 3.730924018 4.064597099 -1.202625403 0.580083532 -1.031668148 -1.183548443 -1.162767138 -1.241134177 1.406538353 -1.229119218 -0.335791043 -1.875001599 0.559485019 0.489798367 -0.078976546 0.891712601 -0.850142943 -0.13062128 -0.25666477 -0.933420198 -0.480673315 0.897583392 0.093741635 0.068404827 0.054087124 0.646330908 -0.366113371 0.497868783 0.748171656 -0.430537378 -0.383903847 0.05057062 0.120889455 0.046549577 -0.170432062 -0.086479352 -0.485475982 0.275014132 0.069480102 0.027898537 0.321064763 -0.348957823 -0.109065776 0.333084574 -0.047269669 -0.112421424 0.008828721 0.302727125 -0.099062705 -0.086297534 0.04288552 -0.25308229 0.192018683 0.060695765 -0.089871174 0.067654311 0.246305763 -0.05018138 0.033683362 0.2195651 -0.042740203 -0.145854847 0.035098102 -0.114920482 0.141386705 -0.296002572 0.266923534 0.228028733 -0.344029472 -0.003557816 0.10366517 -0.016020722 -0.142874645 0.069945715 0.038760129 -0.122782624 0.063681814 -0.010746117 -0.045987999 0.134375778 0.073899304 -0.112823247 -0.044024027 -0.058985821 -0.051013561 0.001361516 -0.054199008 -0.13856216 -0.059296652 -0.104662911 -0.070989856 0.144706063 -0.08276833 -0.143678558 0.096830649 -0.138949382 -0.079673847 -0.021101298 0.09866012 -0.231533838 -0.074118483 0.025385966 -0.045451133 -0.312765107 -0.014883898 0.153237468 -0.047889678 -0.143300043 -0.106982338 0.097229856 0.010580558 0.074599901 -0.065491588 0.108735924 -0.153357456 -0.101809983 -0.069507976 0.127186254 -0.044444857 0.185469123 0.110380695 -0.115817303 0.146959729 -0.000862684 0.104922156 0.075608194 -0.067140204 0.056663793 -0.063288522 -0.01423544 0.11047055 0.028378376 -0.219972117 0.124902373 0.160991006 0.026385409 0.027265312 -0.06844373 -0.054394884 -0.063098325 0.147057232 0.098375736 0.013698365 0.111356037 0.030137138 0.085110664 -0.024242111 0.010987763 0.010937013 -0.075411944 -0.032573501 -0.105562932 -0.012109524 -0.020247992 -0.011437427 -0.024819726 -0.086652625 -0.047320752 -0.04765843 -0.071662047 -0.075501117 0.097581283 0.128409902 -0.105691747 -0.031666343 0.03854074 0.035955751 0.070477864 0.015205203 -0.15080921 -0.137778985 -0.134134478 0.003319131 0.023806478 0.064927592 0.040247881 0.035466062 0.037659853 0.016210244 -0.02014358 0.030962768 0.008246799 0.064455938 -0.073707787 0.154852594 -0.088097974 0.080857173 -0.074035943 -0.011986419 0.027037935 -0.013758432 -0.090982273 -0.040962492 -0.036376648 -0.017855577 -0.057014973 0.002093235 -0.013367185 -0.040802444 -0.02131634 -0.072999006 0.058841245 -0.082347653 -0.020092046 0.063296388 0.085434456 -0.02317882 -0.021434675

sample_98 -5.973033816 -2.117580272 0.724715252 0.46926762 -1.446953396 -0.135979062 -0.431097495 -0.145584027 -0.501127258 -1.462605241 0.884215325 -0.417960685 1.123963638 -0.675562984 -0.44932251 0.084337808 0.758968144 -0.172120576 -0.012633821 0.046518733 -0.630662604 -0.674541287 -0.28081943 0.301747997 0.28764843 0.119346925 -0.649344482 -0.35252687 -0.152583262 0.021316166 0.679104398 -0.732561939 -0.597274544 0.014341963 0.3656798 -0.587401181 -0.139109627 0.055761221 -0.010898954 -0.184387732 -0.037525601 0.304800461 0.237051378 0.133423595 -0.066483986 0.196497642 0.29447681 0.354283436 -0.051260292 0.030106053 -0.10814902 -0.274636927 0.056891222 0.040905785 0.021067984 -0.146907277 -0.146916486 0.160453371 0.168948985 -0.173651739 0.141999152 0.092982189 0.188641455 0.060932283 -0.06476981 -0.172690437 0.1698671 -0.021949629 -0.080672282 -0.246278485 0.008346149 0.095321188 0.148423638 -0.079990309 -0.00675949 0.02346447 -0.11574902 0.080994412 -0.083434353 -0.090627953 -0.122181062 -0.214305251 -0.093233144 -0.049663625 -0.140584135 -0.185160364 -0.093155089 0.096750065 -0.093683047 -0.10169768 -0.063129431 0.002297674 0.060399744 -0.011568826 0.014096499 0.019109606 0.018541917 -0.076712738 0.059115938 -0.106726765 -0.051920865 -0.077684063 -0.0995535 -0.037988738 0.06539105 0.050669593 0.159657879 -0.004524808 -0.006502567 0.1422564 0.029061547 -0.053202978 0.002723426 0.016772556 -0.016694867 -0.119516468 -0.017558052 0.237151793 0.108817933 0.004236603 0.031309959 0.055627045 -0.07237017 -0.06363031 0.096727566 0.02551947 -0.026203072 -0.00511876 -0.04109876 0.037605253 -0.010507238 0.042872332 -0.058065997 0.018316794 -0.11532849 0.007758711 0.000704436 -0.028468823 0.06570632 0.028418203 -0.022130957 0.010065698 -0.072411298 -0.085072955 -0.019914042 -0.118473186 -0.111591113 -0.020182865 -0.003830411 -0.035057734 0.006720003 -0.048138945 0.08849755 -0.0794623 0.067608479 -0.063989647 0.001666922 -0.05873994 0.061608196 -0.053876034 0.076877478 0.008583225 0.131652412 0.114238032 0.024540953 0.019548882 -0.133931432 0.027719383 0.018475429 -0.023261448 0.025020461 -0.035866617 0.020240755 -0.055372614 0.04354164 -0.018597036 -0.012381488 0.060495023 0.042743528 0.093298925 0.061112162 -0.018768917 -0.02587033 0.059298628 0.045879422 0.034493147 0.047279055 0.014355618 0.014773341 -0.010596253 -0.064847681 -0.038125053 -0.024698473 0.070821814 0.009898118 -0.004669133 -0.01217585 -0.002065217 0.058642751 -0.040921079 0.002172488 -0.02536052 -0.033670498 -0.01403886 -0.047082408 -0.012642261 -0.013220934 -0.026817337 0.022177686 0.032416928

sample_99 -7.582457333 1.94454017 2.152664257 8.849664529 0.693066905 0.292884497 -3.465076536 -1.576745985 0.710810639 -0.072953207 1.025580928 -1.266569649 -1.502908747 -2.157239131 -0.47340144 0.722495017 -0.20239731 -0.386822592 -0.083832193 1.556821469 0.134629279 0.09809969 -1.019805099 1.541107558 0.599386653 0.56196824 -0.552891246 -0.513626723 -0.446735026 -0.000554055 0.235949637 0.039447333 0.148407202 0.276299784 0.178248254 0.277097878 -0.116268906 0.678359768 0.004103326 0.738065256 0.271970468 -0.401820426 -0.247390817 0.368708664 -0.382037381 0.185646332 -0.075942669 0.10793524 -0.188054666 0.540791704 -0.046353893 -0.244365934 0.165724213 -0.012370243 0.023362779 0.411940942 0.200643161 -0.140992976 -0.034223425 0.144460298 -0.193196312 -0.196844635 -0.177119605 -0.075497261 0.155844794 -0.196862172 0.125496663 0.041884629 0.00062406 0.44244242 -0.216618609 -0.175345909 -0.145279042 0.096159405 0.239517929 0.175613918 0.230716503 0.166588422 -0.110467072 0.112182486 -0.026357756 0.392425467 0.056349528 0.077293766 -0.144597486 0.267137274 0.259258076 0.009208155 -0.149478774 -0.007028756 -0.144886691 0.011767667 -0.174743184 0.071348896 -0.262720539 -0.014794169 -0.026280656 -0.07504906 0.156765926 -0.064181889 -0.021740114 -0.233732996 -0.151750598 -0.003974594 0.139177513 -0.20390929 -0.017308063 0.031409061 -0.042207037 -0.077138296 -0.253742116 0.227637995 -0.019507914 -0.0953875 0.012395489 -0.064222548 0.052130943 -0.073878479 0.036212776 -0.061974873 -0.123953659 0.047112808 0.050403246 -0.074793249 -0.126164826 0.051685923 0.038672384 0.035614658 0.123833382 0.183998103 -0.088508889 -0.058953789 -0.02183174 0.131967696 -0.229859502 0.003403148 0.119004201 -0.06939992 -0.026237526 -0.003379356 -0.133709816 -0.082430762 -0.048405062 0.100801653 -0.033224112 -0.11958916 -0.013438044 -0.067455208 -0.030029905 0.049545255 -0.097516633 -0.003270865 0.019555276 0.071747352 0.033721772 -0.076457106 0.109671111 0.067725345 -0.131242251 0.065989329 0.071724915 -0.128254491 -0.038999529 -0.207576296 0.340969639 -0.070916248 -0.011716476 0.046248787 0.086021504 0.028886706 -0.044486825 0.019862153 0.045438987 -0.004800565 0.036288365 -0.109028357 0.014685398 -0.017724485 -0.039472097 0.10802711 -0.165878202 0.032569503 -0.120627665 -0.002015083 0.041773398 0.0180156 -0.058591927 -0.079436939 -0.090463836 -0.030793908 0.088400676 0.070231461 -0.030839313 -0.022072788 -0.128697114 0.070351787 0.082508711 0.057175876 -0.047186382 -0.074123643 0.056674115 0.020800147 0.021959909 0.045802933 -0.061209695 0.000228416 0.051243479 0.095844541 0.020269027 0.028785115

sample_100 7.091843307 -1.041577727 1.194056121 -1.25359827 0.05407528 -0.046813848 -1.49742837 0.536466885 -0.855884134 0.095052778 0.189948408 -0.275291795 -0.924823927 0.087982488 0.097583274 0.857602308 0.184425424 -1.061995458 0.710979241 -0.258387082 -0.1399199 0.5100387 -0.194493874 -0.046808257 -0.996043348 0.088123733 0.304876514 0.025764747 0.026197641 -0.398008619 0.464566559 -0.406532483 0.000852474 0.947764789 -0.385792732 -0.068352295 0.523972157 -0.051056112 0.049712933 0.095849743 -0.06915376 -0.086792089 -0.330959581 0.055370497 -0.249264195 -0.0675147 -0.233666488 0.249509153 0.123209294 0.054571103 -0.073343494 0.166187824 0.226581409 0.001641127 0.209998051 0.226552313 0.081557422 -0.070056693 0.126413395 -0.059966304 0.250937653 0.04343332 -0.145455329 0.132146736 -0.166232952 0.211812407 0.03498402 -0.032249142 -0.011572354 -0.169651314 -0.053618364 0.001312541 -0.007192477 -0.006131741 0.295714688 -0.075176796 -0.171789565 -0.122296018 -0.135348938 -0.087667998 0.13719875 0.010834143 -0.095724564 0.21572359 -0.082454847 -0.211042753 -0.001196204 0.046872842 0.024302686 -0.037536578 0.003196949 -0.036606045 0.082447634 -0.112706692 0.012057361 0.050879066 0.060821842 -0.102949543 0.191428495 -0.122325397 0.063992753 -0.234411903 0.028324601 0.253796058 -0.018605809 -0.071802307 0.164085378 -0.01722601 0.036936009 0.017575721 -0.104212204 -0.143359923 -0.073845581 -0.030479001 -0.099991878 0.042846499 0.184441996 -0.013581623 -0.111209889 -0.002119616 0.034455329 -0.105769929 -0.13651779 -0.068168021 0.095633005 -0.07050799 0.071798705 -0.024421923 -0.006983794 0.114348542 0.028406774 0.057421919 0.00715887 0.058108788 -0.062554753 0.009411909 -0.025934729 0.014965711 0.036598903 -0.085671313 0.018086724 0.044076987 0.142734946 -0.065760403 -0.033010854 -0.039875068 0.115489428 -0.038264509 -0.110184016 -0.0061786 -0.064443916 -0.096844388 -0.011713913 0.060194126 0.057212829 0.037145999 -0.014195638 0.096117038 -0.063279791 -0.02112727 0.043428443 0.033007658 -0.001846807 -0.009613631 0.048336197 0.006277037 0.049715579 -0.078562985 0.006807642 -0.024590161 0.00020096 0.025703105 0.02348674 0.038934921 0.042102468 0.157199578 -0.026249987 -0.026440297 -0.009309524 0.038693589 0.077551064 -0.006688494 -0.006653226 0.029660282 0.049890431 0.029007712 -0.037331594 0.049815885 0.024874766 -0.014929074 0.009339707 0.007006566 -0.037911427 0.005108513 0.054180935 0.013621284 -0.067521853 -0.014638771 0.058543238 0.006465814 0.02732956 0.02127684 0.046863506 -0.064383555 -0.052609848 -0.006185995 0.0563111 0.050174106 -0.033732255 0.022145166

sample_101 -4.043303556 0.114104057 1.570531395 3.104004512 -1.539338158 -0.016587414 -0.891301911 0.09160268 0.71179109 -0.071300318 0.177642721 -0.185525607 -0.079303809 -0.522747204 -0.037629613 0.052854393 -0.014977744 -1.118466691 0.236030019 0.077947374 0.335667931 0.376330278 -0.036381343 -0.053860244 0.054346429 -0.239593015 -0.522767949 -0.105388775 0.363861868 0.060775614 0.26451946 0.039897705 -0.460741674 0.098281226 0.206778936 0.019580897 0.005495022 -0.082482166 -0.072480511 0.00404942 -0.172843434 -0.112018165 0.24284343 -0.226270751 -0.084345658 0.139270858 0.24419084 -0.292115941 0.127750929 -0.101199053 0.263029935 0.083688376 0.114666931 -0.386484696 -0.05844319 0.039654942 0.094728426 0.042437297 0.248332798 0.010719286 -0.245948294 -0.081513233 0.065813754 0.018474133 -0.010264425 -0.061098767 0.261915828 0.032293761 0.214796117 0.033500233 -0.04714552 -0.01284255 0.099121431 -0.11077351 -0.01154829 0.026549707 -0.22326817 -0.142372154 0.084842148 -0.045583582 -0.058408574 -0.047645074 0.193380396 0.030978976 0.165586239 -0.084963118 -0.04620246 -0.163723867 -0.007351677 -0.07647871 -0.039425821 -0.194105494 -0.038702108 -0.12468236 0.120433357 -0.053999479 0.016080217 -0.101321102 0.102776362 0.008614214 -0.062309322 0.110099938 0.006189552 0.069694559 -0.142284939 0.009477133 -0.047292344 0.0355164 0.011820715 -0.049823236 -0.132874935 0.0682096 0.041059052 0.13225357 0.035214621 0.030386919 0.087274514 -0.097781434 -0.118698575 0.042988689 0.01831693 0.03703636 0.019496932 -0.000396148 -0.064211383 0.014158819 -0.103476063 0.054663703 -0.01583183 0.020033686 0.122549595 -0.085982231 -0.073478704 0.079015095 0.071473254 0.025759767 -0.017046637 -0.072516548 -0.000612415 -0.073483584 -0.095676371 0.075456628 0.05996571 0.047013525 -0.02801438 0.035843037 0.017847929 -0.022726593 0.025881153 -0.003436069 -0.019528016 -0.035311684 0.037561701 -0.066648357 -0.00325347 0.123445114 0.005004156 -0.036874491 -0.069445521 -0.009365016 -0.057890219 -0.000409824 0.030502884 0.004956856 0.101133655 -0.166357121 0.013796918 0.086514009 0.058833219 0.014566567 0.056726705 0.123092066 -0.062784253 0.025574863 -0.027764602 0.010491408 0.078605271 -0.010727287 0.018881335 0.017647077 -0.102633293 0.063796919 -0.022983488 -0.052065864 0.017113688 -0.046310595 0.022927306 0.061493427 0.005197933 0.028267282 -0.006027064 -0.015644624 0.060420273 -0.028978649 0.008327499 0.009295066 -0.007901354 0.03061215 0.039265225 -0.036372124 0.018541803 0.041446239 -0.050738034 -0.02106381 0.012375161 0.071266825 0.009026803 -0.009694096 0.023369235 -0.05827497

sample_102 0.245517266 2.088460677 4.836301451 1.237682643 -0.910524413 -0.119009773 -1.663162387 0.315582763 -0.232844944 -0.299388906 -0.084288004 -1.722038762 -1.652981459 -0.991326923 0.379634855 0.388789624 -0.847697003 0.343406955 -0.923824652 -0.299794231 -1.198358903 0.288195263 0.366586314 -0.058421683 -0.064966996 0.630742093 0.91926013 0.177791431 -0.202656511 0.260810304 0.100377046 -0.139374964 -0.221371126 0.196579633 0.23432147 -0.27970056 -0.284673814 -0.302784221 0.229285832 0.538798804 -0.297235471 -0.616842094 -0.023313614 -0.344072512 -0.110792846 0.201904361 -0.167882115 -0.287372306 0.209000887 -0.175904816 0.288088417 -0.147979914 -0.369159029 0.024694056 -0.077896501 -0.129959085 -0.170889243 -0.274904489 0.081094047 0.136809157 0.13344758 -0.005547286 0.145303706 -0.3700292 0.221352522 -0.038821192 0.128813239 -0.25197051 0.184704383 0.036267104 -0.090234592 0.14574178 0.197937845 -0.254548803 0.214076819 0.128491287 0.176126869 -0.223683612 0.152893784 0.286431539 0.074718065 -0.221210772 0.186192293 -0.254471856 -0.009582002 0.016208757 0.280660715 -0.215411372 0.161933032 -0.110607343 0.076898477 0.082930746 0.205244743 -0.023860116 0.046379336 -0.086128954 -0.061625706 0.087702088 -0.055787806 -0.084073206 0.127422023 0.034724405 0.266299809 0.146109972 -0.039688165 -0.276977759 0.059309294 0.104141985 0.11111878 -0.291394218 -0.073644564 0.016715024 0.119238248 0.044167201 -0.202586504 -0.058950571 -0.056594718 0.017456885 0.019078261 -0.158598954 0.050789792 0.000111575 -0.097159893 -0.077806114 -0.057889412 -0.010450478 0.06470612 0.183488064 0.073765438 0.050871532 0.126462244 0.048498246 0.021570122 0.08337618 -0.088816448 0.147061131 -0.057306853 -0.002763994 0.103465183 0.122136998 -0.092514668 0.023004531 0.142592798 -0.033686055 -0.079337948 -0.028476726 -0.006529465 0.017699678 -0.095291809 0.02515554 0.152495368 -0.032055766 -0.000455923 0.1104017 0.009269998 0.015936969 -0.0409942 0.056075489 0.042332957 -0.063513693 -0.089491047 0.030217524 0.050459108 0.006666176 -0.103775554 -0.01971835 -0.013879359 -0.179118834 -0.016782412 0.029216424 -0.083757798 0.054839921 -0.035628058 0.130213113 -0.017841077 0.066373874 -0.179282809 -0.076254284 -0.01831592 0.096194863 -0.028320141 -0.132047047 0.095479501 -0.004195147 0.006067525 -0.116449306 0.085128471 0.054249097 0.031336034 0.002350202 0.081395639 -0.025967448 0.005133286 0.04062059 0.073728898 0.023303305 -0.089361537 0.042734031 -0.060144815 0.060451374 0.038589155 -0.075752795 0.046343462 0.064792141 0.132363321 -0.030150288 0.059045921 -0.057180297 -0.045510971 -0.031935319

sample_103 0.140111023 10.28083121 5.434605645 1.475671693 -2.590204934 2.856249376 2.730592196 0.838391714 -0.427735689 2.277277716 0.718176986 -0.08059271 0.838170515 1.019358404 -1.734639263 -1.426365547 0.35123887 -0.494266499 -1.76951223 0.479967784 0.109614171 0.756740225 -0.213008236 1.300023712 -0.082016881 -0.924980357 0.594398382 0.219470136 0.375931302 -0.731090281 0.636379997 0.462021488 0.48201122 -0.798568586 -0.821976343 0.067990307 0.691437534 1.769289381 -0.07155012 0.592981771 0.018466524 -0.14792526 -0.337401828 -0.509298562 0.48751496 -0.449452542 -0.248410175 -0.28018091 0.139347308 -0.046633308 -0.080302035 -0.79912623 0.545631996 -0.558481134 -0.537037839 -0.038871821 0.038863048 0.30713461 -0.070948285 0.198670212 -0.366661441 -0.048569105 -0.52458483 0.185507404 -0.225336581 -0.545004225 0.403116322 0.546333322 0.650010778 -0.343065912 -0.057681534 0.17036012 -0.153538481 0.130494466 -0.285291455 0.173604532 -0.81565063 -0.042416916 -0.078357064 -0.192397698 0.035843632 -0.448921309 0.059466419 0.172571688 0.424157268 0.375531035 -0.21970465 0.03225811 -0.090964017 -0.005448762 0.069720019 -0.353125286 -0.159474184 -0.057126771 0.004260826 0.579827851 -0.061527106 0.107879035 -0.061080041 -0.059915241 0.326287004 -0.294402854 -0.009663758 0.051365835 0.067217418 0.048263365 0.004351409 0.262740468 0.299967401 0.026144079 -0.137958837 -0.249321264 -0.02263596 -0.054792906 -0.017621522 -0.037848216 0.037618337 0.170296443 0.182465302 0.271585255 0.33845414 0.038192698 0.018330711 0.008131396 0.135390043 -0.018612883 -0.121929933 0.1576858 -0.055776229 -0.062219388 0.129883989 0.004972009 0.074606061 -0.13304597 -0.006316062 0.002244865 0.064792441 0.16069651 -0.092581607 0.058544666 0.210993832 -0.009302599 0.020856282 0.119767072 0.257243163 0.109654217 0.069081592 -0.097722654 0.080694386 -0.02733708 0.081883643 -0.090243071 -0.222292289 -0.041814637 0.176230735 0.060158817 -0.011061623 0.087374958 0.046564019 -0.051045723 0.0146638 -0.12985695 -0.151516564 -0.141347959 -0.092470571 0.112775624 -0.139484532 0.054071409 -0.004620105 -0.17711378 -0.009395571 -0.090187152 -0.019913246 -0.038457171 0.086534667 0.098395357 -0.125023201 0.012404778 -0.002992181 0.083318993 0.075371676 0.075194536 0.045110798 -0.076013897 -0.039797228 0.00044919 -0.026687107 -0.090351322 -0.096497363 0.140760225 -0.043883677 0.05920177 0.001215907 -0.03702431 -0.036453972 -0.103999526 -0.01893214 -0.110358104 0.009550234 0.0827317 -0.054471412 -0.002013722 0.066115091 -0.034043413 0.024895822 -0.062830402 0.01352674 0.090550627 0.122943891 0.044189492

sample_104 0.365041513 6.186183315 3.136050504 -0.212612694 -0.893377049 0.129409664 1.420444555 -0.109003797 -1.142175499 0.209940147 0.205413211 -0.125804876 -0.476747093 0.508883581 -0.183225833 -0.384851325 -0.708201307 -0.452129398 -0.572667767 -0.088104237 -0.319806427 0.385775435 0.418950251 0.117785659 0.71250891 -0.048754209 0.15932055 0.003276629 0.279066555 -0.334653028 -0.109837925 0.13955624 0.14890943 -0.206461464 -0.201166732 0.170958693 -0.027289664 0.285812785 -0.251135663 0.066072065 0.025456103 -0.130724754 0.049326561 0.230814926 -0.106705291 -0.240202741 0.368666888 -0.110360623 -0.024883224 0.085786151 0.063845554 -0.062989733 -0.067636873 -0.334452786 0.114123023 0.090861818 0.173719485 0.002375384 0.027691913 -0.288869211 -0.153984859 -0.277609136 0.188243866 0.057448149 -0.235649765 -0.211516024 -0.302548072 -0.223640746 0.323144223 -0.132888033 -0.214674047 0.153979766 0.170364199 -0.072006254 -0.31869294 -0.026870119 -0.057326205 -0.386861214 0.01135582 -0.253759256 -0.119736724 0.064686793 -0.145780856 0.124523704 -0.011288372 -0.096953686 -0.134403831 0.079931123 0.013742306 0.081423251 0.052053774 -0.112669933 -0.003445762 -0.060652478 0.18736812 0.053479448 -0.077675376 -0.134597484 0.078528866 0.056428376 -0.010916003 0.046345307 -0.116750036 0.053477732 -0.055368181 0.114292134 -0.175068711 -0.130768245 -0.125491281 -0.166801212 0.197443624 -0.134045739 0.053395188 0.022549237 0.121854693 0.065183842 -0.043209832 -0.066021058 0.103623223 -0.043880939 0.101542294 -0.081657696 -0.058049008 0.004431663 -0.02062298 0.064734149 0.100454725 -0.034334163 -0.051728234 -0.013774521 -0.042252633 -0.108092103 -0.003927613 -0.011252683 -0.024326031 0.038765606 0.038635067 0.109826354 -0.003649214 0.053699296 -0.048615018 0.023372718 0.043713858 -0.050954858 -0.01684315 -0.013781394 -0.075479131 -0.044261614 0.165802693 -0.053348599 0.038063785 -0.027528391 0.115652614 0.073209157 0.072179044 0.009855953 -0.082560352 0.056328028 -0.069630055 0.108085973 0.018475245 -0.020900928 -0.045233115 -0.118896804 0.119835196 -0.008351577 0.020989568 0.023436328 -0.003522304 0.0085747 -0.045045536 -0.070056626 -0.071530994 -0.101622533 -0.013435662 0.051812444 -0.013542913 0.017320441 0.064895144 -0.010514349 0.105433964 -0.023664887 0.001348843 0.028041716 -0.060699162 0.114116123 0.004551427 0.022644626 0.041940621 0.002343197 0.014524179 -0.001049382 0.031317034 0.077837278 0.063367987 -0.030576767 -0.004429584 0.079202137 -0.036362169 -0.069845773 -0.024610071 -0.026350435 0.019587935 0.015864709 0.022270243 -0.012823685 0.00646674 0.054443089 -0.037051769 -0.06633233

sample_105 -5.869431087 3.155938208 -3.340484783 3.293212834 0.671338233 -2.127472301 3.219853035 -1.561125295 1.697702832 -0.788262219 -0.965579274 -0.190911612 0.221716136 -1.500322959 -0.725174781 2.026178988 0.29958908 -0.996732656 1.150714262 1.555867012 -0.963383003 -1.085049436 -0.455183644 -1.091595201 -0.282513234 0.337914323 1.193416751 0.34658292 1.080150234 -0.254544801 -0.42026772 0.008983092 0.804213018 0.262758921 -0.285570942 0.29376255 -0.68117394 0.016583749 0.115126936 -0.411644567 0.511423257 0.670958133 -0.107957974 -0.450460613 -0.213436371 -0.598013534 0.125744055 0.48115332 0.04589149 -0.27881572 -0.01383269 -0.321566735 -0.075197354 0.312026602 -0.242758552 -0.745714697 -0.489980467 0.15539141 -0.031026048 -0.205499585 0.078986568 0.026546643 0.566667066 0.095042903 -0.437713071 -0.253987741 -0.095732942 -0.024429037 -0.079570639 0.280474252 0.184701815 -0.073874891 0.019945524 0.172266325 0.183798739 -0.090225157 -0.351670639 -0.020812742 0.070285841 0.214355177 -0.193442376 -0.165441209 0.080683072 -0.035769881 0.478751264 -0.21966597 0.010034405 -0.115939359 -0.017846484 -0.121520333 0.12891503 -0.02636301 0.01064052 0.076729605 0.103088577 0.206396361 0.072436953 -0.108249596 -0.078369799 -0.267422933 0.134714214 0.012265034 -0.083429183 0.051413763 0.301925831 -0.133840589 0.105882658 -0.232637265 0.053361381 -0.071812113 -0.085994554 -0.037408032 0.061937608 0.087450007 0.143640215 0.183100899 -0.028322094 0.029413107 -0.022295867 -0.054864239 0.175692097 0.041219439 0.018923653 -0.041638177 0.161723498 -0.157093 -0.104226715 0.071736254 0.084034786 0.065056808 -0.001302227 -0.19683103 -0.003916134 0.061566873 0.054335657 0.101208645 0.070406241 -0.040494414 0.140979651 -0.003446322 -0.032189712 0.179926138 0.067697672 0.048115817 0.063793746 -0.022386778 0.000961171 0.041058869 0.071522499 -0.02927055 -0.06335787 -0.1733898 -0.029346258 -0.094384173 -0.026670238 0.028795914 -0.048863428 -0.029828941 -0.031911578 0.042515962 -0.053547461 -0.043281659 -0.000274253 0.057004841 -0.007418869 0.044423163 0.097920648 0.016039106 -0.00661286 0.01180946 0.038040206 0.074757714 0.06028074 -0.054432827 0.103353701 -0.046715339 -0.042163575 -0.079924143 0.015845858 0.003469708 0.047507366 -0.031715508 -0.026617193 0.063259226 0.015349328 -0.047458844 -0.067133401 -0.066325335 0.062507192 -0.021537793 -0.066522157 -0.045686173 -0.038678469 0.024921187 0.05217281 -0.069553895 -0.033447143 0.013388971 -0.036979877 -0.033134451 0.043036455 -0.060499406 0.014912012 -0.031623818 0.009280575 0.041170979 0.05722385 0.013657483 0.046269429 -0.166622574

sample_106 1.951880667 -2.830202502 -1.190184044 -0.136947731 -1.42330191 0.679442796 -1.114409145 0.946662027 -0.315982959 0.263688525 -0.915696744 -0.029498769 -1.425575053 -0.119360653 -0.317656756 -0.086002453 0.547147999 0.388035677 0.525288853 0.15869637 -0.194061966 -0.248445258 0.06219368 0.03592106 -0.575493983 0.014179222 0.157734593 0.285904028 -0.841130414 -0.30427048 0.059582097 0.040543116 -0.256747709 0.142977618 -0.475149821 0.503229907 0.183941282 -0.078544591 0.092640313 -0.165538398 -0.186107579 0.105114998 0.377718192 0.05247023 0.256188688 -0.205340259 -0.106751871 0.077886044 0.05690786 -0.143125078 -0.155589045 -0.00494691 0.160083 -0.008845118 0.255993807 0.132014445 0.198872677 0.052631533 -0.013098526 0.137607475 0.039240296 0.067500927 0.153438624 0.053460508 -0.125743534 0.004777161 0.0561286 -0.03059708 -0.205229737 -0.143899945 0.039109991 -0.027973716 0.164184428 -0.250843601 -0.068662944 0.080467727 0.218052352 -0.151981132 0.124474818 -0.021967903 0.123122272 -0.184753847 -0.025917419 -0.052420391 0.035225284 -0.255192638 -0.013701572 0.019212746 -0.203013378 0.10629247 0.145038166 0.006758539 -0.046953013 0.09643647 -0.02442554 0.13474509 -0.080275886 -0.0820833 0.025859566 0.008267773 0.106430993 -0.059165348 0.017329595 0.161914681 0.005446822 -0.159416049 0.111303407 0.148864915 0.080172355 0.032852395 0.117591355 -0.04414444 -0.029931445 0.037231072 -0.076993451 -0.008756068 0.106251196 0.071949592 -0.036553696 0.044709986 0.070359788 -0.044874894 0.155762997 0.150421564 -0.102934935 0.024027944 0.10132968 0.014199735 0.033643071 -0.080212724 -0.087661488 0.042673966 -0.007723827 -0.031358111 0.05462776 0.008374872 0.00589118 0.020241601 -0.052720908 0.062744559 -0.099800911 0.055432948 0.092686177 -0.044648033 0.041268478 0.002076203 -0.03725166 0.034056934 0.078629876 -0.023483296 0.018333469 -0.037307718 0.031416613 0.084836788 -0.043874793 0.070368804 -0.00549382 -0.031576985 0.028641458 0.153253896 -0.037072263 -0.077005619 -0.014398751 0.015987678 0.017667032 0.109485718 0.004710583 -0.048496769 0.028473265 0.028354745 0.011283415 -0.07005833 -0.024058243 0.040145015 -0.032939621 0.024060227 0.059244594 -0.043162819 0.049286311 0.020642452 -0.016524158 0.066770596 0.011407417 -0.023060992 -0.019753755 0.039306051 -0.069148039 0.015505567 -0.033652763 0.003167804 -0.046306485 -0.029124859 0.001172457 0.049366785 0.04723669 -0.093552464 0.046599768 -0.047588883 -0.104562586 0.027712871 0.006876357 -0.037245654 -0.012418785 -0.00336684 0.022581085 -0.010301827 0.037347541 0.024068305 -0.024464651 -0.046582289

sample_107 3.229362673 -0.598778359 -0.165781043 -0.646406346 -0.430563778 -1.805046139 -1.034726376 0.053296777 -0.920782383 0.177762527 -0.253038623 -0.288158078 -0.846900791 2.050815486 -0.589141359 1.106526772 0.107391689 -0.085344003 -1.062051234 0.379157658 0.712041486 0.003109118 -0.309672129 0.210932778 -0.256671864 -0.338060772 0.531617332 -0.784875633 0.121936399 -0.1659772 0.485486899 0.041529572 0.609541426 0.356221583 -0.089803163 0.026630028 -0.156888339 -0.190304116 0.215800129 -0.128294333 0.104569984 -0.355377808 0.204813505 0.194265272 -0.022840328 0.207442648 -0.139019514 0.35760258 0.305321838 -0.265036219 -0.221841433 -0.081292738 -0.088503501 -0.040637834 0.326567429 0.038115382 0.35452062 -0.169678422 0.217606777 -0.073854561 -0.020244896 -0.198599922 0.036562216 -0.05498946 -0.089547306 -0.259370973 -0.300957654 -0.212157695 0.015081055 0.073096757 0.20732936 0.005701634 -0.030834566 0.00955899 -0.088675353 0.090900868 -0.025261301 0.015720021 -0.140650094 0.11471961 0.003248248 -0.011827325 0.082405461 -0.276235084 -0.055445266 -0.04268596 0.070680601 0.060160872 -0.176003375 -0.166675415 0.058070267 0.088426512 0.066357854 -0.080425257 0.020792625 -0.054591258 -0.198290708 0.146119236 -0.073879291 -0.000667907 0.086868385 0.010915666 0.059209666 -0.091325251 0.239037175 -0.003072358 -0.111338819 -0.08510237 -0.047106164 0.145417503 -0.013840594 -0.074831047 0.083570726 0.034387864 0.002364311 0.123868141 0.067650795 0.050301311 -0.104232517 -0.020754141 -0.099420222 -0.022413056 -0.072557757 0.022866147 -0.044495593 -0.047096964 0.052882686 0.071866816 -0.057896929 0.049840055 0.041264748 -0.005912943 -0.099369815 -0.107574392 -0.092255452 0.023049731 0.0340466 -0.03597086 0.10787813 -0.088039056 0.019350483 0.024515445 0.047978846 -0.042516315 0.056657902 -0.018043852 -0.066920497 0.008255649 -0.050140423 0.065872798 -0.057660738 0.030824641 -0.073232758 -0.074170174 0.020064821 -0.040769806 0.00910509 0.026279901 -0.046327035 0.012650246 -0.011822665 0.087733336 -0.091781876 -0.00389014 -0.077276895 -0.016547885 -0.068917772 0.057773317 -0.09175887 -0.020826532 -0.046540315 -0.062535623 0.01813187 0.072181006 0.038743571 0.029139365 -0.018639349 0.007939464 0.016097278 0.008164299 0.012323412 -0.020023546 0.024958708 0.020389696 -0.057931523 0.030395536 -0.037135414 0.000427803 0.01314276 0.011701103 -0.003220085 -0.006708265 0.008851239 -0.021124029 -0.023150403 0.049010475 -0.098141163 -0.022550157 0.014688068 -0.039615008 -0.022576664 0.058611115 -0.056835317 0.027113106 0.001315673 0.06356602 0.001110894 0.06265601 0.031844053 -0.009613565

sample_108 -5.967024434 -1.218781122 -0.860893125 0.037502523 -0.151827565 -0.151144131 -0.549532943 0.336262063 -0.442423964 -0.557729286 -0.678695432 0.413068138 -0.470497618 0.444920615 0.539270922 0.122350437 0.752106832 -0.199939919 -0.118611328 0.326489866 0.096375871 -0.188533961 -0.213969931 0.28134092 0.145856225 -0.240706698 0.495431982 -0.023396875 0.101847163 0.033067905 -0.247236621 -0.21521565 -0.029948383 0.031450453 0.270526241 -0.32639452 0.231788198 -0.214619977 0.485149578 -0.199709789 0.343148556 0.291755295 0.398925551 0.006851748 0.028801119 -0.179277554 -0.23375771 -0.082912548 0.033674531 -0.135454939 -0.02248271 -0.089613622 0.17042133 0.154718229 -0.217332676 -0.170864655 -0.109723818 0.084316403 0.196315532 -0.140846955 -0.067821454 0.084712289 0.140021448 0.202883209 -0.067326964 -0.111424534 0.036463143 -0.169106 0.033938278 -0.013760084 -0.054643339 -0.033442753 -0.003432168 0.115285144 -0.046892389 -0.033987308 -0.008184438 -0.069312631 0.058088455 0.013200834 -0.022119144 -0.044705216 0.015171516 -0.132736076 -0.009770752 0.027395071 0.101166831 0.039728886 0.099638944 -0.029282697 0.078980602 -0.042545265 0.038452818 -0.098896421 -0.004477937 -0.021962724 0.07369299 -0.002263779 -0.136841621 0.111259832 0.093586677 0.084280398 0.021144999 0.027814075 -0.017257895 0.020589422 0.013006157 -0.107968175 0.030630284 0.185420463 0.013362806 0.050168138 0.054292039 0.109267191 0.109108151 0.03279634 0.054781128 0.027964404 0.040843038 0.042744196 0.000494476 -0.052144915 -0.024696912 -0.002387102 0.017672369 0.086598379 -0.010147064 -0.067478262 0.015998781 -0.129251948 0.075691445 -0.001813994 0.047455938 -0.029846906 -0.040123489 -0.070951968 -0.02294567 -0.057820552 -0.001021768 -0.016857545 -0.015707008 -0.008224119 -0.021403971 0.00488626 0.027455349 0.024676295 -0.109858543 -0.126894391 0.042785371 0.011265611 -0.003354554 0.055911743 -0.007153568 -0.032043725 0.043314498 0.018827767 -0.049810568 -0.02587733 0.05720144 0.031861799 0.033692321 0.068516032 -0.071314826 0.035295972 0.031543415 0.027357048 -0.049877299 -0.003297168 0.037017288 -0.039275893 0.009994298 -0.075237206 0.027420799 0.03597999 -0.063357752 -0.012854292 -0.003475093 -0.012261285 -0.053241951 -0.000469341 0.098497574 -0.001099992 0.004618799 0.092490842 -0.008807183 0.026217521 0.035471453 0.022368685 -0.048305474 -0.022012651 -0.001471271 -0.012701706 0.028788815 -0.011538102 0.068926037 -0.030814841 -0.027810632 -0.034534069 -0.004451398 0.022504964 0.008802978 0.045012299 0.009367138 -0.046850616 -0.022093561 -0.011672729 -0.020647278 0.031909296 -0.026903911 0.017459988

sample_109 -1.227948407 -4.939141108 0.940308796 -0.415106526 -3.999582573 -0.54431919 0.367809018 -0.709874461 0.644765415 -0.250130795 1.374673146 -0.455480763 0.669355698 -0.26833162 1.231608675 -0.508064807 -0.354930607 -0.420924863 0.671346816 -0.279235674 0.846957352 0.182464888 -0.321697828 0.045161983 0.32541794 0.181105377 -0.071497688 0.39174247 0.674298024 0.10544494 0.284487428 -0.168816002 -0.180891886 0.137744304 0.353724059 -0.108432538 0.496709824 0.03283262 0.106363113 0.409762053 -0.0868812 0.082828503 -0.090940947 -0.286542693 0.250612001 -0.144563651 -0.097647215 0.352075601 0.274006644 0.008013552 0.029671824 -0.136077609 -0.052308805 -0.153322556 -0.331647896 -0.089605696 0.01767388 0.250552823 0.076820114 0.070263423 -0.09799011 -0.166880392 0.201492566 0.187603379 -0.219743381 0.166283486 0.369723256 -0.192492736 0.140496986 -0.203380088 -0.006215094 0.087804592 0.00505695 -0.1081591 0.096471184 0.176396918 -0.06251167 0.079838136 -0.024561053 -0.053570137 -0.120358977 0.051841263 0.025360238 -0.023971572 -0.162747696 -0.200738988 0.116336095 0.093434406 0.112790192 -0.206428911 -0.097956556 -0.102164479 -0.033041961 0.016805997 0.126354615 0.008986784 -0.120317842 -0.096996502 0.180726187 0.16173836 -0.068435047 0.06065507 -0.025076771 -0.092657308 -0.14700833 0.059550993 0.02746001 -0.069044665 -0.005641769 0.080976499 0.043256155 -0.058502068 0.081179959 -0.035167373 -0.034492684 -0.014038777 -0.036197432 -0.061659039 -0.023806037 0.087675504 -0.018782612 0.06754115 0.097569167 0.063395059 0.205049601 -0.050352157 -0.064565025 0.036453919 0.034944475 -0.025561195 -0.042257838 -0.074974847 -0.128748262 0.017648719 0.052641799 -0.062227163 0.04271252 -0.061248395 -0.162106388 -0.042379862 -0.245484716 0.005946981 0.017877843 -0.026350402 0.003433243 0.008629475 0.000539936 -0.025275942 -0.106454918 0.039386 0.073127964 -0.045874583 0.047941853 0.082469229 -0.102128894 -0.035770919 0.027847887 -0.015663631 -0.070524029 0.019741967 -0.02110189 -0.04672541 -0.023040039 0.033420203 -0.063022157 -0.015211005 -0.129561625 -0.020986547 0.136076698 0.092135174 -0.005442215 0.118530061 -0.057904033 0.033642521 -0.001069424 0.059686841 0.038493527 -0.071982821 0.046294761 0.114381385 0.024524666 -0.025915889 0.01537138 -0.046229601 0.096096939 -0.035239951 0.075843303 -0.03751458 0.066384502 0.001382521 0.011662698 0.024250086 0.11445157 -0.115386387 0.037158469 -0.073324158 0.032731744 -0.076087391 -0.123249584 -0.056200237 -0.096394604 0.04883981 -0.078358558 -0.059056196 -0.000579548 -0.024902753 0.025560726 0.003600143 0.035049135 -0.041089372

sample_110 -0.903146376 -4.979814828 1.111025137 0.763471047 -1.921837196 -0.741522723 0.953205107 -0.528784214 0.364242685 -0.910542359 0.874950461 -0.101675323 1.279594014 0.142061223 0.236878329 -0.498560124 -0.175264198 -0.160914422 -0.224333228 -0.154129938 -0.136342724 -0.571018373 -0.255135388 0.069120286 -0.036841775 -0.341437002 -0.103217846 -0.154658719 0.036728641 0.078085549 0.136986861 -0.153010685 -0.246603348 0.378047235 0.406563241 -0.284274675 -0.119786045 -0.023578903 0.108368052 0.020746977 0.043407423 0.018280108 -0.12208644 0.04745541 0.016353607 0.205040698 0.242734111 0.355869147 0.049441479 -0.053286938 0.097238187 -0.115949083 -0.160427871 -0.212240792 0.067976505 -0.095978817 -0.113513267 -0.021053443 -0.213694322 -0.045236461 -0.117728683 -0.072247577 0.147605879 0.095910401 -0.022067202 0.080644344 0.019886261 -0.022621366 0.016659233 -0.169006825 0.079902308 0.090702306 0.073053413 -0.183638028 0.083571557 0.031661355 -0.117931849 -0.046495446 0.050827732 -0.034801987 0.034614073 -0.003269587 0.01347498 0.116065696 0.220125565 -0.117069094 -0.030221109 0.129852154 0.062528661 -0.041331944 0.042512808 -0.061256562 0.053183623 -0.08480309 0.072765877 -0.044607631 0.030132069 0.001855713 -0.038870291 0.009032222 -0.097948247 -0.063961782 -0.180233096 -0.106395908 -0.082846461 0.03858787 0.038583284 -0.061014998 0.016258451 0.100157842 0.070528031 -0.050169666 0.043546459 -0.015641609 -0.007927226 -0.139474177 0.035140005 -0.050333736 0.002627977 0.044365645 -0.049966891 0.151413188 -0.031345642 0.076439706 0.003270571 -0.007013009 -0.020551412 -0.068966333 -0.001318382 -0.129611478 -0.03201841 -0.082332355 0.035202524 0.139853788 0.02060799 -0.076477872 0.039460377 0.045164207 -0.036940102 0.006845065 -0.051300772 0.084099168 -0.103313505 -0.073348781 -0.070715707 0.020691741 -0.037151564 -0.040242636 0.002508824 -0.023619943 0.059621617 0.009935027 0.004298449 -0.086284414 0.042208479 -0.009658944 0.008574288 -0.016012345 0.000319464 -0.014522842 -0.067606114 -0.137462601 0.034116427 0.014021212 -0.016541929 -0.054391425 -0.036542381 0.010166491 0.002625948 -0.014186254 0.018674659 -0.003657436 -0.015306201 -0.016698051 -0.004894878 0.029732782 -0.046315547 -0.055306855 0.031358879 0.059239813 0.016427857 -0.035824689 0.013655164 -0.062090391 0.04505953 -0.007289546 0.038309278 0.009212392 -0.027565715 -0.019574664 0.020179793 0.028696723 -0.01138663 -0.027274016 -0.054135914 0.070566078 -0.04230371 -0.035124373 -0.035909029 0.015915711 -0.068132331 -0.034901888 -0.027273998 -0.021083113 -0.020821496 0.019264777 0.025101115 -0.010630502 0.03311615 0.045076246

sample_111 -3.81301164 3.022624565 3.521261612 3.067167341 0.386863282 -0.134347122 -1.797670446 0.337146827 0.166725822 1.208145615 1.279959677 -1.050392888 -0.90504352 -1.093420682 -0.08595297 0.474740164 -0.971098618 -0.06985271 -0.194399412 0.945509609 -0.693283023 -0.160021864 0.168002547 0.215989114 0.217905177 0.636186605 0.220430241 -0.358064394 0.022675406 -0.290500234 0.15800452 0.052957279 -0.911807384 0.330525567 0.001015614 -0.155681664 -0.084696028 -0.023035782 0.15853965 0.615726671 0.020228129 -0.455269027 -0.353431277 -0.455780036 -0.103467004 -0.223825699 -0.174689843 -0.215186028 -0.124346406 -0.174697364 0.166086695 -0.372650446 -0.289382136 -0.051636199 -0.357831409 0.245807484 0.056175784 -0.204396549 -0.238043825 0.17106038 -0.158501183 0.154921818 -0.201174374 -0.347763382 0.189991633 -0.077033711 0.107558207 -0.00169983 0.329666954 0.222243804 -0.100184777 0.250895137 -0.111834856 -0.201403579 0.110393821 0.207717407 0.395431269 0.268163763 0.020862588 0.019638579 0.062575424 -0.12092855 0.260332613 -0.297917721 -0.063707857 0.206094683 0.064446062 0.032085091 0.038177097 -0.041654473 0.049531674 0.309150855 -0.010124692 0.126286433 0.071712336 -0.164407752 -0.016788029 0.092094985 0.089364232 -0.033544984 0.132929127 -0.04811997 0.099058308 -0.088946201 -0.09213824 -0.088691885 0.106574581 0.095058309 0.040367497 -0.12216204 -0.081805275 0.063721017 0.027074914 0.183455282 0.089630411 0.103973945 0.157757174 -0.068034809 0.046743625 -0.119032424 -0.127797583 -0.104709821 0.021862216 0.089949178 -0.042786486 -0.139264599 0.201948505 0.249396493 0.193753126 0.113918967 0.068930459 -0.044007378 0.024035506 0.048230203 0.05831969 0.115338422 0.136326179 0.042314073 0.076264723 -0.115431487 -0.000943159 -0.032606002 0.018860987 0.012345826 -0.002606665 -0.00951796 -0.014865685 0.040750885 -0.041546302 0.026441542 0.121677133 -0.137531535 0.143210294 0.000129556 -0.070004296 -0.184268667 0.066946879 -0.102009677 -0.159061371 0.029920436 0.049883362 0.014807983 0.079570553 -0.077199571 0.067843264 0.03546334 0.021265345 0.007732399 -0.110119907 -0.015886535 -0.066569877 -0.022011818 -0.064678901 0.064545723 0.043487891 -0.020907439 -0.067816433 -0.126264555 -0.035131274 -0.023646346 -0.050775604 -0.029931951 0.036818747 -0.013887587 0.006771897 0.026456228 0.0515867 0.043852031 0.03607999 0.016248783 0.009020729 -0.106100514 -0.026956806 0.063971987 0.015912643 0.021494401 0.058548577 0.063651423 -0.078553036 0.047175709 0.07710859 -0.025929587 0.019279016 0.048335771 -0.013744744 -0.026199811 0.007913409 0.040172225 0.061166675 0.058027653

sample_112 -0.284721351 -3.017822963 1.93875203 1.127089035 1.241838766 0.179868968 1.043486267 0.536686352 -0.305741372 -0.778635375 0.466247928 -0.186611326 1.1545775 0.083757428 0.033676901 0.673950949 -0.482861163 0.235736336 0.162394857 0.199144212 0.155526194 0.041684027 0.03902925 0.228972461 0.023116388 -0.324070581 0.100729014 0.126596478 -0.244679018 -0.078070955 -0.474871874 0.011623441 -0.091213554 -0.075353705 0.195077168 -0.004236477 -0.049662161 0.103815992 0.008973891 -0.246826141 0.014640185 0.088689248 0.106202663 -0.048881282 -0.385616927 0.104221646 0.271028159 0.186479709 0.067433015 0.108404094 0.159900912 -0.272769025 -0.10246363 -0.099354951 0.049136002 0.061348706 -0.085688507 0.105313523 -0.041458765 0.02761239 -0.124744444 -0.132343352 0.021215255 0.061640088 -0.037160303 0.027186624 -0.006139409 -0.095928454 -0.16905939 -0.15624247 -0.057662979 0.05609035 0.081289568 -0.005078481 -0.08310777 -0.133885693 -0.124001114 -0.048593686 -0.053621175 0.015104456 -0.0061967 0.053883578 -0.103788665 0.198297885 0.084660697 -0.060214471 0.142437308 0.104190551 -0.031741621 0.026812826 0.016051964 0.087945191 -0.127733165 -0.161740777 0.133982388 -0.0041073 0.011964973 0.040918984 0.085269248 0.054576203 0.070436136 -0.042969133 0.067685668 -0.055629841 -0.094706854 0.067225377 0.001272988 0.015149812 -0.042951667 -0.018911621 0.027242373 -0.015357364 -0.021377457 -0.031664132 0.014119841 -0.029469875 0.018009261 -0.00020581 -0.015767147 0.093375892 -0.01279264 -0.012005583 -0.050670728 0.00397603 -0.041165572 0.068991593 0.091145964 0.031783457 -0.068503768 -0.05020158 0.008227539 0.025583547 -0.036261939 0.066827161 -0.039604785 -0.056448373 0.03118216 -0.027165075 0.045159165 -0.05560029 0.032403972 -0.001178915 0.008694825 -0.0786264 -0.055999309 0.09108269 -0.027742671 0.030078245 0.036830667 0.06067439 -0.021232713 0.047942457 -0.008310841 0.00177427 0.018741106 -0.023875812 0.031219165 0.037034699 -0.047919352 0.025848397 0.036704734 -0.029483258 -0.00218538 0.037349459 -0.045901117 -0.00038256 -0.069106024 0.014490723 0.002102583 0.016274203 0.036660539 -0.007887436 -0.102557606 -0.006376049 0.003306151 -0.00826689 -0.011716512 0.01869098 0.017686815 -0.012505375 0.059931313 0.063903829 -0.025805424 0.052039284 -0.004941379 0.02861592 -0.016039868 -0.003013148 -0.017740712 -0.010139586 -0.049391845 -0.003386002 -0.012218367 0.024308722 -0.013891014 -0.020070749 0.043556374 -0.007286196 0.026696904 -0.03384178 -0.028904231 -0.001299977 -0.013966673 -0.018328903 -0.02876664 -0.005025039 -0.028702491 0.011165764 0.01162354 0.035045847

sample_113 2.061773574 -3.745334594 0.121844792 0.265655571 -1.967673108 -0.193818159 -0.262989642 1.331142682 -0.022529081 0.947028335 -0.583534568 0.259664706 -1.053662978 -0.380766963 0.764153501 -0.52476297 -0.130640455 0.610732527 0.430586176 0.826831142 0.519028436 -0.152292393 -0.395098615 0.284042624 0.118252728 -0.253493469 0.13292603 0.24487054 -0.211362169 0.881170403 -0.012992217 0.346833282 -0.382384886 0.329300904 -0.345653343 0.384652577 -0.256619368 -0.573875415 0.18922896 -0.218603953 0.072134537 -0.261045911 0.217902121 -0.315164532 0.249490491 -0.321067487 -0.548853691 0.170134238 0.271940263 -0.100769811 -0.14706759 0.025041107 0.005737534 -0.118943327 0.001860512 0.148541181 0.42287486 0.143917612 0.217611181 0.038195804 -0.276004047 0.110562238 0.025520541 -0.004083861 -0.297376474 -0.012262327 0.194869264 -0.078256037 0.151916357 0.342935425 0.073165266 0.005521137 0.203950059 -0.118779779 -0.215126819 -0.10182292 -0.02353774 0.021199977 -0.116115265 -0.015584514 -0.005191925 -0.002975143 -0.055721925 0.023416167 -0.052467311 -0.083220564 0.099995695 0.022063827 -0.260707078 0.120764325 -0.103635778 -0.226582351 -0.056018695 -0.180848045 -0.010034852 0.159872445 -0.194481139 0.035670993 0.120212179 -0.066888805 0.193879661 0.215556963 0.042428714 -0.072456312 0.145481003 0.042361464 0.004807215 -0.008952239 0.153400648 -0.11790271 0.049242815 -0.110615205 0.007164254 0.040417948 -0.109249696 0.089725882 -0.074929787 0.03823028 0.05818345 0.134724035 0.075300634 0.089022855 0.191388886 0.050270172 -0.08475032 -0.004481221 0.177530653 -0.026237478 0.067438391 -0.149359934 0.106285297 -0.092199449 -0.07377256 -0.055757665 0.068193839 0.118284841 0.11312713 0.067018592 -0.137938478 -0.034002419 0.008958159 0.054320601 0.009937952 -0.025460683 -0.004062837 0.001856657 -0.076895168 0.020696742 0.089418185 0.146363477 -0.09523695 -0.034312252 -0.107761933 -0.03078941 -0.057904643 0.063659241 -0.013516893 -0.070744503 0.020368314 0.049264804 0.090355045 -0.006401675 -0.033202967 -0.01085384 -0.048011406 0.040384473 0.001414943 -0.089053552 0.107717363 0.088703099 0.078976817 -0.053022334 -0.016595345 0.049185871 0.053030387 -0.026355994 -0.036739806 0.043585432 0.013571515 -0.070019544 -0.012652423 0.002945887 0.099643374 -0.002854913 0.035679152 0.06677875 0.012252224 0.01814089 -0.031480977 -0.05811023 -0.013575166 -0.102641669 0.052863024 0.054121449 -0.05205122 -0.017527962 0.053646956 0.041179414 -0.045537107 0.003518115 -0.019054135 0.004760397 -0.062804808 0.004006857 0.011430469 -0.020085698 0.007499879 0.015166514 -0.030339326 -0.131419152

sample_114 -6.37980992 0.790136225 0.524638087 1.31729642 2.080920249 -0.627368466 0.182317287 -0.00820099 -0.120967421 0.362072832 1.042993048 0.442981812 0.618162491 0.993395149 0.15479497 -0.520658116 0.161314287 -0.109194372 0.128837131 -0.785679564 -0.068715286 0.391357916 0.068614305 0.158565755 -0.039604293 -0.059659464 -0.395322112 0.303958093 0.09415543 -0.027965046 -0.112656083 0.046933506 0.145587703 -0.41648854 -0.303681963 -0.1509674 -0.475620169 -0.314222589 -0.301709411 -0.244164541 -0.171596998 -0.246027789 0.058019991 0.241594347 0.109701172 -0.085083753 0.111141552 0.255064747 -0.051477219 0.176653962 0.034438349 0.002301436 -0.123097287 0.003830564 -0.028292994 0.188298269 -0.077863712 -0.084337125 -0.123596997 -0.222487789 0.162013754 -0.30139886 -0.038824309 -0.05456511 -0.207128603 0.175980062 -0.135640852 -0.095800728 -0.056962252 -0.275835926 -0.085678369 0.148958976 0.04190296 0.010480816 -0.072926835 -0.344754327 -0.082440771 0.113778796 -0.016106203 -0.012883712 0.026523825 0.104847101 -0.039605339 -0.038003192 -0.010760108 -0.130757705 0.011748159 0.01490453 -0.073136746 0.182606775 0.106549456 -0.050052405 -0.086132144 -0.051483873 0.164120687 0.03379635 0.001535324 -0.136199797 -0.045787822 -0.048455802 0.051765975 -0.10767452 0.05068294 -0.12311175 0.007871917 -0.044951704 0.073283726 0.208213029 -0.065765365 0.042028952 -0.113812044 0.073126346 -0.071332009 -0.05047018 -0.051507924 -0.010851372 0.019541022 -0.001895822 -0.043089283 -0.026525075 0.110859268 0.015308542 0.033561368 0.017800319 -0.067261795 -0.080932737 -0.039604282 0.048607008 -0.132538317 0.078619126 0.057827606 0.020202604 -0.045272647 0.008715249 -0.03521648 -0.043328144 -0.025186396 0.013100847 -0.008143012 -0.072748074 0.067537888 0.029681032 0.075952073 -0.072806315 -0.02265107 -0.086333443 0.092851779 0.07205612 0.031670672 -0.021776804 0.009449021 -0.011697124 -0.060952594 -0.063855889 -0.112249221 -0.041360727 0.038155419 -0.025336057 0.04525132 0.037881593 0.037303697 0.021223856 -0.059176129 3.09E-05 0.064631995 -0.011097802 0.012572642 0.03281698 0.014245596 0.102110227 -0.00668295 -0.046446483 0.012912861 0.035089291 -0.040636245 -0.033350997 0.00819336 -0.001873944 0.002435957 0.001800669 0.006737957 -0.029403444 -0.009152271 0.004013213 0.006306662 0.004925941 -0.08934634 -0.023459675 0.016972762 -0.017551934 -0.024552414 -0.041469784 -0.001716159 0.057795444 -0.032830502 -0.053186832 0.027167168 0.03953367 -0.01171535 -0.052114201 -0.031391373 0.0018393 -0.019139544 -0.010318205 -0.009082835 -0.00149146 -0.057576109 0.062987361 -0.00708915 -0.003684388

sample_115 -5.39799769 -0.849688465 0.83283005 0.531792883 -2.322428397 -0.707528526 -1.869224136 1.188484149 0.190889952 -0.766766392 1.007027221 -0.492298605 0.673633166 -0.700097377 -0.908961621 -0.140337921 0.91200546 -0.205428112 -0.508162628 -0.698337055 -0.200922309 0.10604466 -0.192244264 -0.490832936 -0.548356755 -0.076933598 -0.19424616 -0.018607551 -0.318754748 0.169335731 0.30012325 -0.578635087 -0.636719249 -0.000309465 0.037771641 -0.468180728 -0.399606978 -0.049174837 -0.71964221 -0.328746229 -0.062928119 -0.251607418 -0.240616785 0.108803811 -0.148655003 -0.010711256 -0.066408689 0.829209857 0.359287763 0.104158741 0.142347065 -0.259448223 0.0719193 -0.239657298 -0.421733385 -0.119423059 -0.324087183 -0.265783838 0.154341275 -0.068171074 -0.033897946 -0.078883686 0.145548337 -0.338392312 0.119890265 0.055513818 0.09235277 -0.160302459 0.147912086 0.017872898 0.270331697 0.096563466 0.160359782 0.01060808 0.060386511 0.055847686 -0.042296853 -0.041036923 -0.130201107 0.038272532 -0.136441444 0.047993059 0.270833616 -0.024614935 0.066147091 0.019312023 0.073746796 0.318006858 -0.087268566 -0.096553915 -0.166795354 -0.028669993 -0.259012421 -0.127237665 0.202842857 -0.032481493 -0.183134148 0.148910988 0.071336838 0.173423351 -0.111758651 0.193262439 -0.370869226 0.055478662 -0.114448975 -0.177679371 -0.091190213 -0.043147045 -0.004148745 0.044428939 -0.225875301 0.092078654 0.014527415 -0.032913808 -0.12664789 -0.113428079 -0.02473676 0.003590598 -0.081636994 -0.095141315 0.125518935 -0.013413256 0.013644318 0.04432051 -0.01031401 -0.157032288 0.042427191 0.10190816 -0.14458411 -0.001594235 0.123349939 0.106900595 -0.028556395 -0.05940189 -0.034485325 0.039687301 0.013689013 -0.123034337 -0.064808926 0.006921444 -0.152206789 0.036746428 0.003301257 -0.059457315 -0.074784559 -0.077794129 -0.038955735 0.030013193 0.023232428 0.011930338 0.039899818 -0.016801353 0.155426704 -0.13949748 -0.243103397 -0.021726728 0.110266247 -0.04661075 -0.06482264 -0.129726169 -0.059052176 -0.045272777 0.058223162 -0.143763104 0.002093041 0.075521921 -0.020904317 -0.036072479 -0.011093755 -0.150100256 -0.069918868 0.056014997 0.008154632 -0.045503695 0.057285854 -0.141564168 -0.021173249 -0.190236016 0.031333313 0.084424937 0.009673608 -0.051803805 -0.06794731 0.026382928 -0.040757366 -0.032561279 -0.01806558 -0.075018231 0.167845052 -0.042537987 0.02420938 -0.033987915 0.153354663 0.089895289 0.031328587 0.072077195 -0.096555484 0.019170431 -0.013975717 0.09363306 -0.090638432 -0.035844124 -0.141456968 0.061795428 0.019621325 -0.000857611 0.071494833 -0.07104252 0.008716189 0.057305049

sample_116 5.945431604 2.84343585 2.406424287 -0.812933937 -0.659567853 -2.029302383 -1.342494157 0.672225593 -0.576810538 0.533603565 -0.460286118 0.136179419 -0.112722555 0.885971494 0.989152961 0.717494521 -0.854448517 0.253529173 0.733505724 0.882527298 -0.340571587 0.597383448 -0.061483636 0.272370561 0.068786197 0.070058925 0.34843068 -0.427020773 0.289967583 0.201877988 0.082310586 -0.140172645 -0.43411596 -0.756639671 0.610725792 0.039522838 -0.172637301 -0.15202453 0.112296451 -0.063599445 -0.211874303 -0.314294209 -0.140984126 0.105040895 -0.437737432 0.157358788 0.300759368 0.056252409 -0.288002511 0.121350469 0.319346968 -0.034948746 -0.181561131 -0.099953576 -0.190074202 -0.101686604 -0.001987149 -0.220332758 -0.095628459 0.023085598 -0.029194052 0.172035721 0.157608782 -0.03828211 -0.150720269 0.05048284 -0.307657665 -0.00582329 0.32206309 -0.009065654 -0.208592313 0.218412491 0.22074858 0.073538103 0.080447128 0.022199939 -0.15475821 -0.010924047 0.029131289 -0.028283651 -0.086004889 0.019136461 -0.033197696 0.029269147 0.179293365 -0.082672204 -0.049315912 0.119049713 0.071195911 -0.100112723 0.006427189 -0.042030161 -0.059987682 0.06911661 0.081407589 0.028617997 0.068287438 -0.168878739 -0.075725325 -0.033247692 0.003271682 0.098014735 -0.077154838 -0.033608911 0.133010151 0.017310188 -0.068713941 -0.145424094 -0.034703594 -0.021197827 0.198664149 -0.030649985 0.098032089 0.004447005 -0.043704239 -0.016872134 -0.088604335 0.068191924 -0.077178399 0.078523957 0.005985379 0.035697059 -0.034564441 0.085114841 0.066490899 0.00949498 -0.032480355 0.045355511 0.085186244 0.028861411 -0.039241364 0.06120292 0.047931371 0.052234771 0.092654367 -0.034212741 0.076393733 0.027963063 0.014483173 0.049560955 0.000871279 -0.032299188 0.012519673 0.017807651 -0.039798169 -0.012519344 -0.016683169 0.017787138 0.077449254 0.060523976 -0.044709519 0.060769006 -0.013806384 -0.049948899 0.05149168 0.020901813 -0.117290086 -0.019686588 -0.105783194 -0.057368101 0.01455192 0.035349085 0.041507173 -0.001859055 -0.028682363 0.016158173 -0.033894093 -0.020218293 0.091560332 -0.092399917 -0.023206362 -0.067463931 0.013536358 0.011630485 -0.01912924 -0.093631063 0.025535611 0.055331165 0.007426146 -0.090661922 -0.000680926 -0.028301101 -0.06507587 0.039902295 0.000860532 0.053575933 0.080259012 0.032854351 -0.023257561 -0.029976499 0.067797327 0.02591622 0.008601647 0.029037933 -0.024976577 -0.02372022 0.076591577 0.017700269 0.058629399 -0.046522426 -0.004189931 0.018967555 0.054430106 -0.017144213 0.042315572 -0.020752179 -0.08366843 -0.038822794 0.005009053 -0.033089668

sample_117 -3.046026002 0.130041429 2.862954418 -0.142483744 1.743243946 0.46916915 -0.184423627 -0.131347468 -1.334992843 -0.228145795 -0.316668042 -0.260185816 -0.198310764 -0.052471684 -0.29432752 0.939178392 -0.416878732 -0.195123777 -0.727824446 -0.688362247 0.24941102 0.478381428 -0.094630997 -0.522449789 -0.018486769 0.406502825 0.683474681 -0.450150241 0.313919268 -0.470020898 -0.452096816 -0.097793754 0.024000524 0.076754972 -0.509180393 -0.292761078 0.354511232 -0.012457733 -0.300541614 -0.029199288 -0.215980135 0.152036901 0.148269526 -0.149331136 0.126289465 -0.192034991 0.336454285 0.352973353 0.027879197 -0.130420374 0.111957914 0.074669318 -0.350706915 0.111572249 0.305804312 0.120562111 -0.038488627 0.009427376 -0.169247592 0.056668571 -0.029021683 -0.046894088 -0.313905141 -0.162352124 0.110303091 -0.036091461 0.019740289 -0.060341126 0.126668292 -0.048545142 -0.089887062 -0.015277699 0.138126501 0.018482275 -0.149986815 0.078968206 0.024588047 -0.225348669 0.152649443 -0.109018245 0.092127451 0.180075604 -0.063000385 0.142615831 -0.00595397 0.146978411 -0.137137761 -0.048800266 0.013624775 -0.115498514 -0.03810044 0.056165031 0.172083151 0.059384036 0.037838045 0.176661289 -0.035019764 -0.032625161 -0.025130667 -0.027876764 0.001467728 0.064409863 0.164929972 0.005495674 -0.051797531 0.030093322 -0.0530322 0.105810485 -0.174027853 -0.05456599 0.04095808 -0.027968162 0.171614493 -0.023106349 -0.023108867 -0.00903245 0.130879244 -0.114899553 0.035583416 -0.077962071 0.015741516 -0.068848138 0.060485508 -0.060431407 -0.062349397 0.104140787 -0.032785596 -0.032993109 -0.047174199 0.037177876 0.165050019 -0.074947156 0.026545158 0.038088441 0.072383434 0.01972879 -0.060896879 0.03999311 -0.019928043 0.054661168 -0.029627146 0.142684026 0.011669944 -0.089035195 -0.05681658 0.043483145 0.043845222 -0.026488917 -0.006680635 0.053980269 -0.058549718 0.102159057 -0.063933061 0.124600161 -0.022642142 -0.013647134 -0.108542704 -0.001889648 0.048432403 0.046574181 0.05745938 -0.078350713 0.066803471 -0.048924449 0.024101779 -0.005243331 -0.013585327 0.112626149 -0.002761291 0.045918352 -0.056804694 0.100715256 -0.034164039 0.018634303 -0.064198829 0.023785142 -0.022019 -0.022327391 0.023333237 0.007019892 -0.039933312 -0.01689175 0.036311336 -0.078586823 -0.054834828 0.048215282 -0.002029838 0.029563111 -0.003331212 0.024712419 0.009984269 0.004742552 0.04947917 0.007577085 0.037614125 -0.047951849 -0.050492217 0.011415948 -0.000510522 -0.022531826 -0.020935929 -0.026963239 -0.013750671 -0.003325055 -0.027242992 0.011803211 0.017289305 0.05860709 -0.028744533 -0.041274158

sample_118 -6.099968387 0.463766466 2.028619878 2.363494381 -1.261316243 -0.780719924 -3.261225547 0.071569096 0.870144331 -0.324838085 0.368376394 -0.782397075 0.522741871 -1.842794057 -0.531072184 -1.128659026 0.374779989 0.723869265 -0.175485797 0.302851162 -0.143631378 -0.693552735 -0.98702903 0.104529859 -0.386617781 -0.326592345 -1.189118119 -0.568379469 0.25214896 -0.28750206 0.598679983 -1.249979634 -0.171230226 0.041964914 -0.153814725 0.041789528 -0.378127932 0.634861459 -0.5893566 -0.138770721 -0.141771428 0.195325496 0.370120446 -0.098258429 -0.028737662 -0.066203306 0.527855627 0.335592938 -0.193094739 -0.281069662 0.729539189 -0.490061104 -0.140875414 -0.342855024 -0.134211647 -0.102138845 0.401165554 -0.113935728 0.386714873 0.004520284 0.376259101 -0.254862905 0.40804647 -0.099995049 0.113813716 -0.042511579 0.114269474 -0.030611568 0.059223235 -0.134125633 0.063751676 0.366335954 0.406285902 0.068991391 0.52220205 -0.45437196 0.273353558 0.296202797 -0.179838627 0.349268261 -0.268220869 0.153490487 0.253627594 -0.116868761 -0.353034644 0.27218423 -0.081401528 -0.108663434 -0.128775745 0.15471672 -0.523617587 0.624970792 0.065917388 0.024240863 0.416727427 0.070355291 -0.216913512 0.081888 -0.039992888 0.115306213 0.002855501 0.07421887 -0.396751427 0.097341644 0.093062794 -0.187391171 0.287943908 0.055541994 0.143810828 -0.058519995 0.134669246 0.205880521 -0.079523775 -0.343709048 -0.148768243 -0.096102815 -0.058151147 0.098712734 0.044717665 0.214105174 0.04662382 -0.018969197 0.004508501 -0.064186707 0.066663301 -0.234000393 0.172474447 0.306358793 0.138543517 0.013952481 0.14339731 0.211706022 -0.134626664 -0.016303787 -0.252900961 -0.050978675 -0.003859628 0.0197613 -0.061723295 -0.066454173 0.028957399 -0.17135589 -0.033612565 -0.052805773 -0.118045163 -0.066032151 0.03308708 0.086932311 0.044256921 -0.006941158 -0.182959823 -0.132338985 0.032789705 -0.239706744 -0.070085341 -0.049548446 0.144737055 0.115990457 0.037236373 -0.148558136 0.118090873 -0.047552251 -0.054506797 -0.044705433 0.095340925 -0.138049663 -0.177319911 -0.041250652 0.118787123 -0.069332246 -0.053389996 0.017496619 -0.02418656 -0.133088783 0.09562525 -0.074563267 -0.014029556 0.068969251 0.017839017 -0.034063176 0.172497347 0.044836698 -0.023794661 0.095674925 -0.13633773 0.019321106 0.017827336 -0.081033336 0.223140233 -0.015998493 -0.042271209 -0.087232999 0.081325916 0.137754873 0.156100083 0.027317382 -0.060289391 0.014102075 -0.02321313 -0.017396251 0.05275743 -0.067534849 -0.208635764 0.08395293 -0.032707056 -0.098878593 0.045088438 0.000674072 0.09717241 -0.021355185

sample_119 -6.753647169 3.203385545 2.025433417 4.54813135 1.498740532 -0.878119289 -2.298369696 -0.679293946 0.29976365 1.937168591 1.219843761 0.64944244 -0.382830462 -0.613184129 -0.440796496 0.211680458 -0.8207495 -0.323263575 -0.476211662 0.688517189 -1.033724708 0.013086997 -0.153263433 1.33278319 0.576368082 0.570840174 0.338759781 -0.03669786 -0.397780617 0.251069961 -0.284488916 0.286705836 0.635010209 -0.144325674 -0.482878739 0.365832397 0.08073948 0.316483753 -0.150542629 0.401044368 0.0130954 -0.830480166 -0.426506464 0.049335082 0.226636787 0.069782575 -0.697126442 0.05427934 -0.15070119 0.694922952 0.208479254 0.057483653 0.032882043 0.187877834 0.121462615 0.45074378 0.320776913 -0.115417572 -0.421467012 0.095972771 -0.129962299 0.191435128 0.173951174 -0.114107174 0.110019824 0.47528042 0.137278293 0.187874573 -0.07233696 -0.152238634 -0.170889083 0.289567874 -0.222965222 0.030704557 0.073764798 -0.134800977 0.642817728 0.242735012 -0.171008228 -0.131956447 0.03322027 0.065766576 -0.036604104 -0.030461944 -0.046998023 -0.036673952 0.031545606 0.095771432 0.254763056 0.151231153 -0.045492648 0.109290554 -0.212738547 0.028182756 0.111733701 -0.071312898 -0.149940401 0.06502518 -0.011658126 0.285312523 0.296150402 -0.04037135 -0.375951253 -0.290737548 -0.192846837 0.036994583 -0.002988114 0.170616096 -0.061446296 0.057510427 -0.111759958 0.184800907 -0.19152944 0.042943555 0.152772809 -0.194245052 0.029781456 -0.079461726 0.034060836 -0.122210605 0.282781284 -0.126094433 0.227935726 -0.083997162 0.328003415 -0.090215296 0.190589233 0.056586591 0.181306351 -0.024292727 -0.048589938 -0.156990661 0.072889135 -0.055851945 -0.196877772 -0.031362572 -0.032024691 -0.060064195 0.241163904 -0.251036424 0.189048527 -0.015816116 0.063405123 0.00610104 -0.150523808 0.013222108 -0.168222334 0.02780242 -0.072096985 -0.106457736 0.039293487 -0.054717482 -0.030498002 -0.118592574 -0.009807852 -0.039405591 0.040652021 -0.065666115 -0.000381612 -0.112481291 -0.081246814 0.04980718 0.027424765 0.088520786 -0.004610972 -0.009270804 0.116968452 -0.009230604 -0.093518894 -0.005623005 -0.090698367 -0.036834044 -0.160117191 0.063140423 0.000197134 -0.085138494 -0.02519001 -0.054304031 -0.065755556 -0.11233146 -0.015527373 -0.150348139 -0.009425858 0.049083372 0.047765878 0.087830745 0.059203277 -0.081179837 -0.042662975 -0.045969842 0.16208116 0.043994673 -0.048874979 0.005072897 0.001164961 -0.100043905 0.059348711 0.078213342 -0.100366406 -0.028386283 -0.038581436 0.001313637 0.014487205 -0.00683333 -0.102468942 0.058536619 -0.167147783 0.026968572 0.042664144 0.145678812

sample_120 7.171814229 0.310820342 -0.906010004 -0.848589266 -1.713301011 -1.576943397 -2.215721548 -0.822365832 0.497726246 -0.857029485 0.085642828 -0.733798723 0.263740414 1.278652189 0.443463736 0.300630025 -0.158923608 0.373942813 0.999997521 0.094510172 -0.326353458 0.3522771 -1.01600843 -0.135190965 -0.404298571 -0.565857631 0.350504392 -0.150547903 0.146840704 0.089543647 -0.486004344 0.090737769 0.067453989 -0.544920945 -0.107053931 0.295154216 0.318828107 0.168647058 0.290819598 -0.048762622 -0.129320906 -0.18423286 -0.084917703 -0.096336181 0.355824677 0.039791911 -0.289855027 -0.056052491 0.295842707 0.145796732 -0.077975187 -0.373066956 -0.410634366 -0.416159603 -0.040561968 0.240314163 0.126967683 0.010118385 -0.113069138 0.060508578 0.219941309 -0.241522817 0.064505484 -0.094678217 -0.003590978 -0.033710883 -0.017785584 -0.042530773 -0.044667534 -0.059993727 -0.108423758 -0.148247732 0.059243076 -0.164844969 -0.094412776 -0.067068792 0.005782858 0.090291765 0.234293221 -0.048398229 0.147937645 -0.06326133 -0.159901423 -0.148343167 -0.110715455 -0.092661713 -0.148209445 0.046948332 -0.103939396 0.110894592 -0.034817997 -0.074042776 0.08269108 0.108972303 -0.257123609 0.019269295 0.052237281 -0.113053371 0.092830445 -0.170607712 -0.001699997 -0.039582044 0.025445187 0.135631033 0.029616919 -0.020491773 0.005401149 0.025792071 -0.010455638 0.028833794 0.099594728 0.075986087 -0.038413013 0.037374182 0.01087519 -0.043277326 0.055440524 0.055594625 0.014281017 -0.034730692 -0.129796307 -0.058030471 0.017921056 -0.051006665 0.03822531 0.037193102 -0.061064204 -0.051629203 0.079365645 0.066953125 0.081087778 0.049672228 0.11890656 -0.025063242 0.036652877 0.047858224 0.062851638 0.014175802 0.121289736 -0.005411851 0.061561529 -0.037130878 0.093126062 0.021996357 0.075321924 0.04392154 -0.027585067 0.037885479 -0.020245762 -0.040724286 0.059520674 -0.018078302 0.013233922 -0.025785808 -0.034250452 -0.013956826 0.037068896 0.019973347 0.042640832 0.027414363 0.041650294 0.011732924 0.03791637 0.013733065 -0.019344681 -0.024744944 0.06168672 0.019837659 -0.063361397 0.005435443 -0.005779718 0.094907465 -0.009537332 0.003490389 -0.109932177 -0.081266259 0.069088384 -0.044139956 -0.016911854 0.009149382 -0.015026779 0.0522122 0.060062215 0.014090367 0.013591655 0.038870195 0.041894272 -0.047892648 -0.018012565 -0.018637218 -0.024209064 0.032065635 0.027703781 0.024685457 0.039939231 0.021795376 -0.005267783 0.038412563 -0.029226045 0.008262702 0.046972377 -0.028986755 0.037131707 0.017036332 -0.026019433 0.000632982 0.022730639 -0.057541042 -0.019875274 -0.047282162

sample_121 0.0893533 -2.408092588 -1.419309391 -3.750102892 -0.154208604 2.400718694 -1.20596813 -0.598459562 3.018977386 -0.149581416 -0.349872801 1.043132321 -1.414161421 -0.168903922 -0.762783566 1.121077087 -0.120873698 -0.115973306 0.086601109 -0.606761603 -0.074666084 0.337429551 0.021901213 0.364986049 0.08408281 -0.169764797 0.105519147 0.011062008 -0.475204716 0.294164628 0.399267156 0.11228167 0.210414059 -0.347040402 -0.015939418 0.449398695 -0.007446892 0.522871245 -0.198508671 -0.025084464 -0.254339896 0.28785879 -0.396875178 0.071624636 -0.397384765 0.106009235 -0.103512064 -0.042623256 -0.282201138 -0.285168782 -0.097078572 0.104752393 0.067704034 -0.171481566 -0.243156176 0.048584247 -0.176366075 0.151765593 0.051526179 -0.015785781 0.166604124 0.243670263 0.028153639 -0.126748776 -0.151262851 0.183995233 0.136352532 -0.171263718 -0.046238771 0.235020251 -0.011994205 0.204187685 0.049886446 -0.137596956 -0.037087481 -0.103353468 -0.048483277 -0.04208788 -0.04059363 0.270286385 0.108451527 0.037617266 -0.014593671 0.047582503 -0.149137914 0.095979232 0.062621927 -0.093348533 0.087887884 -0.026381686 0.106029905 -0.0639872 0.049211234 0.057176307 -0.111963297 0.076927354 -0.039522127 -0.178187204 -0.146020283 -0.005125747 -0.040105569 0.004893287 0.000931612 -0.034055059 0.117576029 -0.031947374 -0.046100072 -0.036210015 0.125515781 -0.020066212 0.02210432 0.016475203 -0.057341108 0.012917266 -0.021449861 0.172623911 0.141038057 0.068066112 -0.003510256 0.113855163 0.071700115 0.018976947 0.067765099 -0.038858891 0.002305851 0.020332063 -0.005590959 0.066289738 -0.124451673 0.106205626 -0.032462803 -0.013909965 -0.023674335 0.039954395 -0.080621297 -0.048109184 0.011825431 0.125842353 0.012631409 -0.069278576 0.074996635 0.051836965 0.03353664 0.004462176 0.028747576 -0.061120483 -0.007440739 0.064262258 0.115891614 0.033858888 -0.120343894 -0.005993658 0.09334497 0.030071619 -0.106010364 -0.059962156 -0.01535246 -0.051614581 0.053437143 -0.002723281 0.004626811 -0.12835966 -0.040914301 -0.021146936 -0.010235578 -0.096554592 0.043913693 0.02014935 0.073391039 0.036591155 -0.001729201 0.003593047 -0.005489902 -0.039880972 0.049645724 -0.075770078 0.036888017 -0.043532877 0.010930657 -0.037994858 -0.01158776 0.06198789 0.089900964 0.054884074 -0.041329743 0.046447106 -0.028608001 0.007256073 -0.028323798 0.054837716 -0.077110639 -0.061051582 0.033116866 -0.006561554 0.073188781 -0.027466274 -0.010234764 0.001582036 0.015872188 0.002771335 0.020995366 -0.060064231 -0.101279831 -0.033300626 0.008801181 0.027238707 -0.018338012 -0.017417618 -0.011434191 0.053805027

sample_122 -0.265630591 -4.074735378 -2.891449057 -1.468943325 -1.953369111 2.349094839 0.215021754 0.326752929 1.037099122 1.541313557 -0.060662541 0.073179857 -1.106355932 -0.122198141 0.565767099 0.153058089 0.924081106 0.037875692 0.823900074 0.265675577 -0.150063744 0.672761525 -0.046848622 0.414526057 0.600810452 0.456415308 -0.236210653 0.380273669 0.509741734 -0.244002017 -0.358886078 -0.185748324 0.216073936 -0.441869822 0.03640761 0.125128122 -0.074900796 0.076771424 0.290829634 0.17044808 -0.373399315 0.05119473 0.371582238 0.072475711 0.103972782 -0.147743452 -0.301906462 -0.131063111 0.070866105 -0.03744792 0.2145252 0.052223319 -0.362387914 0.176187037 -0.064826534 -0.053508278 0.031598785 0.344674386 -0.061010803 0.019225599 0.008376627 -0.154932449 -0.100645066 0.368753921 -0.126321163 -0.164383363 0.118753937 -0.262328735 0.176834711 0.124682727 0.049149845 -0.051083061 -0.118374857 -0.045141324 -0.179354247 0.199764101 0.078071954 -0.053409902 0.044590951 0.053043534 0.062648336 -0.235538714 -0.069835413 -0.004823523 -0.227836395 0.087021963 0.150069082 0.047022539 -0.074079278 0.205648473 -0.142664939 0.139380828 0.031091972 0.008427545 0.016934835 0.036181801 0.083718518 -0.077436983 0.175534648 0.123285563 0.096421691 0.025220364 0.005183472 0.02749599 0.067412217 0.08523227 -0.085482187 -0.178770013 0.052018679 -0.052396835 0.113073758 0.01309346 -0.029180267 -0.090654475 0.077671962 -0.173857543 -0.098816092 -0.014728324 0.027791643 0.122200279 -0.052910653 0.177853341 0.115000507 0.048844985 -0.003458867 0.008644422 -0.039303237 -0.00985863 -0.108363177 0.069107129 -0.084975238 -0.053673872 -0.002296959 -0.154871854 -0.222237263 0.099204621 -0.012362327 -0.040757966 -0.152725521 -0.084116176 0.10844867 -0.02851462 -0.021457204 -0.162822475 0.022246617 0.176594194 0.000961736 0.052873933 0.089492214 -0.087511526 -0.154464968 0.007129437 0.046864608 0.056022626 0.022063817 0.025600287 0.100744343 -0.034598505 0.007642258 0.03051533 0.065870003 -0.051804516 0.037990071 0.063023776 0.045505392 0.019294342 0.016534115 -0.126367963 0.046455716 -0.138195219 0.091447148 0.102490959 0.054707846 0.067080001 -0.036825349 0.024669438 -0.001940472 -0.040877593 -0.058196639 0.140494022 -0.091036431 0.123921849 -0.007231806 0.074656592 0.038941872 0.118043276 -0.024150583 0.090263681 -0.020663795 -0.003770124 0.015220753 -0.059578979 -0.000580157 0.011443797 0.014265866 0.004445288 0.031204088 0.021386266 -0.006644113 0.012372215 -0.01633028 0.104849373 -0.022313545 0.072219736 -0.019850394 0.015871426 0.035103508 0.064349137 0.000291525 -0.049340849

sample_123 -6.249418595 2.333846312 -1.501801517 -0.223093771 2.941784767 -1.498892345 0.736456892 0.649216145 1.083321232 0.970222243 0.546352238 0.412794442 0.791181719 2.333832992 0.472474291 -2.020759019 0.043598766 0.845209544 0.252397133 -0.242735465 -0.29072094 -0.410964885 0.20289205 0.181460159 -1.059591009 0.613466482 -0.651654222 -0.274685564 -0.55822438 -0.01550204 0.314936364 -0.005435196 0.257519913 0.330315563 0.37768509 -0.016913142 -0.187292355 0.459481152 -0.049763404 -0.34773817 -0.03256395 0.138046995 0.149343515 -0.053711765 0.483159029 0.338884795 -0.093270234 -0.03791876 0.001753841 0.362490751 0.390142177 0.107107082 -0.194197875 0.170979124 -0.352336539 -0.281324969 -0.014289349 0.255005441 0.191313266 0.020406411 0.326921551 -0.191598265 -0.245928141 0.09968286 -0.199312417 0.010188074 -0.008952048 -0.20580011 -0.248293233 -0.157066371 0.0818958 0.014875061 0.223214493 0.038009023 -0.156587526 -0.073525216 0.009636019 0.123908601 -0.083123848 -0.002681204 -0.043726894 0.164976994 0.136464974 0.097636919 -0.282348554 -0.105375789 -0.070088744 0.063413168 0.11965047 -0.108204415 0.113608638 -0.178680049 -0.038709895 0.066935156 -0.17036936 0.201877032 -0.144053762 -0.307093852 -0.108764951 -0.042169327 0.071133323 -0.064626695 -0.011185269 0.074111381 0.124393122 -0.211566731 -0.024928348 0.099862048 -0.228926034 -0.057726763 0.042201456 0.037692794 0.05768985 0.088698617 -0.016310503 0.094419711 0.041080433 -0.108597002 0.035417163 -0.165367635 -0.044310177 -0.007274414 0.138868358 -0.10986419 0.060081959 -0.070959171 0.072214202 0.019865268 0.054225622 0.17571087 -0.222288928 -0.093812655 -0.004206072 0.172223111 0.059806938 0.146760677 -0.047374514 -0.001326761 -0.067205328 0.028440459 -0.072965302 -0.125197991 -0.060246188 -0.010958987 0.053228206 -0.040407267 -0.068277485 0.060690633 -0.141572031 -0.002696966 -0.091164099 -0.016957366 0.113432508 0.005775803 0.08720523 -0.012309849 0.043848573 0.125342122 0.087748718 0.068570869 -0.047659579 -0.085003212 0.045903558 0.005667775 0.018088181 0.009188931 0.081889915 -0.093106464 -0.014569315 0.019869727 0.023281754 -0.043133859 0.05776326 -0.050696477 0.040424273 -0.035099136 -0.014899127 -0.052957892 0.016934214 0.047836063 -0.033299787 -0.092310598 0.001340264 -0.064076722 0.034371451 -0.061393133 -0.010455692 -0.033605763 -0.030934632 0.047462986 0.064437815 -0.00664302 -0.097218029 -0.010440513 -0.003771868 -0.06780111 -0.009446896 -0.007191309 0.034645936 0.038207593 -0.016413409 -0.04180928 -0.067325099 0.051416025 -0.050168127 -0.015844303 0.015816238 -0.044365119 -0.004325555 -0.008525735

sample_124 -5.677202063 -2.185633661 0.9680181 1.441157629 0.899139592 0.149543607 0.232354916 -0.044013851 0.052783551 -0.784003861 0.831166446 -0.669330743 1.580340933 0.266763523 0.284948513 -0.464709653 0.570392797 0.105200886 -0.390781215 -0.658956587 -0.253167216 0.014759401 -0.081144479 0.319438332 -0.245753301 -0.534763325 -0.699715977 -0.237813635 -0.157907426 0.135849266 0.150020161 0.005080786 -0.075459943 0.080928214 0.044944317 -0.355804339 -0.687901557 -0.027306717 -0.104770638 -0.085658222 -0.253650698 -0.009044394 -0.229124181 0.373074208 -0.203000772 0.315260522 0.369907002 0.305576854 0.114483588 0.091773082 0.042473336 -0.067480342 -0.013809921 -0.181926515 -0.026016785 -0.162026067 -0.012835424 0.056318589 0.008404238 -0.167177005 0.014397329 -0.11896576 -0.044454508 -0.044832757 -0.259174879 -0.003952585 -0.032131923 -0.019796079 -0.028498947 -0.028433784 0.064448228 0.112218896 0.134144754 -0.094905445 -0.178076951 -0.003309383 -0.098351869 -0.01507204 0.03631962 -0.065655403 0.077913255 0.047382471 -0.070724698 0.016560663 0.142305065 -0.280367544 -0.191059708 0.115954563 -0.08605347 -0.001112833 0.051054307 0.067700271 0.001532509 -0.080669712 -0.055832158 0.081278955 0.043655355 -0.016679267 0.056147869 -0.017562933 0.04728579 -0.239155736 -0.123196734 -0.124341719 0.076081773 -0.062443311 0.057315166 0.062181247 -0.030625587 0.027215952 0.038863151 -0.16617798 0.002662346 -0.008279164 0.006768556 -0.014111808 0.048481878 -0.064369766 -0.02601243 -0.00652938 -0.046153966 0.07894474 0.023169978 0.032270757 -0.033249309 0.051975098 -0.092489763 -0.058610494 -0.013565479 0.049759111 -0.070918779 -0.062697332 -0.027926664 0.067544983 0.078233406 -0.071235838 -0.050301159 -0.000398982 -0.057601405 0.083812231 0.050888688 -0.018754416 -0.04121071 -0.091954853 0.018735952 -0.015540672 0.03227384 0.085306073 -0.054494318 -0.085629089 -0.049915129 -0.010780695 0.044337902 -0.047631987 0.071916276 -0.018148773 0.038007992 0.019933461 0.052329897 0.024139414 0.111699393 -0.019431306 0.015408873 0.096980319 -0.015208069 -0.068596622 0.060830657 -0.029200418 -0.042792743 0.034300746 -0.00697706 -0.065580438 -0.00621499 0.019567951 0.015657052 0.011509308 0.035611984 0.022016145 0.132535092 -0.003155041 -0.026932842 0.019656769 -0.016473549 -0.069583468 0.026693044 -0.012951481 -0.021407504 -0.043287973 -0.035666529 -0.017406544 0.025176275 0.037458027 -0.024084745 0.027449899 -0.062248343 0.033502848 -0.000534467 -0.008782903 0.067472196 0.008472634 -0.013545754 -0.03586437 0.067020016 -0.011907008 0.002284318 -0.014829819 0.023651786 -0.027000581 -0.023898695 0.015981341

sample_125 4.283493649 0.784306175 -3.938435302 -0.769052038 1.803323356 -0.270721517 1.248544611 -0.039912884 0.258965732 -0.948658752 -0.030704761 -0.882160566 0.547515149 0.659236555 -0.750699935 -0.691586877 -0.393725172 -0.754931861 -0.528084831 -0.000286373 0.114671677 0.163313693 -0.706271424 0.111578468 0.546117979 0.001891701 0.42655864 0.257013968 -0.068923924 -0.15792907 0.08627867 0.132376602 0.338837266 -0.438378123 0.453233433 -0.554539836 0.217727442 -0.332449305 -0.016961139 0.06597109 -0.086988581 -0.229946183 0.053951946 -0.234467606 -0.280446679 -0.041714831 0.103917028 -0.073783952 -0.002528833 -0.021043312 0.182883501 0.169283846 -0.128896937 0.023412074 0.216007119 0.053998893 0.078972162 -0.153198442 -0.103458366 0.09545798 0.00833121 0.067317758 -0.005550672 0.071337794 0.127617423 -0.069790558 0.025258837 -0.019196899 0.058497982 0.266140233 -0.163097039 0.036762854 -0.176323499 0.060520993 -0.105273759 0.087994275 0.290449209 0.088586859 -0.052687408 0.081756991 -0.033205615 -0.070216961 -0.043670091 0.041717905 -0.080546844 0.097534188 -0.006354808 -0.062174298 -0.079440471 0.02105521 -0.213192922 0.101042278 -0.260313729 -0.012328119 -0.059119861 -0.142995993 -0.11415232 0.082307189 -0.018832286 -0.10706178 -0.049856925 0.064971053 -0.049298738 -0.08023563 0.174301368 -0.112687627 -0.060251154 0.056418234 -0.193997437 -0.023191175 0.029646974 -0.022861453 0.015073749 0.130984901 -0.11835548 -0.036725152 -0.150034145 0.01576316 -0.115356379 0.096782917 -0.064789357 0.021298673 -0.020003101 -0.045287514 -0.004836277 0.049317202 0.156061502 0.009373906 0.082982525 0.036619811 0.042353096 -0.025058607 0.093163571 0.013309713 0.000150024 0.014721202 -0.048306956 0.069269255 -0.027121168 -0.052520097 -0.024110304 -0.048997698 -0.015982457 0.048766647 0.112508902 -0.023122964 0.025267267 0.055587005 0.000182191 -0.037217872 -0.02565354 -0.029253709 0.039151005 0.035424407 -0.090327446 -0.012824695 0.106837576 0.095917213 -0.07740743 0.014775454 -0.041201007 0.02448761 -0.053727574 0.022543348 0.045043059 0.02293954 0.038981345 0.081864881 0.052840302 0.06002667 -0.057872037 -0.055729434 -0.000913881 -0.104324506 0.029819546 -0.015939234 -0.026873879 0.062712115 0.049248544 -0.023864872 -0.078412182 -0.038284729 -0.037909672 -0.058099695 -0.002147075 -0.024008101 0.048545388 0.005881932 -0.013560301 -0.022165397 -0.081999742 -0.00843232 -0.043068072 -0.023868518 0.055377461 -0.029474162 0.087624811 -0.059516264 -0.078289191 0.00187949 0.008971979 0.008368557 0.004800487 0.025911827 0.014079309 0.035611521 -0.004501259 0.004942094 0.01726913 -0.013527847

sample_126 -0.951496354 -1.142614978 -3.192978022 -1.521815948 -1.271948762 1.425425635 -0.864475216 1.936514385 1.194554495 0.546634084 -0.54763547 -0.389312413 -0.251009409 0.747887164 -0.565741798 0.334186076 0.286873061 0.457286806 0.726428164 0.691291724 0.172452274 0.011801808 -0.047142562 1.102087022 0.486787711 -0.238630749 0.183342364 -0.013827326 0.326896679 0.032134187 -0.656339164 -0.524579576 -0.164838252 -0.25418998 -1.159011668 0.602468773 0.033817534 -0.531613833 0.103564236 -0.249659225 -0.276251893 -0.232974319 0.205328721 -0.336656869 0.360266902 -0.166372539 -0.109444992 0.165946504 0.020413394 -0.172379946 -0.000369022 -0.109053494 -0.024093476 -0.162302016 -0.103198515 0.10341894 -0.195911173 -0.107960361 0.182572711 -0.294384536 0.254387201 -0.554200549 -0.178383245 -0.146770152 0.069133061 -0.1092785 0.18993589 -0.293256729 -0.188446441 -0.216898065 0.064378368 -0.234696955 -0.113074613 0.119172796 0.112020832 0.12800868 0.245382276 -0.294329473 0.199311813 0.22887228 0.134816254 0.016248385 -0.070823591 -0.006584126 0.061670105 -0.04443882 -0.042517968 0.031614602 -0.319085415 0.360845451 -0.32123481 0.219129321 0.103599339 -0.170988377 -0.106758791 -0.014611213 0.117902489 -0.064145187 0.027653726 0.097724689 0.002378255 0.067969975 -0.042413256 -0.132564307 0.061013337 -0.145549286 -0.25093338 0.007218369 0.016727289 0.085402227 0.068336195 0.101338181 -0.107621011 -0.050299991 0.073354005 -0.010014138 -0.008192103 0.02570356 0.047125449 0.017618617 -0.064731277 -0.098627938 0.025705443 0.037655401 -0.042488886 -0.059246322 0.00407724 -0.122273557 0.040246416 0.008898684 -0.055352386 0.21911006 0.008892857 0.183928821 0.010701086 0.034515141 0.057803322 -0.033808527 0.075831312 -0.222768757 -0.020822737 0.108444617 -0.061315674 -0.018389962 0.083113586 0.000638724 0.146958496 0.010692932 0.068862953 -0.040732494 -0.14855506 -0.003133186 -0.074077768 0.156600099 0.168485448 0.027025105 -0.058637453 0.044786436 0.124975299 0.045112812 0.215516132 0.058211808 -0.056343201 0.002872832 -0.012945649 -0.034689336 -0.031840632 -0.166484712 -0.042636475 0.017492097 -0.017261946 -0.022476421 0.058072081 -0.025203903 -0.140459271 0.049910033 -0.093692701 0.086207759 -0.016766976 0.180621723 -0.14725992 0.006919275 0.008170424 0.054370838 -0.008757262 0.119822665 -0.091801307 0.128957469 -0.065403113 -0.024696766 -0.018150166 -0.135265492 0.05983409 0.039869293 -0.076019011 -0.051286155 0.053036463 -0.017343086 -0.070930102 -0.045184012 0.02787304 -0.000100467 -0.015386452 0.101814117 0.103232228 0.057176605 -0.02365707 -0.10296826 -0.055384085 0.054292453

sample_127 3.760087265 7.01114822 -3.843576176 0.796494951 -2.213345382 -0.937751231 2.580502527 2.855511723 4.503108591 0.683811173 6.303903709 -3.677329913 0.16798546 -2.203353092 -0.394333098 -0.051580395 1.448645405 1.755874223 -1.929943904 1.339739616 3.134560842 -1.860582309 2.884571685 0.669795904 -1.82439775 -0.976722136 1.129288624 0.592827086 -0.771512231 -0.540206103 0.071027719 0.787157516 2.368410398 -2.832007634 1.246901308 -0.86679476 2.554061452 -0.315306584 2.983907667 -1.727511537 -1.577377143 -0.635144313 -0.611496554 0.871071287 1.054908939 -1.147704769 0.785999002 0.776995454 -0.838842608 2.597980961 -0.429290103 2.011350067 1.277766102 -0.84955315 2.445165893 -1.062727681 0.799814385 0.048863902 0.337056432 0.929401362 3.917883164 2.483401701 0.020174221 -0.213035806 0.841141402 0.52744039 0.946032624 0.199264901 3.17776224 0.001672831 3.134106341 0.489646122 -0.063121088 2.641633104 -0.688042015 0.246160721 -0.683183109 -2.007712671 -0.983305336 -0.520664192 -0.023530763 1.1724416 -0.517768895 2.077557023 -0.542957735 -1.1545233 0.188138262 0.829759839 -0.479241924 0.938024236 1.267265283 0.856375883 1.14731191 -0.619156922 -0.094488319 0.307946625 -0.15123053 1.132222953 -0.251073324 1.097646476 0.241527288 1.181509105 1.025658271 0.801829634 -0.412053648 -0.422790432 1.078229305 0.180692163 0.020963352 -0.005582705 0.537171797 1.634539976 1.031947773 0.896828384 -0.016335824 1.270614562 -0.497235936 0.761393832 0.350491125 -1.26632613 0.539522022 1.164653534 0.127004535 -0.196881321 0.164047344 -0.685539376 0.265747294 0.753593656 -1.09890721 0.56183923 0.448805125 -0.14298338 0.567101802 0.148663432 -0.752218743 -0.684673267 1.374148155 -1.089093216 0.608309594 0.047182278 -1.079572218 0.519338695 -1.244734295 -0.525763531 0.005107573 -0.143362645 -0.672491658 0.981882499 1.025012371 1.357207734 0.044629469 0.57817088 -0.279916044 -0.531521888 1.372163654 -1.023381715 0.809391093 0.135171268 0.082979242 0.204830339 0.475414666 0.839592263 0.184519841 -1.418435744 0.413636998 0.005963262 0.312565841 0.864604295 0.055404983 -0.447597382 -0.089425732 0.710985392 0.892464818 0.590877404 -0.175900474 -0.390930558 1.091451569 0.299844716 0.470463027 -0.293585773 -0.112651487 0.44945947 0.078349869 -0.428538867 0.132341528 0.172757356 -0.539032448 0.716181189 0.782606512 -0.68864932 -0.157115811 -0.404194855 -0.334188934 0.233824899 -0.410321523 -0.078931785 0.199861911 -0.299676098 -0.298496461 0.24223969 0.337481543 -0.181228696 0.351821433 -0.50054544 -0.328986798 -0.114001419 0.795374253 0.115303319 -0.209879888 0.173184403

sample_128 -0.056239548 -4.664692774 -2.887937659 0.87596938 -1.948308929 1.25185616 2.866335441 -0.519484033 1.103322462 -0.76319786 -0.792824279 -0.279477106 0.038961511 0.718723015 -0.002804389 0.195411013 -0.265299653 0.463552691 -0.198842395 0.522907943 -0.428062179 0.014225244 0.381248224 0.100182133 0.599855572 -0.649630729 -0.347687349 -0.509513219 -0.051238436 -0.582128653 -0.127326842 0.127102462 0.533960099 -0.103946062 0.020753721 0.401317405 0.302845495 0.034423722 0.489863596 0.012947711 0.01924605 -0.077542383 0.156881239 -0.088282521 0.221211145 -0.016682108 0.060784651 -0.244936229 -0.11064346 0.134764685 0.374694409 -0.356157365 -0.250044274 -0.127895445 -0.270308763 0.146359508 -0.397678508 0.02319524 -0.14805739 -0.198259709 0.124842004 0.164957432 -0.023939646 0.127959205 0.181184959 0.044582435 0.175645998 -0.31024058 -0.166708461 -0.055560514 -0.165563909 0.193857716 -0.341736467 0.128899372 -0.00858411 0.080241349 0.070413729 -0.063260431 0.102490878 0.052999215 0.201745887 0.025221646 -0.11718042 -0.092129681 -0.206776428 -0.02096813 0.000177825 0.082578309 0.095026624 -0.009023165 -0.085284274 0.415921814 0.089267078 0.189101809 0.111277267 -0.11822572 -0.092106602 -0.054624002 -0.150428474 -0.212308702 0.125774008 -0.009580198 -0.0422343 -0.062512956 0.026496951 -0.0689266 0.02032715 -0.06346406 -0.016935338 0.052945451 0.011178696 0.114306361 -0.08338614 -0.202894326 0.019488313 -0.00717851 0.01145148 0.108991195 -0.224698039 -0.087261206 0.138569953 -0.020495279 -0.057702282 0.094530589 -0.026510117 -0.155174626 0.01822192 0.029251958 0.133321088 0.100827887 0.011986331 0.076794826 -0.164324078 -0.053423229 -0.099019594 0.079654896 0.181426378 -0.25667163 -0.026099723 -0.05913 0.12799925 0.036609416 -0.125265487 -0.118548285 0.029170718 -0.023507703 -0.066092536 0.051720414 -0.109380962 -0.106956441 -0.03471805 0.044634554 -0.031097845 -0.07917879 -0.06314655 0.007685045 -0.046832035 0.015127425 -0.030621143 -0.05064732 0.062081236 -0.041809907 0.095962424 0.03299516 -0.032242409 0.014501235 -0.015925481 -0.081104978 0.00556007 -0.080683838 0.056847252 -0.001283564 0.160037829 0.062539176 0.037024769 -0.072101176 0.046236807 -0.025555649 -0.081260589 -0.001841527 0.114514929 -0.046532289 -0.074309307 0.023760894 0.001522177 0.016031226 -0.026925674 0.037302462 -0.048323111 0.002457189 -0.004289914 -0.063109374 -0.146539893 -0.032931187 0.061823172 0.069971232 0.026576332 0.074617057 -0.000736177 -0.020000718 0.101906429 -0.019576368 -0.056979299 0.04267066 0.021438849 0.004956751 -0.107074585 0.009175201 -0.067875107 -0.033954801

sample_129 -6.74425677 2.203114899 -2.451406364 1.085978022 2.922364745 -1.436552611 1.670268487 -0.490354598 1.125657539 0.382383211 -0.169604749 0.854341727 0.478156859 1.660432385 0.449933327 -1.271930194 -0.140086455 0.217892664 0.811522564 0.052948202 -0.353784441 0.732005847 -0.109371894 1.089189435 -0.872983381 0.471753695 -0.18961215 0.751162183 -0.098366098 -0.029592455 -0.116758151 0.037992876 0.216241554 0.147361556 0.593083842 0.008707611 -0.132605455 -0.31098628 0.229256012 -0.325030352 -0.484778284 0.840153011 0.169856223 -0.359339213 0.126509601 0.129536785 -0.135755617 0.102407755 0.080812363 0.165704335 0.107988652 -0.011846041 0.209899431 -0.204430839 0.103382045 0.094872376 0.03200726 -0.037234461 0.40486217 -0.00334359 0.149467828 -0.291821601 -0.088810589 0.192952137 -0.218593931 -0.117274624 0.096026131 -0.090161889 -0.139712121 -0.030696434 -0.093629542 -0.028299626 0.090352443 -0.075228976 -0.01503749 -0.110651994 0.064166594 -0.078044169 -0.015797624 -0.179853691 0.030263771 0.134164875 -0.161637391 -0.051140265 -0.040236974 -0.138640523 0.042372666 0.002665036 0.115260185 -0.347597182 -0.048079836 -0.074563345 0.039791262 0.123220089 0.214738358 0.065309209 0.085647545 -0.176971833 -0.029614448 -0.084304514 -0.164419104 -0.215184666 0.094434236 -0.029072187 0.172731688 -0.120183632 -0.124946127 0.108357075 -0.04842046 -0.20901061 -0.164209853 0.069089218 0.148292847 -0.142660595 0.107726268 0.102725756 0.029948785 0.015332896 -0.002208639 -0.189451447 -0.030912858 -0.04292512 -0.052307109 -0.011880749 0.137983082 -0.019779984 0.015182326 0.080913672 -0.008200784 0.03990151 -0.006677391 0.097024014 0.12655198 0.032088977 0.015854548 -0.013827934 0.006966408 -0.021339903 -0.038044989 0.004478365 -0.026126075 -0.032566381 0.007681447 -0.002418653 0.02573394 -0.038558154 0.004333929 0.069868747 -0.008353632 -0.04174082 0.057691703 -0.078582667 0.022944605 0.060989574 -0.005942662 -0.024977096 -0.067152141 0.148735886 0.021700271 -0.047898731 0.035266188 0.005040437 -0.052976383 -0.030052785 0.000896291 0.062812738 0.003756274 -0.117996801 0.040842154 0.042436395 0.017374423 0.042168805 0.064842236 -0.069874909 -0.016098396 0.045638246 -0.069582215 0.071970864 -0.01290941 0.061922696 -0.03877267 -0.050572627 -0.005802331 -0.105463735 -0.086867893 -0.123273096 0.006944915 -0.027556354 -0.051326518 0.012458539 -0.028411873 -0.0564602 0.075545319 -0.052213135 -0.025479729 -0.014317289 -0.038267058 -0.066470575 0.017200288 -0.007846386 0.013907388 0.07761599 0.026225412 0.033715925 0.009522465 -0.07205949 0.040677741 -0.008955734 -0.007963234 -0.017402123

sample_130 -4.399121184 -1.791171012 -0.966477137 -0.966753548 1.813517097 -0.919194223 1.057762039 -0.785816447 0.665930225 -0.295013815 0.66754202 -0.696829402 0.618094676 0.676911852 1.373046582 -1.594492398 0.175500641 0.545411431 0.613277809 0.371328924 -0.837957961 -0.030734682 -0.117791697 0.754240171 -0.048021992 -0.618625184 -0.598465297 0.167917682 -0.352888782 0.365648782 -0.655507783 0.361084615 0.651321026 0.139310457 0.24371579 -0.149571999 -0.172321909 -0.011226726 0.683112723 -0.121087295 -0.496072561 0.119991633 -0.641735915 -0.739061855 -0.405662399 0.048003554 0.122896498 0.098854529 -0.132672341 -0.303341191 0.240782759 -0.135256711 0.002534485 0.107818226 -0.348500351 -0.325271566 0.072966618 0.000924757 0.217605852 -0.166891632 0.08319995 -0.124022251 -0.29130071 0.427698971 -0.19848607 -0.011844668 0.000558107 0.064806731 -0.183634342 0.08703269 0.082146229 -0.230830672 -0.134541974 0.058669387 0.207685744 0.215582488 0.14640058 -0.231949373 0.271923781 -0.070838712 0.07799924 -0.036965238 -0.053389215 -0.018215189 0.148911376 -0.157539492 0.042423298 0.130344032 0.081811716 -0.09406717 0.008623378 0.004478507 -0.024909203 -0.042266049 0.045460435 -0.10105479 0.035687445 -0.234992203 0.044566498 0.017950296 -0.0315795 -0.249411208 0.110837509 -0.05924238 -0.033399653 -0.114673681 -0.373949464 -0.10306995 -0.262335866 -0.105844173 0.211239821 -0.198322569 0.04011599 0.052720904 0.003940231 -0.039784503 0.087112822 -0.051045399 0.105084101 -0.15978462 -0.019001363 0.166237576 -0.032876679 -0.059795415 -0.054786722 0.094153535 0.096792672 0.071168962 0.007302611 0.133885376 -0.082582989 -0.099638113 0.15215526 -0.130489735 0.043968135 -0.080752826 -0.031575696 0.059301009 -0.023823006 0.165272364 -0.024925989 -0.100167457 0.014254356 -0.037849781 -0.00372717 0.056758794 -0.005760119 -0.038525902 -0.037514547 0.002286566 -0.105929284 -0.017757497 -0.015072119 0.066154913 0.000462468 -0.038002038 -0.002516114 0.056615269 -0.053623095 -0.012206328 0.007408933 -0.066911103 -0.059728883 -0.010504553 -0.050449184 -0.003905596 0.072548845 -0.009685452 0.010738195 0.08535845 -0.055341433 0.019866966 0.031466916 -0.010023893 -0.10643344 0.089689072 0.029822381 0.097376542 -0.017642974 -0.010184791 -0.071207129 0.02589158 0.011706909 -0.113892227 -0.030821803 -0.025971818 -0.097910804 0.038861014 -0.047726732 -0.015609404 0.031260756 -0.022477844 -0.050893197 -0.01544884 -0.003908924 0.043745932 -0.019118973 -0.089372124 0.069681612 0.057280707 0.138756021 0.040357877 -0.071427838 -0.006426424 0.066023751 0.078829635 0.015458498 -0.002314873 0.010316326 -0.028614761

sample_131 -0.311727487 -2.571639353 -1.598819646 -3.552050514 -0.277075286 1.89569252 -0.807337542 -0.261925442 1.34675328 1.32915621 1.172557793 0.404826295 0.701630992 0.077891682 -1.105158112 1.092000443 -0.827292327 -0.032192084 0.732529042 0.014506129 0.389006964 -0.048315232 -0.209294187 0.596844966 0.361996768 0.157294407 0.324403765 0.093998532 -0.058393039 0.219322673 0.563819989 0.359971987 0.363658017 -0.167840245 0.584401188 -0.149526515 0.284353994 0.375345813 -0.160678426 -0.102680074 -0.207151213 0.03683159 -0.046583131 0.02198805 -0.482589583 -0.078083722 0.087631252 -0.056867341 -0.223811729 0.261072552 -0.172488047 -0.094207275 -0.013955389 -0.141069255 -0.037317251 -0.202235746 -0.174281403 0.136327881 -0.19853049 0.047007992 0.08878175 0.169176766 -0.114996982 0.341593169 0.122094368 0.07693414 0.133403378 -0.155028518 -0.026833989 -0.051700391 -0.0326831 0.135026899 -0.070842646 -0.020675151 0.017210506 0.025766523 -0.114746017 -0.093743001 -0.237518904 0.315295775 0.032236736 -0.258726906 -0.195697373 0.237735492 -0.152477751 -0.074065894 0.243066324 0.079055516 -0.017402606 -0.144847201 0.032217329 -0.057522738 0.293657427 -0.164522 0.014754796 -0.203280173 -0.010240174 -0.012308298 -0.145198525 0.221892632 -0.116799281 -0.098281374 0.07845476 0.097582409 -0.008510062 -0.031135042 0.069406114 -0.274588006 0.070064016 -0.000913281 0.183702351 -0.025070572 -0.113119743 -0.0128932 0.104173634 0.074819516 0.017470428 0.015358713 0.035433025 0.079086971 0.016319823 0.053424985 0.024500627 0.072948366 0.029896605 -0.040069746 -0.162494667 0.057280904 -0.109138545 0.086569422 -0.098077678 -0.008319532 -0.117759644 0.112723365 -0.162346064 -0.034507588 -0.027830866 0.067847118 -0.046272151 0.050670507 -0.045719699 -0.116749489 0.012392533 -0.049476753 -0.019736998 0.028460848 0.056998653 -0.008596379 0.063408296 0.140160462 0.117378132 0.068025095 0.056365063 0.059651963 -0.176794403 -0.02789334 0.035908954 0.041704747 -0.106590416 0.019876528 0.095317231 -0.104062234 -0.102795112 0.08054054 0.06599509 -0.178651878 0.169286533 -0.080489374 0.101386287 0.014131241 -0.064428876 -0.05009032 -0.120358393 0.062257654 -0.205051413 -0.083915192 0.05614204 0.063419076 0.037677493 0.089360461 -0.004016277 0.050880644 0.025812073 -0.06099517 0.043021589 -0.012681483 -0.085589779 0.029387342 -0.000751711 0.111650014 -0.021624087 -0.045443633 0.035599474 0.056918163 0.059673844 -0.061190668 0.073056898 0.003148911 -0.066954428 -0.03283532 0.015536765 0.041861855 -0.008372427 -0.013032681 0.053541116 0.035558043 -0.028388365 0.001876209 0.025383189 0.004993485

sample_132 1.143396011 -4.318915184 -1.791472183 -0.13852268 -1.71979806 1.292227293 0.571065772 -0.164937822 -0.999438863 1.965080084 -0.131365072 0.235019768 -0.881799268 -1.146665566 0.832292198 0.268192233 0.257215159 0.291473215 0.882481672 0.687018951 -0.111236515 0.060851499 0.506051 0.36316372 -0.797384325 -0.116740773 -0.046489937 0.615216708 -0.120681202 -0.042385451 -0.276655347 0.279297697 0.039434463 -0.223307346 -0.381089115 0.478452782 0.326420596 0.013569287 -0.194174356 -0.231782387 0.150675092 -0.117009534 0.809034381 0.322435702 0.284978 0.181219423 0.021141298 0.526485894 0.139998642 -0.210077501 -0.060575511 0.294641156 0.040361628 0.170233223 -0.017489243 -0.138850708 0.181319146 0.004700274 0.255230217 0.119608951 -0.134571667 0.084979209 -0.003942101 0.069190967 -0.200356456 0.186891609 0.154614842 -0.198627973 0.181368022 0.148133908 0.044505168 0.141339031 0.16284156 -0.067627561 0.101348144 0.11478675 0.282962149 -0.047342737 -0.018902592 -0.029005204 -0.159304415 -0.156188894 -0.095225496 0.250700668 0.032196332 -0.027646858 -0.02504656 0.172533806 -0.13990302 0.121158838 0.189079505 -0.121800988 -0.082575822 0.040514264 0.006593597 -0.047467172 -0.260753755 -0.113109188 0.046865445 0.235429006 0.079660513 0.144246645 -0.049040003 -0.036631152 0.132039144 -0.01227052 -0.002577092 -0.033225565 0.06255522 -0.064817684 -0.092179823 -0.074612077 0.013498149 -0.058561015 -0.091147442 0.092382413 0.11567697 -0.126288854 0.025851156 0.151435697 -0.067943429 -0.082984595 0.048796641 0.081846766 -0.017306968 0.052175172 -0.003625252 -0.050711215 0.011832888 -0.07683303 -0.032205581 0.044293726 -0.068760393 -0.195181351 -0.098089667 0.134586677 0.049905908 -0.002131622 -0.131388174 0.091647829 -0.065662908 0.038801788 0.133191696 -0.04578715 -0.005485948 -0.030628453 0.032112866 0.034220422 -0.04007741 0.050479464 0.008520939 -0.11648197 -0.181157221 0.092559653 -0.080075036 -0.07664244 0.098869915 -0.024527919 0.081934023 0.206362373 -0.047898051 -0.062586973 0.014348517 0.040110992 -0.010166289 0.026152735 0.004290237 0.016102594 0.10378777 -0.01752022 0.042427987 0.047841137 -0.02422009 0.000670975 -0.03423481 0.003875409 -0.073364449 0.084402669 -0.004036313 0.00659911 0.002896325 -0.036733225 -0.178962238 0.064273546 -0.057539858 0.042438537 -0.048476453 -0.046217946 0.006017438 0.039898426 0.050808561 -0.067215806 -0.033770961 0.044487987 -0.016433289 0.005018737 0.085489924 -0.017467535 -0.021099397 0.020817656 -0.059318218 -0.038299146 -0.010741123 -0.031588147 -0.013158087 -0.046349779 0.019290455 0.088142818 0.002311103 -0.021056061

sample_133 -4.084390188 -2.230893919 -1.075317835 -0.446102267 -0.238814893 0.791200294 -0.088200498 0.566555523 -0.678591344 -0.508045428 0.10011922 0.050617788 -0.835357605 0.433708066 0.369976288 0.118430277 0.700145009 -0.060232318 -0.597553634 0.257158699 -0.120519909 0.39764335 -0.292497325 -0.336045025 -0.130536643 -0.225730469 0.277215181 0.064083289 0.037324631 0.048434303 -0.08764365 -0.097605003 -0.224185363 0.183221229 -0.071668704 -0.400465927 0.162216155 -0.273398145 -0.06490279 -0.022097095 0.068950086 0.199755779 0.157810322 0.033178872 0.056253526 -0.223846407 0.101218765 0.054363643 -0.134504785 0.022844903 -0.181168112 0.004094321 -0.082597205 0.147681702 0.068298807 -0.064189148 0.090305462 -0.013297644 0.007539851 -0.16519256 -0.18528475 0.003035542 0.058341528 0.188421806 -0.168116458 -0.057065634 0.087754763 0.013345709 0.025094611 0.05718948 0.036876869 0.125197723 0.007378645 0.072123355 -0.025190159 0.049699253 -0.009744384 -0.05266191 -0.059037183 0.023023119 -0.1289377 0.03173894 0.054506762 -0.01702014 0.070635238 0.019662884 0.105045163 0.010299243 0.088534738 -0.046882729 0.024042178 0.008367881 -0.013452402 0.038423064 -0.00284342 0.040707427 -0.081637796 0.034853974 -0.057712668 0.038002604 0.027649311 0.023904093 0.012849426 0.015853213 0.098170264 0.040876108 0.025332072 -0.054542053 0.035385063 0.139890786 0.031376781 -0.167459544 0.007085894 0.037302878 0.038258341 0.035679823 0.077098464 -0.014291918 -0.041391803 -0.036082085 0.022297594 0.005795607 0.063149005 0.028411386 0.047538604 0.040344117 -0.029003497 -0.014966264 0.070747671 -0.060991714 0.023424578 0.027796872 0.017053098 0.044395025 0.001488155 -0.084052454 0.015860788 0.012606878 -0.005837142 -0.07923402 -0.011703932 0.014774189 -0.016957024 -0.012218573 -0.005377663 0.032440943 0.030670719 -0.095344846 0.036961868 0.027383505 -0.005826843 -0.030192611 0.025017927 -0.008026454 0.014261808 -0.083557854 0.015597478 0.018031393 0.005629069 0.040236292 0.019463377 0.02467896 -0.021899145 -0.067848761 -0.010386174 -0.042571079 -0.02317797 0.043372091 0.015970705 -0.011590579 -0.024291387 0.012542556 -0.038530592 0.012366528 -0.034807992 -0.023587863 0.005461918 0.02008772 -0.016809321 0.012919806 0.016130443 -0.025556797 -0.015391685 -0.005350733 -0.029828963 -0.005574859 -0.044750524 -0.005967113 -0.019990993 -0.048018179 0.001390829 -0.005154651 0.003233234 -0.011753109 0.011390305 -0.014990476 -0.001722333 -0.02128365 -0.004782346 0.005766442 -0.005474748 0.023755845 0.026227789 -0.051933222 0.017388575 -0.016572505 -0.02664396 -0.010322857 0.003548911 -0.008092979

sample_134 -5.815676377 0.080733605 -1.358028616 3.013283596 1.886029752 -0.372177997 0.341478875 -0.562759167 0.523057922 -1.034445458 0.17109224 0.370658588 -1.095679119 0.94827175 -0.087633827 -0.536317462 0.533610964 0.35470234 0.071789762 -0.539574039 -0.620105715 0.665695893 -0.315947694 -0.384037668 -0.36410786 -0.707069245 0.382677445 0.179480332 -0.089455405 0.514945194 -0.483707058 0.114234609 0.296926658 0.203467948 -0.165415566 -0.305223394 0.299853527 -0.399049379 0.283243827 -0.092227765 -0.045169218 0.261466058 -0.136634531 -0.122405816 -0.033358404 -0.271274092 -0.104373514 0.217665377 -0.345493372 0.123238692 -0.033267043 -0.09394178 0.088700831 0.222042134 0.122501701 -0.032885628 -0.264849914 -0.110251032 0.20474397 -0.241577966 -0.04803258 0.192790429 0.164865459 0.174874058 -0.137519311 0.308679509 -0.0029036 0.062471808 -0.045405049 0.040920737 -0.004645902 0.117864569 -0.118987918 0.032863881 -0.209234123 0.198499106 0.139203457 -0.229098464 0.042127351 -0.041092736 -0.185132467 -0.135423047 -0.137967825 0.050973363 0.183378127 0.006698275 0.064207693 0.016357577 0.214748004 -0.039118321 -0.000575571 0.104340353 0.117270621 -8.64E-05 0.062217556 -0.224065388 0.082916246 0.059654134 -0.148951607 0.106565301 -0.026454305 0.076795201 0.075023783 0.073885544 -0.132851668 0.214433606 -0.007348259 -0.007555626 0.17523386 0.108554852 0.04612831 -0.063720307 -0.002242505 -0.049878284 -0.087445022 0.073553778 0.036004652 -0.063380741 -0.059923637 0.019028921 -0.034534983 0.068319001 -0.071841423 0.042730646 -0.070152969 0.122321964 -0.168028496 -0.048690129 0.063294744 0.028523988 -0.016274495 0.165909557 -0.088830762 0.007422021 0.027747127 -0.109134598 0.024725109 0.011503503 0.04773491 0.08274294 0.070087579 -0.153632233 -0.056876862 -0.046748788 -0.024811237 0.007882418 0.027233104 -0.137943562 0.070144189 0.024147357 0.062756071 0.106513728 -0.065000837 0.013131439 -0.041235511 -0.091111688 -0.017037524 0.005590899 0.04993395 -0.056670813 -0.07374797 -0.010784614 -0.071211841 0.020079161 -0.007959147 -0.001213679 -0.006568239 -0.013091421 -0.034152747 -0.043285447 -0.019464698 -0.105080816 0.102191403 0.027429235 0.060503752 0.027100502 -0.077341567 -0.04251751 0.009855622 0.043320362 -0.007012694 0.023539714 0.043326403 0.031545519 -0.06676951 -0.094259576 -0.032818803 -0.001705122 -0.090964982 0.011674761 0.057184442 0.081629782 -0.065426972 0.002642657 0.022968951 -0.03175449 -0.014213228 -0.013906962 -0.027526469 0.085037391 -0.031996192 -0.04600053 -0.034929981 -0.020413129 0.064634713 -0.049384335 0.006611012 -0.046384828 -0.118717611 0.020071056

sample_135 5.555208858 -1.788935082 -1.22524306 1.064908316 -1.766980572 -0.236056323 -0.659363639 0.353728221 -1.259262773 -0.250063179 0.50560524 0.116809395 -0.184841146 0.850133285 -0.028602142 0.410137586 -1.165442226 0.052470607 0.007724989 0.235000402 -0.448265767 0.311514117 -0.361094952 0.03038517 -0.265559915 0.220352567 0.370020586 0.080799801 -0.07105149 0.325938345 -0.099008247 0.223575972 0.279064328 0.353724404 0.087485366 0.717898975 0.034369015 -0.19679792 0.158639356 -0.221057829 -0.684466266 0.173538025 -0.115824112 0.284866767 0.37092797 -0.151519636 0.287945479 0.223616329 0.056296479 -0.335111967 -0.158145232 0.006855345 -0.217933259 -0.090812006 0.080965933 0.025279565 0.211199373 0.060214727 0.057950235 -0.096060864 0.353358048 0.068604575 0.024357348 0.276553774 0.180468111 0.01877473 -0.038576025 0.103282782 0.104945348 -0.03672084 -0.032646487 -0.005924078 -0.152678096 0.052997472 -0.191155996 -0.204164487 -0.016074831 -0.105914075 -0.110710111 0.230993071 -0.026719514 -0.044619674 0.092921861 -0.004628508 -0.036092312 0.122350541 -0.226346211 -0.084411582 -0.141333243 0.122901816 0.087132311 -0.229125912 -0.054971352 -0.029948627 0.012333434 0.113723569 0.000513606 0.061877829 -0.110663024 -0.042095953 -0.091237738 0.075154647 0.131982485 -0.077419446 -0.089760849 -0.073387714 0.077377719 -0.124876813 0.033329403 0.058992744 -0.100592274 -0.034842449 -0.001927148 0.076666061 -0.041434457 -0.051028479 0.016167123 0.087515468 -0.128796565 -0.040377766 -0.058927257 -0.071446723 -0.031668895 -0.102953199 -0.198494466 0.047958918 0.048940755 0.038029328 0.028924479 -0.036627119 0.027223276 -0.005287314 0.100892805 0.083793588 0.04822934 0.038160228 -0.121367864 -0.005176761 -0.012873703 -0.033671972 0.01989084 0.074060945 0.123362553 0.033252801 -0.018724556 0.008176367 0.007987707 -0.06018965 0.018833895 0.070470405 -0.012471549 0.029962991 0.049560001 -0.016797721 0.00318925 0.125032646 -0.004200454 -0.072450751 0.013232303 0.016783459 -0.012199288 -0.010094301 -0.061505742 -0.069434233 0.018024513 0.050775358 0.049002345 0.016778746 -0.071011659 -0.011204666 -0.078398245 -0.00021341 0.02316286 -0.044726083 0.060549072 -0.000185049 0.014113708 -0.062770114 -0.082423584 0.026063614 0.068579274 -0.023979053 0.03145374 -0.029952939 -0.028065835 0.027260355 -0.002028892 0.067808694 0.000173523 0.008748603 0.001871702 -0.026531558 0.014918383 0.04269724 -0.043547895 0.024923271 0.023119046 -0.038754035 0.027594723 -0.012976498 -0.043925791 -0.086402697 0.01194554 0.042901204 -0.046462415 0.001359255 0.027666178 -0.068748206 -0.010461202 0.020488085

sample_136 5.762788793 -0.414008856 -5.04317064 0.489682699 0.751253465 0.960011516 0.370497803 -0.324036086 -2.20159383 0.356216779 1.953914342 -0.34806734 0.705632316 1.056135293 -1.458144887 -0.179342535 -1.51611472 0.286152753 0.540453692 0.375479637 0.620297967 0.01562636 -0.541087881 -0.204020849 0.416310805 -0.19050647 0.598831516 -0.728904087 -0.150858322 0.403530959 -0.162255006 -0.597007961 0.657268365 -0.817615205 0.783572629 -0.127272552 -0.720521719 0.362191205 -0.224357585 -0.354726281 -0.33575018 -0.27205481 -0.250756417 -0.204616704 0.098964776 0.1254826 0.323376557 -0.501194868 0.088917848 -0.15066488 0.097523496 -0.337064632 -0.347815799 0.112720614 0.43477643 -0.198868244 0.150166983 -0.225874944 0.096887075 -0.075577159 0.304032876 -0.106148297 0.185759458 0.3798881 0.195230644 -0.09294472 -0.213814936 0.140661368 0.180094302 0.602842205 -0.167839202 0.112409073 -0.383885915 0.090760944 0.071864928 -0.110492613 0.166625452 -0.031037683 -0.090174296 -0.039545866 -0.046433709 -0.152908523 -0.156117212 0.148470394 -0.105420702 0.157192629 -0.059096432 -0.01928969 0.055503158 -0.033394354 -0.036090779 -0.169056335 0.002188558 0.039754419 0.038444239 -0.243217147 -0.078354804 0.073270063 -0.030267616 -0.117893665 0.094621292 0.024070373 0.09191978 0.043151916 0.038324634 0.162823588 -0.134704282 -0.106646809 0.056186901 -0.060599358 0.047303614 -0.040714924 0.076512423 0.042431434 0.136878824 -0.003880558 -0.137444422 -0.134501755 -0.049482762 0.071409643 0.087314153 -0.231647072 -0.118648364 0.069428175 0.014342827 0.108257077 -0.00622244 0.055106791 0.102465743 -0.169840296 -0.015514806 0.075713608 0.036499935 0.000345006 0.113600994 -0.039523971 -0.021762724 0.062509223 -0.004229249 -0.068960183 0.066076495 -0.100190372 -0.068835723 0.051250999 0.094452853 -0.064233203 0.110951967 0.069717483 0.000954757 0.002128643 -0.083892725 0.023517724 0.058560752 -0.003813718 0.037688584 0.061340135 0.107519865 -0.056638446 0.074476544 -0.088182376 -0.130957813 0.027972093 -0.202604044 0.000269585 -0.008593252 -0.079885566 -0.033221696 -0.013922107 0.072931429 0.035679989 -0.051000155 -0.097767157 0.020736345 -0.030433242 0.033196263 0.011336864 0.010053071 -0.032267017 0.015286232 0.111105814 0.025314352 0.07786148 0.042806428 -0.068451783 0.034020007 -0.01896807 -0.067448573 0.06821596 0.011420182 -0.086655236 -0.0409422 0.053477447 0.044765421 -0.073506987 -0.029989292 -0.03653024 0.038304103 0.006027431 -0.012506418 -0.037318209 -0.011839275 -0.097108735 -0.014860924 -0.042382425 0.004204207 0.033112261 0.038115809 -0.04057984 0.024524876 -0.0403071

sample_137 0.867205966 3.550654921 -5.511602667 0.135472078 0.184601254 1.078833536 0.587574018 0.833281275 1.447652317 0.807009128 0.419325434 -1.223408932 -1.195434193 0.185465192 0.14660222 -0.630328173 0.767382778 1.589219982 -0.437119632 -0.293420043 0.785113486 -0.30403424 1.123167844 1.54632857 0.02393262 -0.087005008 0.231373302 1.073794237 0.926492743 -0.552248556 -0.364547225 -0.457609604 0.613779918 -0.715833942 0.176351716 0.75322492 0.732588787 -0.567613933 0.804578046 -0.75639822 -0.118544314 -0.264113998 -0.222496135 0.191675001 0.660375642 0.005002683 0.335415409 0.324165781 -0.023760317 -0.112102171 0.286730309 0.489317621 0.168371308 -0.665680918 0.115841453 -0.242254732 0.413430502 -0.015725617 0.207823913 0.16227158 0.44732239 0.256163595 0.653205585 -0.568575375 0.598710724 0.040977586 0.415504071 0.169575759 0.530906368 0.230864942 0.625605676 -0.165694965 0.127216224 0.636244513 0.20600347 -0.121286082 0.038444831 -0.299320819 -0.109179563 -0.237585918 -0.335292832 0.358273287 0.16540027 0.585372781 -0.098720461 -0.163601363 0.067560079 0.205084355 -0.024488865 -0.079533263 0.23509989 0.248797556 0.325869031 -0.049939961 -0.080299139 0.223721182 0.354995418 0.113342085 0.039629987 0.281428262 0.174269485 0.032339428 -0.058248213 -0.099579876 0.206728423 -0.046988382 0.228886334 0.197795264 -0.214567892 0.175615406 -0.093445062 0.67533475 0.280797211 0.127902598 0.146851806 0.169422709 0.04820772 0.261709601 0.183543302 -0.312348723 0.052047743 0.433304686 -0.145949412 -0.009119779 0.265536364 0.034312196 0.069044235 0.186584708 0.125398914 0.304933426 0.146966271 0.123396757 0.223946092 0.071679012 0.000599484 -0.085097262 0.054071957 -0.350460928 0.15231697 -0.091042248 0.152680625 0.335444489 -0.133223867 -0.107854058 0.019446621 0.27361433 0.054908077 -0.105591335 0.30270362 0.253377561 0.073054032 0.149188625 -0.099376751 -0.009767461 0.285945147 -0.053638987 -0.018115039 0.092664105 0.005175566 -0.047879639 -0.049417861 -0.081816226 -0.001965392 -0.134511402 0.11262075 -0.186984384 0.09528697 -0.183029366 -0.05063587 -0.008773331 0.079929674 0.032782196 0.20392052 -0.001292876 -0.160079442 -0.155623783 0.013329943 0.074845999 0.041725732 -0.053478546 -0.08236607 0.040752357 -0.016900777 -0.109112385 0.262357181 0.044059601 -0.006946467 0.038453556 -0.006514416 -0.105877146 -0.156111026 -0.089820817 0.177558299 0.01555954 0.005950411 0.07609192 -0.142280558 0.046211285 -0.009808456 0.071330501 0.010265006 0.068131537 0.07674188 0.135459683 -0.108846724 0.030018651 -0.003201008 -0.0335309 -0.015639096 0.184066913

sample_138 3.015570454 9.445551172 6.399045743 -1.43123054 -5.312746014 5.144085404 1.54907562 -1.398570388 -0.310879909 1.317792097 -1.803672809 0.365431186 1.165568718 -0.153514214 -0.59734444 -0.447326433 1.439997862 1.072524068 0.902874479 0.369482517 0.734681492 0.661290551 -1.286853513 0.126460688 -0.739850399 -0.439244819 -0.521991326 0.048457286 -0.856241027 0.27731605 -0.355339278 -0.675848386 0.177595212 0.106958938 0.048728663 -0.043658589 0.020695711 -0.38465493 0.370933519 -0.104595976 0.103264832 0.086221612 0.05631525 -0.078095982 -0.031655851 -0.310549968 -0.26872286 0.192385885 0.077945658 0.078439237 0.320337205 0.235608133 -0.039681826 0.466177247 0.567158919 -0.42711426 -0.351453011 -0.061425752 -0.180010961 0.044028957 0.377245104 0.074147454 0.404137572 -0.023970501 0.142626429 0.278807251 -0.035337317 0.060812378 -0.320472738 0.044947995 -0.096499281 0.040153388 0.008108307 -0.114432966 -0.069018641 0.087787821 0.311520925 0.171267591 -0.031063497 0.099936744 0.213836366 0.120179501 -0.150939111 -0.062064267 0.006868305 0.041732257 -0.011927192 -0.186148581 -0.110869703 -0.097111887 -0.032931569 0.02516177 -0.273655789 -0.114868064 -0.236083655 -0.16709978 0.01113715 -0.030150195 -0.221864256 -0.061364722 0.001344885 -0.07434398 0.090553122 -0.015289915 0.001885416 -0.023207416 -0.000935327 0.003776586 -0.242897807 0.13840725 0.007631059 0.107554356 0.004396225 0.01415972 0.009755852 0.182749099 -0.051017462 -0.028415684 -0.203049524 -0.054255507 0.012964011 0.045275644 0.03350213 -0.141192062 -0.050588495 -0.082683919 -0.102777104 -0.121972473 0.060497228 -0.066420054 -0.021503013 0.071430082 0.014730771 0.03794258 0.05488653 -0.052456735 -0.125526681 0.05399688 0.120542205 -0.098757865 -0.121649426 0.025795314 0.00762133 0.008133765 -0.079015871 0.051002974 -0.007764157 0.045821305 0.020252748 0.068495908 -0.107174146 0.001782548 -0.023120483 0.063151326 -0.051904005 0.036026469 -0.009228386 0.001865013 -0.028560203 0.030700194 0.016212291 0.059816389 0.020886847 0.046641915 0.034093914 -0.020177599 -0.050575056 0.053815516 -0.054911769 0.083945552 -1.30E-05 0.063214307 0.070228402 -0.000385436 0.001547613 -0.042761831 -0.047628207 -0.100786906 -0.022869388 -0.00774172 -0.018695805 0.021620337 0.072350848 -0.015484174 -0.055616521 -0.015160918 0.003458403 0.097450401 -0.006681008 -0.034103756 0.039814496 0.083366994 -0.05602363 0.026719275 -0.047703736 0.080935655 -0.028988987 -0.028013701 0.025887264 -0.033462801 -0.054112109 -0.014068405 0.025125603 0.000932735 0.008534871 0.032022246 -0.02065335 0.011633573 0.000455159 0.032802379

sample_139 0.764536485 0.857720904 -5.84282744 0.579285631 -0.829801042 2.567917583 -0.242219609 0.348367787 0.011009099 -0.576037829 -1.973100305 -1.505758835 -1.269848475 -0.912345694 0.030221885 -1.167944254 0.091399718 1.502703886 0.431499318 -0.171881816 0.453389189 0.191206178 0.461284987 0.944758314 -0.440684041 0.498273457 -0.047251136 0.669218458 0.406456553 -0.633769199 -0.280920641 -0.198125522 0.287611557 -0.404371817 -0.779941999 1.020440205 0.566366635 -0.222769752 0.340815147 -0.052667054 -0.351978944 -0.343490343 0.027724561 -0.620471101 0.456472419 -0.174475377 0.900373803 -0.256671169 -0.056538167 -0.267774053 -0.319494554 0.194536642 -0.137747855 0.066806944 -0.517271582 0.113956919 -0.250581538 0.10455972 -0.268260105 -0.013337784 -0.360412084 -0.05960104 -0.012653923 -0.310629278 0.426716778 0.031710809 0.158832567 0.030625225 -0.027285302 -0.052268949 0.056394897 -0.096630204 0.240859402 0.308360802 -0.227017789 -0.048098104 0.067546089 0.248693583 -0.027432432 -0.173340034 0.006249529 -0.145185538 0.317532394 -0.175239813 0.066992706 -0.314966107 -0.108017151 0.010634464 -0.032277753 0.2203753 -0.144510708 0.193717209 -0.125622629 -0.204557383 0.140097024 -0.279599528 0.016561671 0.13410505 -0.085675359 0.273186947 0.196510482 -0.015260855 -0.1266361 -0.050804995 0.16566992 -0.184257975 -0.197896399 -0.220077325 -0.185671227 -0.102842267 -0.275504588 0.013312305 -0.229938964 0.09261014 0.015301631 -0.178032301 0.036409276 0.015545384 -0.005295847 -0.123794557 0.029070638 -0.197088758 -0.108828787 -0.008457595 -0.068088852 0.133675016 -0.015262051 0.125320347 -0.07252807 -0.032497149 0.028784128 0.078015377 0.048051782 -0.040998013 0.055783479 0.067431243 0.033764548 -0.038549171 -0.085731771 0.033151848 0.09590836 -0.001963801 -0.053360837 0.165668 0.060178132 -0.054660994 -0.030547529 0.016376773 -0.038125249 0.075232395 0.028319494 0.195523253 -0.045444588 -0.042204476 -0.038060425 0.050396805 -0.199282033 0.003952402 0.024876357 0.012175178 0.013226768 -0.149264416 0.035387512 0.021354537 0.010936922 0.063364314 -0.18142187 -0.009384363 -0.052310898 -0.017390393 -0.074110471 -0.09784123 -0.001502848 -0.016551859 -0.148372955 -0.033618631 -0.028866225 -0.041974455 0.085210946 -0.028081032 0.016046135 -0.044258984 -0.046762021 0.086212151 0.038063389 -0.084960792 -0.150203057 -0.017500189 -0.123860376 0.041820997 -0.016392622 -0.036870274 -0.099700331 -0.003099738 0.101886619 0.033773725 0.154723057 -0.073625715 0.011518034 0.014223461 -0.020275987 0.088904682 0.033649915 0.057559352 -0.017812363 0.033471664 0.0229487 0.031050808 -0.048809261 0.006394047

sample_140 -5.432003535 0.859159535 0.594587695 -0.314957175 0.452618225 0.16283632 -0.38905444 0.074843968 -0.012920544 -0.512878608 -0.413402737 0.262173179 0.425442448 -0.060406387 -0.580992148 0.238628939 -0.182941364 0.053609109 0.253828876 -0.368769701 -0.034545315 -0.302463581 -0.634577715 -0.300710427 0.538223399 -0.516023633 -0.580274742 -0.377706286 0.084510442 -0.379720851 -0.099645475 -0.141428066 -0.159651363 -0.097961612 0.341738098 0.050946106 0.226622107 0.184353392 -0.156653612 -0.064522307 -0.247023714 0.507267818 0.045420903 0.043230024 0.171350277 0.034500676 0.204052106 0.069138269 -0.155194275 -0.165597285 -0.002960712 -0.109755475 0.114601648 0.054220358 0.071786437 -0.098555111 0.005041961 0.270473021 0.152256838 -0.049469847 0.148037343 0.068372275 -0.082591873 -0.02341037 0.094528141 -0.081026579 0.073388835 0.144326097 -0.129703735 0.013486012 0.294921946 -0.088494958 0.084035216 0.048399582 -0.031446607 0.075437842 -0.040857744 -0.119075711 0.065981874 -0.109172605 -0.032766204 -0.162446661 0.016604115 0.103138581 -0.076257937 0.044630434 -0.023892021 -0.076349299 -0.005567852 0.061850621 -0.026896803 0.017180702 0.037126884 -0.008517161 0.089040438 -0.005280889 -0.10909907 -0.034931909 0.086674998 -0.023689681 0.002986654 -0.011407977 0.008403446 -0.005748715 -0.029589398 0.050508152 0.057288341 0.068886437 0.076077326 -0.191206711 0.056017597 -0.009183457 -0.111332975 -0.033647975 -0.018577838 -0.049334499 0.052390333 0.011968525 0.068706728 -0.031078985 0.022793546 -0.028439611 -0.007950463 -0.040955354 0.027107581 -0.030013394 0.098178437 -0.070827002 -0.017112206 0.057005249 0.024389967 0.116199347 0.006385407 0.01387947 -0.023082344 -0.076250966 0.01970565 0.002317021 -0.042994532 0.085703822 -8.27E-05 0.023894814 0.026342909 -0.087845443 -0.026033206 0.010597948 0.011657988 -0.004664959 -0.009457827 0.044777016 -0.079949905 -0.028557253 -0.001732782 0.102747966 -0.005208803 -0.028558892 -0.09933082 0.026451336 0.044529531 -0.015190944 0.049422182 0.0032048 -0.03411618 -0.011017012 -0.045562794 -0.076703278 -0.001837588 0.017806488 -0.016932495 -0.025456968 0.051101654 0.0038637 -0.045195554 0.035312057 -0.048695217 -0.048063491 -0.015858149 0.046709561 0.031077891 -0.006151889 0.029984552 0.023386914 0.056180892 0.01454532 0.027831484 0.045983633 0.017995855 -0.003417443 -0.015487396 0.060741888 0.035076548 -0.045866764 -0.075805035 0.014836722 -0.000501053 0.05950476 0.004216871 0.034323804 0.018149508 0.02974476 0.018282323 -0.024190265 0.042797596 -0.014187863 0.034885172 0.01384961 0.036011007 -0.022787525 0.005545031 -0.011950304

sample_141 -2.733151727 -3.182910791 -1.114368398 0.214121293 -3.308228169 1.808147055 -0.371747975 0.687284391 -0.517314318 -0.563448458 0.165233275 -0.251918818 -1.462242296 -0.188036038 0.9931416 0.231000071 0.922140716 0.552257202 -0.018115818 -0.434401158 0.282733227 0.166264097 -0.78204667 -0.152370888 -0.524026558 0.749476514 -0.054006937 0.450219322 -0.374416367 -0.597861329 0.07682734 -0.000650259 -0.26938016 -0.010765833 0.055964309 -0.246592557 0.289173949 0.059856626 -0.312974354 -0.11170081 -0.500339444 0.176956262 0.13670833 0.122745273 0.122431571 0.109063278 -0.350327793 -0.205885586 0.019087341 -0.172148127 -0.234468306 0.119280138 -0.090930194 0.231834945 0.109276918 -0.042783885 -0.002312313 0.335817003 0.262745593 0.128455307 0.013418082 0.159662856 0.381999954 -0.076756438 -0.185088586 -0.172269922 -0.056303122 -0.15422908 -0.045992625 0.186157649 -0.161458773 0.207627346 0.131490164 0.112921243 -0.072269395 0.017588484 -0.088277792 -0.144090044 -0.186542544 -0.12952519 -0.226816075 -0.20392371 0.109931764 -0.166864471 0.015696005 -0.144828665 -0.030105284 -0.099030791 0.019926633 -0.202830705 0.022638847 -0.032031497 -0.042261714 0.035213586 0.01147238 0.036820981 0.171823372 -0.189308906 0.03879369 0.114679382 0.13051747 -0.058848301 -0.018676573 0.093846816 -0.027264478 -0.123798971 0.038464565 0.164728184 -0.034110131 -0.041805026 0.017984619 -0.10689349 0.085217479 0.101302489 0.034068941 0.076261684 0.015421605 -0.101638183 -0.116175948 -0.104628866 -0.005795722 -0.103110683 0.070158784 0.048532427 -0.049888058 0.180293303 0.060745616 0.04677932 -0.008147401 -0.149976404 0.011107003 -0.10260289 -0.015265027 -0.085048639 -0.062404393 0.083499398 0.056865987 -0.038621891 0.018723024 -0.036086844 0.014287374 0.085128528 0.080867721 0.035822887 0.078036041 -0.023136742 -0.143782019 -0.084423769 0.025271077 0.037996875 -0.008197483 -0.109570714 0.042924521 0.063637942 0.160378186 -0.020765974 0.103451313 0.017590604 0.005669415 0.042925552 -0.002451234 0.001066484 -0.025002413 0.111068679 0.01211133 0.048679529 -0.069189194 -0.098227345 0.092689116 0.057458529 -0.06401561 0.020485746 -0.109775926 0.104005422 0.057405037 0.018834682 0.127456737 0.041421233 0.052227204 -0.008863967 -0.038605204 0.074419326 0.03536257 0.055585562 -0.033816418 0.016998847 -0.120008214 -0.036685071 -0.103669434 -0.100947543 -0.027215975 0.087572157 0.065764701 -0.021616696 -0.021518935 -0.033534786 0.013895228 -0.071539056 -0.025183606 0.002313876 -0.051612918 0.021577148 0.012726789 -0.065946901 0.125184324 0.049298348 0.026374424 0.046203771 -0.026535429 -0.030438102

sample_142 -2.052365819 -0.712323852 -0.800794686 0.235434051 -2.11648103 0.408592969 -0.382758144 1.656082549 -0.125046255 -1.22372285 -0.798056451 0.884983205 -0.539557168 -0.164425593 0.389921657 0.163091252 -0.133585562 -0.54137422 0.415151294 0.018196222 -0.284964578 -0.245795686 0.269280682 0.139580423 0.070063913 -0.621779385 0.38259413 0.071014007 -0.053056357 -0.173026946 -0.304776678 -0.06824511 -0.409825127 0.151970668 -0.281394353 0.007532361 0.05087155 0.041547096 0.118840114 -0.145820443 -0.065667266 0.537811596 0.52071575 -0.301710523 -0.009141999 0.182796774 -0.337791294 -0.023597551 0.075619225 -0.008507926 -0.00703369 0.384631123 -0.068615208 -0.171151097 -0.132242258 -0.046616145 0.025078555 0.018851147 0.091491084 -0.014221406 0.163766919 0.209891949 0.123079823 -0.056833294 -0.062058323 -0.025230688 -0.150388428 0.005618583 0.194144537 0.128624834 -0.104276155 -0.056757426 0.213127578 -0.045908873 0.008834697 0.040002267 0.167500993 -0.109855785 -0.010916429 -0.013822808 0.005256039 -0.016752703 0.091278213 0.009850732 -0.011456694 -0.114714284 -0.03989809 -0.141912051 -0.049933028 0.085570172 -0.046507242 -0.036871016 -0.184635436 -0.022724154 0.002479163 0.118041655 0.015091799 -0.060567487 0.200812645 -0.00624599 0.13194722 0.110607813 0.032790214 0.012417251 0.077040476 -0.044589512 0.049044787 0.042078933 -0.022578146 0.046913501 -0.02656823 0.006314123 0.106593552 0.072601149 0.039583659 0.005055878 0.103241383 0.015548377 -0.058432549 -0.210640024 0.068245148 -0.175616906 0.054437631 -0.12880182 -0.088379144 0.081736468 -0.013883409 0.042074856 0.007415135 -0.073172 0.16105956 0.063991529 -0.106520264 0.004025058 0.075307546 0.039775895 0.118707895 0.044351632 0.114568529 -0.092245405 -0.089318014 0.04557362 0.016231517 0.056955369 0.062671598 0.029481361 -0.040679074 -0.050950762 0.030337241 0.105132604 0.038332411 -0.063452597 0.035372057 0.072731896 0.010035012 0.068085262 0.003786116 0.023533723 0.005369859 0.03674058 0.057738276 -0.073888359 0.021673715 -0.033461511 -0.019963475 0.058693888 0.002093768 0.07504416 0.023459158 0.032514239 -0.017714929 -0.056707011 -0.004077653 -0.040251806 -0.022005645 0.047811001 0.094167805 0.019859263 0.004269491 0.034431034 -0.010142708 0.030206951 0.002024216 0.016375056 0.049612847 0.005638277 -0.025918936 -0.021571169 0.029264836 -0.026844993 -0.005134999 0.028697982 -0.020418046 0.064832282 0.031993602 -0.07805778 -0.055653386 0.001483864 -0.122349836 -0.023953525 -0.070901413 -0.00573141 0.021310947 0.002743397 -0.006590404 -0.029718098 0.029974841 -0.032249956 0.002437641 -0.010045178

sample_143 -5.660431445 1.106718087 1.213071188 1.852158678 0.257184207 -0.655519217 -1.057095808 -0.414529989 -0.948774334 0.15991621 -0.376772195 0.118112979 -0.104375682 -1.39982394 0.270995906 0.732284712 -0.116374226 0.117072641 -0.377056617 -0.015141989 -0.50086504 -0.383685091 -0.658381834 0.019066234 0.469852612 0.079882076 0.287057055 -0.019871168 -0.108645969 -0.02596219 -0.044960395 -0.21834665 -0.109340733 -0.148929102 -0.129880629 0.299593295 0.068280369 -0.10481216 -0.247367752 0.095885669 -0.043844447 -0.003066859 -0.038195908 0.085780719 0.188280324 -0.105476911 -0.078396035 -0.220667514 0.099453036 0.031564777 0.160121574 0.07004817 -0.051329429 0.083119799 -0.000338279 0.208842541 0.114002042 -0.149805444 -0.098563902 0.091036638 -0.006359564 0.136686661 0.095729105 -0.203955317 0.015860635 0.0440797 -0.063285664 -0.005974672 -0.078799327 0.100250284 0.03022671 0.049866288 -0.051244224 0.160980933 0.112775833 0.001410214 0.03653697 0.035075712 0.025642738 -0.139602373 0.245373366 0.036609099 0.028695289 -0.04633846 -0.025672458 0.124621548 0.107973134 0.039472476 0.06994112 0.143748696 0.095332115 -0.136156339 0.013425806 -0.058370266 -0.007900156 0.004670581 0.059012656 0.134110144 0.107700517 0.112049672 -0.017248721 0.061457771 -0.043302414 0.01539488 0.009642007 -0.138360406 -0.183754002 -0.033669031 -0.071555757 -0.062395946 0.034926359 -0.009367559 -0.036810028 0.102074501 -0.06116629 0.045589511 -0.11028635 0.024251445 -0.061419088 -0.007634814 -0.069243375 0.023975594 0.012270154 -0.047251933 -0.02200181 -0.054800547 0.034985673 0.046749287 -0.111769355 0.013594334 0.0090697 -0.086078061 0.136547448 -0.115705571 -0.045151764 0.027570109 -0.009846419 0.07988904 0.050224356 -0.01713894 -0.047322152 0.011890388 0.089066845 -0.055569993 0.007871044 -0.021805412 0.039852388 -0.053695848 -0.059290575 0.014957651 0.084175772 0.063553184 -0.019047039 -0.00703482 -0.001053728 0.037855127 -0.013490157 0.015328157 0.017753337 -0.12269604 -0.087295478 -0.029337861 0.012514821 -0.0348719 -0.01674475 -0.024238845 -0.014907514 -0.033270832 -0.00760121 0.00093455 0.021852753 0.001663196 -0.067717056 0.033421413 0.046200354 -0.043199875 0.025022522 0.036897385 -0.024398547 0.053560627 0.127298416 -0.021819712 0.009374151 0.019925884 0.008806958 0.022852604 -0.003589467 0.04262317 0.022696766 -0.027152442 0.002699686 -0.010491119 0.031786941 -0.054114007 0.026963056 -0.022454914 0.004859859 -0.025517667 0.026332255 0.019021027 0.013827923 -0.005838911 -0.038060954 0.024541217 0.041599443 0.010704416 0.049332048 -0.030377176 0.015375088 0.043124234

sample_144 -3.531621763 -1.62914971 -2.65118108 0.744735989 -1.110402423 1.63038221 0.638006042 0.849338528 -0.82885063 -1.396594389 -0.483273915 -0.447649942 -1.419741854 0.541759482 -0.093391032 0.665348374 0.31726219 0.617599278 -0.318728695 0.54890774 -0.387627962 -0.264632144 0.014935576 -0.107648524 -0.156039037 -0.089139127 0.088355874 -0.029939669 -0.044873906 -0.010860169 -0.111517069 0.325098015 -0.191759259 -0.095227861 -0.584071468 0.053357615 0.17216356 0.188289889 0.387487578 -0.36122799 -0.184183265 0.193260051 0.086707938 -0.147206963 0.218199077 -0.029642104 0.08575276 -0.061103748 -0.196037 0.004464987 -0.178321972 0.195970513 -0.064864927 0.419195645 -0.073208505 0.028541995 -0.048439421 0.09296574 0.214122294 -0.093427988 -0.102734857 0.256806726 -0.187583459 0.062220787 -0.091573619 -0.018147274 -0.049857279 -0.106509101 -0.088373349 0.072470869 -0.058428501 0.162535462 0.15582821 0.168050182 0.109585017 -0.248866198 0.17782549 0.03740964 -0.109633549 -0.082851151 -0.10436326 0.036490625 0.034664942 -0.201073319 0.123808658 -0.000104476 -0.217521351 -0.031149849 0.014420735 -0.114534656 0.037823057 -0.072824596 -0.00794454 -0.00291459 0.014632984 -0.017899945 0.028027836 -0.108274352 -0.001438737 0.077951081 -0.004507016 0.011642407 0.049364422 -0.007961594 -0.035686885 0.003058966 -0.010733163 0.020546865 -0.009571914 -0.016635231 0.138265776 -0.028360402 0.016029712 0.000963982 -0.041588563 0.067924017 -0.006255985 -0.080795993 -0.080657468 0.098551669 0.067496532 0.001248956 -0.031582277 -0.028994867 -0.058538225 0.115108805 -0.028498816 -0.051497047 0.110867706 -0.16696851 -0.03903681 0.066956792 -0.028656475 0.062301043 0.214326331 0.019825805 -0.029357597 -0.143333143 0.04110246 0.035140966 0.005602908 0.070658757 0.059865397 0.028064284 0.028964825 0.048768432 -0.072576498 -0.102764031 0.00466579 0.00835118 0.114641334 -0.112980011 -0.065321121 0.045586073 0.076541164 -0.05005357 0.044135434 -0.035978554 -0.018941619 -0.022767259 0.010589145 -0.003761023 0.015538091 -0.003164836 0.086026894 -0.034021784 -0.021137717 -0.088592113 0.020091439 -0.066765664 -0.001711618 -0.063296718 -0.061115794 0.097601277 0.034938753 0.014894716 0.064066566 -0.043750835 0.008179582 0.017489916 0.103429159 0.011209891 -0.006646263 0.02626069 -0.095417837 -0.00141609 0.0195834 0.087442138 -0.02117329 0.021569939 -0.028389217 0.082030018 0.018898882 0.012059221 -0.018348224 -0.038514106 -0.011553666 0.006096261 0.045955636 0.025909938 0.002272585 0.046900946 -0.067264757 -0.041980251 0.038614449 -0.025498475 0.019258122 0.040961312 -0.004296416 0.033820827

sample_145 5.032657997 3.840136012 4.104060927 -2.54342227 -2.117655938 0.848107555 0.661552224 1.060816572 -0.170218357 1.237000448 -0.255353985 -1.371159411 -1.591934239 -0.580564518 0.876226251 0.804191701 1.337051732 -0.131780825 3.149646733 -1.577368706 1.70439517 0.499472483 -0.379087659 -0.618574829 -1.564609314 0.421407441 -0.293532019 -0.326813747 -0.001443671 -0.162068365 0.17535786 -0.00678978 0.141764725 1.060774294 -0.695427814 -0.682593992 -0.164544457 -1.165976494 0.070006859 0.367187426 -1.344658746 -0.561612469 0.112592906 -0.300450952 -0.253742174 0.479704999 0.290581813 0.087266469 0.382328853 0.576011441 -0.521386252 -0.458505173 -0.541081195 0.525745675 0.248027302 0.458208247 -0.321291388 0.002099032 -0.013172962 -0.377890037 -0.111401723 0.691386713 0.512113404 1.117200449 -0.488780905 0.050329526 0.017847353 0.104927789 -0.50915397 0.372142839 -0.063617185 -0.195274707 0.570340153 -0.151173307 0.323276171 0.213634177 -0.02668627 0.139457232 0.248549841 -0.087703112 0.49777076 -0.405333657 0.156344319 -0.104384691 0.286490971 -0.491009112 0.119900463 -0.514982531 -0.537938182 -0.623567104 0.206922735 0.346565708 0.385481368 -0.383400198 0.043822765 0.156019172 0.237316253 -0.054068182 -0.038436898 -0.054354149 -0.309229895 -0.276632714 -0.471806656 -0.290061237 -0.204996334 -0.399873584 0.270632167 -0.752861652 0.368638578 -0.047189368 0.53649472 0.567069839 -0.07197756 -0.089323355 0.215102295 -0.711063357 0.311469031 0.400223161 -0.087019176 -0.308415382 0.064759487 -0.419084964 -0.013317412 0.284373307 -0.014669158 0.121704915 0.444461021 -0.446155049 0.374460056 0.729832552 -0.005853299 0.130085716 -0.275138695 -0.167434123 0.604318864 -0.018665834 0.080107992 0.134519484 0.409196106 0.141110849 0.280568842 -0.075200613 0.249888575 0.105240716 -0.190356924 -0.221895586 -0.075622848 -0.019781657 0.052117825 -0.261073418 -0.124763647 0.276159512 -0.111878677 0.265063412 0.097128923 0.169363572 0.262772708 -0.247318661 -0.013640623 0.039812397 -0.283395192 -0.06061917 -0.0620869 -0.51285024 0.088306666 -0.279017092 -0.067206741 -0.40469676 0.001851094 -0.155035631 0.075201068 -0.246999499 0.103883225 0.37418472 0.034923767 0.150534609 0.039363114 0.383619347 -0.378598474 -0.006799578 -0.319191824 -0.05381232 0.008717164 0.043084863 0.092425407 -0.170676454 -0.241468115 -0.133610115 -0.269913035 -0.181575321 0.046943516 -0.372107516 -0.078284463 0.103349683 0.100545407 -0.36209055 -0.242889592 -0.267640995 -0.047819083 0.203311815 -0.404532932 -0.247045497 0.027550105 0.006075283 -0.144394514 0.03672766 0.121066429 0.231450853 -0.071413646 0.176193838

sample_146 8.660757941 1.58415557 4.856234787 0.054282344 0.982384557 -1.56946623 0.04865545 1.796283019 -0.557523957 -0.139545401 -0.955764714 0.955930496 -0.450142864 -0.023650006 0.452220131 0.63628469 -0.1656229 0.146644745 0.583932046 0.532009729 0.406831096 0.125447986 -0.18738377 0.313440728 -0.09920773 0.65459171 0.123473027 -0.598699667 0.160517317 0.95338742 -0.217755814 -0.239119901 0.113417194 -0.667793625 0.201310897 0.012603687 -0.121436015 -0.020305264 0.277860324 -0.699066513 -0.546878088 -0.034566576 0.162850058 0.327317827 -0.25073434 -0.048809438 0.18733962 0.121694435 -0.312966085 -0.381855126 0.0617103 -0.216039369 -0.048395462 0.001727848 -0.585833175 -0.08491827 -0.08694524 0.356105723 -0.132058099 -0.266349208 -0.126842362 0.006655639 0.027692685 0.210307253 -0.213991114 0.001735668 0.359858482 0.090824503 0.246165604 0.15407469 -0.028905063 0.046943841 -0.197964177 0.029902147 -0.168113694 -0.154837579 -0.027950904 -0.049226214 -0.077102784 -0.055638809 0.088535011 -0.003118187 0.08214451 0.264830248 -0.222321824 0.09328223 0.168570923 -0.049664299 0.015594759 -0.161670612 -0.140209979 0.01320932 0.11218988 -0.043709711 0.044485454 0.08196053 0.047730379 -0.056344609 0.100668438 0.106835749 -0.00260729 -0.178748715 -0.320387602 -0.218813705 0.041083517 -0.093156941 0.082896176 0.049427885 -0.020602195 0.129496564 -0.002254976 -0.206350017 0.041323586 -0.081170976 -0.071992204 0.003783745 -0.010006599 0.113033724 0.006731642 0.032164077 -0.10978939 0.131223945 0.016383534 0.066997741 0.037975439 -0.034830192 0.103256815 0.070978551 0.053620029 -0.054813065 -0.162008123 -0.122695678 -0.141643728 0.080826782 0.028481474 -0.025938201 -0.021228486 -0.161535575 -0.100324589 0.088179219 -0.03939378 -0.044473633 -0.075474425 0.023023114 -0.027647717 -0.048614055 -0.100592707 0.020182984 0.049101457 -0.004516473 -0.000275232 0.073775856 0.038268586 -0.028097204 0.019746311 -0.011063546 0.00403868 0.086472437 -0.087501816 -0.101416082 0.0594295 0.009972972 -0.046324582 0.038991934 0.067884318 0.099121589 -0.055334343 0.014351803 0.033165347 -0.040682539 0.063643285 0.034603111 -0.037539047 0.053697751 -0.051570877 -0.008807879 -0.004324274 0.079198714 0.095265367 0.057214206 0.064062412 -0.006978482 -0.077346097 0.041565098 0.005082677 -0.084109717 0.084675964 0.011919906 -0.024721801 -0.049798744 -0.031423923 0.027238317 0.013321896 -0.035279105 -0.050417945 -0.091315361 -0.01944385 0.017666069 -0.002518581 -0.001925418 0.016527243 -0.053791107 0.001750926 -0.020658161 -0.005827614 -0.006396175 -0.063253824 -0.037335265 -0.043546308 -0.001630328

sample_147 -0.476484019 3.39180954 -0.960372551 0.02893415 -1.128936749 0.350964562 -1.311177413 1.883691752 -0.090420031 -1.301219966 -0.012483422 0.622260513 -0.553724079 -0.185678015 0.401410079 0.578915914 -0.25918495 0.160846738 0.357423507 0.071608585 -0.275632308 0.617199287 -0.284380289 -0.554303307 0.267220028 -0.286476036 -0.012646414 -0.286644347 0.087738258 -0.068864778 -1.039704933 -0.074779649 -0.682350743 -0.25604473 0.230621768 0.052575532 0.010736078 -0.09993168 -0.013619559 0.204090993 -0.223437333 0.143830919 -0.008709847 -0.495010228 0.695990892 0.1116855 -0.435338447 0.29130044 -0.06485213 -0.027978995 -0.224150313 0.162408666 -0.330695351 -0.193369923 -0.114283055 -0.308139503 -0.013154978 -0.228505721 -0.023327692 -0.201413898 -0.087243838 0.039056301 -0.336643141 -0.063011594 0.13912727 -0.004298551 -0.137387756 0.131477984 0.170781425 0.14682311 -0.039998629 0.227563259 0.20294185 -0.265147491 0.01141877 0.226254422 0.213705924 -0.227282494 -0.003445033 0.117748958 -0.056193358 -0.07809065 0.216659366 -0.187490357 0.338014083 -0.034502173 -0.037473514 0.106847703 -0.01888068 0.130510887 -0.024078517 0.124278684 -0.046277908 -0.059873381 0.128157601 0.138895016 -0.217817369 0.137594341 0.331205144 -0.089013296 -0.036428936 0.044990424 -0.036573929 0.043879862 0.118062663 0.14964397 -0.020068358 0.001298735 0.081280784 0.069358047 0.119100788 0.054753711 0.044393495 -0.078881554 0.048708482 0.089212357 0.244101548 0.177210932 0.071929218 -0.182354954 -0.121349634 0.076000476 0.085023127 -0.046037677 0.055893755 0.001513103 -0.054087188 0.126969017 0.030862388 -0.04751173 0.019130091 -0.014669192 0.072175585 0.034529564 -0.015870393 0.147790673 0.136093811 0.026974592 0.061817787 -0.021707807 -0.037167916 0.003632793 0.009288567 0.061157401 -0.047773069 0.017854898 -0.058829675 0.039513065 0.011386842 0.124010838 0.07020437 0.052350845 0.054875732 -0.012380971 0.177731861 0.023285593 -0.022713865 -0.061685862 -0.075691167 -0.043528449 0.040378385 -0.024570111 0.120306724 0.076823802 0.074837934 0.047697285 -0.022884316 0.050782488 -0.026979782 0.030629331 0.036141356 -0.031911541 -0.042495223 0.097034495 -0.060037466 0.017156098 0.042828545 -0.040511629 0.106646105 -0.008773529 0.092424023 -0.040483345 0.001332703 -0.034870232 0.065188868 -0.093704805 -0.096613352 0.013343954 0.013068165 0.034953351 0.041989038 0.007251787 -0.02490175 0.097923065 -0.004750979 -0.050980775 0.02717983 0.021226425 0.020043599 0.020415024 -0.098971418 0.029672891 -0.041833916 0.005938691 0.020169982 0.071232137 0.053519242 -0.058269987 0.023253498 -0.009449316

sample_148 -1.055649649 -1.341518795 -0.957152321 -0.693710412 -0.749838757 0.942494472 -0.590705615 0.46807015 -1.688624983 0.126092844 -0.985888632 0.086987199 -0.704653946 -0.824048435 0.147926543 0.293149695 -0.496313061 0.391939651 0.812674094 0.339971381 0.307531191 -0.1705412 -0.096839349 -0.06987328 -0.383931431 -0.330449784 0.187562021 0.415301051 0.061387939 0.036655196 -0.564953102 0.369066488 -0.06082523 0.048891014 -0.468454193 0.404955674 0.050904053 0.071062519 0.309333823 -0.267314169 -0.102797545 0.13743832 0.598981511 -0.280593845 0.471126502 -0.058023943 0.086216912 0.124689629 0.063284713 -0.122774797 0.026740849 0.104219442 -0.036807917 -0.026275626 -0.041207518 -0.025295973 -0.038752654 0.125866378 0.232731088 -0.013033956 -0.128034447 0.167309977 -0.013368513 0.123014227 -0.15418527 0.024317191 0.017180853 -0.210585685 -0.011555837 -0.068606458 -0.035561093 0.046874528 0.211365957 0.105594479 -0.156644738 0.041333708 0.142899281 -0.086891167 0.146108642 -0.156653917 0.188574348 -0.099090197 -0.022424661 0.073194256 0.029753224 -0.104415821 -0.013666734 -0.051917755 -0.121887689 0.138168646 -0.043355115 -0.008756972 0.036596043 0.032723481 -0.04015186 0.033701315 -0.166260177 -0.024453281 0.019829202 -0.011158819 -0.004937014 0.063704216 0.00335272 -0.07165647 -0.091177726 -0.017261352 -0.048315829 0.057267183 0.059534257 -0.059700299 0.004005051 0.031822531 0.023582947 -0.05149684 0.023989167 0.026422023 0.116545979 -0.066785566 -0.10140791 -0.054686308 0.06613445 -0.057826835 0.050733121 -0.034283433 -0.104742375 0.029899924 0.107705654 0.052542333 -0.064178569 -0.08102466 0.02089825 -0.047657642 0.085910532 0.055975502 0.164688531 -0.005534269 0.086257797 0.075635408 -0.023560072 -0.023465301 -0.043594107 0.08320714 -0.02334872 0.033472966 0.098256988 0.042611647 0.061923801 -0.043452385 -0.02799254 0.044234326 -0.038127512 -0.032614008 -0.03349535 0.086062081 -0.059408173 0.001022643 0.060603225 -0.009721504 0.061611337 0.047395938 0.009659911 -0.089020669 -0.011476699 -0.003076714 0.006078987 0.06685557 -0.082177418 -0.008368574 0.031995868 -0.028062064 -0.020333613 -0.002010046 0.019340249 -0.049888837 -0.006309869 0.042063093 0.064362808 -0.043907718 -0.020920885 0.038278409 0.017352839 -0.017615639 0.02895836 0.035483517 -0.106449388 0.015158055 -0.048405824 0.029952121 -0.031615928 0.026205538 0.012238749 0.017286494 0.037595892 0.059982749 -0.013846025 -0.002725641 0.019234963 -0.061060002 -0.032903779 0.016534585 -0.004560048 0.02576513 0.034394305 0.058638352 0.011006374 -0.046837427 0.067771363 0.028293734 -0.038146604 0.002454353

sample_149 6.460237625 1.26617812 -4.757386071 -1.104886093 1.406276109 2.165262447 -2.928244171 -0.633584998 -1.151866781 -0.408614406 -0.936044204 -1.008458192 -0.578569609 -0.457338931 -0.439733454 -0.489409659 0.576823801 0.168560119 0.168866261 -0.674485572 0.15282732 0.369558522 0.341913724 0.258618244 0.548672044 0.383487712 -0.248150265 -0.392065488 0.643763805 -0.156295779 -0.731837531 -0.713180827 0.121820483 0.304109287 -0.120518793 0.716077618 0.195198106 0.083568888 0.067879444 0.03927987 0.02567653 -0.046811026 -0.376345282 -0.543041902 0.567779068 0.014286903 -0.344360255 0.112023361 -0.121791823 0.188691434 0.091767309 -0.018583289 -0.213252199 -0.32392186 0.029432218 -0.029078275 -0.04914864 0.410472178 0.098951646 -0.262448244 0.10386647 -0.083470548 0.082394831 -0.121801806 -0.050547134 0.354241885 0.050610808 0.121325506 0.071820179 0.087288307 0.312067929 0.48224522 0.118746907 -0.040362951 -0.164637438 0.151887505 -0.217411934 -0.043471635 0.275941057 -0.091478395 -0.157679302 0.106915825 0.150561613 -0.036299639 0.008923323 -0.235579926 -0.206218313 0.136538097 -0.140797158 -0.038341468 0.077111761 0.155160729 0.055269053 -0.060252063 -0.160612709 0.128659371 0.032595301 -0.091976863 -0.169657519 -0.039783298 0.037500413 -0.045308553 -0.073736345 -0.040053983 0.156622361 0.019036578 0.055135396 0.118661157 0.126891674 -0.168283068 -0.042962593 0.117720328 0.103386171 -0.227564522 0.118930673 0.018529044 -0.063043206 0.170926602 -0.090421743 0.03793987 -0.013559661 0.010740032 0.154048069 0.066019624 -0.06322141 0.105585368 0.017215012 -0.078478219 -0.083626407 0.078705164 -0.078443094 -0.149148145 0.006987326 0.13934059 0.096660385 -0.032964213 -0.129557851 -0.014272091 0.105216673 -0.110321169 -0.011023427 0.022034248 0.135408272 0.070014825 0.082574259 0.095676022 0.009656009 0.089965573 0.057471786 -0.06532588 -0.103491485 0.011778238 0.015951088 0.002547165 0.084704485 0.040795617 -0.070477254 -0.04080848 -0.104187613 -0.086903712 0.081349774 0.033854312 -0.039037512 -0.101467312 -0.010680423 0.099467307 -0.01345592 0.051986972 -0.156051126 -0.055933356 -0.023306098 -0.067980914 -0.153082841 -0.060491767 0.045306427 0.030672804 0.200193021 0.043721558 -0.074855121 0.113448524 -0.067426586 0.014622741 0.007491713 -0.042009162 0.034958775 0.024680834 -0.021981074 -0.069087276 -0.019051707 -0.068886407 -0.03805355 0.007162363 0.090527218 0.003939208 0.00600121 -0.030092368 -0.040545492 -0.115998352 -0.047781201 -0.0839252 -0.034423687 0.033938574 -0.030045341 0.043858722 -0.056226809 0.031869183 0.043425249 0.012384827 0.024277444 -0.012664964

sample_150 -2.237450968 -1.173963441 -0.714939407 2.86485552 -3.62010115 1.008702171 -0.318139302 1.179595878 0.203013733 -1.47594052 -0.628773049 -0.525574952 -0.692600245 -0.457179414 -0.168542065 0.33102489 -0.56124921 0.375756219 -0.41179152 -0.963898577 0.584701103 0.023469956 -0.223838368 -0.053595729 -0.124658853 0.313517556 0.032097378 0.275667941 -0.322777693 -0.109012051 0.368911452 1.061620358 -0.293277933 0.596742802 0.175634785 0.459827589 -0.288454376 0.440867718 -0.182337425 -0.376572078 -0.107298674 0.296109514 -0.26376632 -0.001067638 0.00844218 0.059963413 0.064631979 -0.376151508 0.181039412 0.444614966 0.047102004 0.220030275 0.010004366 0.113080927 -0.082901395 -0.401631761 0.023297455 0.222907939 0.143368313 -0.362844722 -0.123611022 0.102452658 0.190723366 -0.137981714 -0.064614246 -0.129247533 -0.016994616 -0.001303013 0.087174082 0.095485755 0.053712605 0.084404464 -0.089473292 0.084955093 0.288508182 -0.133321225 -0.067602738 -0.171120563 0.20707144 -0.453502743 -0.0428274 -0.080545397 0.097309226 -0.17580326 -0.062778621 -3.18E-05 -0.084668845 -0.302648549 -0.009830758 -0.165427438 -0.171184443 0.025574075 0.083212986 0.093290294 -0.115976308 -0.106806159 -0.028465744 -0.020350476 0.085641086 -0.092312661 0.008395649 -0.027997307 0.18185158 -0.075796406 -0.103094215 -0.059578275 -0.059024671 0.103648075 -0.01299421 -0.070874296 0.0625849 0.114380922 0.029382183 0.077295893 -0.10497128 -0.016782861 -0.033076866 0.162090125 0.032988597 -0.125475536 0.005881134 -0.073097706 -0.09277428 -0.056152938 -0.028626388 0.091477845 -0.03592705 0.04540749 -0.019324426 -0.013103659 0.107284483 0.105395663 -0.07486344 -0.018770252 -0.023736732 0.047995998 -0.096640073 -0.007593949 0.028127415 0.008481538 0.019623513 0.065257179 0.112229626 0.063025685 0.048911026 -0.05081478 -0.029471905 -0.04499007 -0.018964886 -0.046918404 -0.10596645 -0.092404441 -0.01356135 0.098134576 0.130276029 0.006928329 -0.070244007 0.119274554 -0.029013898 -0.026116434 0.033203983 0.064355041 0.000270654 0.119579348 0.072081276 0.026549622 0.047146262 -0.026938898 0.104435721 -0.058651082 -0.086562266 -0.055845442 -0.03469002 0.084550249 -0.002371591 0.082901529 0.129049362 0.041326262 0.011036798 -0.038435429 -0.082874012 0.062707847 0.015425185 0.052667729 0.020342667 -0.025344957 0.002799744 0.019078796 -0.122732521 -0.07203973 -0.009353145 -0.01068522 -0.007677289 -0.029952545 -0.008415156 0.01239247 -0.046632555 -0.039127596 0.010566591 0.028510029 0.058671014 0.05095669 0.008029804 -0.021324816 0.000109165 0.007321229 -0.049674502 -0.056997691 0.044722905 -0.092590767

sample_151 3.223326806 -6.549711203 3.496942058 -0.070523394 0.253539469 -1.621409241 1.557399235 -0.729498172 1.117111423 -0.91709021 0.537913067 -0.128068441 0.502936912 0.330217751 -0.706939052 -0.349835598 0.246269774 -0.122701067 -0.088789754 0.182796309 -0.046309211 -0.020635988 0.227023661 0.066298887 0.409285525 -1.043507617 -0.664978457 0.772482402 -0.315311638 -0.25208971 -0.042274726 -0.497108631 0.06001605 0.337100538 -0.129583533 0.346072014 0.164104669 0.052714898 -0.227116345 -0.031355358 0.047509321 0.133886859 -0.204136422 -0.226527899 0.431391504 0.217508906 -0.022981224 -0.118150443 -0.090485779 -0.087562953 0.044262517 -0.15486288 0.101382945 -0.174996717 0.230744925 -0.130178611 -0.165837788 -0.15915672 -0.257333915 0.028629538 -0.140179027 -0.005181433 -0.006957963 -0.08125792 0.173740573 -0.059548915 -0.087727328 -0.132519665 0.144121061 0.063209584 0.032599642 0.050727458 0.303660884 -0.143185386 0.138505973 0.146761653 -0.187509577 -0.124974441 -0.201841023 -0.081730825 0.060751531 0.036065107 -0.126191112 -0.054433503 0.091026049 -0.024088131 0.108403416 0.14337646 0.059794739 0.095833349 0.116638092 -0.026202683 0.025860652 -0.03870825 -0.164871532 -0.154319884 0.080028706 0.093094682 0.040069012 0.00411788 0.037076941 0.05985426 -0.012907572 -0.065004216 -0.045054181 0.026364046 0.099167409 -0.129474767 -0.126919475 0.056586136 -0.095263824 0.074626023 -0.258504178 -0.002491699 -0.094865114 0.047888335 0.130117765 -0.073939398 -0.051443355 -0.082074302 0.006241218 0.004646131 -0.004468815 0.098451609 -0.108518163 -0.039175389 -0.03681285 -0.097734391 -0.094811157 -0.177331975 0.06398077 0.069754901 0.131626751 0.076771475 -0.036105382 -0.029266596 0.033099093 0.025053757 0.001349916 0.136291552 0.040649389 -0.004682952 -0.02854118 0.018749571 0.011394128 0.043027389 -0.02146356 -0.010069973 -0.021623023 -0.010213167 0.075700439 -0.034294475 0.015174018 0.087739764 -0.014414426 -0.041551561 0.119195551 -0.002384549 -0.144528045 -0.000247047 -0.113126122 -0.091080606 0.018755908 -0.015242982 -0.056287006 0.002191174 0.025922489 -0.081652848 0.045629854 0.091632096 0.035774427 0.020955163 0.001805741 -0.05610632 0.01345482 0.028272249 -0.024201368 -0.074475506 0.106879382 -0.017973071 -0.011957515 0.011026989 -0.054819658 0.003416114 -0.002472954 0.032464851 0.141465769 0.011792939 -0.035745572 0.075656232 0.055781831 0.063402971 -0.019065389 0.043239888 -0.052800344 0.154562654 0.045634715 -0.061131293 -0.009265555 0.04860292 -0.035831012 -0.133257922 0.055718086 0.079810547 -0.052012853 0.003558873 0.007928193 0.029045556 0.030603557 0.025293864

sample_152 7.027257376 2.435751464 -5.72495588 0.630441547 0.789130595 3.596085717 -2.568130714 4.236217026 1.257847605 -0.851623082 0.627438484 0.490587183 1.335443364 -2.442287364 0.985708991 -1.200112796 -0.242989731 0.999112198 0.598978295 -1.070740539 -1.636550798 0.307811898 -0.506288071 0.298522927 -0.5014327 -0.021346533 0.997423889 -0.266842004 -0.135391004 -0.791372688 0.098748397 -1.062471695 -0.222984388 -0.000773543 -0.144585813 0.395157679 -0.423155061 -0.331440753 0.377146139 0.656724389 -0.042070349 -0.394866645 0.544290416 -0.000917835 1.166251241 0.1248872 0.243456982 -0.265829233 -0.055860597 0.319538029 -0.184020937 -0.281500792 -0.236398448 0.236433113 0.537048417 0.427998192 0.169224597 0.697053373 -0.283910676 -0.031069336 -0.032142493 -0.381834374 -0.055054918 0.395196643 -0.341505229 0.20310161 -0.343813694 0.160142181 0.246747755 0.171802874 0.029273075 -0.363951173 0.04084127 0.185093275 -0.104036469 0.425821885 -0.20537047 0.037821781 -0.199186323 0.152419627 0.28622796 -0.07852316 0.189676632 -0.528404307 -0.203007327 -0.467801958 0.046755846 0.055152596 -0.196156526 0.059277883 0.341555302 0.121265644 -0.062395513 -0.196119306 0.054011668 0.01370288 0.110353892 0.179610346 0.124896209 -0.389254779 -0.025285687 -0.025115531 0.35663983 -0.109442171 0.143807143 0.139596889 -0.337396267 -0.407779276 -0.115126184 0.405502276 0.123588266 0.162302896 0.08780923 0.053714684 0.152510627 0.098743761 -0.039751621 0.050870228 0.012758181 -0.186474421 0.094006983 0.367218257 0.161083977 0.041618516 -0.004931128 -0.090402794 0.242432598 0.119454087 0.02817267 -0.186548664 -0.094554797 -0.045979325 0.22852857 0.465310675 -0.061034786 -0.079810662 -0.118039396 0.370833883 -0.081351013 0.312206684 -0.011725048 0.121732264 0.103129295 0.198996413 -0.046619383 -0.019370909 0.084383324 0.097129163 -0.021024779 0.11833133 0.056091945 0.200254325 -0.166627669 0.315531544 -0.177085761 -0.19978145 0.151157211 0.12909629 -0.212172773 -0.06073205 -0.013927379 -0.09437815 -0.051041192 -0.036065798 -0.092267618 0.212682707 0.042380846 0.011194007 -0.102175819 0.051889876 0.2269971 0.015524736 -0.07812472 0.05803735 0.058158824 -0.210044509 0.022233562 -0.005549226 0.078212993 -0.210369424 -0.073695815 -0.168464523 0.015714113 -0.028420158 0.037331966 -0.016689123 -0.130785751 0.008362958 0.057763188 0.045049721 0.072774806 -0.000543968 -0.040275572 -0.026656459 0.20387893 -0.105558078 -0.098453339 0.016290567 -0.128233547 0.105878863 0.224765779 0.028402875 -0.065485308 -0.020713427 -0.076553699 0.041891736 -0.109877819 0.061365949 0.030369337 -0.089441326
[truncated: 4,842,854 more chars]
